# A High-Density Genetic Linkage Map and QTL Mapping for Sex and Growth-Related Traits of Large-Scale Loach (*Paramisgurnus dabryanus*)

**DOI:** 10.3389/fgene.2019.01023

**Published:** 2019-10-25

**Authors:** Jin Wei, Yuanyuan Chen, Weimin Wang

**Affiliations:** ^1^Key Lab of Agricultural Animal Genetics, College of Fisheries, Breeding and Reproduction of Ministry of Education/Key Lab of Freshwater Animal Breeding, Ministry of Agriculture, Huazhong Agricultural University, Wuhan, China; ^2^College of Fisheries, Huazhong Agricultural University, Wuhan, China

**Keywords:** large-scale loach, genetic linkage map, QTL mapping, growth, sex, potentially key genes, genome survey analysis, genome scaffold anchoring

## Abstract

Large-scale loach (*Paramisgurnus dabryanus*) is a commercially important species in East Asia; however, the cultured population that exhibited degradation of germplasm resource cannot meet the market needs, and the genome resources for *P. dabryanus* are still lacking. In this study, the first high-density genetic map of *P. dabryanus* was constructed using 15,830 SNP markers based on high-throughput sequencing with an improved SLAF-seq strategy. The quantitative trait locus (QTL) mapping for sex, growth, and morphology traits was performed for the first time. The genetic map spanned 4,657.64 cM in length with an average inter-marker distance of 0.30 cM. QTL mapping and association analysis identified eight QTLs of growth traits, nine QTLs of morphology traits, and five QTLs of sex-related traits, respectively. Interestingly, the most significant QTLs for almost all the traits were concentrated on the same linkage group LG11. Seven candidate markers and 12 potentially key genes, which were associated with sex determination and growth, were identified within the overlapped QTL regions on LG11. Further, the first genome survey analysis of *P. dabryanus* was performed which represents the first step toward fully decoding the *P. dabryanus* genome. The genome scaffolds were anchored to the high-density linkage map, spanning 960.27 Mb of *P. dabryanus* reference genome. The collinearity analysis revealed a high level of collinearity between the genetic map and the reference genome of *P. dabryanus*. Moreover, a certain degree of homology was observed between large-scale loach and zebrafish using comparative genomic analysis. The constructed high-density genetic map was an important basis for QTL fine mapping, genome assembly, and genome comparison. The present study will provide a valuable resource for future marker-assisted breeding, and further genetic and genomic researches in *P. dabryanus*.

## Introduction

Large-scale loach (*Paramisgurnus dabryanus*; *Cypriniformes*; *Cobitidae*), which distributes naturally in East Asia, is a small bottom-dwelling freshwater fish species (Li et al., 2015). This species has a diploid chromosome number of 48 (Li, 1983; You et al., 2009), and the cytogenetic evidence and result of sex ratios generally confirmed that the sex determination in large-scale loach was determined by the putative ZZ/ZW system (Chang and Yu, 1997; You et al., 2008); so, it is a promising model for studying the mechanisms of ZW/ZZ sex determination system. In addition, *P. dabryanus* has been recognized as a commercially important species suitable for freshwater aquaculture in China, Korea, and Japan due to its rapid growth rate, ease of artificial propagation, high nutritional value, and high economic value (Wang and Li, 2005a). The total production of loach reached 400,209 tons in 2016, and the year-on-year growth was up to 9.29% which ranked third of all freshwater fish in China (Ministry of Agriculture of the People’s Republic of China, 2017). As a result of long term high-intensity stocking and lack of resource management; however, the cultured population of *P. dabryanus* has been gradually exhibiting low growth rate, disease susceptibility, and precocious puberty (Wang et al., 2005; You et al., 2012; Li et al., 2015). Consequently, it is essential to establish a selective breeding scheme for large-scale loach to improve the economic traits and promote the development of large-scale loach industry. The traditional selective breeding based on phenotypes in this fish was affected by some unfavorable factors, such as long breeding cycle, low predictability, and low selection efficiency (Zenger et al., 2017). The marker-assisted selection (MAS) technology, which can overcome these disadvantages by using molecular markers tightly linked to the target QTL to assist phenotypic screening, provided a new approach for selected breeding in aquatic animal (Tong and Sun, 2015; Robledo et al., 2018). In recent years, the genetic linkage map and QTL mapping have become important MAS breeding tools, because of their abilities to identify molecular markers or candidate genes associated with traits (Sun et al., 2017; Feng et al., 2018).

Since the 1990s, the genetic linkage maps have been established in more than 40 aquaculture species, and QTL mapping for important traits (e.g., growth traits, sex determination, disease resistance, cold resistance, and salt tolerance) has been performed in over 20 aquaculture species (Tong and Sun, 2015). Most of the early genetic linkage maps were constructed by using labor-intensive or dominant markers such as RFLP (restriction fragment length polymorphism), AFLP (amplified fragment length polymorphism), RAPD (random amplified polymorphic DNA), and SSR (simple sequence repeats) (Cheng, et al., 2010; Yue, 2014; Tong and Sun, 2015). Traditional genetic marker systems have played important roles in genetic linkage map construction; however, unsatisfied marker density, dominant expression, and not repeatable in different maps have limited their ability of fine mapping as well as the identification of QTLs with important genetic traits (Guo et al., 2018; Zhang et al., 2019). SNP is the most abundant and stable form of genetic variation with a high polymorphism in most genomes (Leonforte et al., 2013). Comparing with other genetic markers, SNPs are the most suitable markers for high-density linkage map construction, despite a great challenge for the large-scale development of SNP and cost-effective genotyping for the non-model species with a large mapping population, such as aquaculture species. Recently, owing to the great advances of the next-generation sequencing (NGS) technology, an increasing number of high-density linkage maps have been constructed for aquaculture species with sequencing-based SNP genotyping strategies, including Yangtze River common carp (Feng et al., 2018), golden pompano (Zhang et al., 2018), pikeperch (Guo et al., 2018), large yellow croaker (Kong et al., 2019), and snapper (Ashton et al., 2019), *etc.*, which provided an important basis for further genetic researches on these species.

A sufficient number of high-quality molecular markers are essential for high-density linkage maps construction and fine QTL mapping (Jiao et al., 2014), as well as the resolution of a genetic map can be significantly improved by increasing the number of markers in a given mapping population (Zou et al., 2012). Several genotyping strategies for the large-scale population have emerged with the extensive application of NGS, such as whole-genome re-sequencing (Lijavetzky et al., 2007), restriction site associated DNA sequencing (RAD-seq) (Baird et al., 2008), genotyping-by-sequencing (GBS) (Poland and Rife, 2012), and specific-locus amplified fragment (SLAF) sequencing (Sun et al., 2013). A growing number of high-density SNP-based linkage maps have been constructed for the economically important aquaculture species using these techniques, including Atlantic salmon (Gonen et al., 2014; Houston et al., 2014), rainbow trout (Campbell et al., 2014), Nile tilapia (Palaiokostas et al., 2015a), channel catfish (Zhang et al., 2019), European sea bass (Palaiokostas et al., 2015b), turbot (Wang et al., 2015), and black tiger shrimp (Guo et al., 2019).

Recently developed SLAF-seq technology, which is based on the reduced representation library (RRL) strategy and high-throughput sequencing, can provide an economical and efficient solution for large-scale genotyping (Sun et al., 2013). Compared with other genotyping strategies, it has several advantages and characteristics: (i) ensure the genotyping accuracy by deep sequencing, (ii) reduce the sequencing costs by RRL strategy, (iii) optimize the marker efficiency by pre-designed RRL strategy scheme, and (iv) employ the double barcode system for large populations (Sun et al., 2013). SLAF-seq has been used for high-density genetic map construction in an increasing number of aquatic animals up to now, such as common carp (Sun et al., 2013), Pacific white shrimp (Yu et al., 2015), freshwater sleeper (Zhang et al., 2017), triangle sail mussel (Bai et al., 2016), Chinese mitten crab (Qiu et al., 2017), swimming crab (Lv et al., 2017), razor clam (Niu et al., 2017), pikeperch (Guo et al., 2018), and sea urchin (Chang et al., 2018). Previous studies indicated that SLAF-seq, which provides an efficient strategy for large-scale genotyping and high-density genetic map construction, can be generally applicable to various species and populations.

Due to the high economic value of *P. dabryanus*, great attention was focused on this species. Although some progress has already been made in biology, physiology, nutrition, artificial cultivation, and population genetics of it (Yin et al., 2008; You et al., 2008; You et al., 2009; Chu et al., 2012; You et al., 2012; Zhang et al., 2015), little is known about the genetic basis for most of the economic traits in large-scale loach. The high growth-rate variety would accelerate the turnover of production, and the fish with larger size generally attain a higher unit price compared to smaller ones in the market; therefore, growth is considered as a trait of great economic importance for all species of aquaculture (Gjerde, 1986; Tong and Sun, 2015). Besides, the previous studies have reported the influence of gender on growth in *P. dabryanus* (Wang and Li, 2005b), and the effect of gonad development on the growth of aquaculture species (Jin et al., 2001). Both sex differentiation and gonad development, thus, are considered to be economically important traits.

The *de novo* sequencing of *P. dabryanus* genome has been finished in our lab (not published yet). The main objectives of this research were to construct the first high-density genetic map of *P. dabryanus*, perform fine-scale QTL mapping for growth and sex-related traits, identify the potentially key markers and genes linked to growth and sex, as well as assist the assembly of *P. dabryanus* genome.

## Materials and Methods

### Genome Survey Analysis and *De Novo* Assembly

A male large-scale loach from the wild population of Dongting Lake in Hunan Province of China was used for genome survey analysis. Genomic DNA was extracted from the muscle tissue. Two short paired-end DNA libraries with insert sizes of 270 base pairs (bp) were constructed from randomly fragmented genomic DNA. Sequencing was performed on the Illumina Hiseq 2500 PE150 platform (Illumina, San Diego, CA) by Beijing Biomarker Technologies Co., Ltd. (Beijing, China). After filtering and correcting, the raw sequence data, clean pair-end reads were obtained.

*De novo* genome assembly was conducted with SOAPdenovo software (http://soap.genomics.org.cn/soapdenovo.html) following the manufacturer's protocol. Subsequently, the scaffolds were constructed step by step using insert size paired-ends. The construction of a *de novo* repeat library was performed following the procedures described by Lv et al. (2017). The peak depth and the number of 21-mers were obtained based on K-mer analysis. The genome size was estimated with the following algorithm: Genome size = K-mer num/peak depth.

The *P. dabryanus* genome was subsequently sequenced, assembled, and annotated in our laboratory. We obtained the first high-quality draft genome of this species but not published yet.

### Mapping Population, Phenotypic Data Acquisition, and DNA Extraction

In this study, a F1 full-sib family was constructed with the cross between a fast-growing female *P. dabryanus* from the population of Hongze Lake in Jiangsu Province and a slow-growing male *P. dabryanus* from the population of Dongting Lake in Hunan Province of China. The previous study indicated that the *P. dabryanus* populations from the Hongze Lake and Dongting Lake show a high genetic differentiation (mean genetic differentiation coefficient, *F* *_st_* = 0.219 > 0.2) and a low genetic exchange (gene flow, *N* *_m_* < 1). The relatively high heterozygosities (mean observed heterozygosity, *H* *_o_* > 0.334; mean expected heterozygosity, *H* *_e_* > 0.563) indicated a high level of genetic diversity in two populations (Li et al., 2014). Due to the relatively high genetic polymorphism and genetic differentiation in both parents, we decided to construct a genetic linkage map using F1 mapping family with double pseudo-testcross strategy (Grattapaglia and Sederoﬀ, 1994). The genetic polymorphism of the F1 individuals was estimated using Arlequin ver 3.1 (Excoffier et al., 2006). The polymorphism information content (*PIC*) was 0.322, and the average *H* *_o_* and *H* *_e_* of the F1 population were 0.410 and 0.413, respectively.

Two parents and approximately 15,000 F1 individuals were reared in Aquaculture Base of College of Fisheries, Huazhong Agricultural University in Wuhan City, Hubei Province, China. One and a half years after hatching, a random collection of 719 F1 individuals were transferred to a separate tank for trait measurement. The seven growth traits and four morphology traits were measured with the method described by Yi et al. (2017). The growth traits include total weight (TW), full length (FL), body length (BL), body height (BH), body width (BW), head length (HL), and empty-body weight (EBW). EBW refers to the weight of an individual after removing all the scales, gills, and internal organs. The morphology traits include eye diameter (ED), caudal peduncle length (CPL), caudal peduncle height (CPH), and intestine length (IL). The sex-related traits include gender (GD, ♀/♂), weight of gonad (WG), maturation coefficient (MC), and stage of gonad development (SGD). MC is also known as the gonad index, which defined as the ratio of WG to TW (WG/TW×100%). SGD is determined according to the method described by Lei and Wang (1990). Pearson correlation analysis was performed to investigate the relationships among seven growth traits, four morphology traits, and three sex-related traits (include WG, MC, and SGD). Student's t-test was utilized to investigate the gender difference among the growth/morphology traits. Shapiro–Wilk normality test (Royston, 1995) was conducted with SPSS 19.0 software to investigate the trait distributions.

After the morphological measurement, we randomly selected 200 healthy individuals from the 719 progenies for sample collection. The muscle tissue of the two parents and 200 F1 individuals was collected and immediately stored in liquid nitrogen. Genomic DNA was extracted using the phenol-chloroform method in combination with RNase treatment. Gel electrophoresis and a NanoDrop 2000 Spectrophotometer were used to assess DNA quality and quantity. The DNA samples were stored at −20°C.

### SLAF Library Construction and High-Throughput Sequencing

An improved SLAF-seq strategy was adopted in the present study. A pre-experiment was conducted to evaluate the enzymes and restriction fragment sizes. The *P. dabryanus* reference genome, not yet publicly available, was chosen for *in silicon* enzyme digestion. An optimum scheme was determined and utilized to construct the SLAF library based on the results of the experiment. Two restriction enzymes (*RsaI* and *HaeIII*, New England Biolabs, USA) were used to digest the genomic DNA of all the samples. Then, a single nucleotide (A) overhang was added to the digested fragments by the Klenow fragment (3'→5' exon) (New England Biolabs, USA) and dATP at 37°C. Dual-index sequencing adapters (PAGE-purified, Life Technologies, Gaithersburg, MD, USA) were then ligated to the A-tailed fragments using T4 DNA ligase. Polymerase chain reaction (PCR) was performed in the reaction system containing diluted restriction-ligation samples, dNTP, Q5^®^ High-fidelity DNA polymerase, and PCR primers (forward primer: 5'-AATGATACGGCGACCACCGA-3,' reverse primer: 5'-CAAGCAGAAGACGGCATACG-3') (PAGE-purified, Life Technologies). PCR products were purified with Agencourt AMPure XP beads (Beckman Coulter, High Wycombe, UK) and pooled according to the manufacturer's manual. Pooled samples were then separated using 2% agarose gel electrophoresis. Fragments ranging from 364 to 444 bps (with indexes and adaptors) in size, which was defined as SLAF tags, were excised and purified using a QIAquick Gel Extraction Kit (Qiagen, Hilden, Germany) following the manufacturer's protocol. Subsequently, gel-purified products were diluted to equal concentration and then sequenced with the paired-end sequencing method (Each end 125 bp) on the Illumina HiSeq 2500 System (Illumina, Inc; San Diego, CA, USA) following the manufacturer's recommendations. Paired-end sequencing reads with a length of 100 bp×2 were used for further analysis and quality evaluation. The *Oryza sativa* genome (http://rice.plantbiology.msu.edu/) served as a control to evaluate the reliability and accuracy of this approach for SLAF library construction.

Real-time monitoring was performed for each cycle during sequencing progress for the sake of controlling the quality of the sequencing data. The ratio of raw high-quality reads with quality scores greater than Q30 (a quality score of 30, which indicates a 0.1% chance of obtaining an error and thus 99.9% confidence) and guanine-cytosine (GC) content were calculated and evaluated as two key indicators.

### SNP Discovery, Grouping, and Genotyping

Marker identification, grouping, and genotyping were performed following the procedures described by Sun et al. (2013). In brief, low-quality reads with quality score < 30 were filtered out, and then, the remaining raw reads were sorted to each sample according to duplex barcode sequences. All of the clean reads, whose barcodes and the terminal 5-bp positions were trimmed from high-quality reads, were aligned to the reference sequence released by the *P. dabryanus* genome using BWA (Burrows–Wheeler Aligner) software package (Li and Durbin, 2009). Sequences with over 90% identity were grouped and defined as one SLAF locus as described by Sun et al. (Guo et al., 2018). Alleles at each locus were defined using the minor allele frequency (MAF) evaluation. For diploid species, one SLAF locus can contain at most four genotypes (Guo et al., 2018). *P. dabryanus* is a typical diploid species (You et al., 2009). Thus, the SLAF loci with more than four alleles were defined as repetitive SLAFs and discarded subsequently in this study. Only SLAFs with two to four alleles were identified as polymorphic SLAFs (Sun et al., 2013).

SNP loci were identified among parents and offsprings using Genome Analysis Toolkit (GATK) (https://software.broadinstitute.org/gatk/documentation/) (DePristo et al., 2011). Polymorphic SNP markers were classified into eight segregation pattern categories (aa×bb, ab × cc, ab × cd, cc × ab, ef × eg, hk × hk, lm × ll, and nn × np). Owing to a double pseudo-test-cross strategy used in constructing the linkage map in this study, SNPs from seven segregation patterns (excluding aa × bb) were selected for genetic map construction. Genotype scoring was then conducted by a Bayesian approach to further ensure the genotyping quality (Sun et al., 2013).

To further improve the genotyping quality, several criteria was applied to filter these SNPs, including (1) the SNPs with a < 10-fold average sequencing depth in the parents were filtered out, (2) the polymorphic SNPs with < 75% integrity among the mapping population were filtered out, and (3) the chi-square test was conducted to examine the segregation distortion, and SNPs showing significant segregation distortion (P < 0.01) were excluded.

Subsequently, the SNP markers were further filtered using the sliding windows according to the location of the markers on the genome, which a SNP with the highest integrity and sequencing depth in the parents was retained within every 50 kb for genetic map construction. These identified SNP loci were finally separated into four segregation patterns, type lm × ll, nn × np, hk × hk, or ef × eg.

### Genetic Linkage Map Construction and Evaluation

To further confirm the robustness of markers for each linkage group (LG), modified logarithm of odds (MLOD) scores between high-quality polymorphic markers was calculated (Stam, 1993). Markers with MLOD score < 5 were filtered before ordering. Because of some inevitably genotyping errors in the markers, a newly developed HighMap software was employed for genetic linkage map construction, which would order the markers and correct genotyping errors within the LGs (Liu et al., 2014). Firstly, recombinant frequencies and logarithm of odds (LOD) scores, which would be applied to infer linkage phases, were calculated by two-point analysis. Subsequently, enhanced Gibbs sampling, spatial sampling and simulated annealing algorithms were combined to perform an iterative process of marker ordering (Jansen et al., 2001; Van Ooijen, 2011). The mapping algorithm was performed repeatedly until all markers had been mapped appropriately.

The error genotyping correction strategy of SMOOTH was then performed in accordance with the parental contribution of genotypes (van Os et al., 2005), and a k-nearest neighbor algorithm was adopted to impute missing genotypes (Huang et al., 2012).

A multipoint method of maximum likelihood was subsequently applied to add skewed markers to this map, and Kosambi mapping function was utilized to estimate the map distances (Kosambi, 2016).

The haplotype map and heat map were used to evaluate the quality of the constructed linkage map as described by Liu (2013). Since some markers showing significant (0.001 < P-value < 0.05) segregation distortion were still used for the construction of linkage genetic map, a region on the map with more than three consecutive adjacent loci that showed significant (0.001 < P-value < 0.05) segregation distortion was defined as a SDR (Fu et al., 2016). The distribution and size of SDRs on each LG were analyzed for evaluating the quality of the constructed linkage map.

### Genome Scaffold Anchoring and Comparative Genome Mapping

The SNP sequences of the sex-averaged map were mapped to the unpublished reference genome of large-scale loach by using Basic Local Alignment Search Tool (BLAST). The collinearity analyses of genetic map and draft genome were performed following the procedures described by Peng (2016).

Besides, the high-density genetic map of large-scale loach was used to perform comparative genomic analysis with the closely related model species. The genome sequences surrounding the SNP loci (200 bp around the center of SNP loci) were extracted from the *P. dabryanus* reference genome by in-house Perl scripts; then, the sequences were aligned with the genomic sequences of zebrafish (GRCz11) (https://www.ncbi.nlm.nih.gov/genome/?term=GRCz11) using BLASTX. The results of collinearity and comparative genomic analysis were visualized by RSCRIPT language following the tutorial introduction.

### QTL Mapping and Candidate Gene Identification

QTL analysis was performed for 15 traits using R/qtl package with composite interval mapping (CIM) algorithms according to the procedure described by Wang (Wang et al., 2018). The significance of each QTL interval was tested by LOD. The LOD score significance thresholds for QTL declaration were determined by permutation tests (1,000 replicates) at a significance threshold of 0.05. The QTLs with LOD scores higher than the significance threshold were declared significant. The phenotypic variance explained (PVE) by a QTL peak was calculated using 1–10 ^−2LOD/n^ (n = sample size) (Gardiner et al., 2012; McClure et al., 2016).

The genes located in the significant QTL regions were considered as candidate genes associated with target traits. Firstly, we identified the candidate markers in the QTL regions. Secondly, these markers were mapped onto the scaffolds of the reference genome (unpublished data) and the unigenes of transcriptome using BLAT (BLAST-like alignment tool). Thirdly, the obtained sequences were explicitly annotated with several public databases, such as Gene Ontology (GO), Clusters of Orthologous Groups of proteins (COG), Kyoto Encyclopedia of Genes and Genomes (KEGG), Pfam, Swiss-Prot, TrEMBL, and NR/NT database. Finally, we performed GO function enrichment, COG function enrichment, and KEGG pathway enrichment analysis with default setting (Kanehisa and Goto2000; Tatusov et al., 2003; Du et al., 2010).

## Results

### Genome Survey Analysis of *P. dabryanus*

Two short paired-end DNA libraries with insert sizes of 270 bp were constructed for the genome survey analysis. A total of 52.58 Gb high-quality reads with 40.57% GC content were produced, which covered approximately 50.23-fold the genome size of *P. dabryanus*, using an Illumina Hiseq 2500 PE150 Platform (Illumina, San Diego, CA). A total of 42,374,856,388 filtered K-mers were obtained from the K-mer analysis, which was conducted among the raw sequencing data based on the frequencies of 21-mers (a specific string with 21 nucleotides). A K-mer curve illustrated that the main peak appeared at the K-mer depth of 38 (**Figure S1**), and the genome size of *P. dabryanus* was estimated as 1.05 Gb with high heterozygosity (0.99%) and approximately 51.90% repeat sequences. The contig N50 size of the highly fragmented genome reads was 670 bp in length; however, the reads covered more than 90% of the transcriptome unigenes from the previous study (Li et al., 2015).

### Phenotypic Analysis of the F1 Population

The seven growth traits and four morphology traits were measured and recorded for 719 F1 individuals. TW, EBW, FL, BL, BW, BH, and HL were 8.30 ± 1.69 g, 6.97 ± 1.42 g, 112.13 ± 10.33 mm, 94.94 ± 7.48 mm, 9.43 ± 1.28 mm, 15.30 ± 1.95 mm, and 15.52 ± 1.94 mm on average (mean ± SD), respectively. The morphological-related traits of ED, CPL, CPH, and IL were 2.72 ± 0.25, 15.30 ± 2.70, 12.36 ± 1.81, and 46.91 ± 4.86 mm on average (mean ± SD), respectively (**Table S1**). As shown in **Figure S2**, most of the growth and morphology traits (except for BW and ED) followed normal distributions (p-value > 0.05) (**Table S1**); however, the sex-related traits deviated from normal distributions (p-value < 0.05) (**Figure S2**).

**Table S2** showed the correlation among each trait. The seven growth traits, four morphology traits, and three sex-related traits were significantly related to each other (p-value < 0.01 for all). The growth traits (TW, EBW, FL, BL, BH, BW, and HL) exhibited a strong correlation with each other (r = 0.857–0.988), and the highest correlation value was observed between TW and EBW. Three morphology traits (CPL, IL, and CPH) showed moderate correlation with each other (r = 0.551–0.878). ED had low correlations with the other traits (r = 0.242–0.511), and the lowest correlation value was observed between ED and MC. WG, MC, and SGD showed moderate correlations with the growth traits and morphology traits (except for ED). A strong correlation was observed between WG and MC, but SGD showed moderate correlations with WG and MC.

In the investigated 719 progenies of F1 family, 307 and 412 individuals were identified as male and females respectively, with a sex ratio of 1:1.34. According to our investigation, almost all the F1 individuals have been sexually mature before sampling. The SGD of *P. dabryanus* is divided into six phases, which denote resting (stages I), recrudescence (stages II), preparatory (stages III), mature (stages IV and V), and depleted phases (stages VI), respectively. The ovarian tissues of 250 females were at stages III, 91 females were at stages II, 52 females were at stages I, and 19 females were at stages IV. The spermary tissues of 176 males were at stages II, 107 males were at stages III, and 24 males were at stages I. The average MCs of female and male individuals were 13.3 ± 5.2 and 3.6 ± 0.7%, respectively.

The results of t-test demonstrated that the seven growth traits data of females were significantly higher than male individuals (p-value < 0.01 for all) (**Table S1**), which indicated that the females of this F1 family grew faster than the males under the same culturing condition.

### SLAF Library Construction and SLAF-Seq Data Analysis

According to the result of pre-experiment, *RsaI* and *HaeIII* (New England Biolabs, USA) were selected for further restriction digestion, and a total of 246,687 SLAF tags ranging from 364 bp to 444 bp were predicted to randomly distribute across the *P. dabryanus* genome. Evaluations of the *Oryza sativa* SLAF library indicated that the cleavage efficiency of *RsaI* and *HaeIII* restriction enzyme was 89.77%, and the paired-end mapped reads accounted for 90.55% of all reads obtained. Consequently, the SLAF library construction of *P. dabryanus* was robust with high-quality.

After SLAF library construction and high-throughput sequencing, about 12.78 billion pair-end clean reads (255.29 Gbp) were generated with a length of 100 bp×2. The number of clean reads in paternal and maternal parents was 33,291,678 and 23,699,226, respectively, as well as 6,103,787 per F1 individual on average. The sequencing depth of SLAF tags in paternal and maternal parents was 54.75-fold and 36.56-fold, respectively, as well as 12.50-fold on average in F1 individuals. The GC contents of paternal parents, maternal parents and F1 individuals were 38.12, 38.19, and 39.06%, respectively. The Q30 ratios of parents and F1 individuals were both over 96%. A total of 1,512,261 SLAF tags were obtained in this study, which satisfied the expected number of SLAF tags. The numbers of SLAF tags in paternal and maternal parents were 475,990 and 349,583, respectively, as well as 387,360 on average in F1 individuals.

### SNP Marker Identification and Genotype Definition

A total of 4,482,083 SNPs were identified by aligning all high-quality reads to the *P. dabryanus* reference genome. Of these 4,482,083 SNPs, 351,338 SNPs (7.84%) were successfully encoded using the genotypes of two parents according to eight types of coding rules. However, aa × bb could not be used for the genetic map construction in this research owing to its lack of segregation in the F1 population. Thus, 272,884 SNP markers with the seven segregation patterns (ab × cd, ab × cc, cc × ab, ef × eg, hk × hk, lm × ll, nn × np) were retained for genetic map construction.

### High-Density Linkage Map Construction and Evaluation

After applying all the filters based on sequencing depth, integrity, chi-square test, and sliding windows, 16,019 SNP markers were used for initial linkage map construction. These identified SNP markers were finally separated into four segregation patterns, of which lm × ll was the major pattern (44.6%), followed by nn × np (42.2%), hk × hk (13.0%), and ef × eg (0.2%) (**Figure S3**). Of these 16,019 SNP markers, 13,906 were homozygous for one parent and heterozygous for the other (7,151 for lm × ll and 6,755 for nn × np), constituting 86.8% of all selected SNP markers. The other two types of markers that could be mapped on both female and male genetic maps only accounted for 13.2% (hk × hk: 2,087 and ef × eg: 26), which could be used as shared markers for the integration of the two parents' maps into one (sex-averaged map).

**Figure 1:**
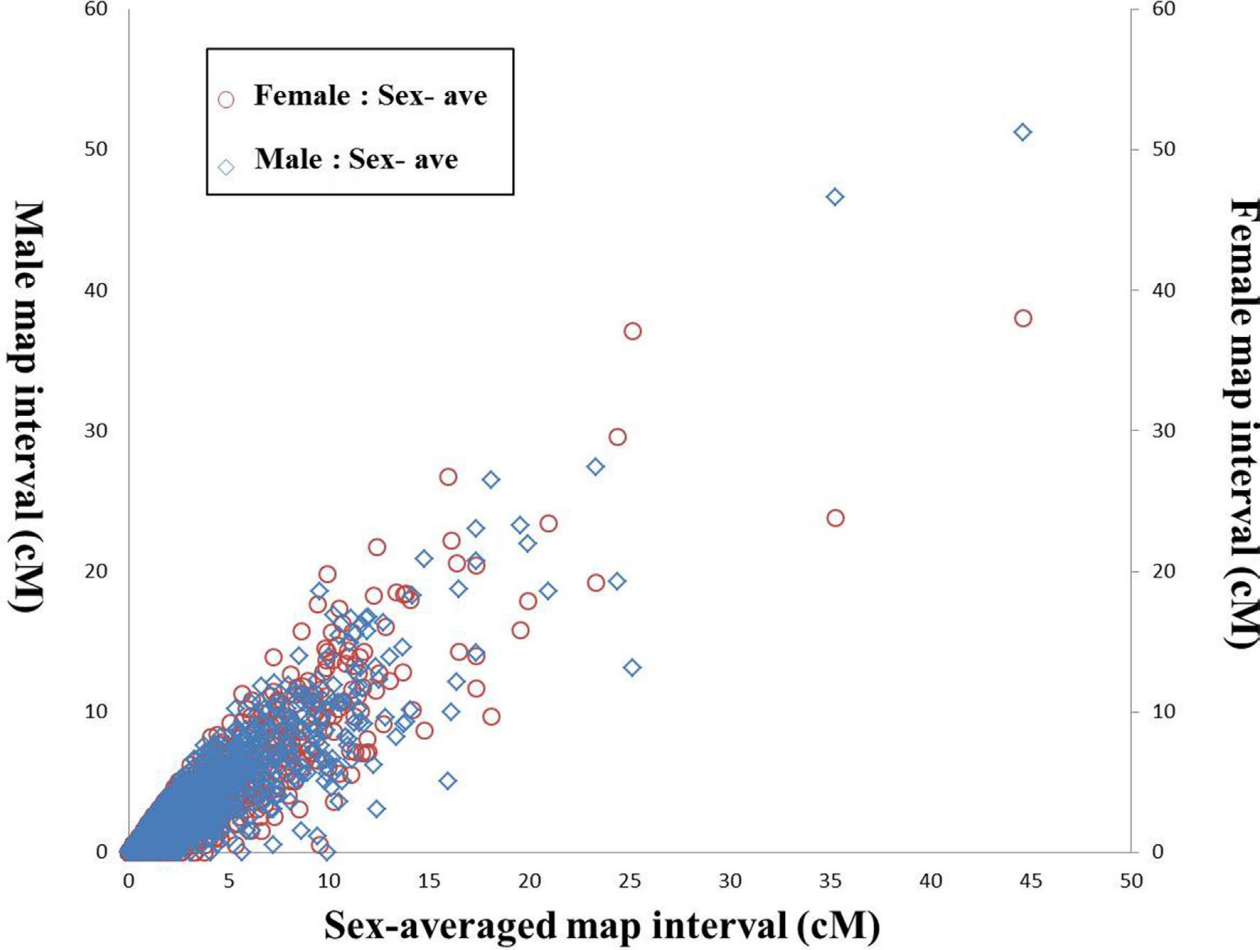
Recombination rates of shared markers between sex-specific maps and sex-averaged map of large-scale loach. The X-axis represents the shared marker interval on the sex-averaged map, the left Y-axis represents the shared marker interval of male map, and right Y-axis represents the shared marker interval of female map. The blue diamonds represent shared marker interval ratio between male map and sex-averaged map (male:sex-ave), and the red circles represent shared marker interval ratio between female map and sex-averaged map (female:sex-ave), respectively.

After filtering the markers with MLOD score < 5, the female-specific and male-specific maps were constructed using HighMap software. As shown in **Table 1**, the female-specific genetic map consisted of 8,693 SNPs was 4,669.31 cM in length with an average distance of 0.54 cM between adjacent markers on 24 LGs. The male-specific genetic map consisted of 9,084 SNPs was 4,530.20 cM in total length with an average distance of 0.50 cM between adjacent markers. The female-to-male LGs length ratio ranged from 0.70 (LG1) to 1.31 (LG6) with an average ratio of 1.03. Recombination rates can be reflected by the genetic length of intervals on LGs. The female-to-male ratio of marker interval ranged from 0.80 (LG1) to 1.37 (LG20) with an average ratio of 1.08. Although the average recombination rate of female-specific map (0.55) was slightly higher than that of male-specific map (0.52), there was no significant difference between both maps (t-test, p-value > 0.05). Further, a total of 1,947 shared SNP markers had been assigned in both female-specific and male-specific linkage maps, respectively. The female-to-male ratio of shared markers intervals on each LGs ranged from 0.71 (LG1) to 1.42 (LG6) with an average ratio of 1.03 (**Table S3**). As shown in **Figure 1**, the average recombination rate of female-specific map (2.58) was slightly higher than that of male-specific map (2.50); however, no significant difference between both maps (t-test, p-value > 0.05).

**Table 1:** Description of the sex-specific maps and the sex-averaged map.

LG ID	Female map					Male map					Sex-averaged map					Female-to-male ratio of marker interval
	Total marker	Total distance (cM)	Marker interval (cM)	Max gap (cM)	Gap < 5 cM(%)	Total marker	Total distance (cM)	Marker interval (cM)	Max gap (cM)	Gap < 5 cM(%)	Total marker	Total distance (cM)	Marker interval (cM)	Max gap (cM)	Gap < 5 cM(%)	
LG1	306	169.74	0.55	3.63	100.00	349	240.84	0.69	10	98.56	600	208.57	0.35	3.73	100.00	0.80
LG2	235	149.95	0.64	4.17	100.00	250	157.66	0.63	4.72	100.00	434	156.61	0.36	2.71	100.00	1.01
LG3	387	191.9	0.5	4.17	100.00	337	177.5	0.53	3.63	100.00	678	191.53	0.28	2.64	100.00	0.94
LG4	405	212.65	0.53	5.12	99.75	299	174.67	0.58	9.52	98.99	607	194.68	0.32	4.35	100.00	0.90
LG5	430	213.24	0.5	15.05	99.30	433	202.18	0.47	6.39	99.31	778	209.48	0.27	6.39	99.87	1.06
LG6	352	207.25	0.59	6.39	99.72	339	158.61	0.47	8.13	99.7	644	188.53	0.29	6.59	99.84	1.26
LG7	369	186.7	0.51	4.72	100.00	400	204.67	0.51	11.16	99	686	196.45	0.29	5.51	99.85	0.99
LG8	347	191.17	0.55	5.21	99.71	408	193.8	0.48	4.17	100.00	681	193.76	0.28	3.11	100.00	1.16
LG9	372	210.43	0.57	7.54	99.46	435	212.17	0.49	4.72	100.00	717	211.3	0.29	2.86	100.00	1.16
LG10	291	173.36	0.6	9.94	98.97	380	187.07	0.49	8.13	99.47	588	182.24	0.31	5.1	99.83	1.21
LG11	446	222.22	0.5	5.27	99.78	495	182.64	0.37	6.96	99.6	806	202.68	0.25	4.42	100.00	1.35
LG12	367	190.65	0.52	5.83	99.73	359	194.11	0.54	8.72	99.44	651	198.78	0.31	5.27	99.85	0.96
LG13	328	189.81	0.58	5.27	99.69	266	171.26	0.64	9.15	98.49	529	181.61	0.34	5.07	99.81	0.90
LG14	251	179.93	0.72	5.83	98.80	266	159.91	0.6	3.37	100.00	458	172.8	0.38	4.75	100.00	1.19
LG15	535	235.17	0.44	5.83	99.63	552	189.75	0.34	4.72	100.00	964	215.32	0.22	3.24	100.00	1.28
LG16	369	197.69	0.54	5.27	99.73	369	207.1	0.56	6.96	99.46	638	205.45	0.32	3.61	100.00	0.95
LG17	393	218.11	0.55	5.27	99.74	331	167.22	0.51	5.54	99.7	627	193.18	0.31	2.75	100.00	1.10
LG18	328	172.02	0.52	6.96	99.69	358	199.06	0.56	9.92	98.88	600	188.34	0.31	4.96	100.00	0.94
LG19	279	166.77	0.6	7.54	99.64	316	171.66	0.54	4.72	100.00	529	170.25	0.32	3.33	100.00	1.10
LG20	261	189.12	0.72	5.27	99.62	345	182.01	0.53	3.09	100.00	531	187.83	0.35	2.84	100.00	1.37
LG21	513	214.12	0.42	8.13	99.8	708	237.8	0.34	11.29	99.86	1,082	226.97	0.21	5.64	99.91	1.24
LG22	278	179.13	0.64	8.13	99.28	309	190.97	0.62	3.09	100.00	547	187.84	0.34	2.54	100.00	1.04
LG23	435	205.24	0.47	9.92	99.54	410	188.74	0.46	6.39	99.51	761	199.77	0.26	6.39	99.74	1.02
LG24	416	202.94	0.49	8.72	98.80	370	178.8	0.48	11.16	99.19	694	193.67	0.28	4.54	100.00	1.01
Total	8,693	4,669.31	/	/	/	9,084	4,530.20	/	/	/	15,830	4,657.64	/	/	/	/
Average	362	194.55	0.55	6.63	99.6	379	188.76	0.52	6.9	99.55	660	194.07	0.3	4.26	99.95	1.08

**Figure 2:**
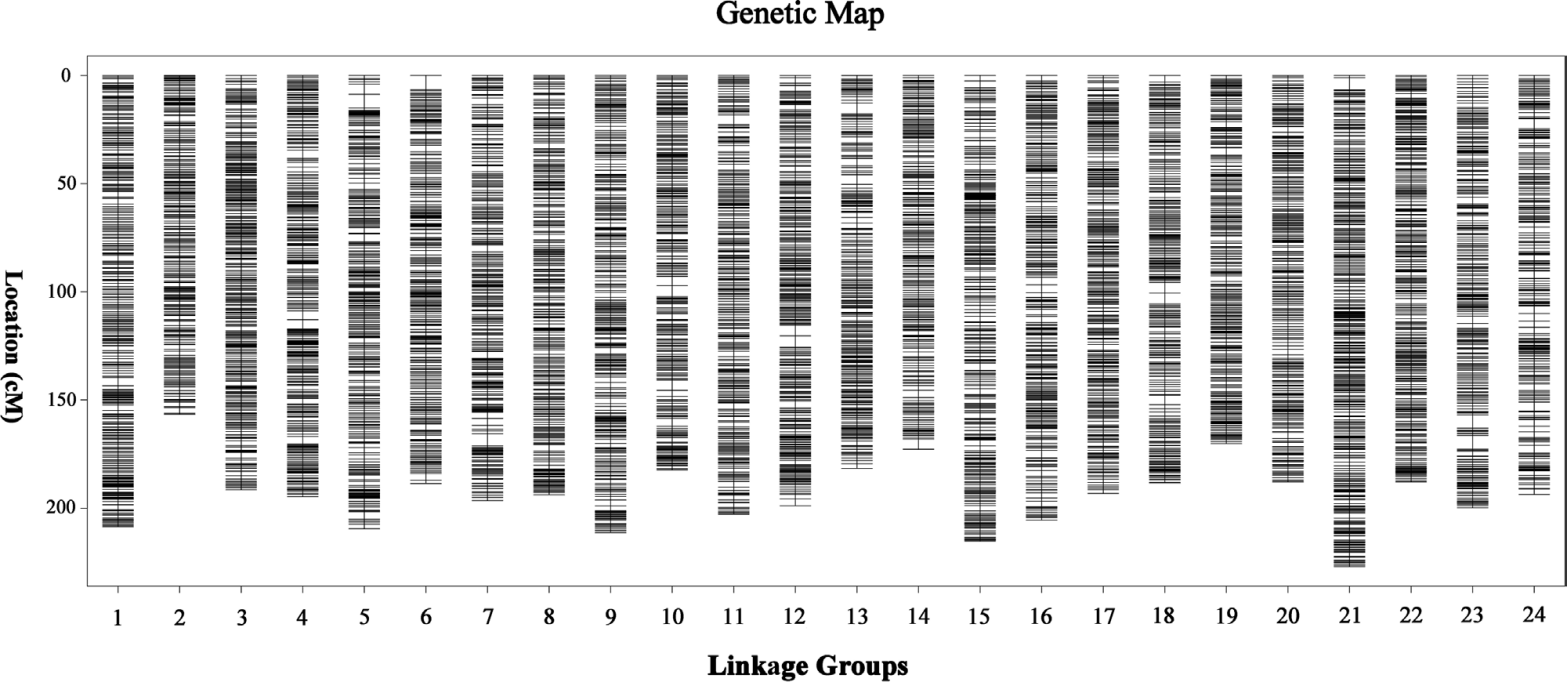
High-density sex-averaged genetic linkage map of large-scale loach. The X-axis represents the linkage group of large-scale loach. The Y-axis represents the genetic position.

Finally, a sex-averaged map was generated by integrating both sex-specific genetic maps, and a total of 15,830 markers were assigned onto 24 LGs (**Figure 2**). As shown in **Table 1**, the sex-averaged genetic map was 4,657.64 cM in length with an average distance of 0.30 cM between adjacent markers. The total genetic distances of the 24 LGs ranged from 156.61 cM (LG2) to 226.97 cM (LG21), with the average markers distance spanned from 0.21 cM (LG21) to 0.38 cM (LG14). Among the 15,830 SNP markers, 1,082 were assigned on LG21, which was the largest one in the 24 LGs. The total length was 226.97 cM with an average distance of only 0.21 cM between adjacent markers in this LG. The smallest LG2 contained 434 SNP markers, which was 156.61 cM in length with an average distance of 0.36 cM between adjacent markers. The maximum gap of the 24 LGs ranged from 2.54 cM (LG22) to 6.59 cM (LG6), and the gap less than and/or equal to 5.0 cM (gap < = 5) ranged from 99.74 to 100.00% with an average value of 99.95%, which indicated the good quality of marker assignment in this study. The sequencing depth of mapped SNP markers in paternal and maternal parents was 165.29-fold and 134.03-fold, respectively, as well as 38.13-fold on average in F1 individuals.

The integrity of the mapped markers (the proportion of markers with definite genotype to the total markers) in 200 F1 individuals was 99.96% on average, which indicated a relatively high quality of the genetic map (**Figure S4**).

As shown in **Table 2**, 181 markers that showed significant segregation distortion (χ2, p < 0.05) were finally assigned onto the map, which were distributed in clusters on LG4, LG5, LG8, LG9, LG10, LG11, LG13, LG15, LG16, LG17, LG20, LG21, LG22, LG23, and LG24. The highest frequency of segregation distortion markers was observed in LG13 (5.48%); however, the largest number of segregation distortion markers were distributed on LG21. A total of 23 segregation distortion regions (SDRs) were detected on LG5, LG10, LG11, LG13, LG15, LG17, LG20, LG21, and LG22 of the map, and the largest number of SDRs was distributed on LG21.

**Table 2:** Description of the segregation distortion markers of the genetic map.

LGs	Total marker		Segregation distortion marker		*χ* ^2^	*P*	Segregation distortion marker frequency (%)	SDR
	Number	Percentage (%)	Number	Percentage (%)				
1	600	3.79	0	0.00	/	/	0.00	
2	434	2.74	0	0.00	/	/	0.00	
3	678	4.28	0	0.00	/	/	0.00	
4	607	3.83	5	2.76	8.346	0.007	0.82	0
5	778	4.91	26	14.36	8.654	0.005	3.34	4
6	644	4.07	0	0.00	/	/	0.00	
7	686	4.33	0	0.00	/	/	0.00	
8	681	4.30	3	1.66	36.003	0.000	0.44	0
9	717	4.53	2	1.10	9.725	0.008	0.28	0
10	588	3.71	11	6.08	8.445	0.005	1.87	2
11	806	5.09	11	6.08	77.686	0.000	1.36	2
12	651	4.11	0	0.00	/	/	0.00	
13	529	3.34	29	16.02	8.074	0.005	5.48	3
14	458	2.89	0	0.00	/	/	0.00	
15	964	6.09	4	2.21	10.320	0.006	0.41	1
16	638	4.03	14	7.73	7.845	0.008	2.19	0
17	627	3.96	16	8.84	7.772	0.008	2.55	1
18	600	3.79	0	0.00	/	/	0.00	
19	529	3.34	0	0.00	/	/	0.00	
20	531	3.35	9	4.97	7.097	0.008	1.69	1
21	1,082	6.84	44	24.31	8.011	0.006	4.07	8
22	547	3.46	5	2.76	6.924	0.009	0.91	1
23	761	4.81	1	0.55	11.320	0.003	0.13	0
24	694	4.38	1	0.55	14.670	0.001	0.144	0

Haplotype map and heat map were employed to evaluate the quality of the genetic map. Since haplotype map reflects the double crossover of the population, it can visually display the recombination events of each individual and suggest the genotyping errors. Haplotype maps were generated for the F1 population and the two parents using 15,830 mapped SNP markers (**Figure S5**) (West et al., 2006). The majority of the recombination blocks were clearly defined, and there was no deletion of markers detected on any LG of the genetic map, which suggested no significant effect on genetic map quality. The heat map was utilized to examine potential ordering errors of markers since it can illustrate the relationship of recombination between markers from one single LG. As shown in **Figure S6**, the linkage relationship was extremely strong between adjacent markers, and it changed from strong to weak gradually with the increase of genetic distance. This result showed that the SNP markers in most LGs were well ordered.

Consequently, the genetic map was established with high quality and reliability for future research on the genetics and genomics of *P. dabryanus*.

### Genome Scaffold Anchoring and Comparative Genome Mapping

The high-density linkage map provided the framework of chromosomes with adequate anchor points for the reference genome sequences (unpublished) assembly and map integration of *P. dabryanus*. Of all the 15,830 mapped markers, a total of 15,313 markers (96.7%) were successfully aligned to 774 scaffolds representing 960.27 Mb of the genome regions, which indicated the consistency among the SNP markers, genetic map, and scaffolds from the genome. The length of scaffold N50 reached 3.0 Mb, which indicated a greatly high quality of genome assembly. Further, the characteristics of oriented scaffolds and unanchored scaffolds were displayed in **Table S4**.

**Figure 3:**
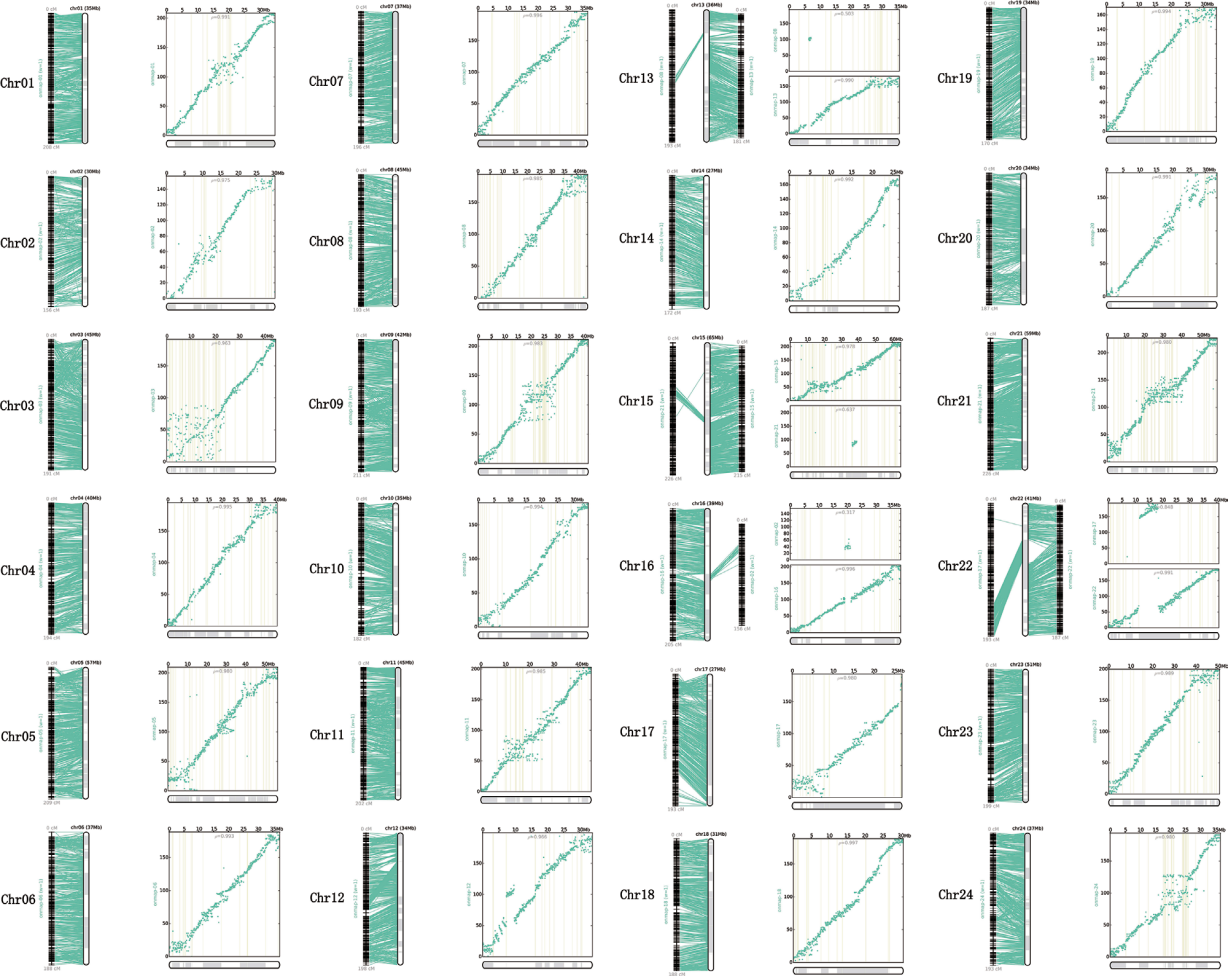
Relationship between genetic map and reference genome of large-scale loach. Each sub-graph represents an assembled chromosome and the corresponding LGs. For each sub-graph, the left part represents the linear arrangement relationship between LGs and assembled chromosomes; the right part represents the genetic and physical positions of markers, physical distance is on the X-axis, and genetic distance is on the Y-axis.

All the genetic markers across the 24 LGs were mapped to the *P. dabryanus* reference genome for evaluating the collinearity between the genetic map and the reference genome. **Figure 3** illustrated the anchors between the genetic markers and the assembled chromosomes. A one-to-one correspondence was observed among most of the LGs and chromosomes, except for chromosome 13 (Chr13), chromosome 15(Chr15), chromosome 16 (Chr16), and chromosome 22 (Chr22), which were all anchored onto both LGs. Each chromosome covered 40.13 Mb of the genome region on average, with a range from 27 Mb (Chr14 and Chr17) to 65 Mb (Chr15). **Figure 3** showed the physical position and the genetic position of the genetic markers using the scatter plots. Overall, the distribution of SNP markers was relatively uniform across the 24 LGs; however, some clear dense-regions and desert-regions of markers were detected in the terminal and middle of LGs. The relationships between the genetic map and draft genome were generally linear for 24 chromosomes with the exception of Chr13, Chr15, Chr16, and Chr22. The Spearman correlation coefficient of each LG was displayed in **Table S5**. The Spearman correlation coefficient between the physical and genetic positions of all LGs is 0.927 on average, which indicated that the genetic map showed high levels of collinearity with the reference genome. These results suggested that the genetic markers of this map were able to accurately and adequately cover the whole genome of *P. dabryanus*.

**Figure 4:**
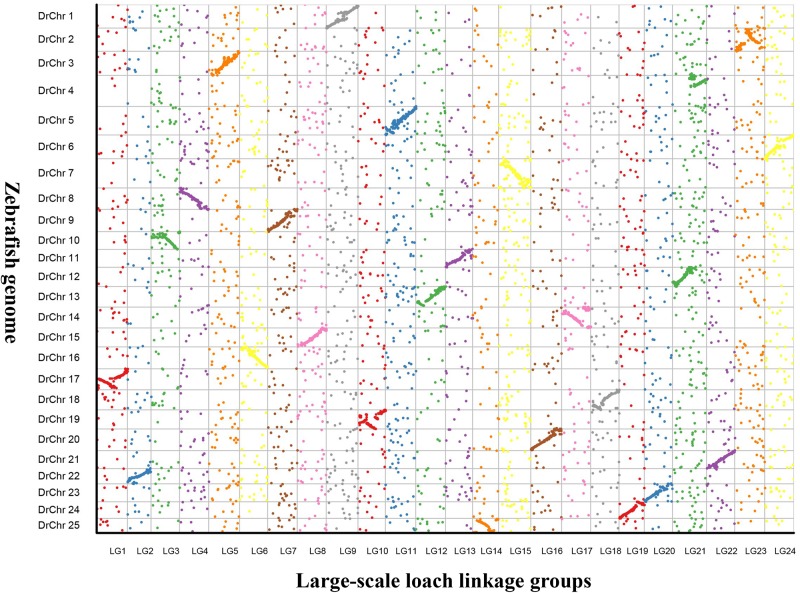
Genomic comparisons between large-scale loach and zebrafish. The colored dots represent the orthologues identified in large-scale loach and zebrafish. X-axis represents the genetic position of orthologues on each LG of large-scale loach, and Y-axis represents the position of orthologues on each chromosome of zebrafish.

In addition, 24 putative chromosomes were constructed based on the LGs of the genetic map in *P. dabryanus*. The comparative genome mapping was performed between large-scale loach and zebrafish as a closely related model species, and the scatter plot was drawn based on the orthologous pairs between two species. A total of 7,615 1:1 best orthologues between large-scale loach and zebrafish were identified on zebrafish chromosomes. The number of orthologues detected on the chromosomes of zebrafish ranged from 204 (Chr22) to 438 (Chr7) with an average of 304.6. As shown in **Figure 4**, all of the 24 LGs were in relatively conserved synteny with the 25 DrChr (DrChr: *Danio rerio* chromosome). Of these chromosomes, LG1, LG10, and LG23 exhibited relatively low degrees of synteny with DrChr17, DrChr19, and DrChr2, which displayed the marker order inconsistency. Moreover, LG21 was found to be syntenic with both DrChr4 and DrChr12 due to the inconsistency of chromosome number.

### QTL Mapping of Growth, Morphology, and Sex-Related Traits

**Figure 5:**
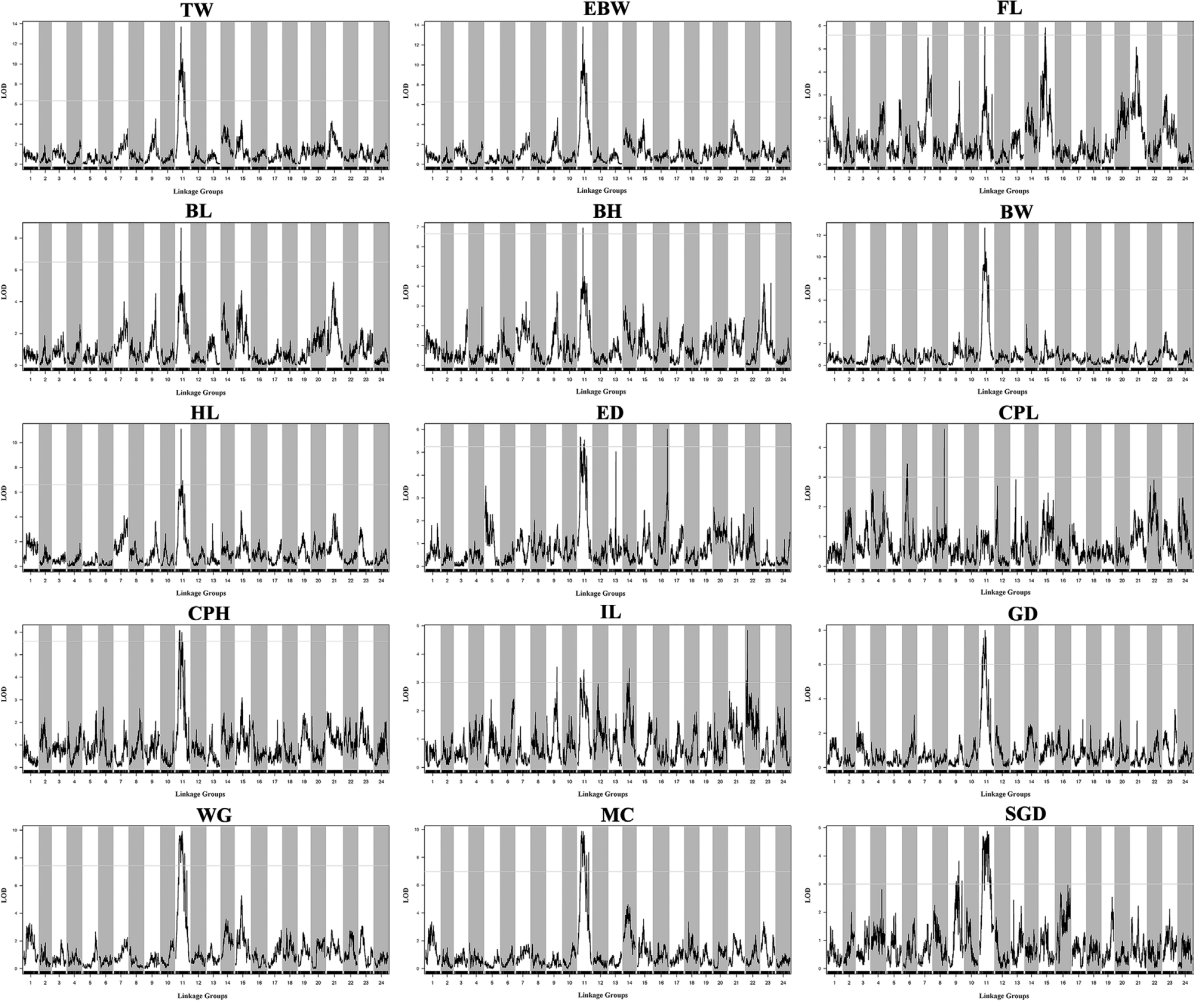
QTL mapping for the traits of growth, morphology, and sex in large-scale loach. Each sub-graph represents the QTL distribution of a target trait. For each sub-graph, the X-axis represents the linkage groups, and the Y-axis represents the corresponding LOD values. Significant QTL regions were identified for each trait.

**Table 3** provided all the detailed information of QTLs, such as LOD threshold, QTL interval, PVE value, maximum LOD, the position corresponding to the peak value of LOD, and number of the markers within the QTL region. The seven most significant QTL regions for the seven growth-related traits (TW, EBW, FL, BL, BH, BW, and HL) were identified on LG11 with LOD thresholds ranged from 5.59 (qFL) to 6.97 (qBW), as well as one QTL related to FL was distributed on LG15 of *P. dabryanus* (**Figure 5**). Similarly, the four most significant QTL regions associated with sex (GD, WG, MC, and SGD) were detected on LG11 with LOD thresholds ranged from 3.00 (qSGD) to 7.44 (qWG), and the other QTL for SGD was distributed on LG9. The nine most significant QTLs for four morphological-related traits were detected on LG6, LG8, LG9, LG11, LG14, LG16, and LG22 with LOD thresholds ranged from 3.00 (qCPL and qIL) to 5.59 (qCPH). Interestingly, three of these nine QTLs were also concentrated on LG11. In conclusion, the most significant QTLs for almost all the traits related to growth, morphology (except for CPL), and sex were located on the same LG LG11 (**Figure 5**), and these QTLs explained a high percentage of phenotypic variance: from 10.4 to 27.4% (**Table 3**).

**Table 3:** Description of the QTLs for the traits of growth, morphology and sex.

Trait	QTL ID	LOD threshold	LG ID	QTL interval (cM)	Peak position (cM)	Max LOD	Max PVE (%)		Marker number
							Minimum	Maximum	
TW	qTW-1	6.33	11	39.57–136.19	77.68	13.693	13.8	27.0	396
FL	qFL-1	5.59	11	77.36–77.68	77.36	5.94	12.7	12.8	3
	qFL-2	5.59	15	82.70–90.39	89.60	5.905	12.2	12.7	15
BL	qBL-1	6.50	11	72.26–77.68	77.36	8.623	15.3	18.0	8
BH	qBH-1	6.64	11	77.36–77.68	77.68	6.935	14.5	14.8	3
BW	qBW-1	6.97	11	47.14–134.23	76.85	12.656	15.0	25.3	320
HL	qHL-1	6.60	11	77.36–101.03	77.68	11.088	14.1	22.5	15
EBW	qEBW-1	6.27	11	39.57–136.19	77.68	13.813	13.6	27.2	396
CPL	qCPL-1	3.00	6	54.02–65.50	58.12	3.454	16.8	17.6	32
	qCPL-2	3.00	8	156.29–156.60	156.29	4.617	12.2	13.1	4
CPH	qCPH-1	5.59	11	52.55–89.37	58.19	6.084	12.5	13.1	37
ED	qED-1	5.25	11	39.57–98.86	39.57	5.672	11.4	12.2	50
	qED-2	5.25	16	191.29–191.68	191.29	6.013	12.1	12.9	3
IL	qIL-1	3.00	9	147.81–148.31	148.31	3.535	7.4	7.8	9
	qIL-2	3.00	11	39.57–93.71	39.57	3.445	16.7	17.6	57
	qIL-3	3.00	14	66.01–84.50	84.50	3.487	9.7	10.7	5
	qIL-4	3.00	22	1.55–22.28	22.02	4.84	6.8	10.5	10
GD	qGD-1	6.01	11	38.14–98.56	80.22	7.992	18.1	21.8	249
WG	qWG-1	7.44	11	48.15–129.99	93.71	9.916	16.0	20.4	302
MC	qMC-1	6.97	11	38.14–160.29	60.04	9.871	14.9	20.3	307
SGD	qSGD-1	3.00	9	104.81–191.84	147.81	3.812	8.7	12.6	27
	qSGD-2	3.00	11	39.57–165.03	111.95	4.871	8.7	10.4	500

### Identification of Potentially Key Markers and Genes Linked to Growth and Sex

**Figure 6:**
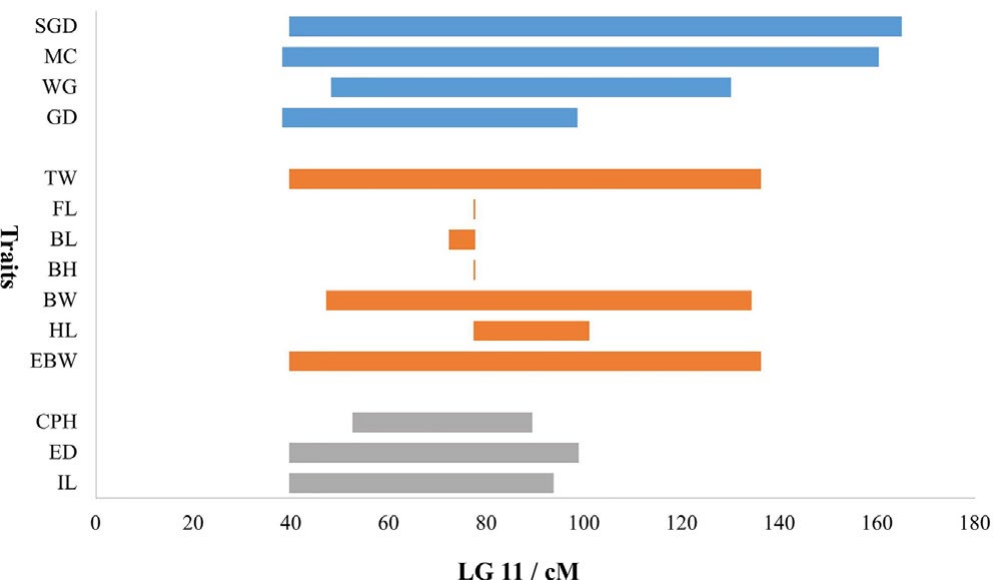
Distribution of overlapped QTLs for traits on LG11. The X-axis represents the genetic location of QTL regions, and the Y-axis represents the target traits. The blue bars represent the four traits of sex, the orange bars represent the seven traits of growth, and gray bars represent the three traits of morphology.

Because most of the significant QTLs for almost all the traits were concentrated on LG11, a bar chart was used to show the distribution of overlapped QTLs in LG11 (**Figure 6**). The QTL regions for all the growth-related traits overlapped on LG11 from 77.36 to 77.68 cM with three shared SNP markers (Marker172604, Marker114865, and Marker159479). Additionally, the QTL regions for the morphological-related traits (except for CPL) overlapped on LG11 from 52.55 to 89.37 cM with 37 shared SNP markers, and the QTL regions for all the sex-related traits overlapped on LG11 from 48.15 to 98.56 cM with 239 shared SNP markers.

Besides, TW, EBW, BH, and HL reached max LOD at the same genetic position of 77.68 cM and correspondingly had the highest PVE value of 27.4, 27.2, 14.8, and 22.5%, respectively. The QTLs for FL and BL with Max LOD were both located at 77.36 cM on LG11, and correspondingly had the highest PVE value of 12.8 and 18.0% (**Table 3**). We identified three potentially key markers (Marker159479, Marker172604, and Marker114865) near these two locations. The QTLs related to gender with the highest LOD score (max LOD = 7.992) and highest PVE value (max PVE = 21.8%) were located at 80.22 cM of LG11 (**Table 3**), and we identified four potentially key markers (Marker51869, Marker329989, Marker51904, and Marker282599) near this location.

In accordance with the above results, Marker159479, Marker172604, and Marker114865 are most likely to be linked to growth, as well as Marker51869, Marker329989, Marker51904, and Marker282599 are closely related with sex determination. These markers were further mapped onto the scaffolds of the reference genome (unpublished data), and the obtained sequences were annotated with the public databases. Finally, *Cyp19b* (cytochrome P450 aromatase B) and *Gh* (growth hormone) genes were detected near Marker114865. *Leo1* (RNA polymerase-associated protein LEO1-like) and *Adgrv1* (adhesion G-protein coupled receptor V1) genes were identified near Marker172604 (**Table S6**). These genes may be closely linked to the growth traits of *P. dabryanus*. *Cyp19b*, *Vtg1* (vitellogenin 1), and *Lgals1* (lectin galactoside-binding soluble 1) genes were identified near Marker51869. *Dmrt1* (doublesex and mab-3 related transcription factor 1) and *Irs1* (insulin receptor substrate 1-B-like) genes were identified near Marker282599. *Acta2* (actin aortic smooth muscle), *Wap65* (warm-temperature-acclimation-related 65 kDa protein), and *Dnd* (dead-end) genes were detected near Marker51904 (**Table S6**). These genes may be closely linked to sex determination of *P. dabryanus*.

Based on the sex-related QTLs results, reference genome of *P. dabryanus*, and public databases, we identified 394, 430, 467, and 893 homologous genes in the significant QTL regions of GD, WG, MC, and SGD, respectively (**Table S7**). To further investigate the function of the candidate genes associated with sex, several function enrichment analyses were performed across genome-wide (**Figure S7**). As the result of GD shown, the three most enriched COG terms were general function prediction only, signal transduction mechanisms, and carbohydrate transport and metabolism, respectively. The three most enriched GO terms were "cell part" in cellular component group, "binding" in molecular function group and "cellular process" in biological process group, respectively. The three most enriched KEGG pathways were "regulation of actin cytoskeleton" in cellular processes, "neuroactive ligand-receptor interaction" in environmental information processing, and "insulin signaling pathway" in organismal system, respectively. **Figure S7** showed all the functional enrichment results, and the enrichment characteristics of four sex-related traits exhibited high similarity.

## Discussion

*P. dabryanus* is a freshwater cultured species with the growing economic value; therefore, further research is needed to clarify the characteristics of genomic and genetic, although the physiology, development, immunology, and genetic breeding of this species have been intensively studied.

There is no reference genome and high-resolution linkage map available for large-scale loach so far. In this study, a high-density and high-resolution genetic linkage map was constructed for the first time, and we provided the first fine-scale QTL mapping for growth, morphology, and sex related traits in *P. dabryanus*. Several potentially key markers and genes closely linked to growth and sex were identified. Besides, the first genome survey analysis, which represented the first step toward fully decoding the genome, was carried out in *P. dabryanus*. The scaffolds of the reference genome were successfully anchored using this high-density genetic map in *P. dabryanus*.

### High Density Genetic Map for *P. dabryanus*

The segregated population types directly affect the mapping efficiency of linkage maps, and different mapping families have both advantages and disadvantages. The commonly used mapping families for linkage genetic map construction mainly include permanent population [such as recombinant inbred lines (RIL), haploid (HAP) and double haploid (DH) families] and tentative population [such as F1, F2, and backcross (BC) families]. However, it is a significant challenge to construct RIL, HAP, and DH families in most teleost fish, and the constructions of F2 and BC families usually take a relatively long time (Peng et al., 2016). Due to the relatively long reproductive cycle, complex genome, and high genetic polymorphism of aquaculture species, the F1 mapping family with double pseudo-testcross strategy is used to construct genetic linkage maps in most of the aquaculture species. In this study, the parents of F1 population were respectively derived from the wild populations of *P. dabryanus* in Dongting Lake and Hongze Lake, which show the relatively high level of genetic polymorphism and genetic differentiation (Li et al., 2014). Besides, the F1 mapping population from a cross between fast-growing and slow-growing varieties was employed for the genetic map construction, which is beneficial for the identification of the polymorphism markers (Ji et al., 2017). Hence, the F1 mapping family with double pseudo-testcross strategy was particularly suitable for the construction of genetic linkage maps in this study.

In the present study, the SLAF-seq strategy, which balances genotyping accuracy and sequencing cost, was applied for the large-scale genotyping and high-density genetic map construction in *P. dabryanus*. We subsequently identified 8,693 and 9,084 SNP markers for the construction of female-specific and male-specific maps, respectively, which can be used as testcross loci for the backcross pattern in a 1:1 ratio. The abundant testcross loci are enabled to significantly enhance the genetic map density and the efficiency and accuracy of QTL mapping. The previous study indicated that the error rate of genotyping declined significantly with the increase of read depth, and the error rate was almost negligible when the read depth reached 12 (Sun et al., 2013). In the present research, the sequencing depth of mapped SNPs in paternal and maternal parents was 165.29-fold and 134.03-fold, respectively, as well as 38.13-fold on average in F1 individuals, which ensured a high accuracy for marker genotyping in this research. Additionally, no significant difference was observed in the average recombination rates between both the specific-sex maps (t-test, p-value > 0.05) (**Figure 1**). These results suggested a high similarity and collinearity of both sex-specific maps for the large-scale loach, which are important for the construction of the sex-averaged map with high accuracy and quality.

Segregation distortion, which is defined as a deviation of the observed genotype frequency from a typical Mendelian ratio, is a common phenomenon in plants and animals (Taylor and Ingvarsson, 2003). Generally, markers with segregation distortion would affect the accuracy of genetic map construction (Liu et al., 2016). Several studies, however, indicated that the presence of segregation distortion markers made little effect on the applications of linkage maps for further analysis such as QTL mapping (Zhang et al., 2010). In addition, the distorted markers for linkage map construction could improve both the genome coverage of the genetic map and the identification of linked QTLs, and filtering out all the distorted markers may lead to a massive loss of genetic information and the decrease of genome coverage (Niu et al., 2017). To increase the genome coverage of the final genetic map, 181 markers that showed significant segregation distortion (χ2, p < 0.05) were retained and assigned to 15 LGs in this study. Most of the distorted markers clustered into 23 SDRs. The highest frequency of segregation distortion markers was observed in LG13 (5.48%); however, the largest number of segregation distortion markers were distributed on LG21. The potential mechanism and function of segregation distortion remain unclear, although it is considered as a potentially powerful evolutionary force (Taylor and Ingvarsson, 2003) and could be associated with preferential selection or gametic/zygotic selection (Weber, 1990; Faris et al., 1998; Wang et al., 2005).

Moreover, the quality of genetic map was evaluated in different aspects with the haplotype map, heat map, gap < 5 cM (%), and mapped marker integrity, *etc*. Compared with most of the previous maps constructed for the aquaculture species, this study provided a high-quality genetic map with higher density, resolution, and accuracy. This high-density SNP-based linkage map serves as an important reference for fine mapping of important economic traits, comparative genomics, genome assembly, and the MAS breeding in large-scale loach.

### Patterns of Sex-Specific Recombination

The sex-specific difference in recombination rates has been one of the focuses of genetics, which was highlighted in a number of research on the genetics of vertebrate and invertebrates (Singer et al., 2002; Lv et al., 2017). The recombination rate of heterogametic sex (such as XY or ZW) was generally more restricted than homogametic sex (Qiu et al., 2017). In the XX/XY sex-determination system, thus, females should have higher recombination rates than males in theory. It was proved in rainbow trout (*Oncorhynchus mykiss*), Atlantic salmon (*Salmo salar*), and common carp (*Cyprinus carpio*) with a significantly high female to male recombination ratios of 8.26, 3.25, and 4.20, respectively (Sakamoto et al., 2000; Moen et al., 2004; Zhang et al., 2013a). However, it's not always the case in the species of XX/XY sex-determination system; the conflicting results were found even in the same species. For instance, other studies indicated that the female to male recombination ratios were observed to be 0.77 and 0.93 in Atlantic salmon and common carp, respectively (Gonen et al., 2014, Peng et al., 2016). Similar counterexamples have also been observed in the species of ZZ/ZW sex-determination system, which should show a relatively low female to male recombination ratios in theory. However, the female to male recombination ratios was observed to be 0.98 and 1.3 in half-smooth tongue sole (*Cynoglossus semilaevis*) and turbot (*Scophthalmus maximus* L.), respectively (Jiang et al., 2013; Ruan et al., 2010).

The genetic mechanism of sex determination in large-scale loach is still controversial. The early karyotypic analysis and sex ratios result suggested a putative ZZ/ZW sex-determination system in this species (Chang and Yu, 1997; You et al., 2008). In a separate study, however, the sex ratio of the progenies in the gynogenetic families was significantly deviated from 1:1 expectation with a female bias of 93.62%, which conflicted with the typical ZZ/ZW sex-determination system (Liu et al., 2016). In this study, the female to male recombination ratios of the sex-specific maps were 1.08 on average, as well as the sex recombination ratio of shared markers was 1.03, which suggested that our data cannot be evidence for supporting a XX/XY or a ZZ/ZW system in *P. dabryanus*. Further studies should be conducted to better understand the mechanism of sex determination in *P. dabryanus* and the relationship between sex recombination ratios and sex determination.

### Chromosome Framework for Genome Anchoring and Comparative Genome Mapping

In the present study, the genome scaffolds were anchored to the high-density linkage map, spanning 960.27 Mb of *P. dabryanus* reference genome. Compared to the previous assembly version of the reference genome, the number of scaffolds significantly decreased after genome anchoring. The scaffold N50 of the reference genome reached 3.0 Mb, and each chromosome covered 40.13 Mb of the genome region on average, which demonstrated that the genetic map can be used for improving the quality of assembly and significantly enhancing the genome integrity.

In addition, the high-density genetic map could provide chromosome framework for genome assembly validation (Hedgecock et al., 2015; Peng et al., 2016). In our study, a certain proportion of the scaffolds disagreed with marker position on the genetic linkage map were identified from Chr13, Chr15, Chr16, and Chr22 of the genome using the collinearity analysis between the genetic map and the draft genome. According to the previous study, this result may be caused by assembly errors, which can be corrected with the new sequencing technologies or the modified assembly algorithms (Peng et al., 2016).

Furthermore, the high-density linkage map provides new chromosome framework for comparative genomic studies with model animals for the species without a reference genome. For instance, the comparative genome mapping has been conducted among common carp, zebrafish, and medaka in the early research, which showed nearly perfect chromosome-scale syntenies between common carp and zebrafish but no significant collinearity between common carp and medaka (Peng et al., 2016). Similar synteny analysis has been performed between large-scale loach and zebrafish in the present study. Although a degree of synteny was observed among all the LGs of *P. dabryanus* and DrChrs of zebrafish, LG1, LG10, and LG23 showed relatively low degrees of synteny with DrChr17, DrChr19, and DrChr2, respectively. These results suggested that a certain degree of homology is present between large-scale loach and zebrafish; however, the chromosome rearrangements could have occurred post the divergence of large-scale loach and cyprinids.

### QTLs and Candidate Genes for the Growth and Sex-Related Traits

Growth and sex are the most important traits for aquaculture species (Zhang et al., 2019). The significant sex dimorphic growth patterns were found in some teleost fish, and the production of monosex populations provides higher economic values (Mei and Gui, 2015). In this research, the females exhibited a faster growth rate than males under the same culturing condition at one and a half years after hatching. This result is in line with the previous study, which indicated that reproductive development would contribute more to the growth of females in *P. dabryanus* (Wang and Li, 2005b).

QTL mapping and association studies on growth and sex-related traits have been intensively performed on many aquaculture species (Yue, 2014). It is noteworthy in this study that most of the significant QTL regions for both growth and morphology traits were concentrated on LG11, which suggested that the main genes contributed to growth and morphology may be distributed on the same chromosome. This result is not consistent with the earlier findings in many aquaculture species (e.g., pikeperch, *Portunus trituberculatus*, Snapper, *etc.*) that the growth-related traits may be contributed to genes from different chromosomes (Lv et al., 2017; Guo et al., 2018; Ashton et al., 2019). Similarly, most of the significant QTL regions for the sex-related traits were also observed in LG11, which suggested that LG11 plays critical roles in the regulation of growth, gonadal development, and sex differentiation in *P. dabryanus*. All the traits of growth and sex had some overlapping QTL regions on LG11, and these genomic regions could be recognized as the candidate functional regions for both growth and sex regulation. Additionally, the sex-related traits showed a significant correlation with growth traits, and the growth traits exhibited significant gender dimorphism. Therefore, we suggested that some of the candidate markers and genes in overlapping QTL regions may have pleiotropic effects, which means they could be associated with two or more traits simultaneously. Similar results in small abalone and Asian sea bass have been confirmed (Xia et al., 2014; Ren et al., 2016).

Since the importance of LG11, we identified several potentially key markers linked to growth and sex determination on this LG, which will be valuable as a utility for further evaluation by MAS. Furthermore, a total of 12 candidate genes closely linked to growth or sex were detected with explicit annotated information near these markers. Previous studies have revealed the functions of some of these genes in other species. GH is a polypeptide implicated in regulating the development and somatic growth in teleost fish (Johnston et al., 2011). Leo1 is a component of the polymerase-associated factor 1 (PAF1) complex, which participates in the regulation of development and maintenance of embryonic stem cell pluripotency (Ding et al., 2009). ADGRV1 has an essential role in the regulation of bone metabolism (Knapp and Wolfrum, 2016). It is reasonable to suppose that *Gh*, *Leo1*, and *Adgrv1* play important roles in the growth and development of *P. dabryanus*. The roles of *Cyp19b*, *Dmrt1*, and *Vtg1* in sex differentiation of aquaculture species have been revealed in the earlier studies (Du et al., 2006; Xu et al., 2008; Esterhuyse et al., 2009). The *Dnd* gene is crucial for primordial germ cell (PGCs) migration and development in teleosts (Wargelius et al., 2016; Hong et al., 2016), and a previous study on turbot suggested that *Dnd* may play different roles in gonadal development of both sexes and can be used as a germ cell marker (Lin et al., 2013). Thus, we speculated that *Cyp19b*, *Dmrt1*, *Vtg1*, and *Dnd* are involved in the process of sex determination and differentiation of *P. dabryanus*. The functional annotations of most candidate genes are consistent with QTL mapping, which indicated that the results of QTL on LG11 were relatively accurate. Interestingly, *Lgals1* and *Irs1* were previously reported to be involved in growth (Mehrabian et al., 1993; Dong et al., 2006), although the two genes were detected near the sex-linked markers in this study. Besides, the *Cyp19b* was simultaneously identified near both candidate markers associated with growth and sex. These results could support the suggestion that the candidate genes in overlapping QTL regions may have pleiotropic effects, and further functional verifications of these genes are essential.

## Conclusion

In this study, the first high-density genetic map of *P. dabryanus* was constructed with an F1 population derived from a cross between fast-growing and slow-growing wild populations in different areas. A total of 12.78 billion pair-end clean reads (255.29 Gbp) with 39.06% GC content were generated using SLAF-seq. The *P. dabryanus* genetic map contained 24 LGs with 15,830 SNP markers, which spanned 4,657.64 cM with an average inter-marker distance of 0.30 cM.

A series of approaches (e.g., the ratio of gap < = 5, haplotype map, heat maps, and collinearity analysis, *etc.*) were conducted to evaluate the quality of genetic map, which indicated that our map was constructed with high quality and high accuracy and reliable for future research on the genetics and genomics of *P. dabryanus*. The female to male recombination ratios of the sex-specific maps were 1.08 on average, suggesting that our data cannot be evidence for supporting a XX/XY or a ZZ/ZW system in *P. dabryanus*. In addition, a total of 22 significant QTLs were identified for the traits of growth, morphology, and sex. Interestingly, the most significant QTLs for almost all the traits related to growth, morphology (except for CPL), and sex were concentrated on the same LG LG11. Several potentially key markers and genes associated with growth and sex were detected in the regions of max LOD and overlapping QTL on LG11. Moreover, the first genome survey analysis of *P. dabryanus* was performed, which highlighted the high ratio of repetitive sequence and high level of heterozygosity in *P. dabryanus* genome. The genetic map was applied to anchor the genome sequences onto the chromosome, which effectively improve the accuracy and coverage of the scaffolds in the genome. The collinearity analysis revealed high level of collinearity between the genetic map and the reference genome in *P. dabryanus*. The comparative genomic analysis demonstrated a certain degree of homology between large-scale loach and zebrafish.

The present high-density genetic map and mapping results will provide essential resources for further genetic and genomic researches, and important basis for future MAS breeding of *P. dabryanus*.

## Data Availability Statement

The sequencing raw data of SLAF-seq have been submitted to the Sequence Read Archive (SRA) database of NCBI with the accession code PRJNA561042.

## Ethics Statement

The study was approved by the Institutional Animal Care and Use Ethics Committee of Huazhong Agricultural University. The permit number for conducting animal experiments of The Scientific Ethic Committee of Huazhong Agricultural University is HZAUFI-2016-006.

## Author Contributions

The authors’ responsibilities were as follows: JW conceived and designed the study. JW also drafted the manuscript. WW performed the manuscript revision. YC prepared the DNA samples. WW critically reviewed the manuscript. All authors read and approved the final manuscript.

## Funding

This work was financially supported by the National Natural Science Foundation of China (Grant number 31372180) and the Fundament Research Funds for the Central Universities (Grant number 2662015PY019).

## Conflict of Interest

The authors declare that the research was conducted in the absence of any commercial or financial relationships that could be construed as a potential conflict of interest.

## Abbreviations

GC, guanine-cytosine; MAS, marker-assisted selection; QTL, quantitative trait loci; NGS, next-generation sequencing; RAD-seq, restriction site associated DNA sequencing; GBS, genotyping-by-sequencing; SLAF, specific-locus amplified fragment; RRL, reduced representation library; bp, base pairs; LGs, linkage groups (); SDRs, segregation distortion regions (); BWA, Burrows–Wheeler Aligner.
