# Supplementary material for: A High-Density Genetic Linkage Map and QTL Mapping for Sex and Growth-Related Traits of Large-Scale Loach (Paramisgurnus dabryanus)
Source: Front Genet. 2019 Oct 25;10:1023. doi: 10.3389/fgene.2019.01023 (PMC6823184; doi:10.3389/fgene.2019.01023)
Supplement: Supplementary file 1 [file DataSheet_1.zip › Caption and Description of Figure S5.docx]

**File name:** Figure S5

**File format:** ZIP

**Caption of data:** The haplotype maps of the genetic map.

**Description of data:** The X-axis represents the 200 samples and the y axis represents the markers. Green represents the female parent, blue represents male parent, red indicates heterozygosity, and gray indicates missing data. The color changed in the same column represents the occurrence of recombination events.
