# Supplementary figures and images for: A High-Density Genetic Linkage Map and QTL Mapping for Sex and Growth-Related Traits of Large-Scale Loach (Paramisgurnus dabryanus) (part 1 of 2)
Source: Front Genet. 2019 Oct 25;10:1023. doi: 10.3389/fgene.2019.01023 (PMC6823184; doi:10.3389/fgene.2019.01023)

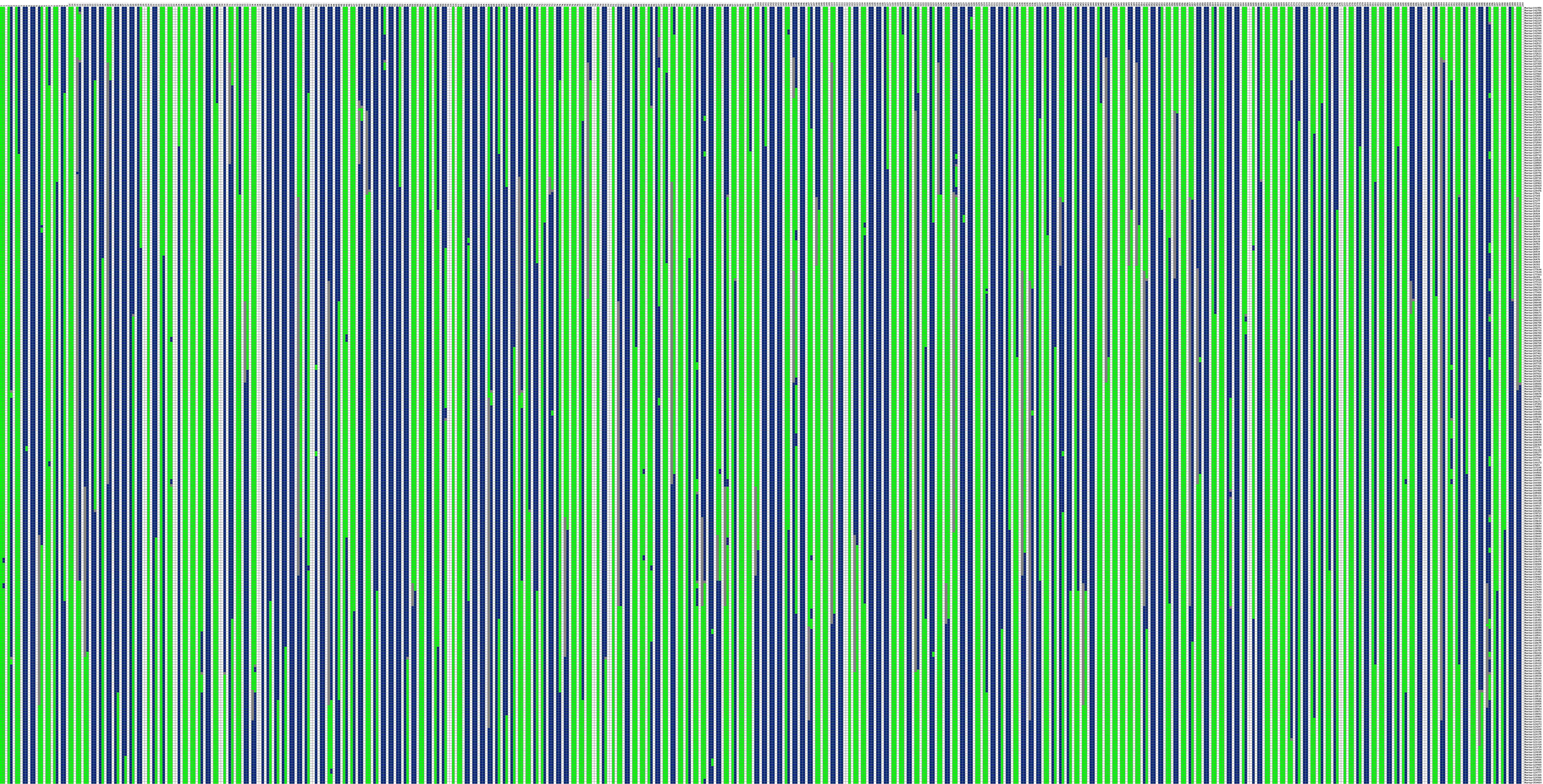

Supplement: Supplementary file 1 [file DataSheet_1.zip › Figure S5/female/LG1.female.haplo.png]

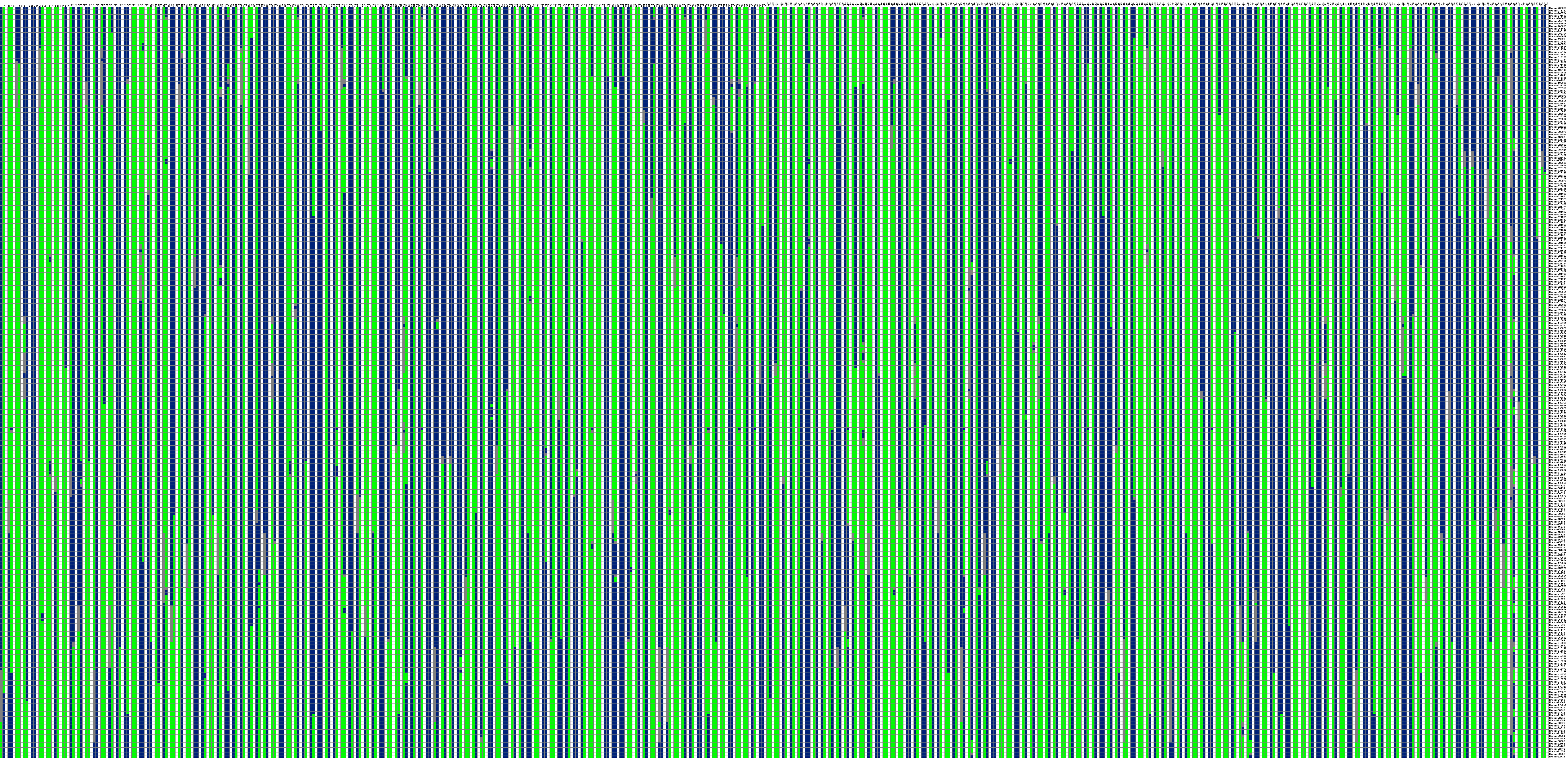

Supplement: Supplementary file 1 [file DataSheet_1.zip › Figure S5/female/LG10.female.haplo.png]

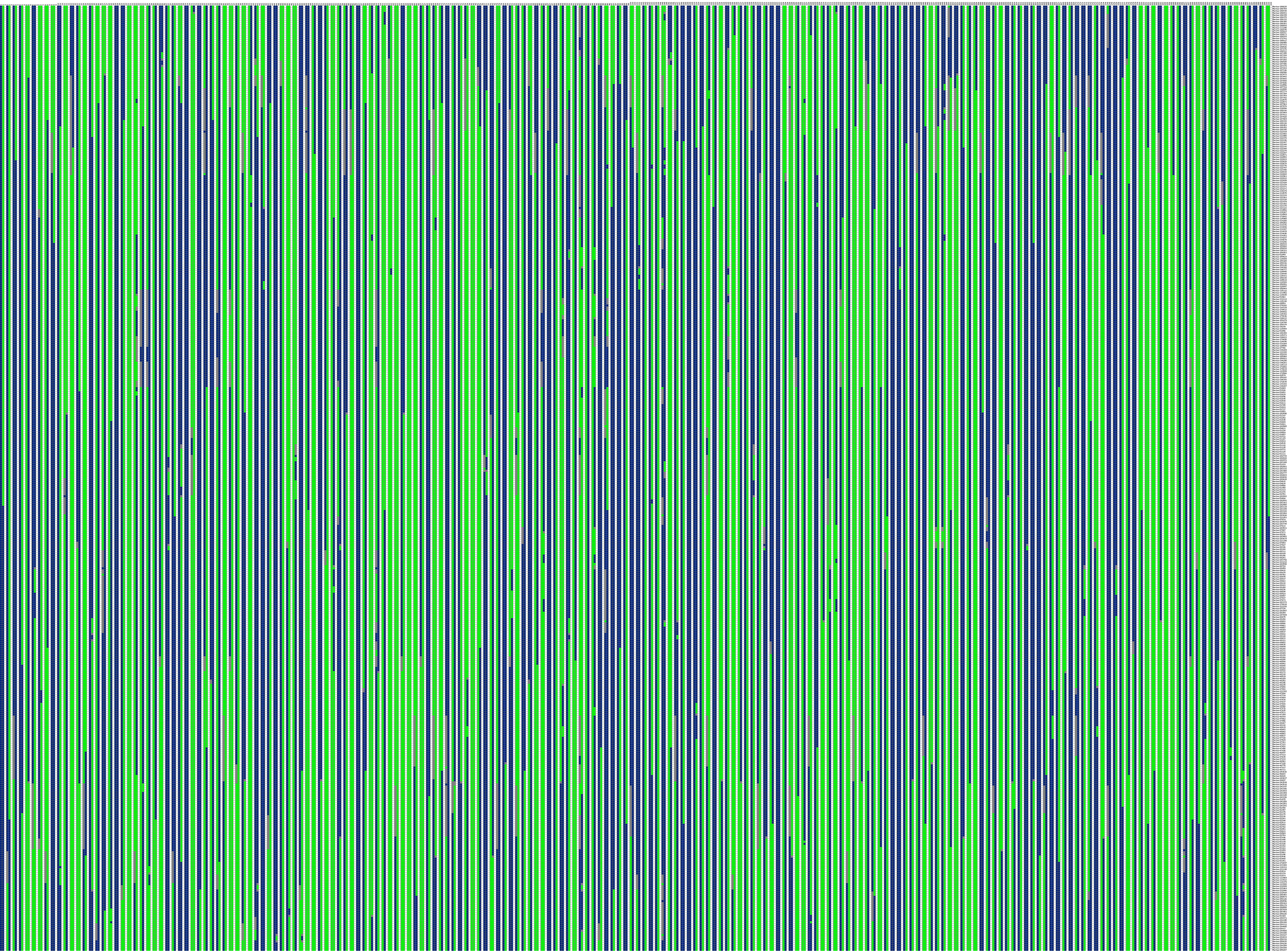

Supplement: Supplementary file 1 [file DataSheet_1.zip › Figure S5/female/LG11.female.haplo.png]

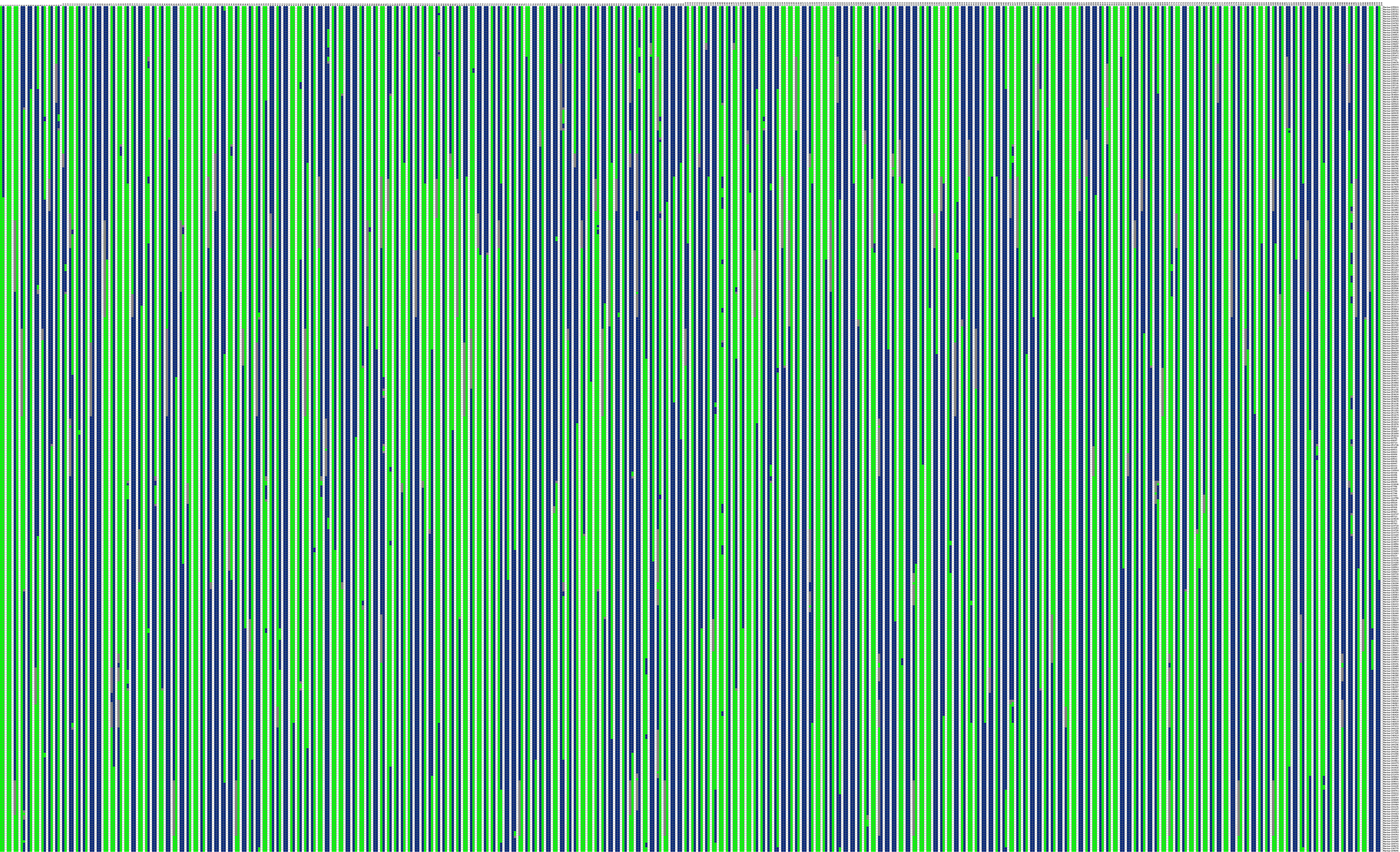

Supplement: Supplementary file 1 [file DataSheet_1.zip › Figure S5/female/LG12.female.haplo.png]

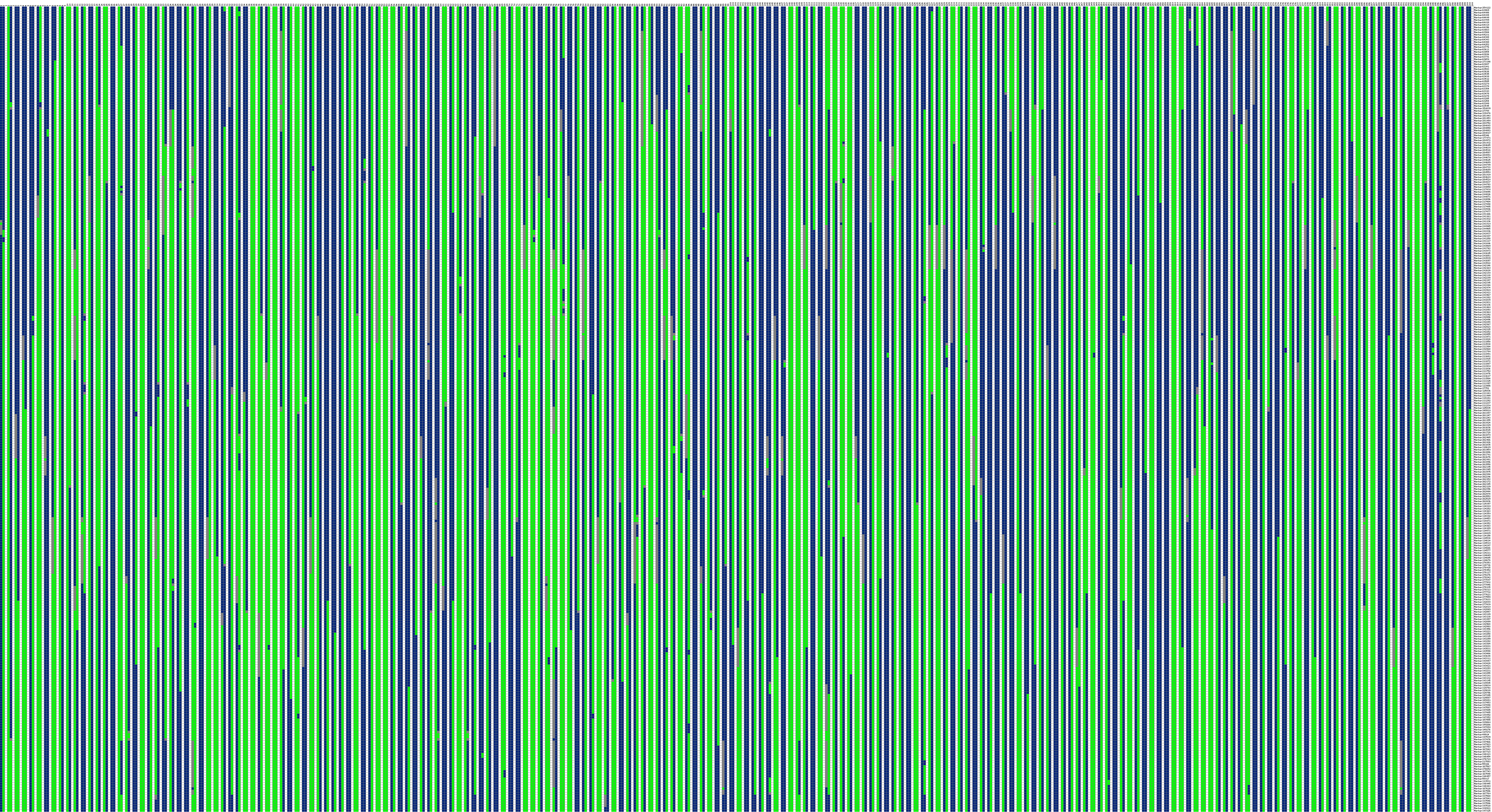

Supplement: Supplementary file 1 [file DataSheet_1.zip › Figure S5/female/LG13.female.haplo.png]

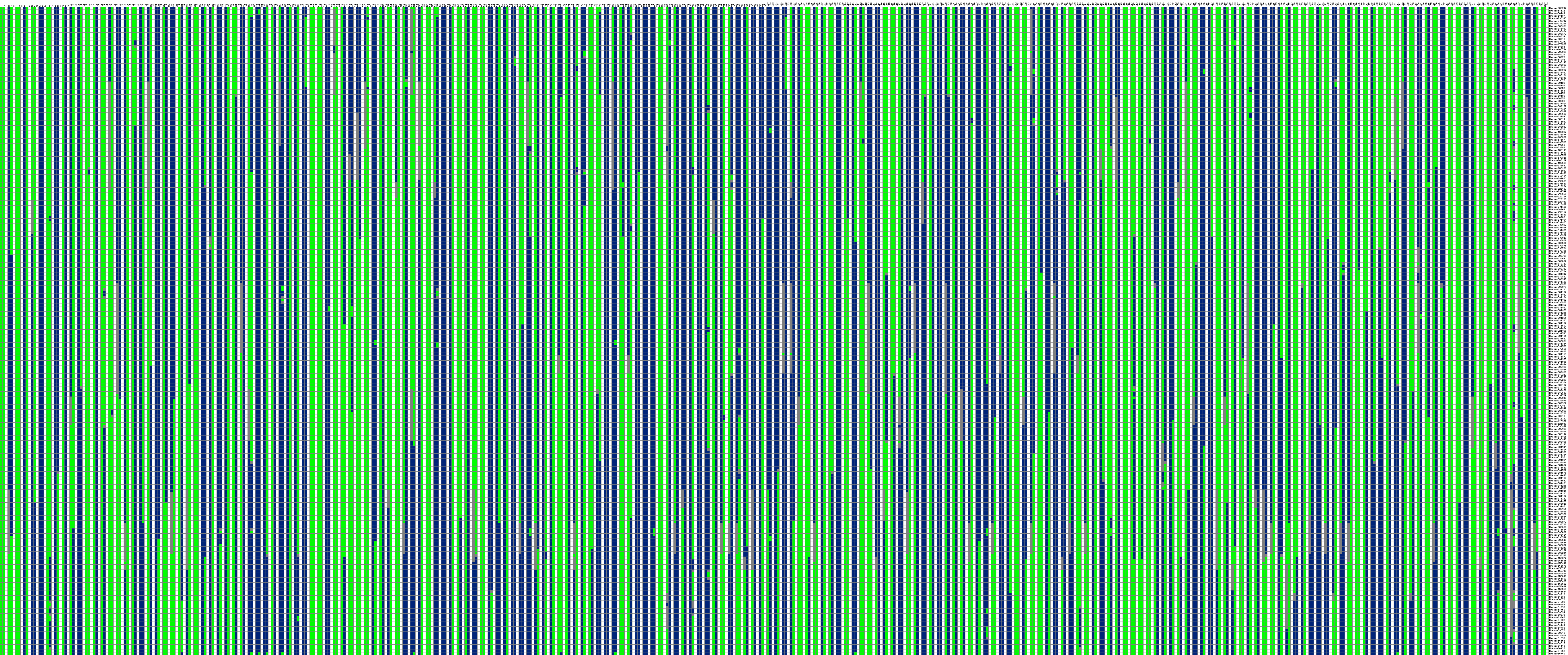

Supplement: Supplementary file 1 [file DataSheet_1.zip › Figure S5/female/LG14.female.haplo.png]

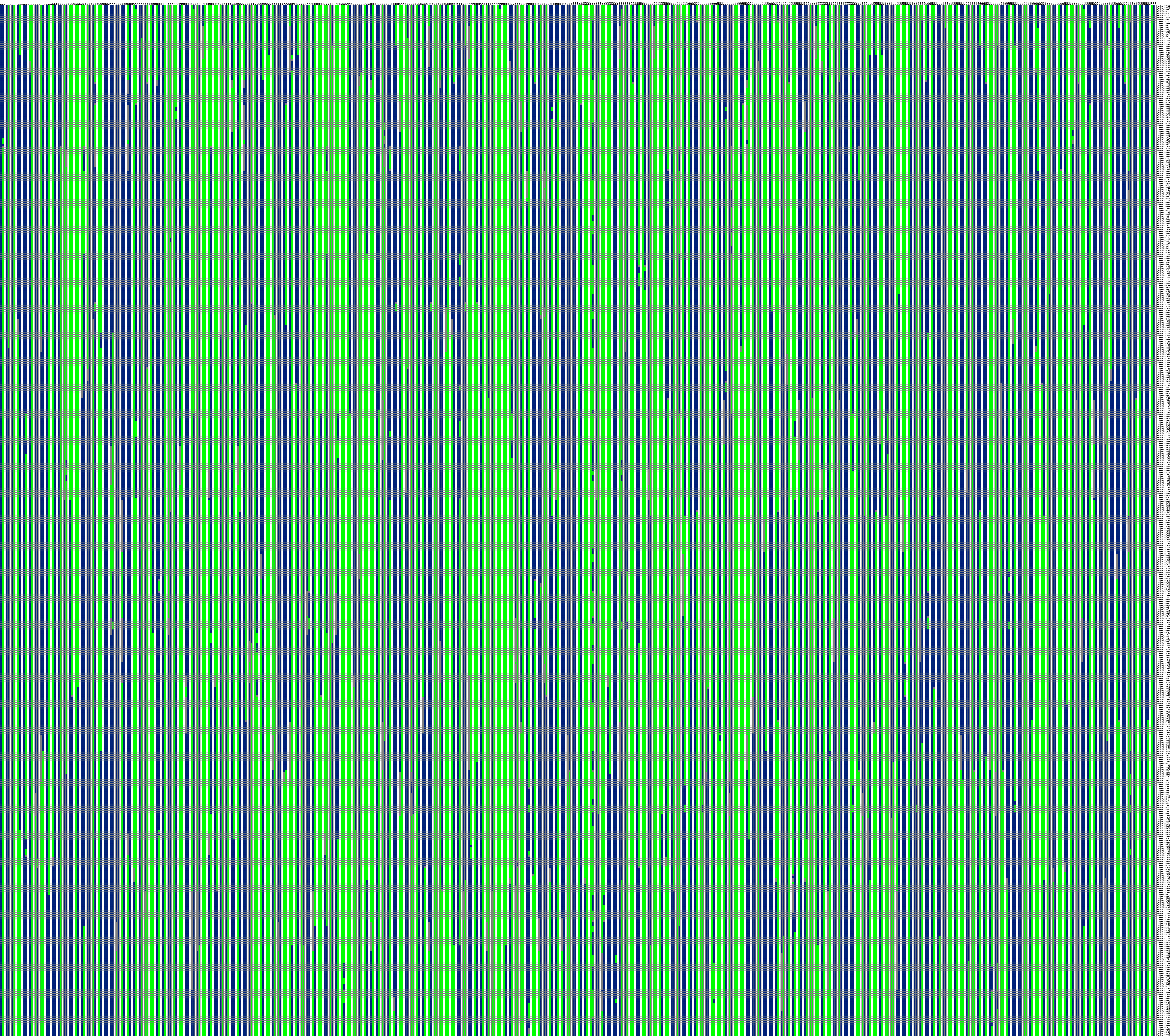

Supplement: Supplementary file 1 [file DataSheet_1.zip › Figure S5/female/LG15.female.haplo.png]

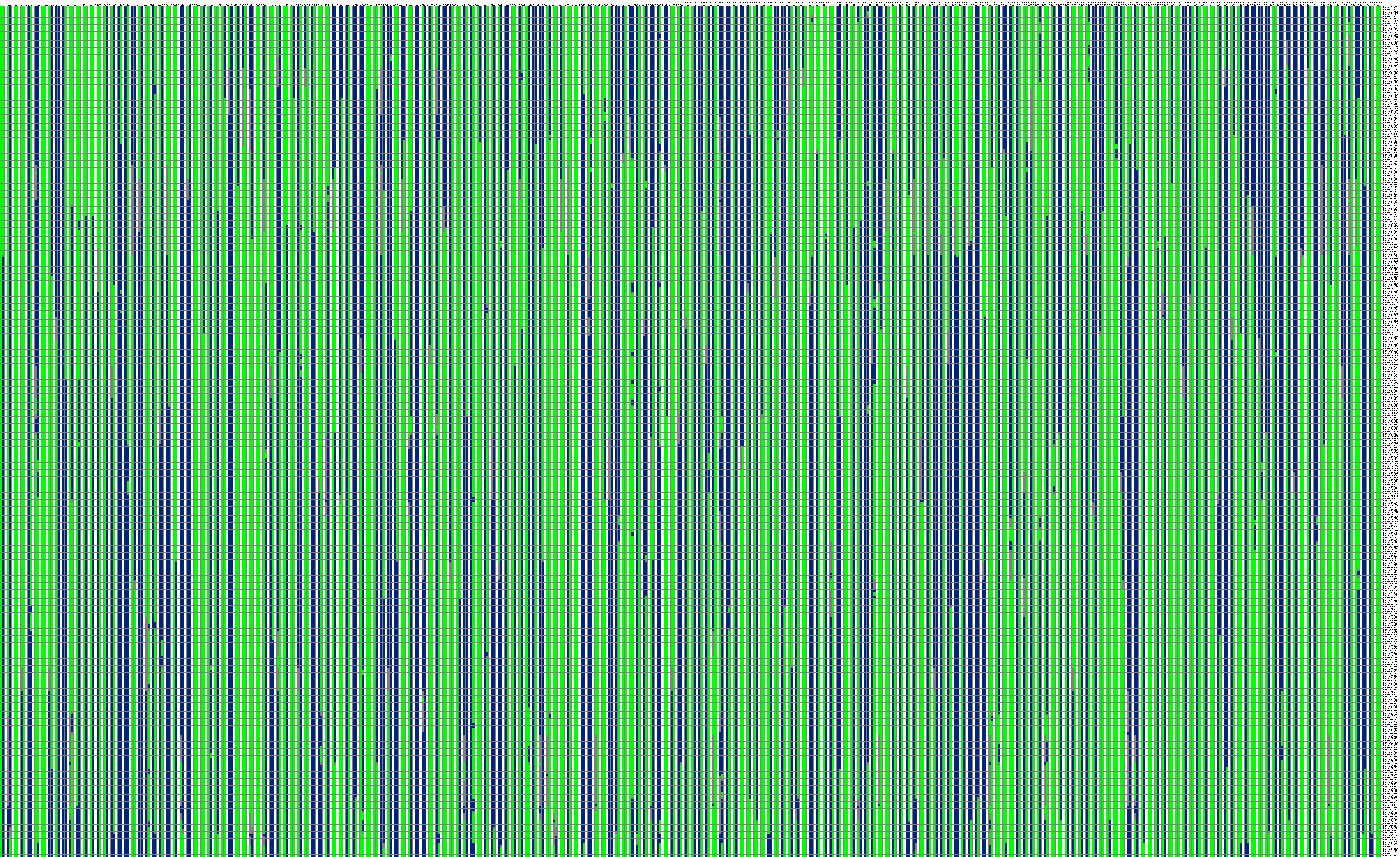

Supplement: Supplementary file 1 [file DataSheet_1.zip › Figure S5/female/LG16.female.haplo.png]

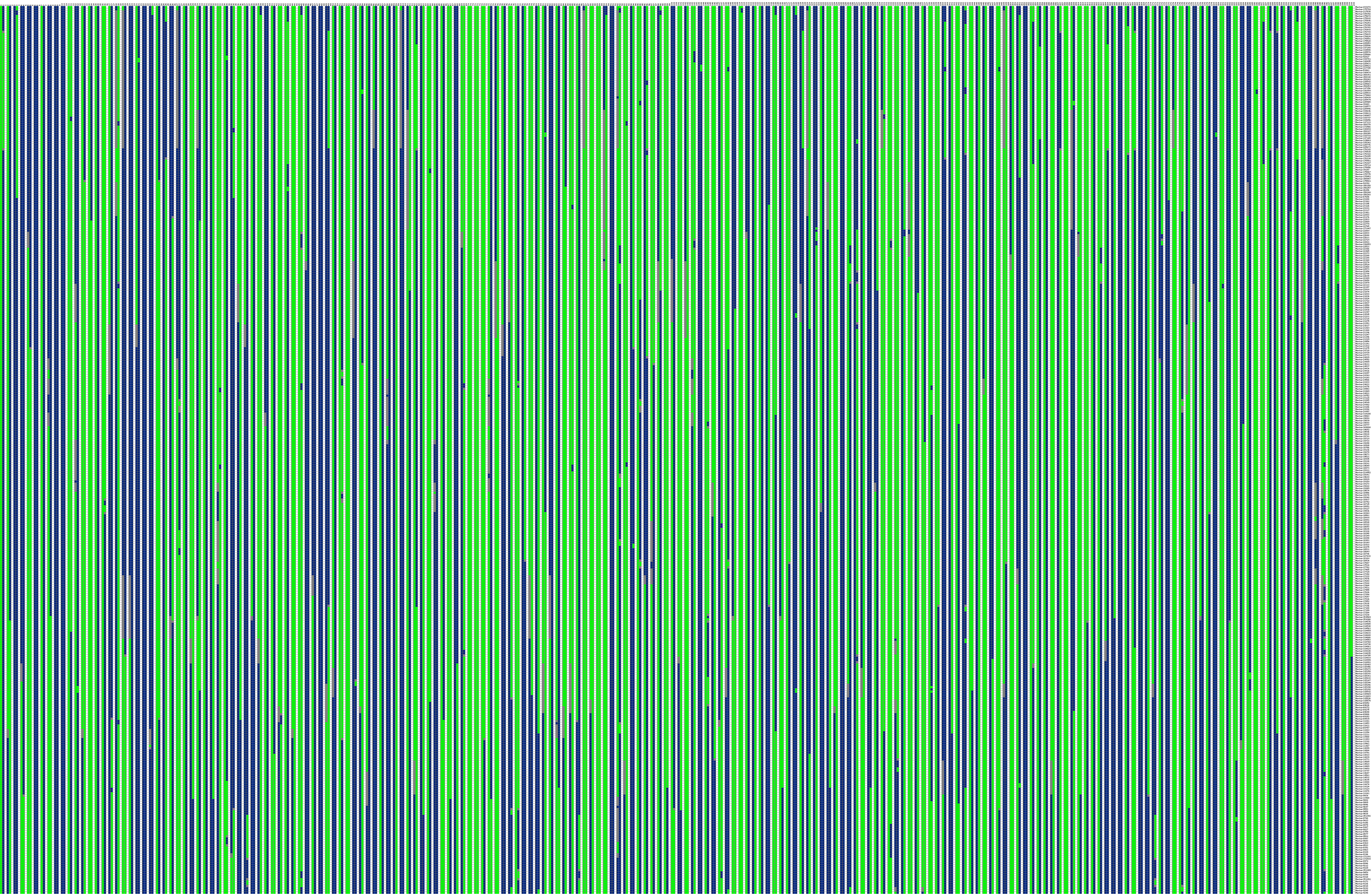

Supplement: Supplementary file 1 [file DataSheet_1.zip › Figure S5/female/LG17.female.haplo.png]

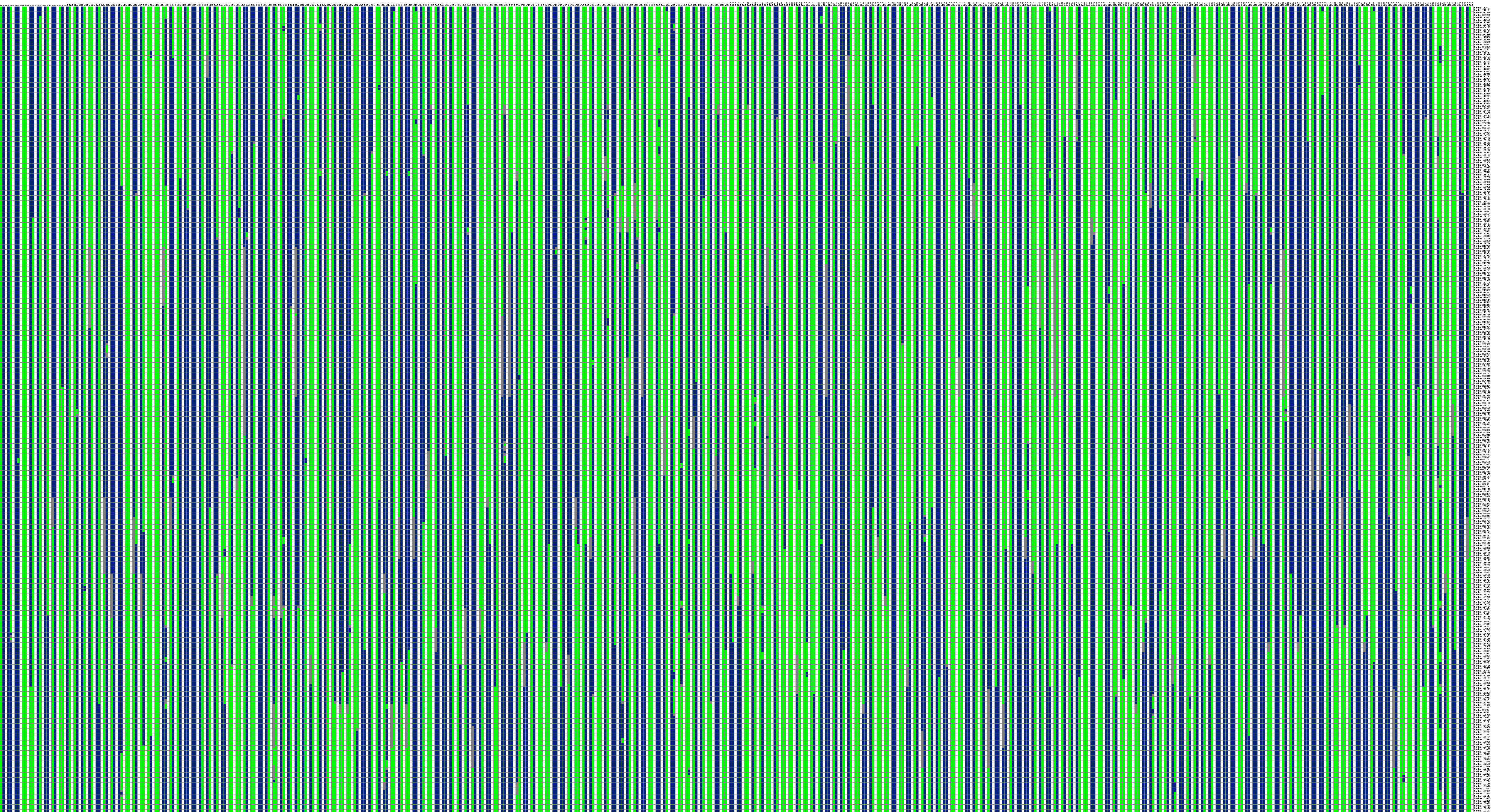

Supplement: Supplementary file 1 [file DataSheet_1.zip › Figure S5/female/LG18.female.haplo.png]

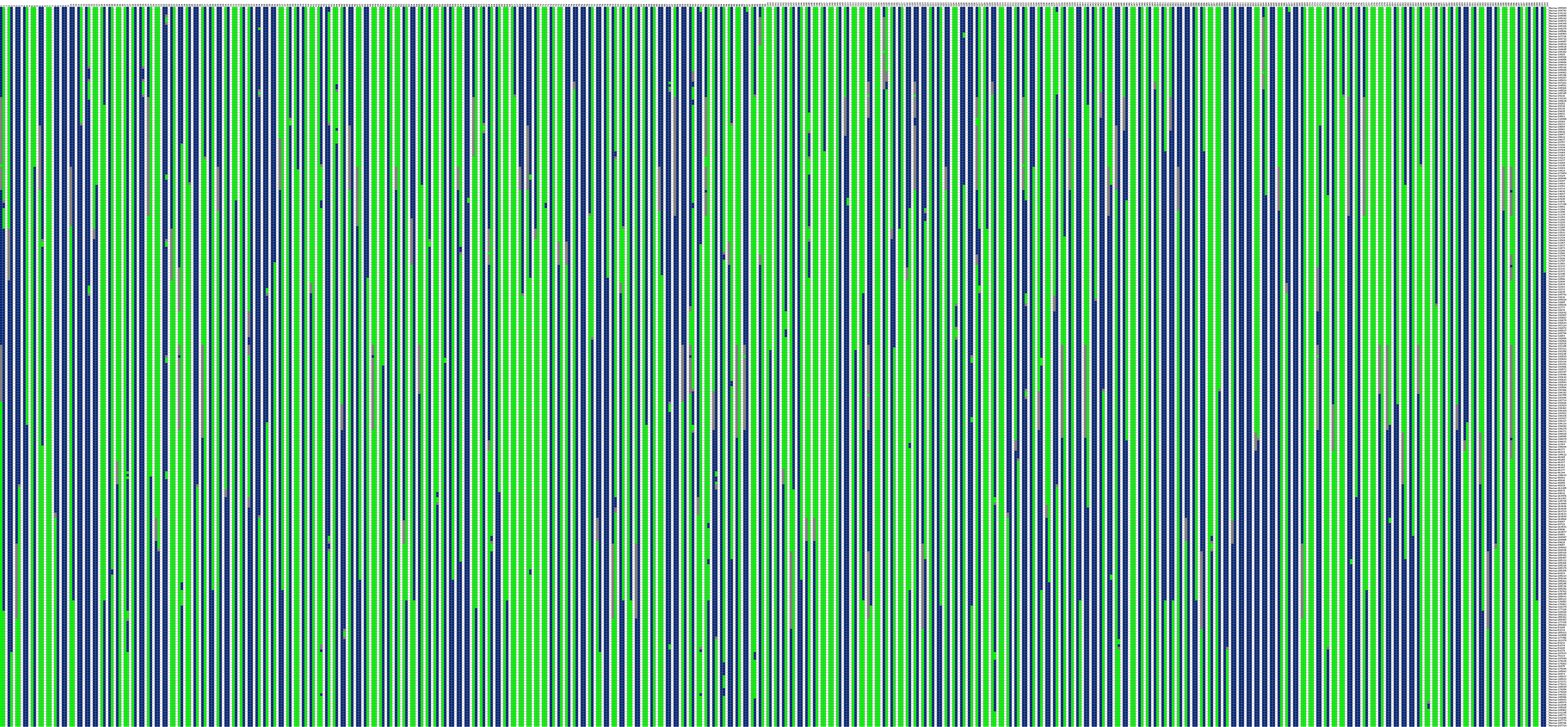

Supplement: Supplementary file 1 [file DataSheet_1.zip › Figure S5/female/LG19.female.haplo.png]

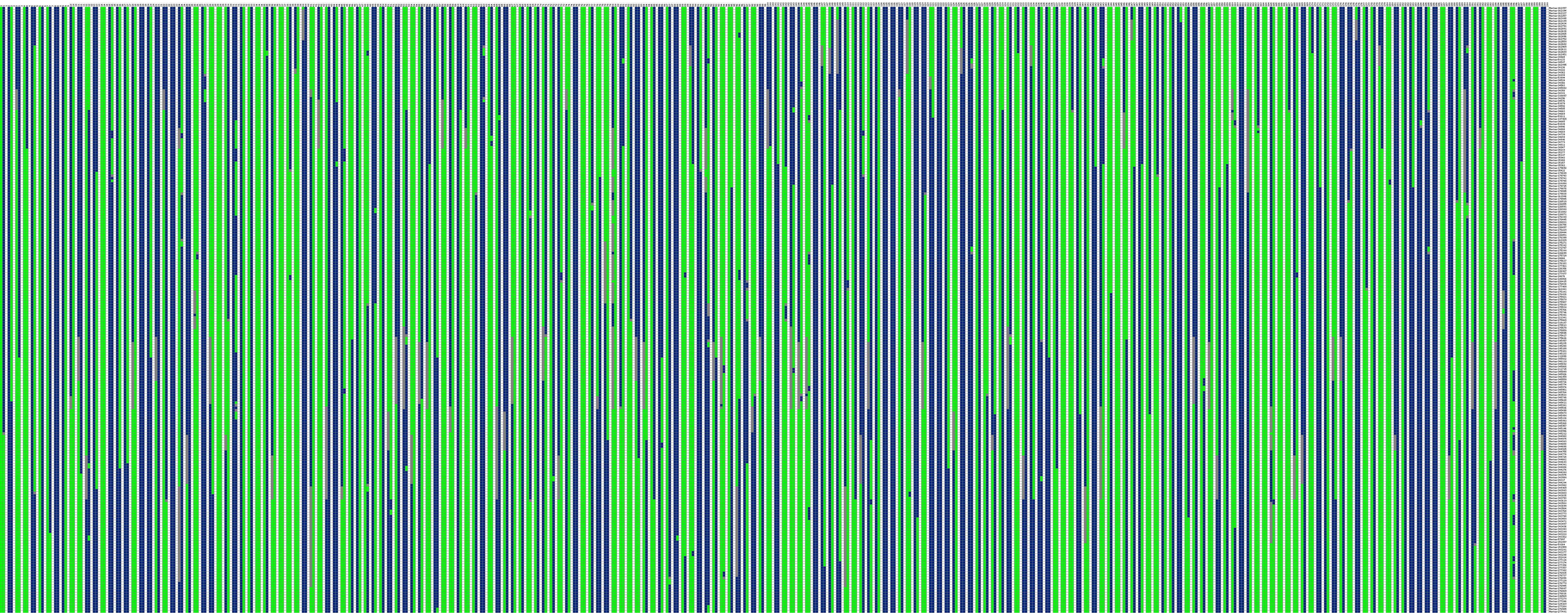

Supplement: Supplementary file 1 [file DataSheet_1.zip › Figure S5/female/LG2.female.haplo.png]

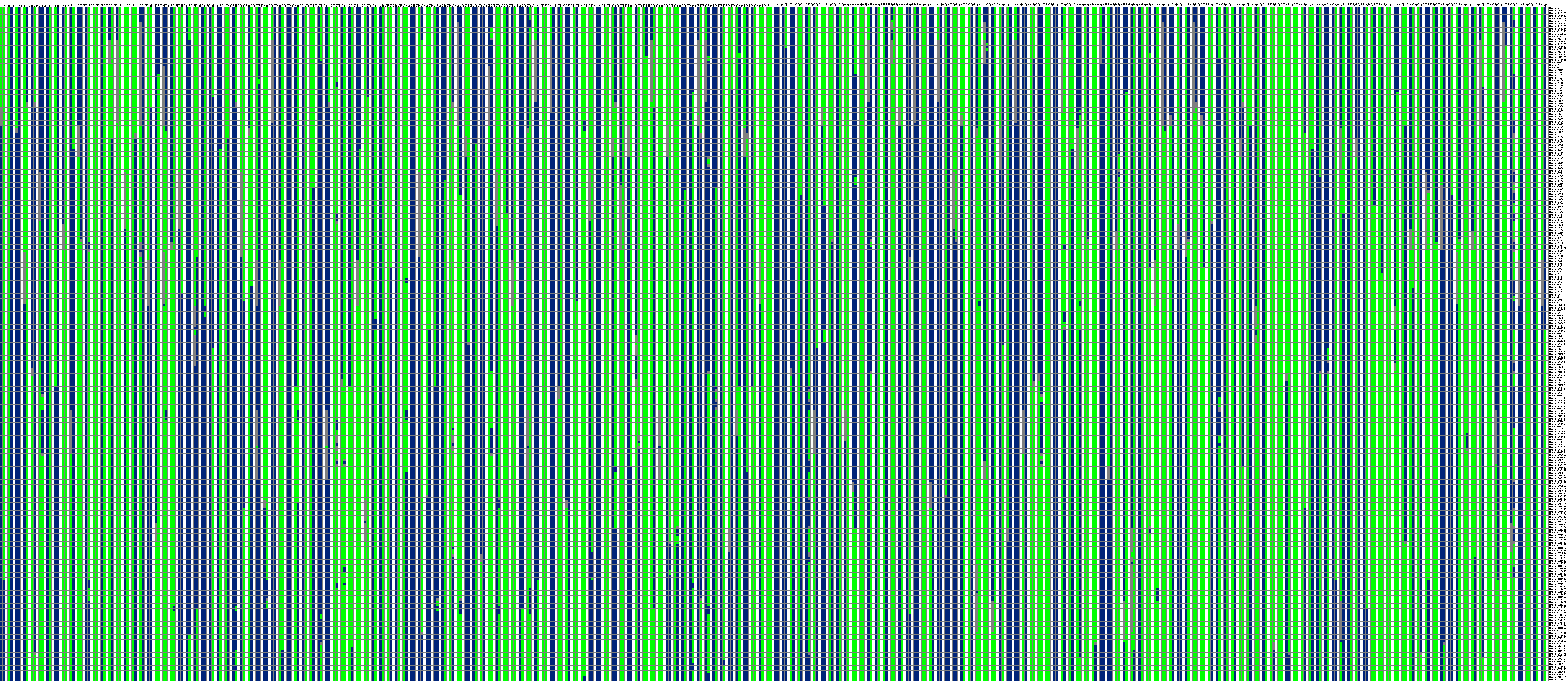

Supplement: Supplementary file 1 [file DataSheet_1.zip › Figure S5/female/LG20.female.haplo.png]

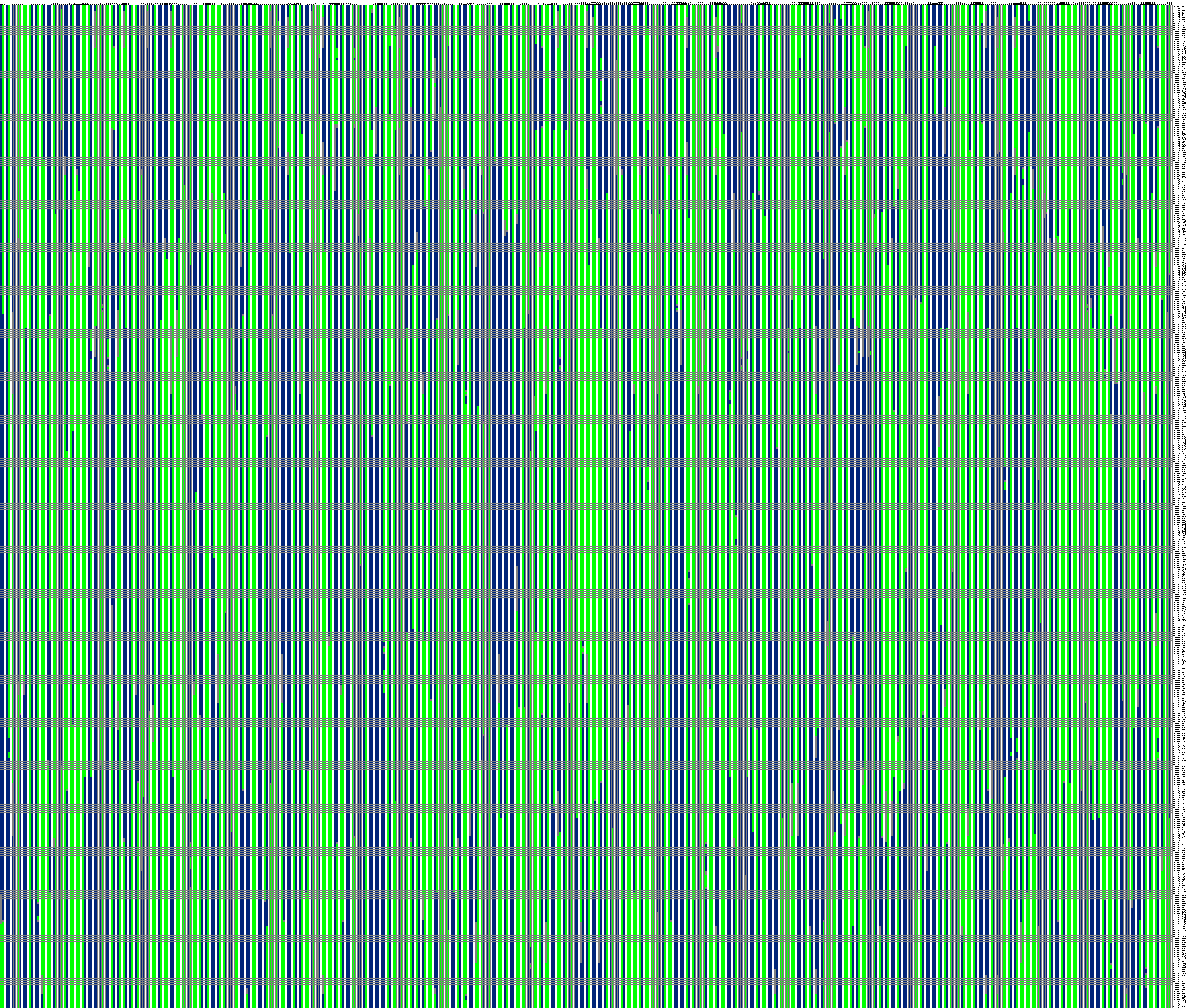

Supplement: Supplementary file 1 [file DataSheet_1.zip › Figure S5/female/LG21.female.haplo.png]

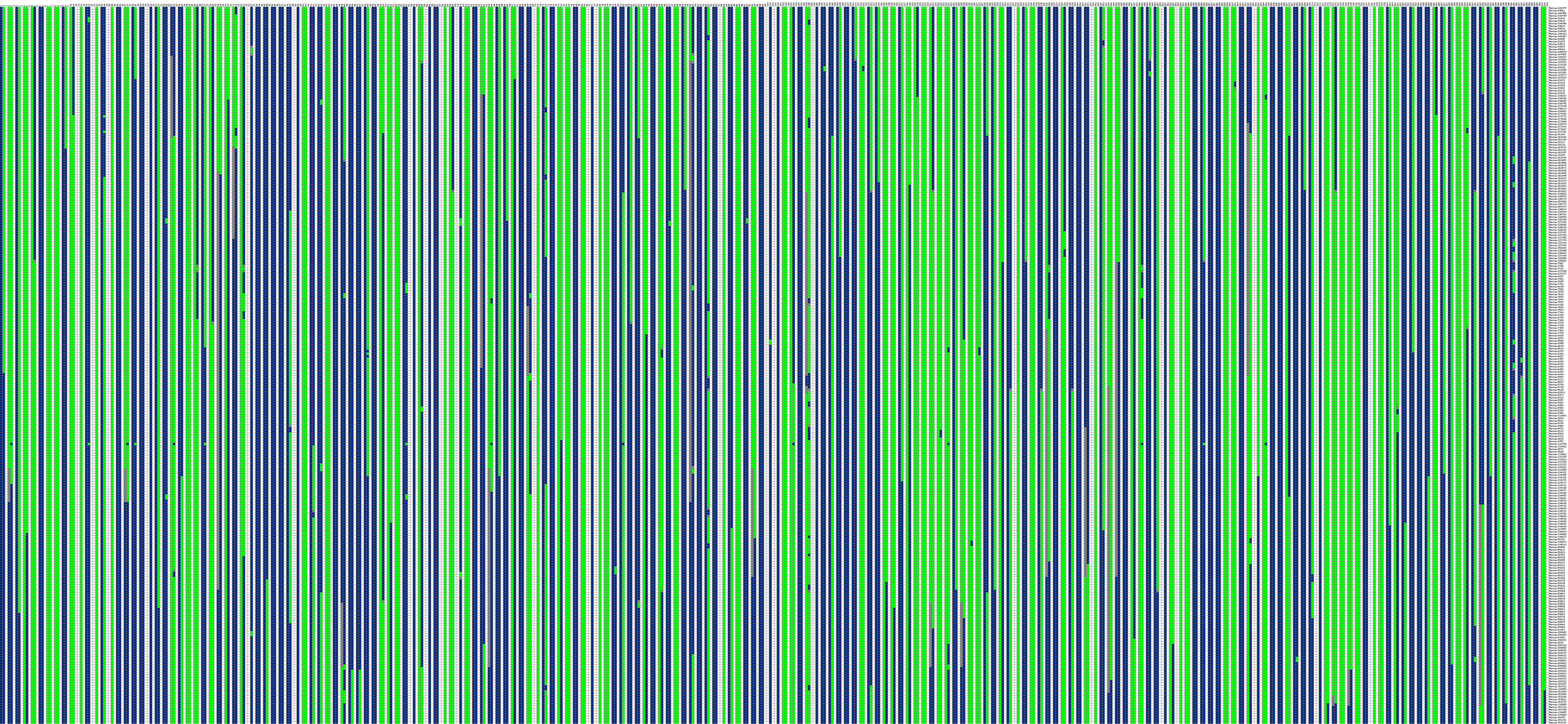

Supplement: Supplementary file 1 [file DataSheet_1.zip › Figure S5/female/LG22.female.haplo.png]

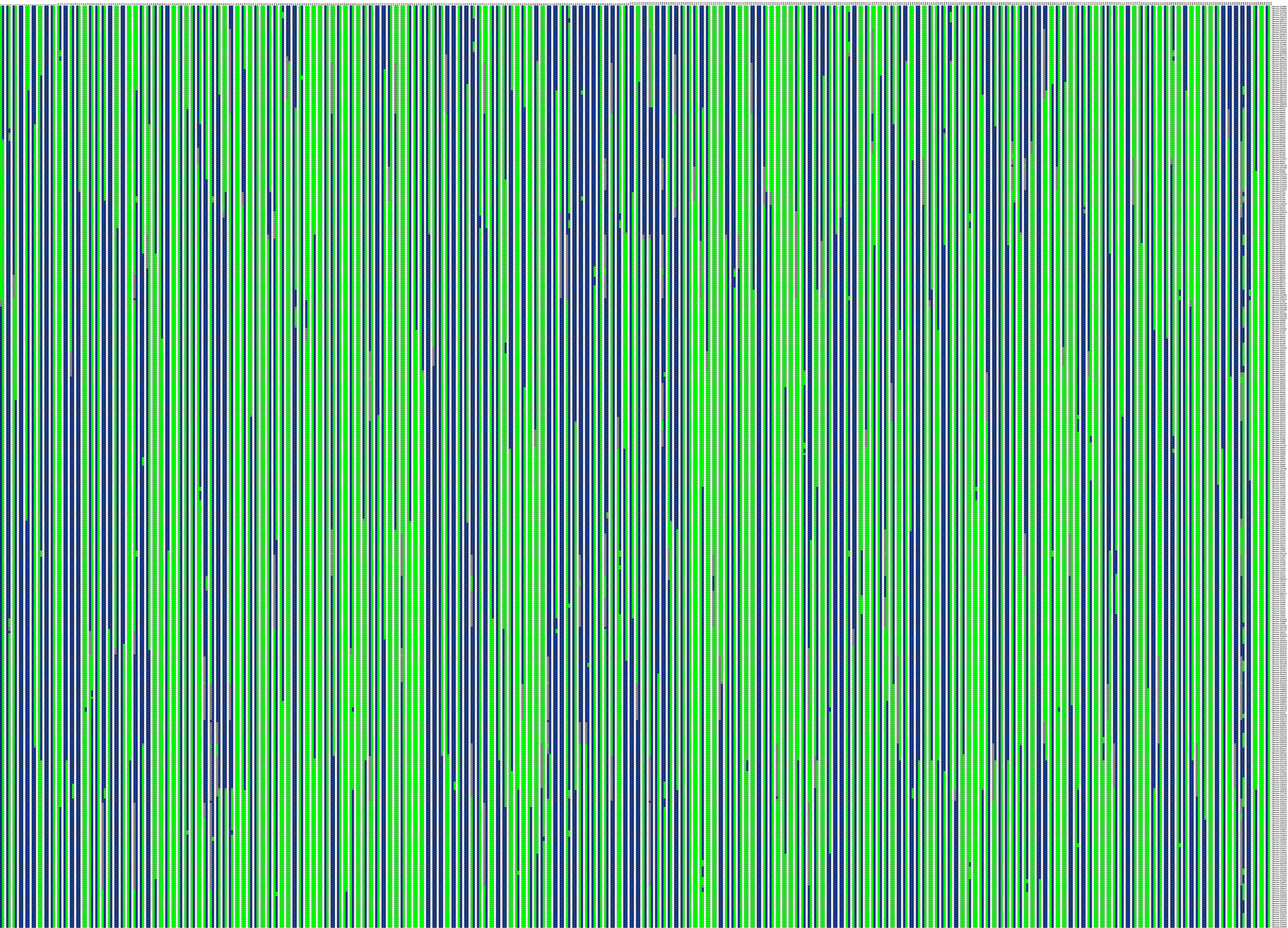

Supplement: Supplementary file 1 [file DataSheet_1.zip › Figure S5/female/LG23.female.haplo.png]

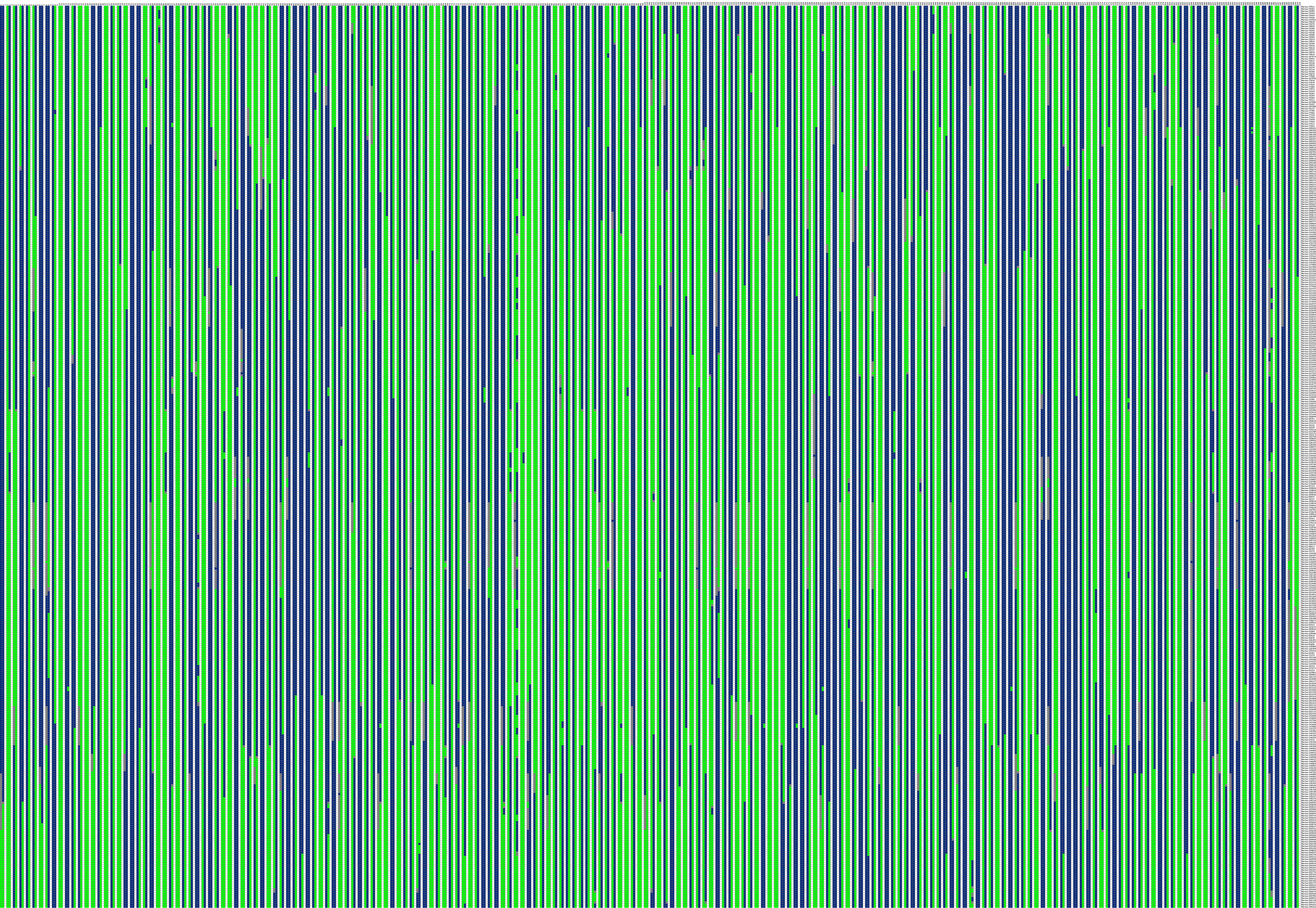

Supplement: Supplementary file 1 [file DataSheet_1.zip › Figure S5/female/LG24.female.haplo.png]

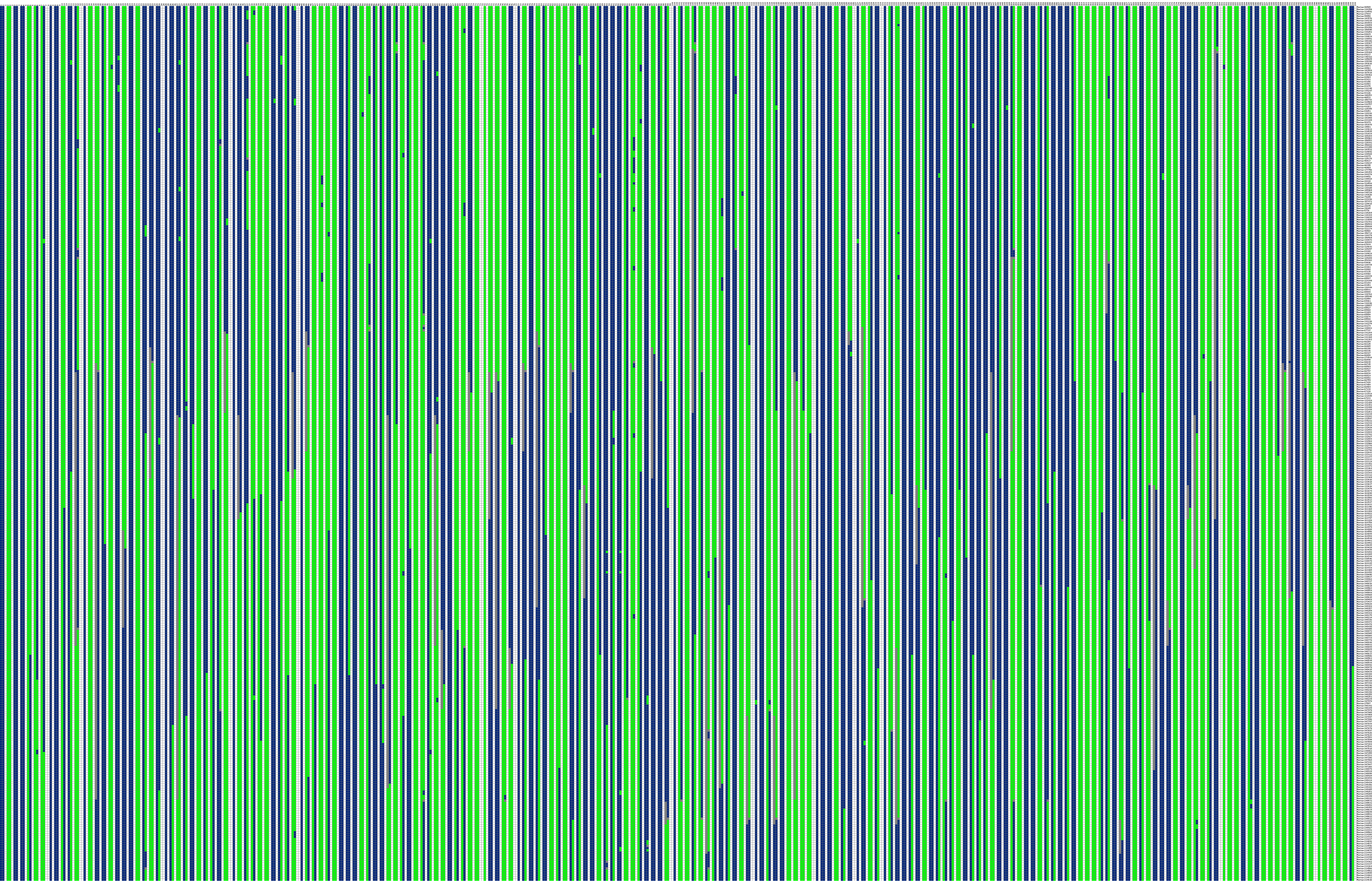

Supplement: Supplementary file 1 [file DataSheet_1.zip › Figure S5/female/LG3.female.haplo.png]

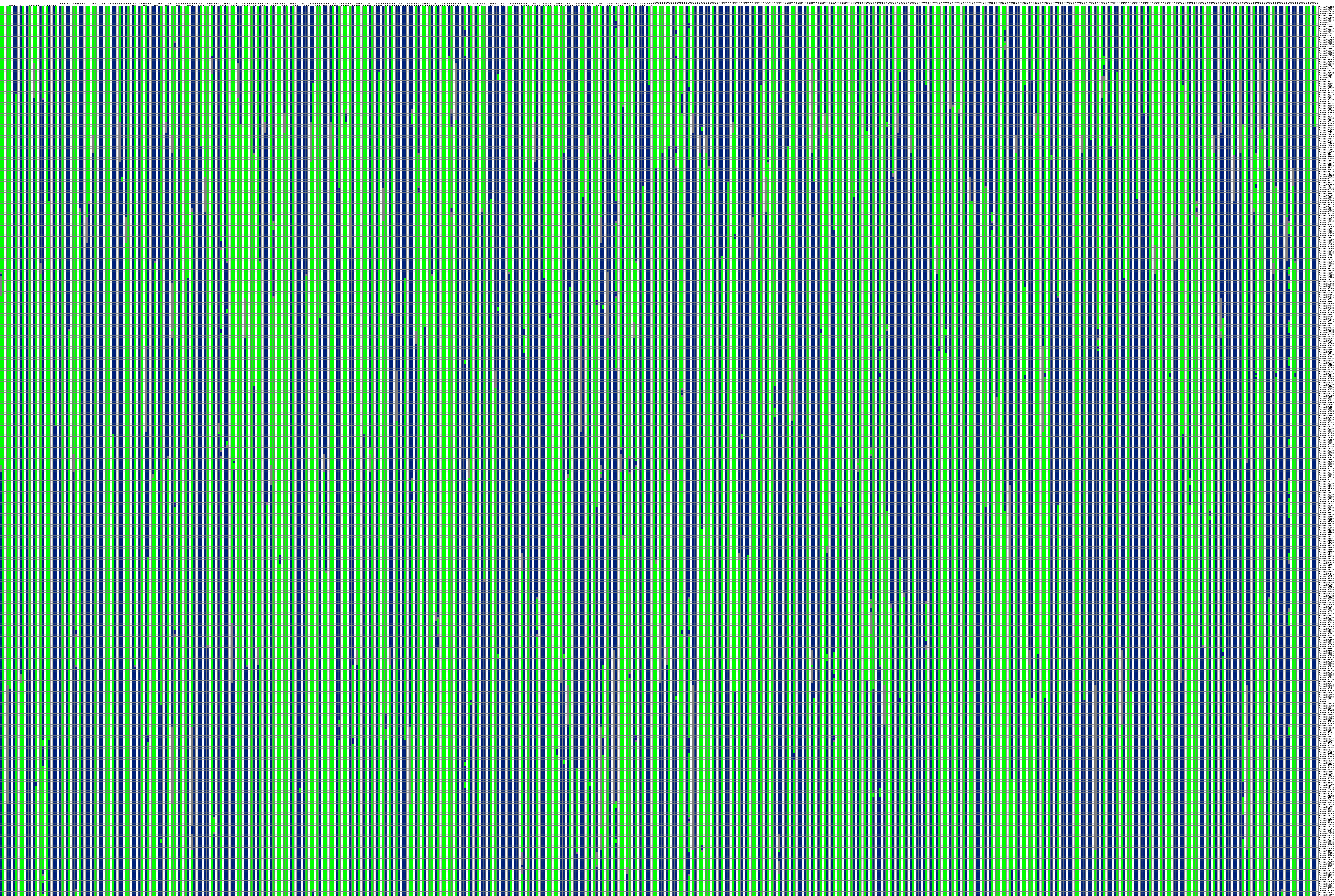

Supplement: Supplementary file 1 [file DataSheet_1.zip › Figure S5/female/LG4.female.haplo.png]

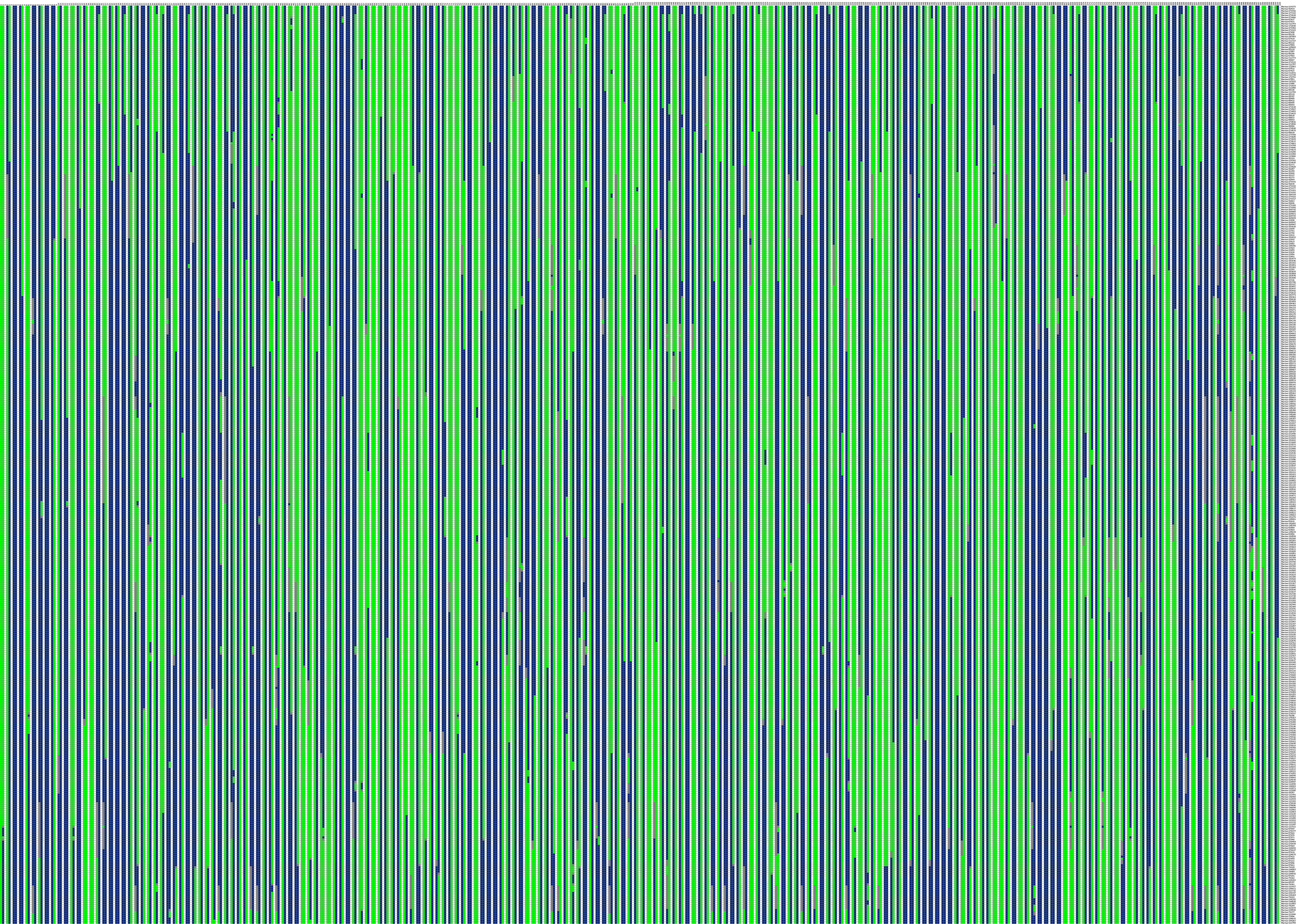

Supplement: Supplementary file 1 [file DataSheet_1.zip › Figure S5/female/LG5.female.haplo.png]

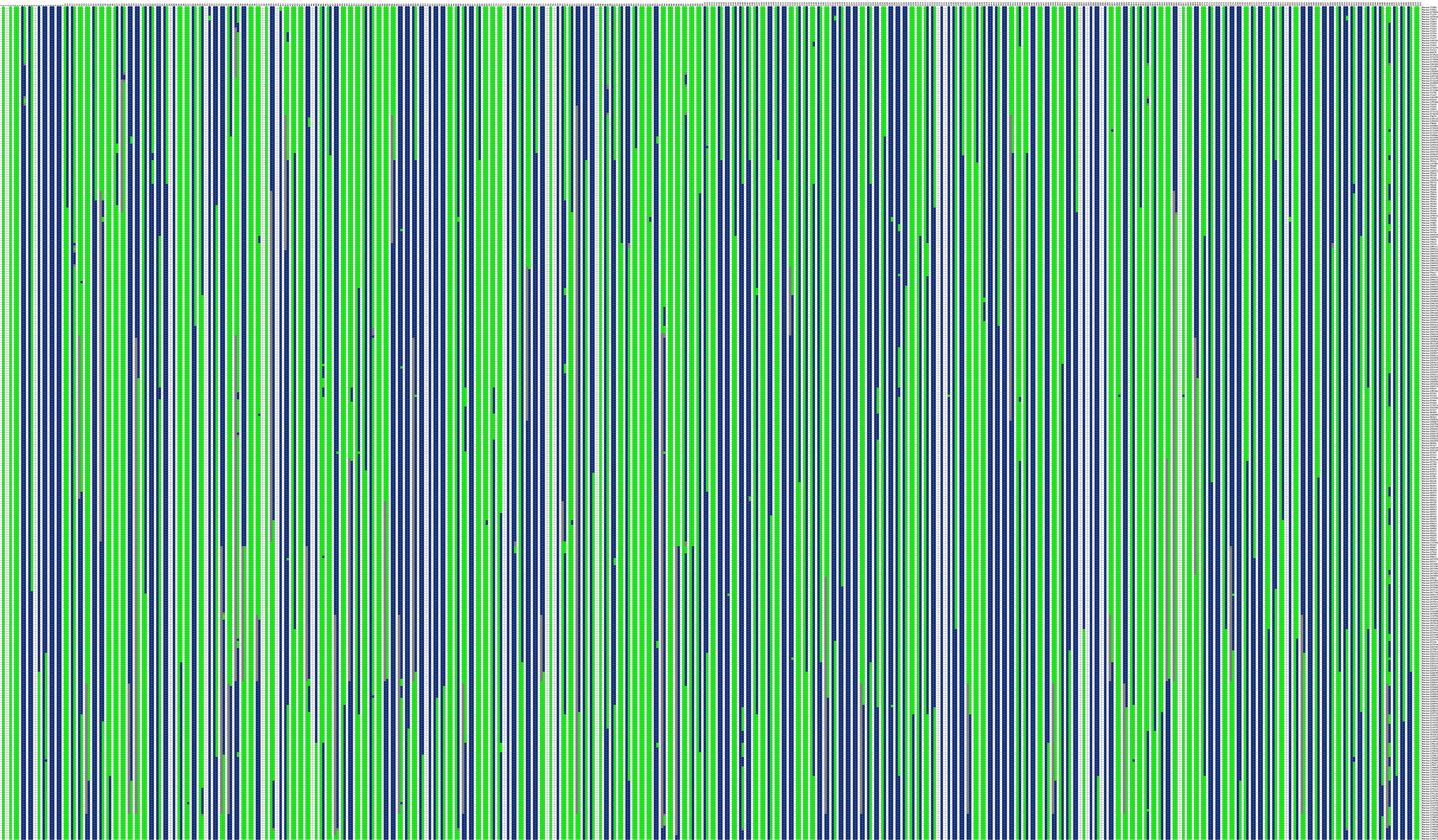

Supplement: Supplementary file 1 [file DataSheet_1.zip › Figure S5/female/LG6.female.haplo.png]

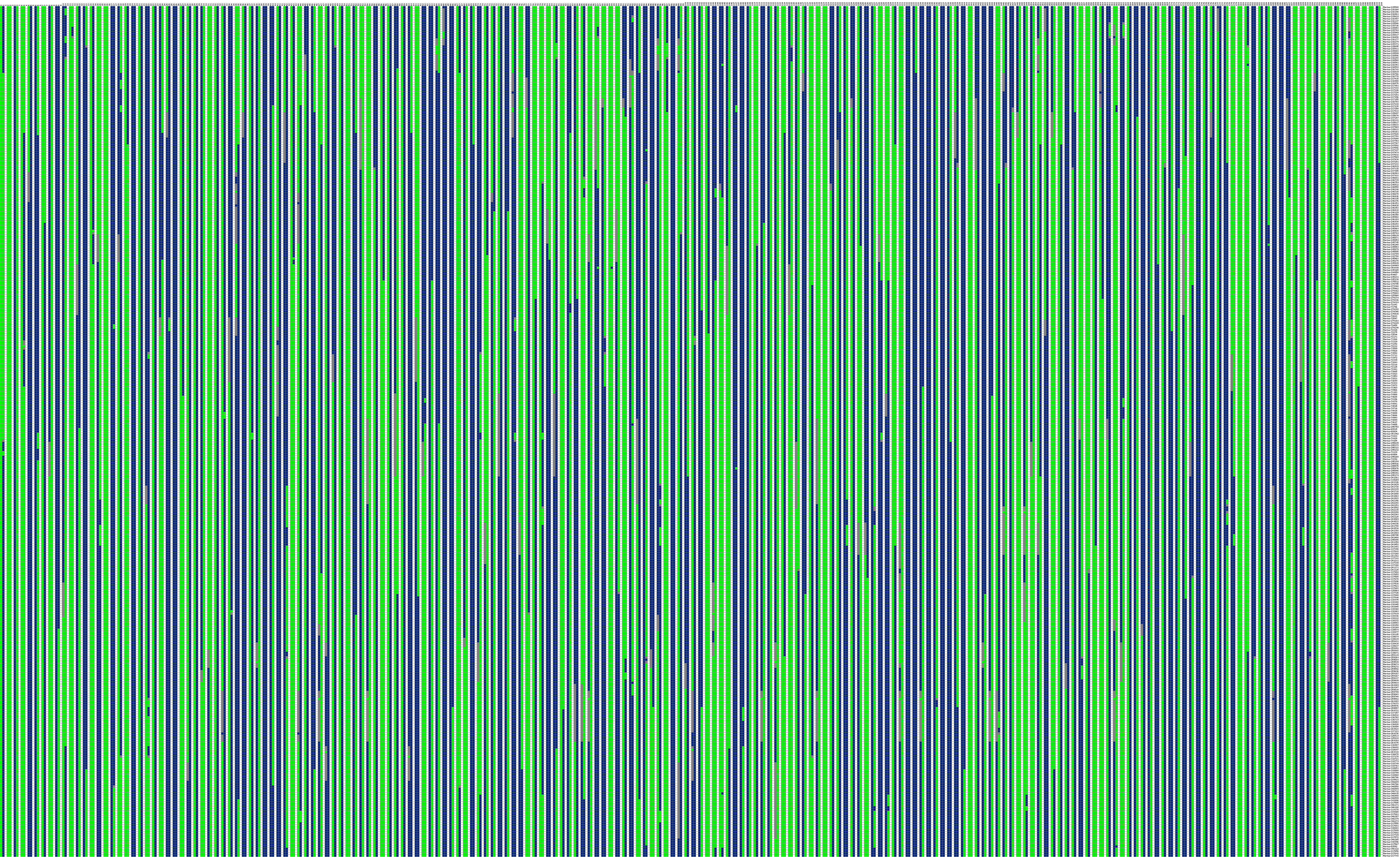

Supplement: Supplementary file 1 [file DataSheet_1.zip › Figure S5/female/LG7.female.haplo.png]

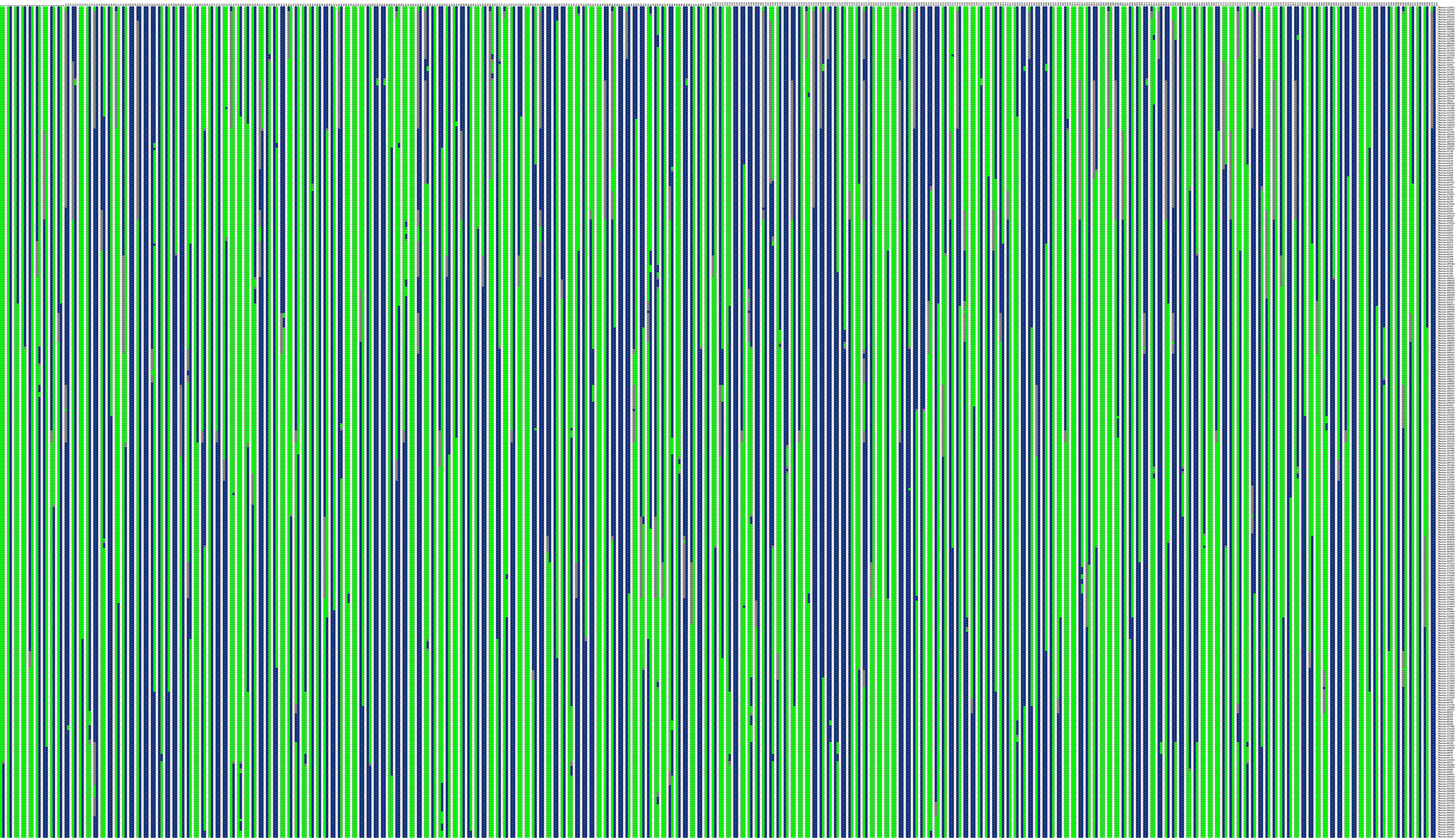

Supplement: Supplementary file 1 [file DataSheet_1.zip › Figure S5/female/LG8.female.haplo.png]

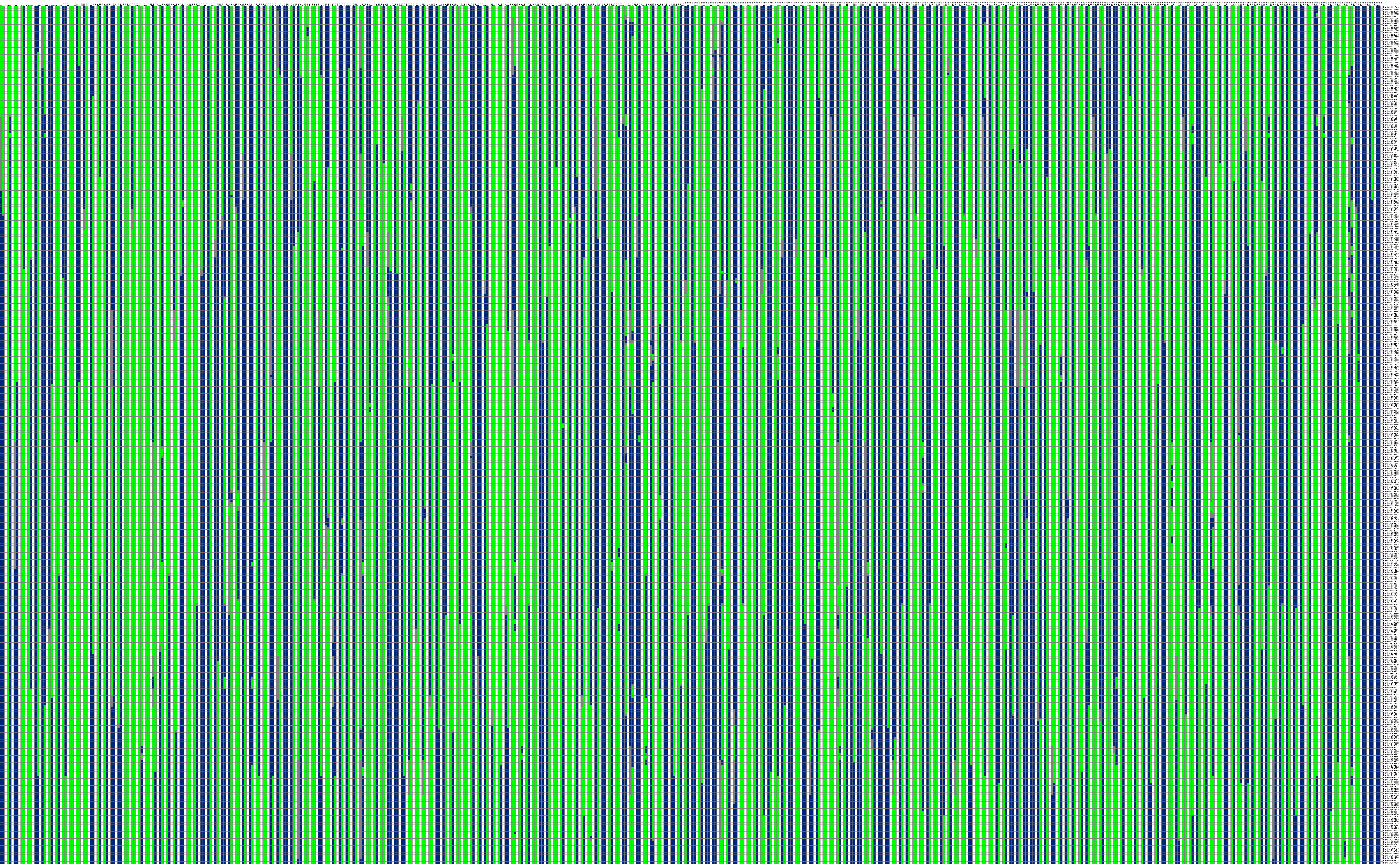

Supplement: Supplementary file 1 [file DataSheet_1.zip › Figure S5/female/LG9.female.haplo.png]

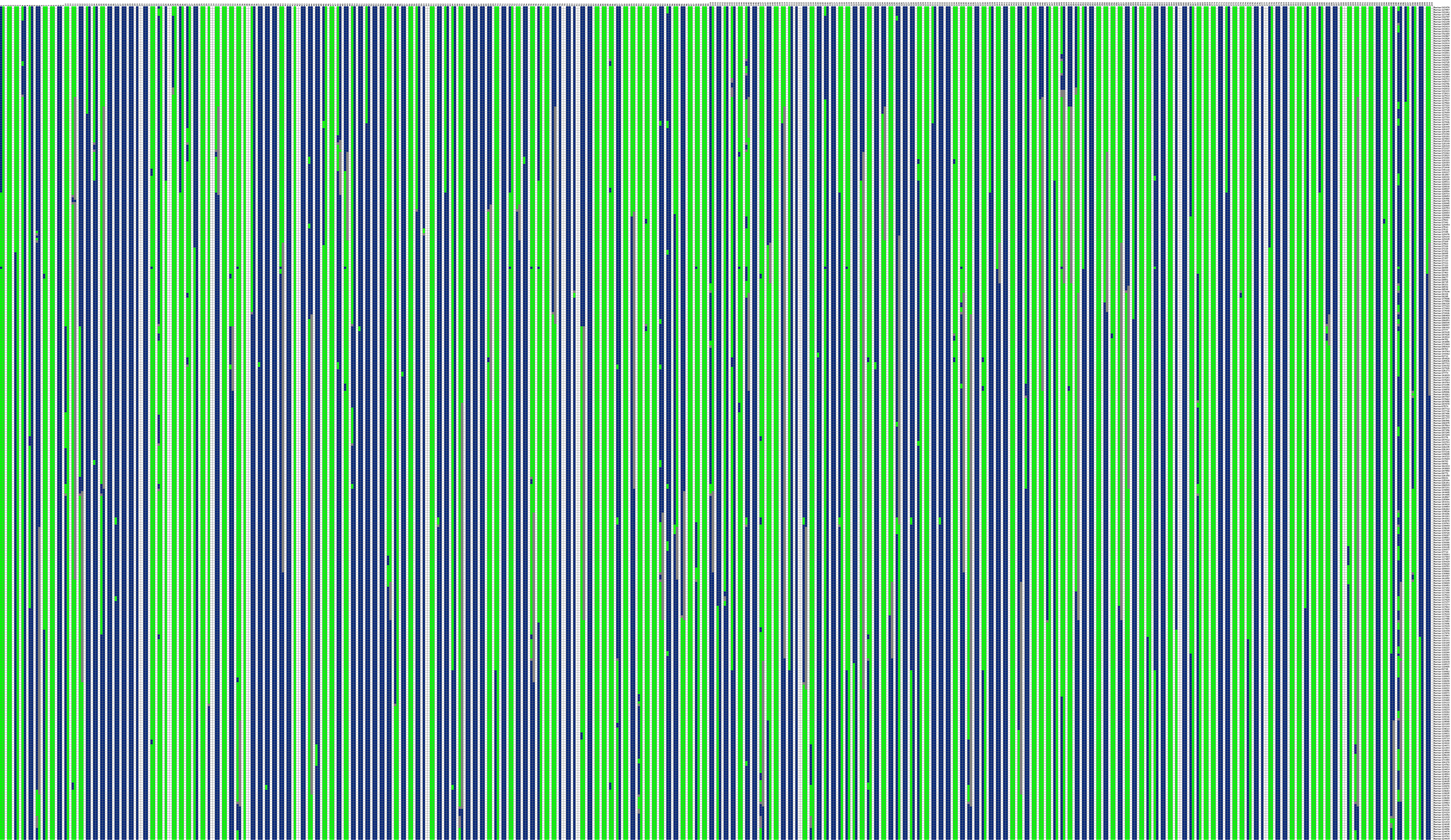

Supplement: Supplementary file 1 [file DataSheet_1.zip › Figure S5/male/LG1.male.haplo.png]

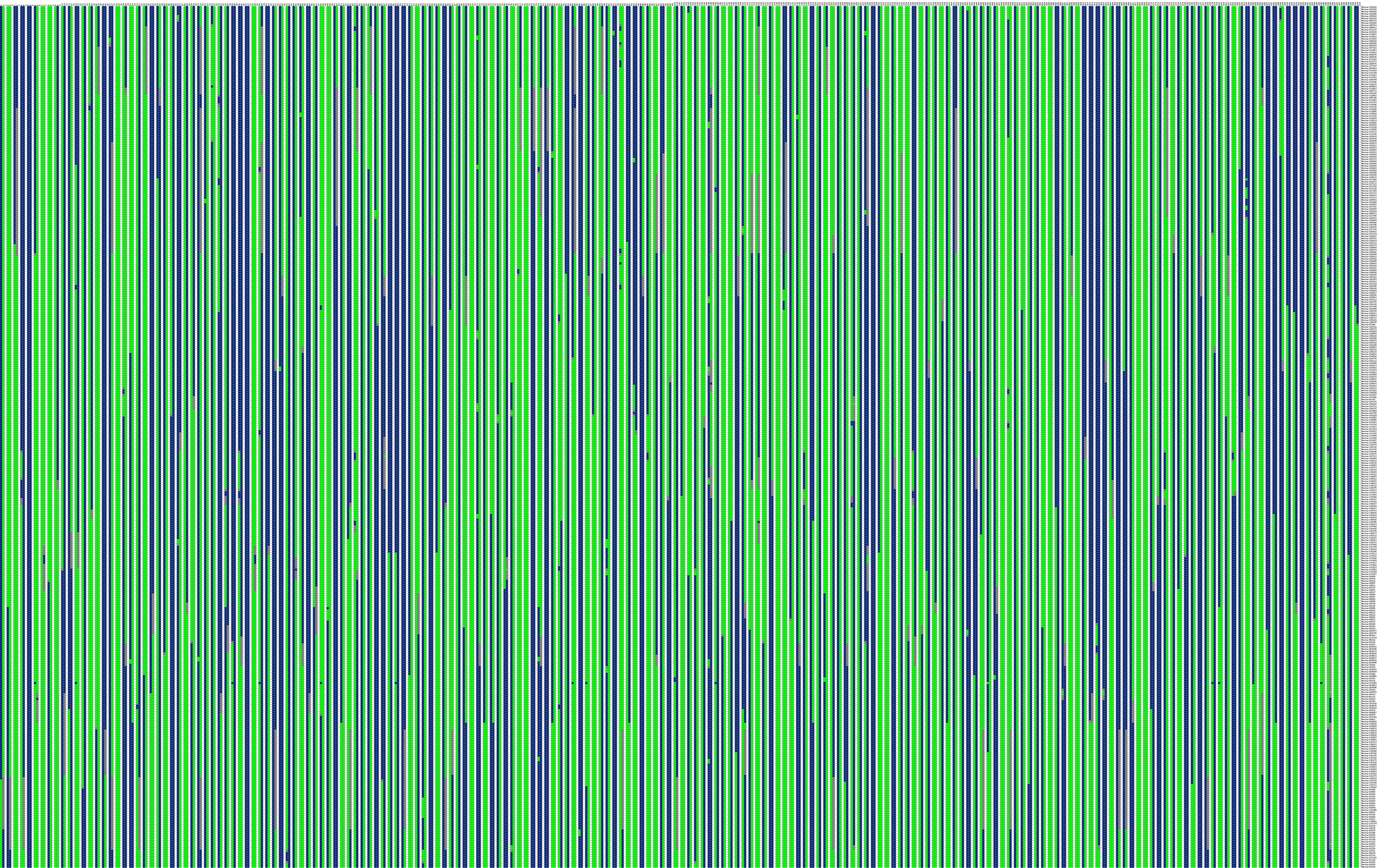

Supplement: Supplementary file 1 [file DataSheet_1.zip › Figure S5/male/LG10.male.haplo.png]

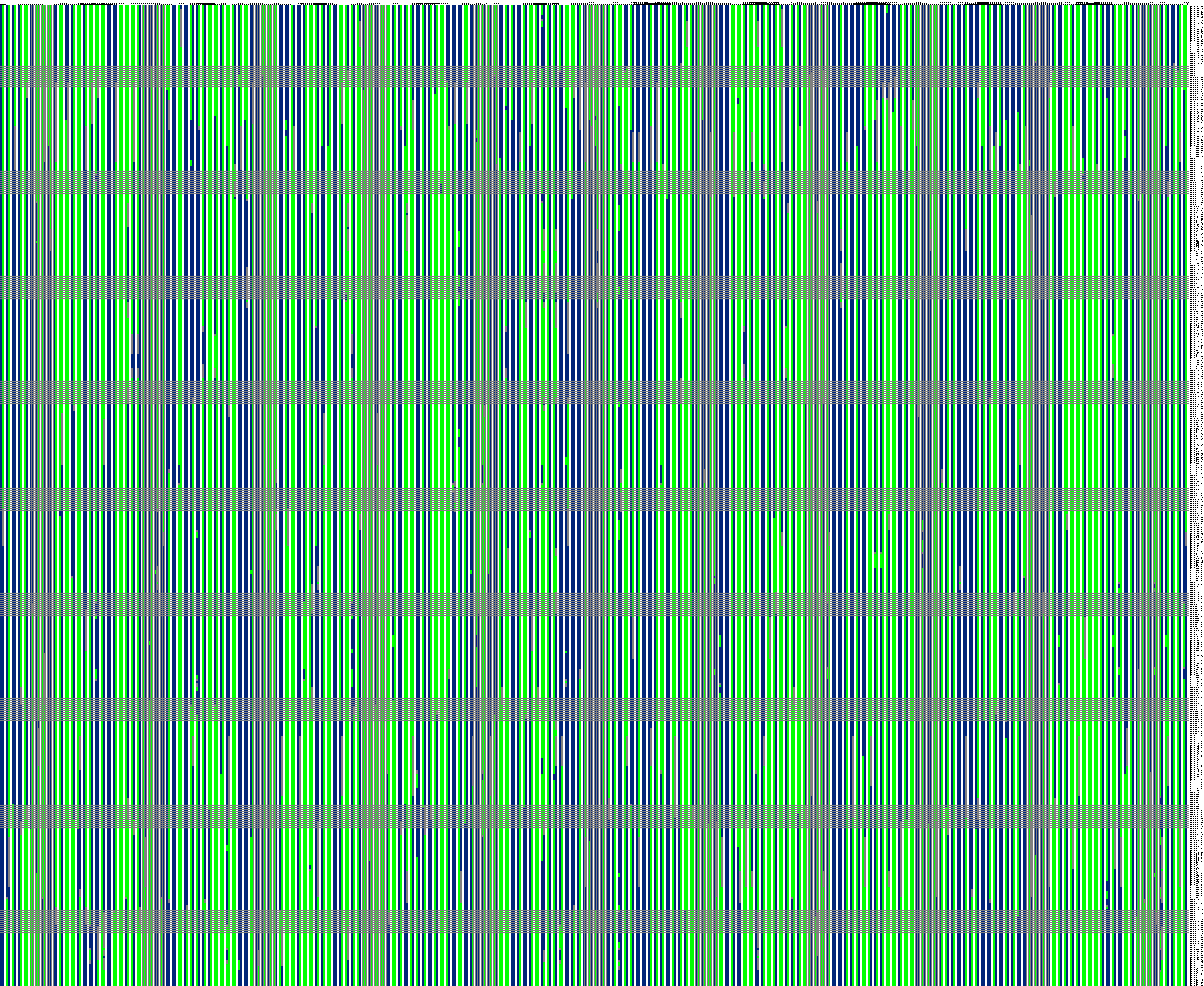

Supplement: Supplementary file 1 [file DataSheet_1.zip › Figure S5/male/LG11.male.haplo.png]

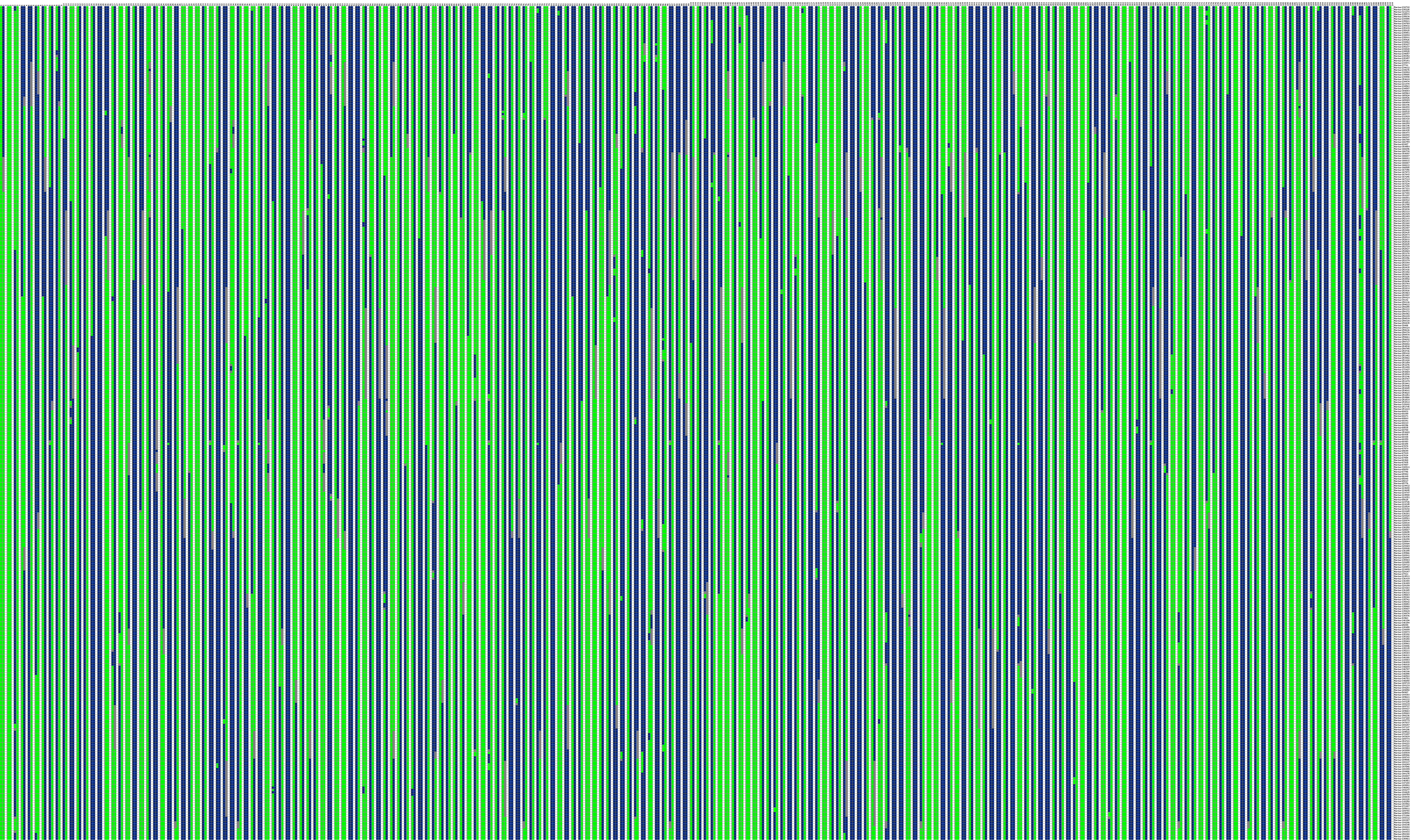

Supplement: Supplementary file 1 [file DataSheet_1.zip › Figure S5/male/LG12.male.haplo.png]

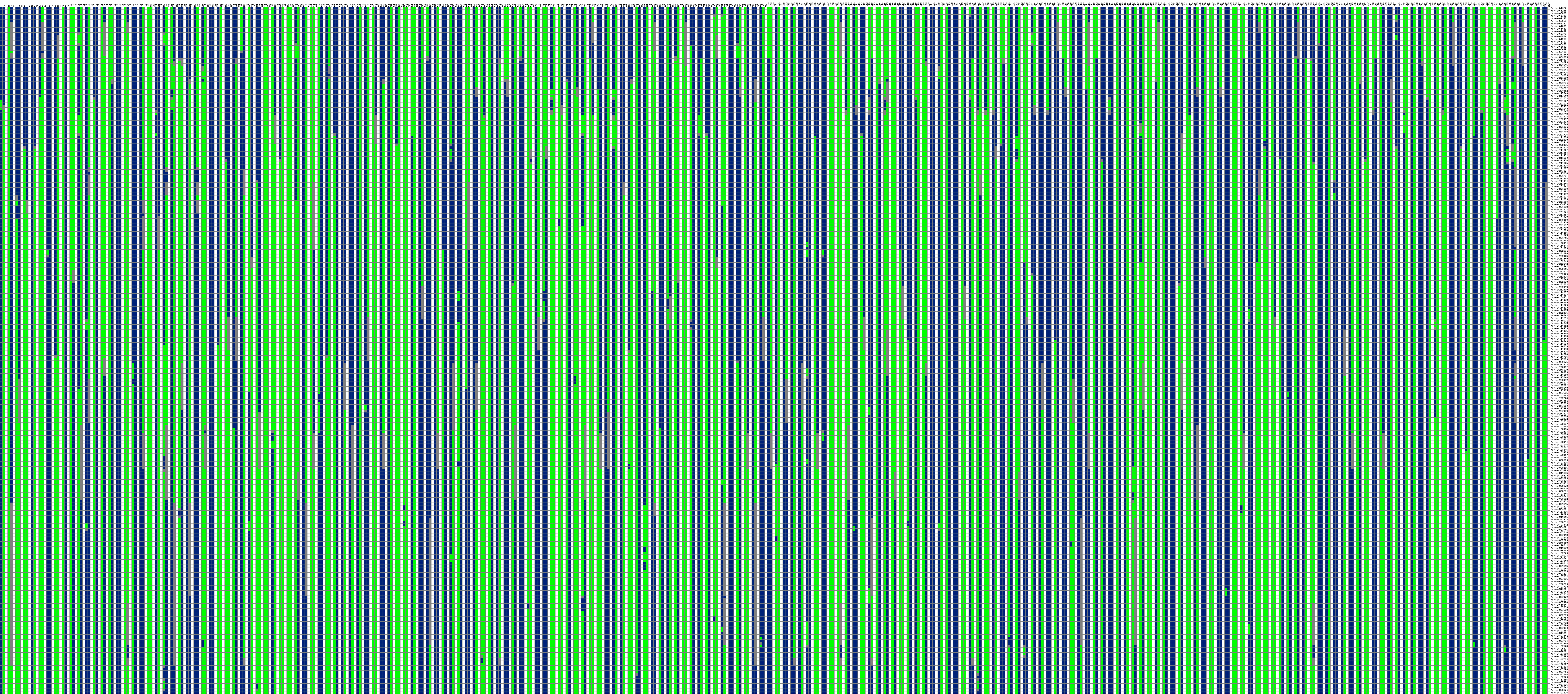

Supplement: Supplementary file 1 [file DataSheet_1.zip › Figure S5/male/LG13.male.haplo.png]

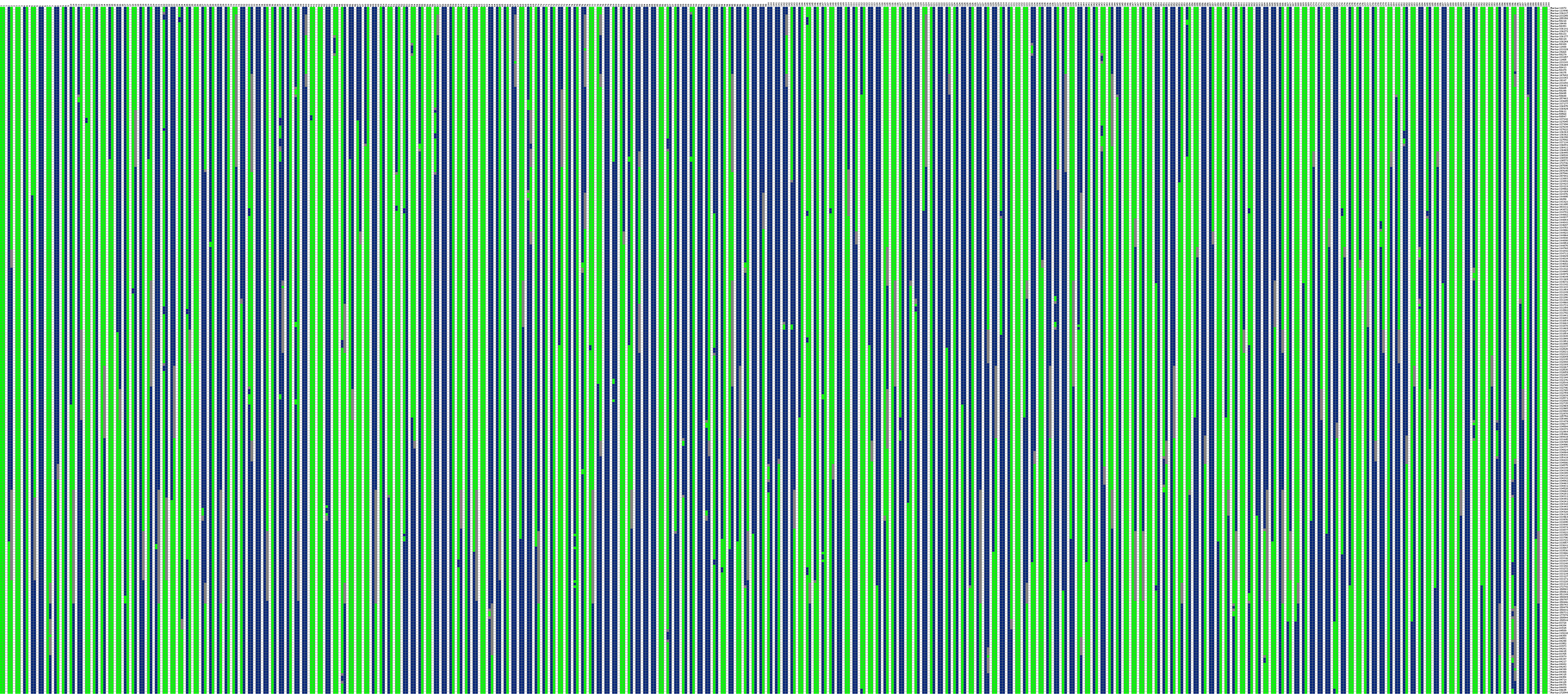

Supplement: Supplementary file 1 [file DataSheet_1.zip › Figure S5/male/LG14.male.haplo.png]

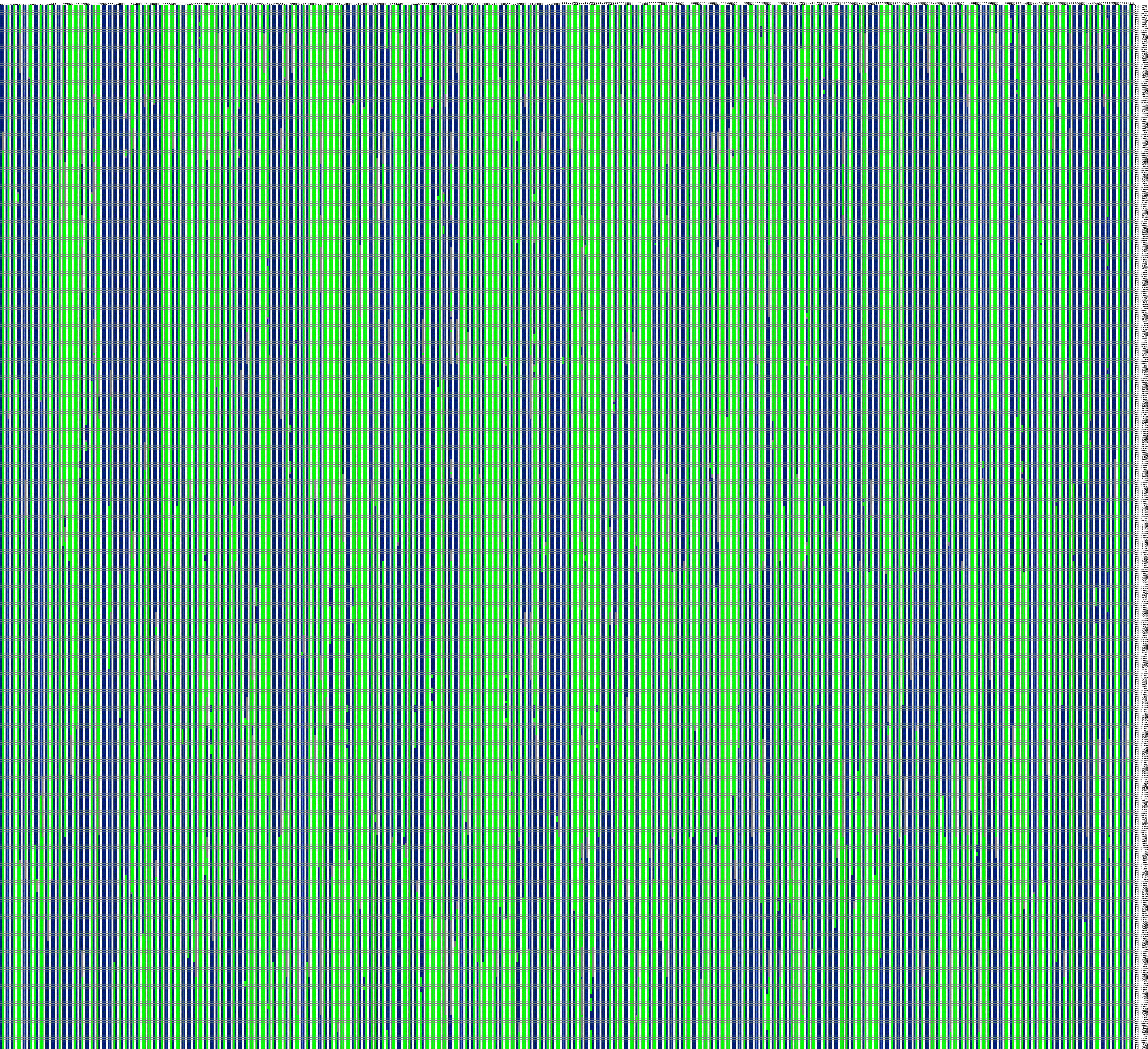

Supplement: Supplementary file 1 [file DataSheet_1.zip › Figure S5/male/LG15.male.haplo.png]

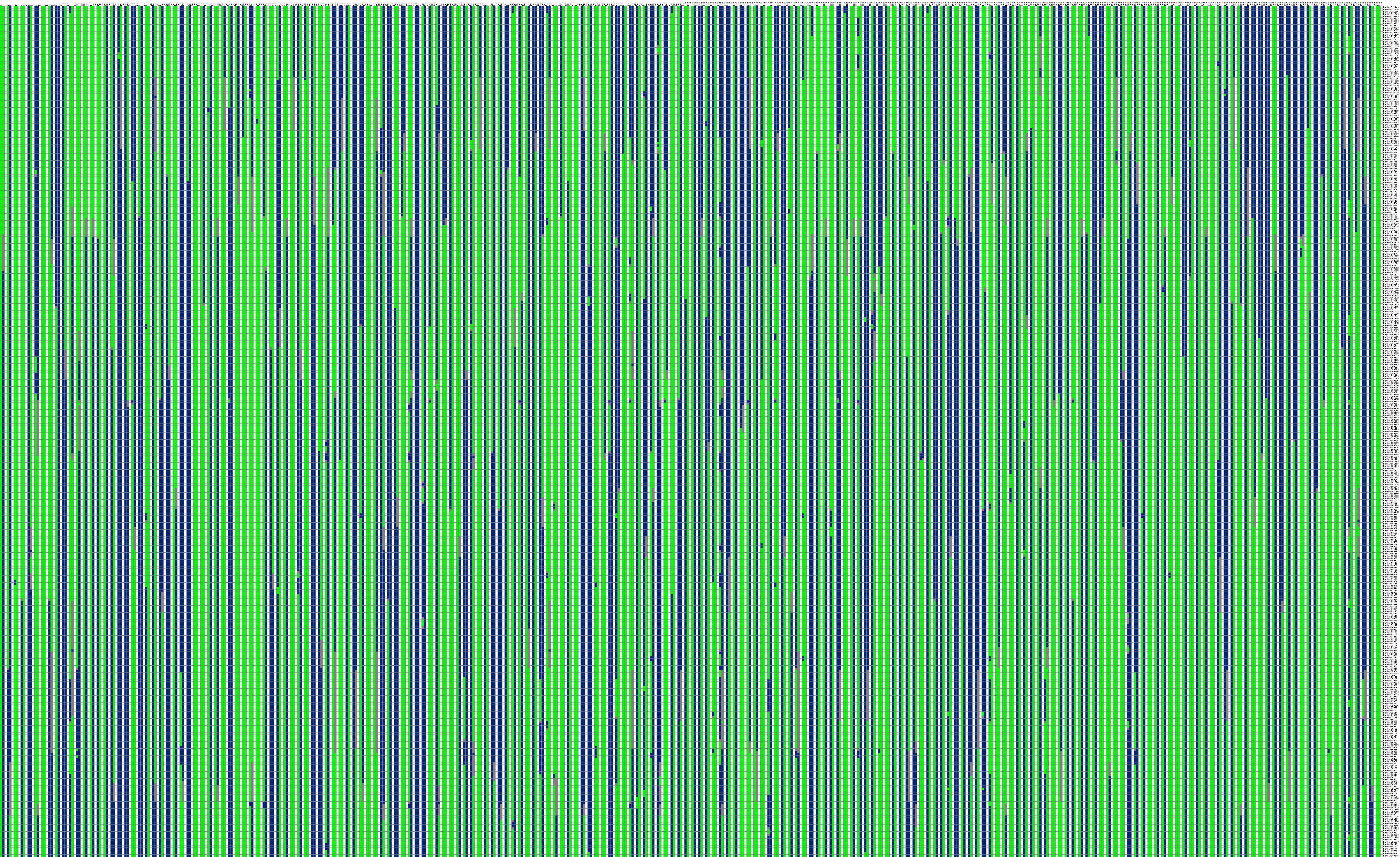

Supplement: Supplementary file 1 [file DataSheet_1.zip › Figure S5/male/LG16.male.haplo.png]

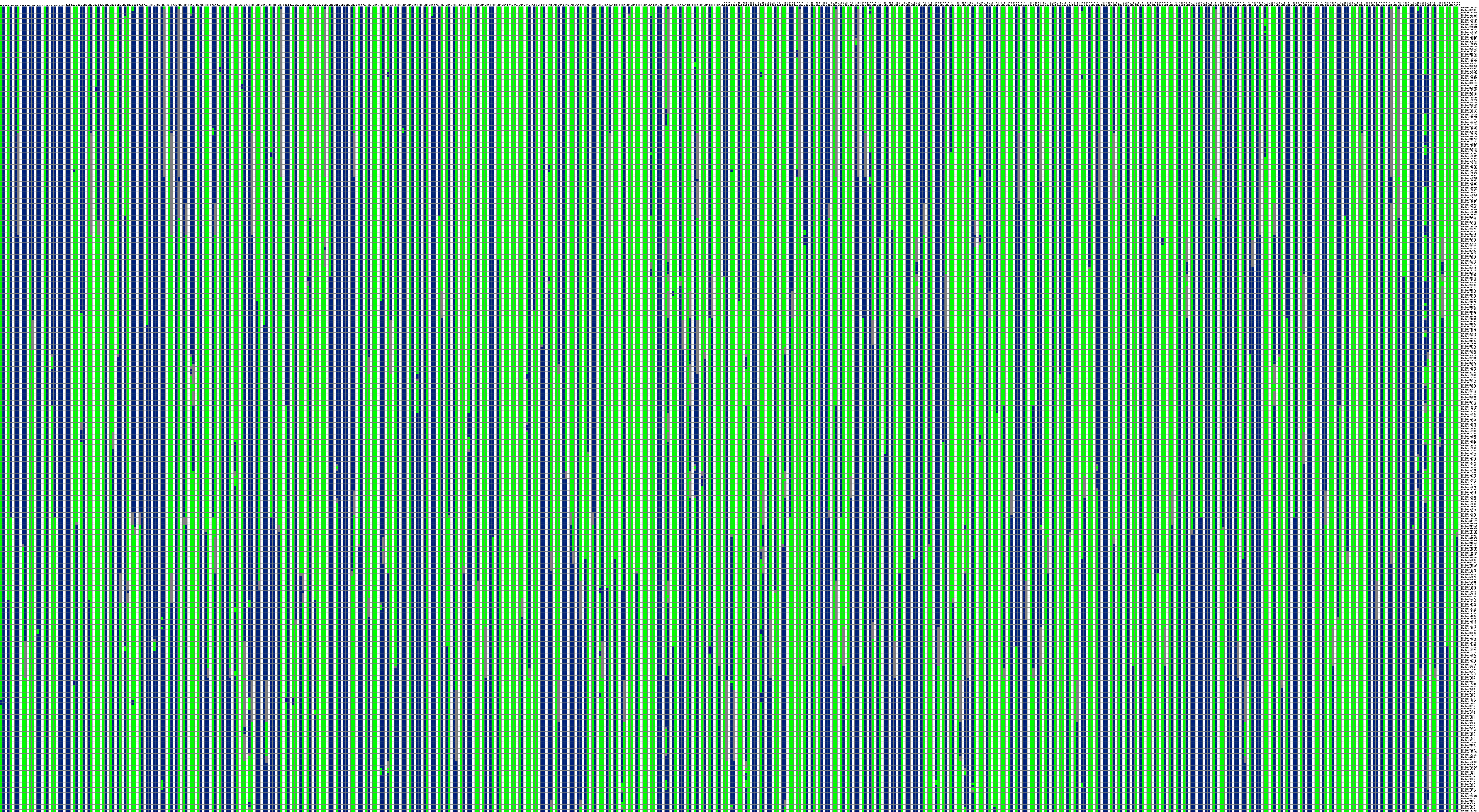

Supplement: Supplementary file 1 [file DataSheet_1.zip › Figure S5/male/LG17.male.haplo.png]

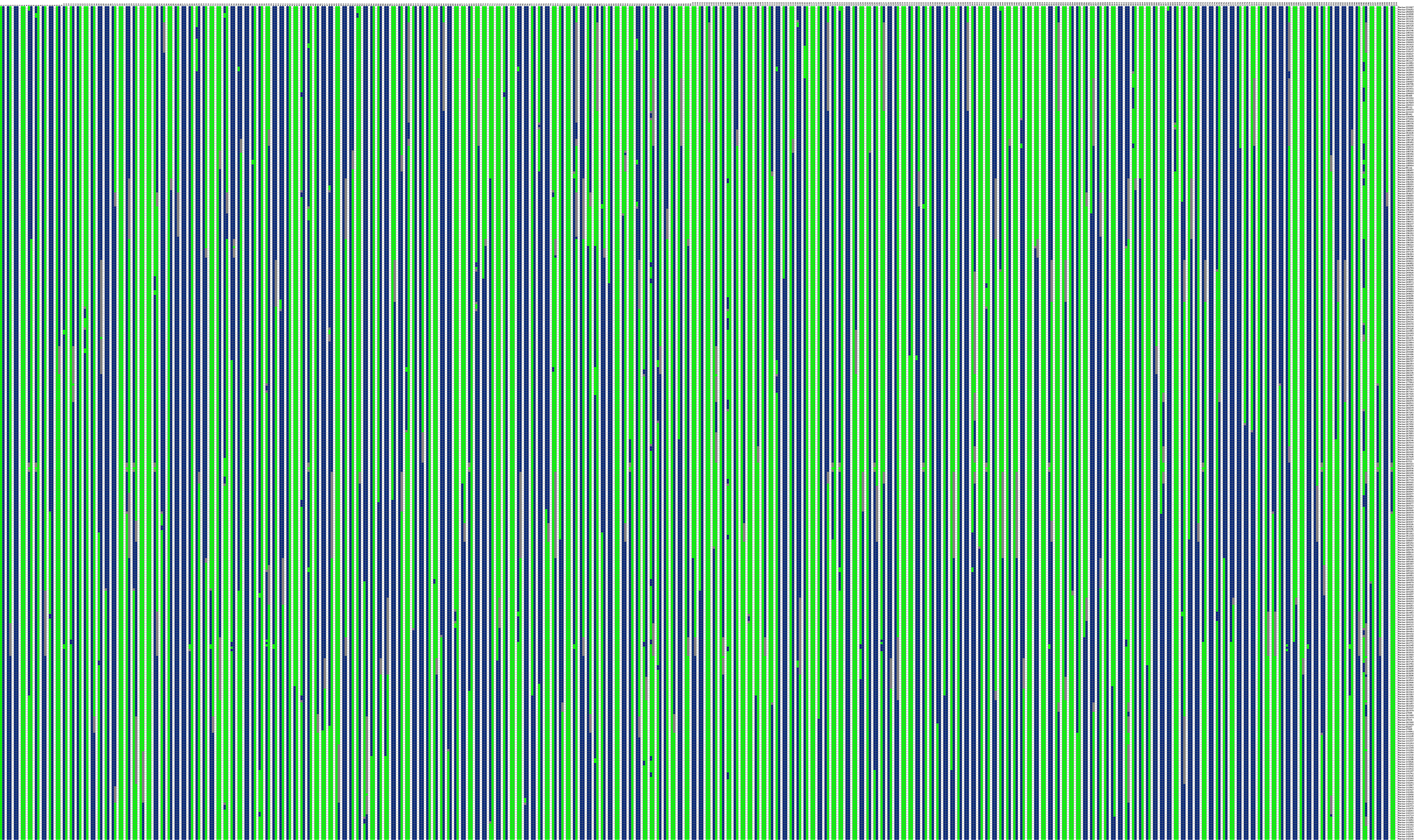

Supplement: Supplementary file 1 [file DataSheet_1.zip › Figure S5/male/LG18.male.haplo.png]

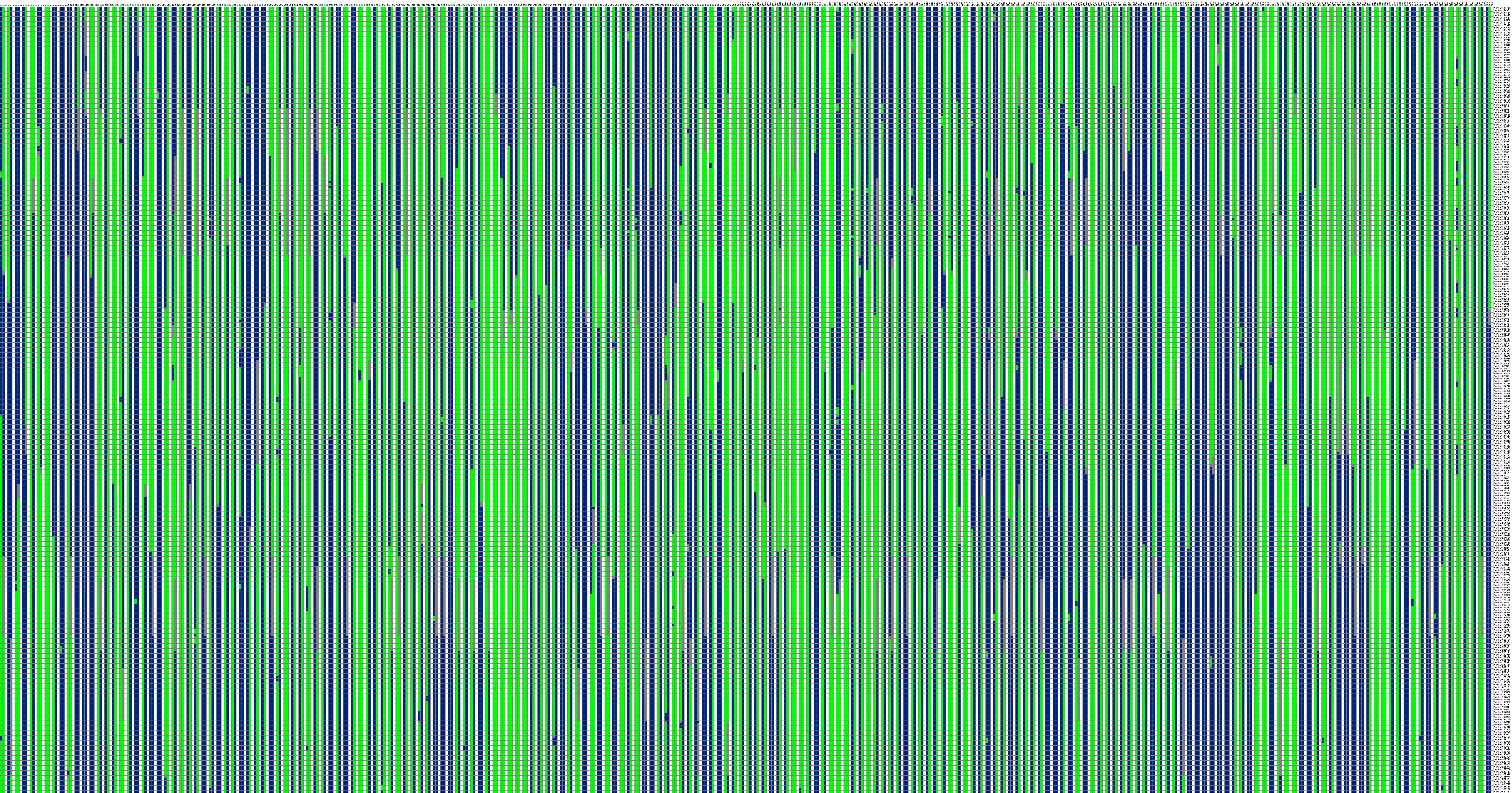

Supplement: Supplementary file 1 [file DataSheet_1.zip › Figure S5/male/LG19.male.haplo.png]

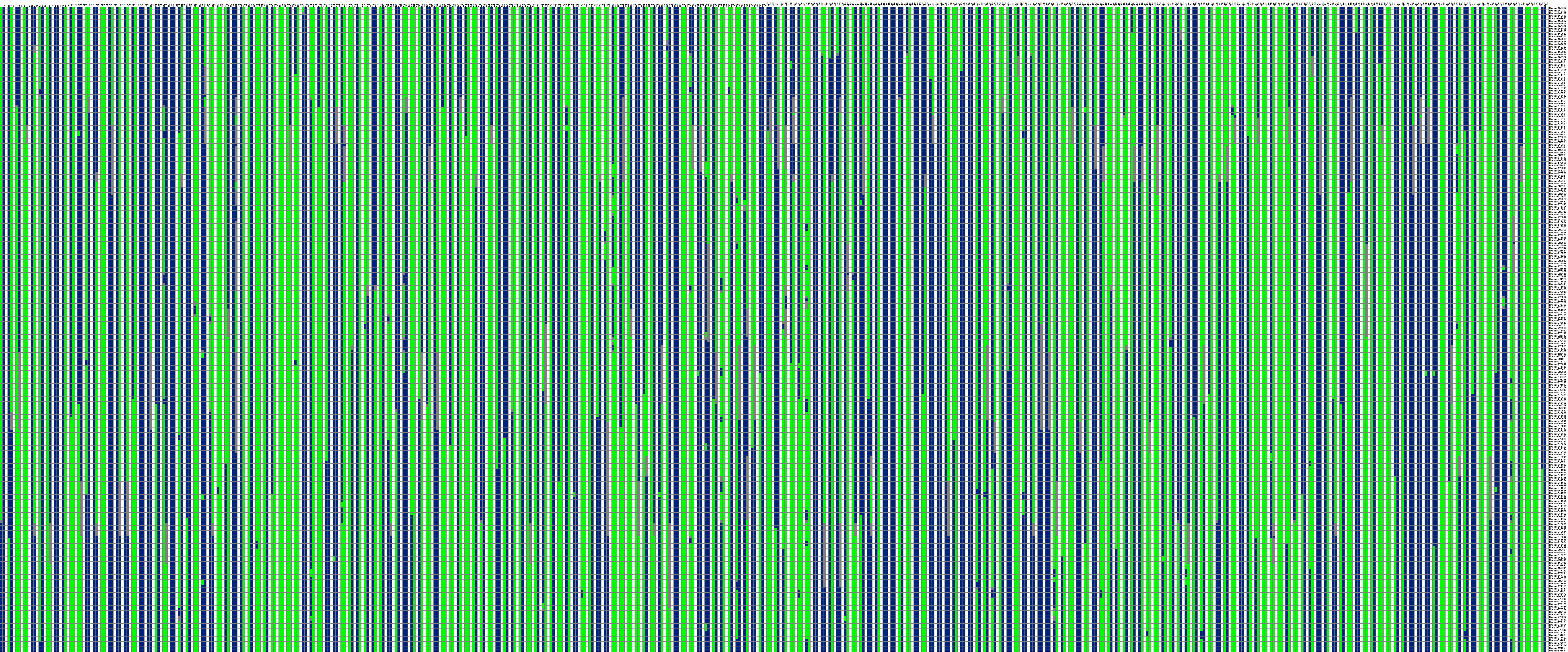

Supplement: Supplementary file 1 [file DataSheet_1.zip › Figure S5/male/LG2.male.haplo.png]

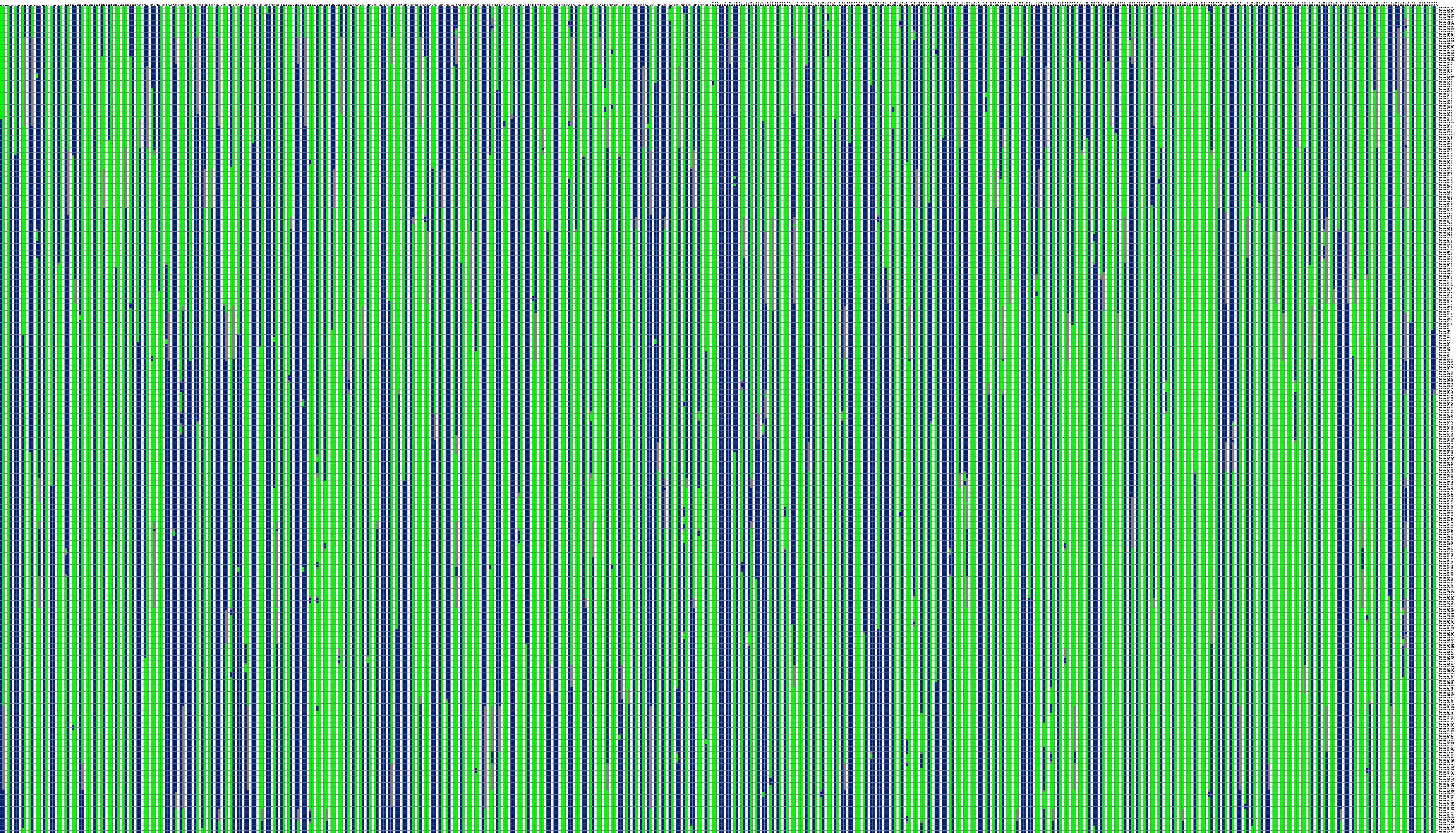

Supplement: Supplementary file 1 [file DataSheet_1.zip › Figure S5/male/LG20.male.haplo.png]

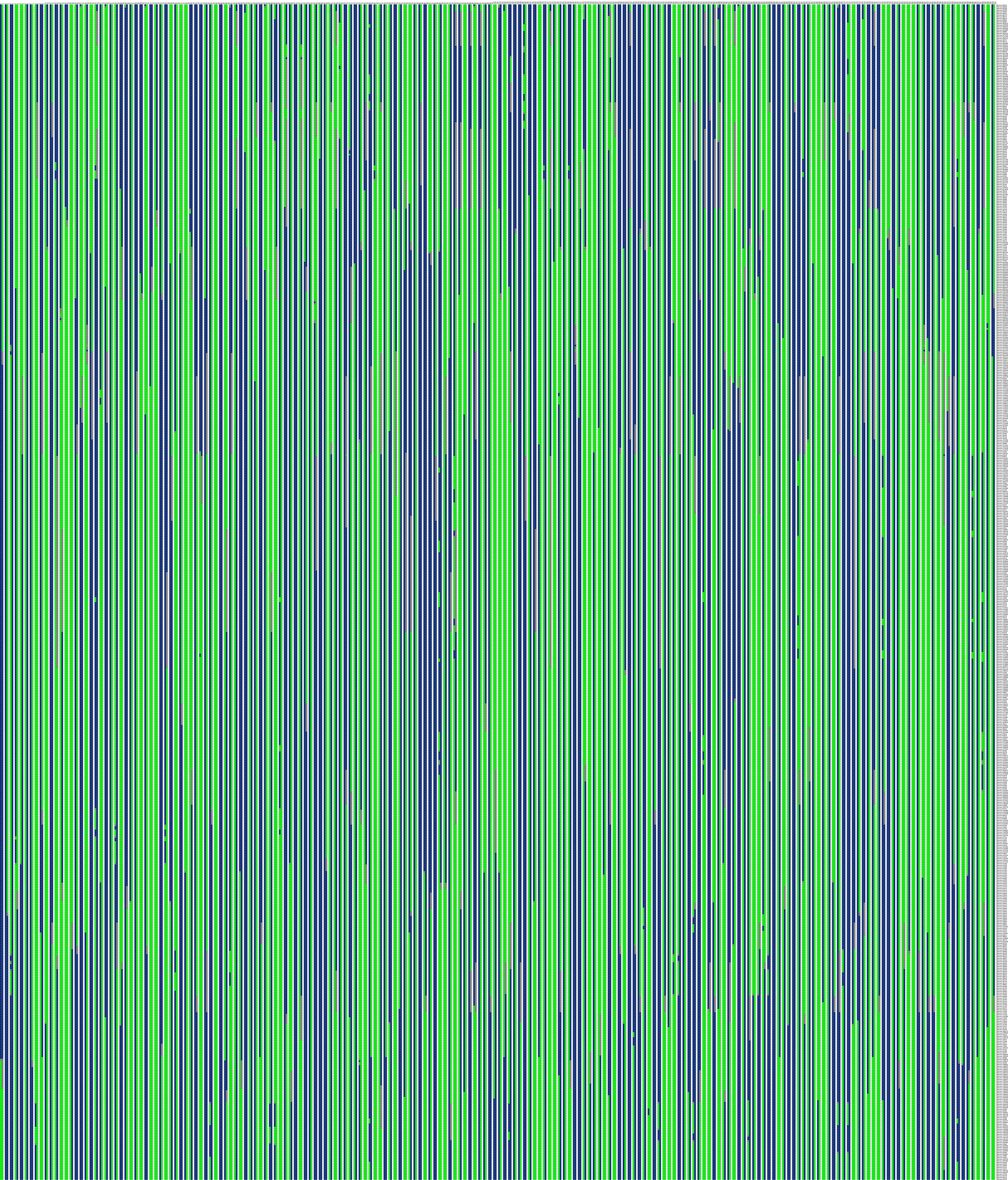

Supplement: Supplementary file 1 [file DataSheet_1.zip › Figure S5/male/LG21.male.haplo.png]

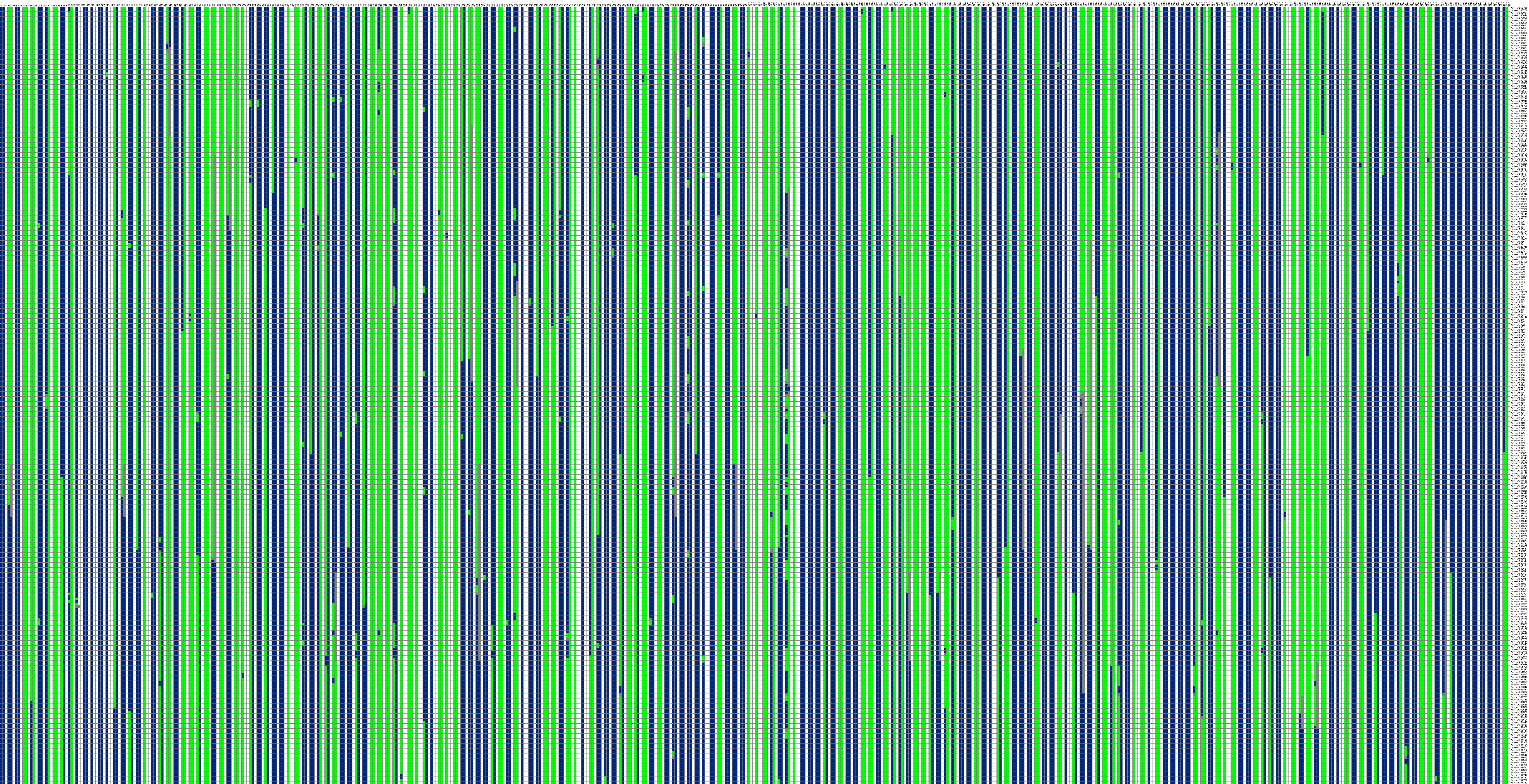

Supplement: Supplementary file 1 [file DataSheet_1.zip › Figure S5/male/LG22.male.haplo.png]

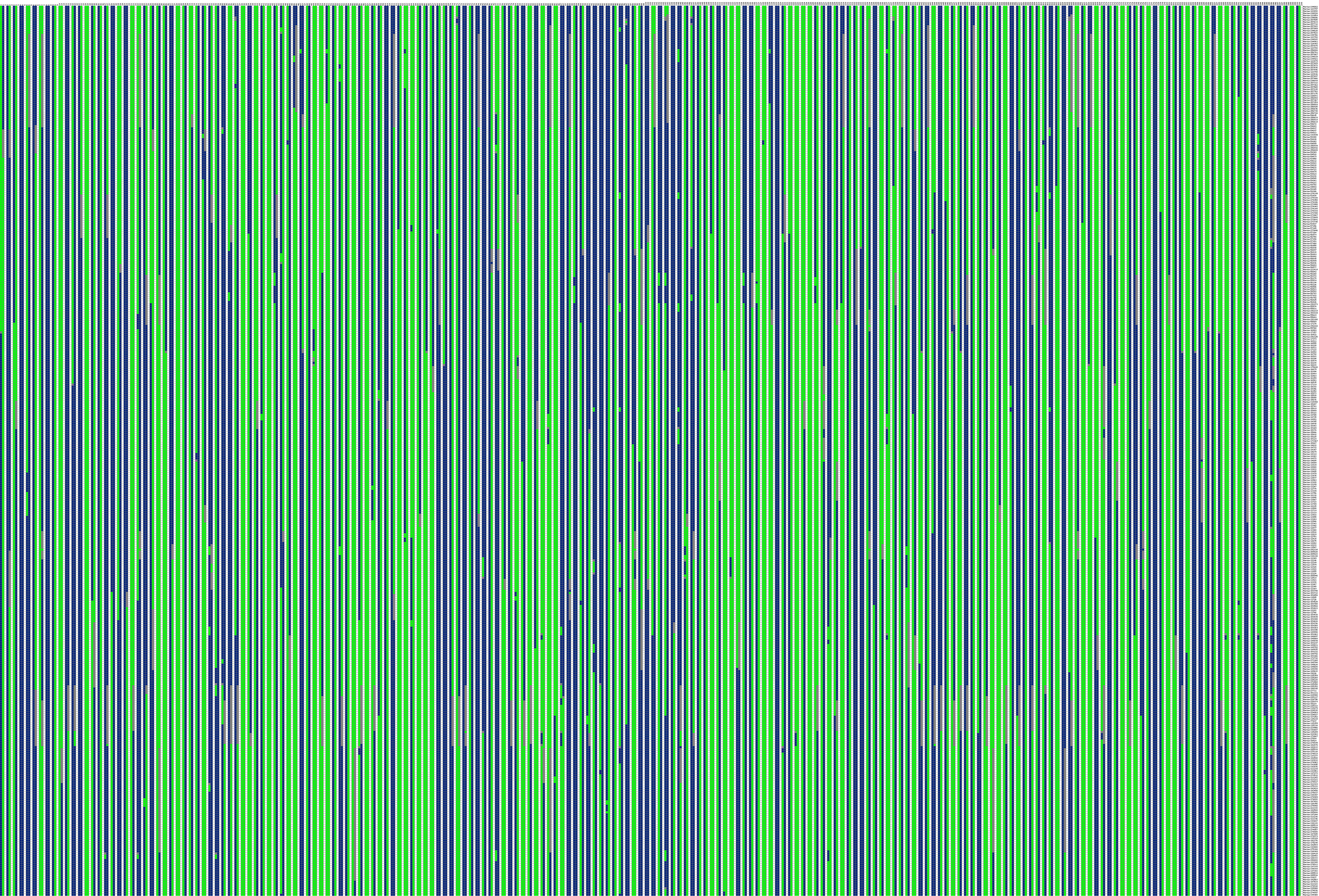

Supplement: Supplementary file 1 [file DataSheet_1.zip › Figure S5/male/LG23.male.haplo.png]

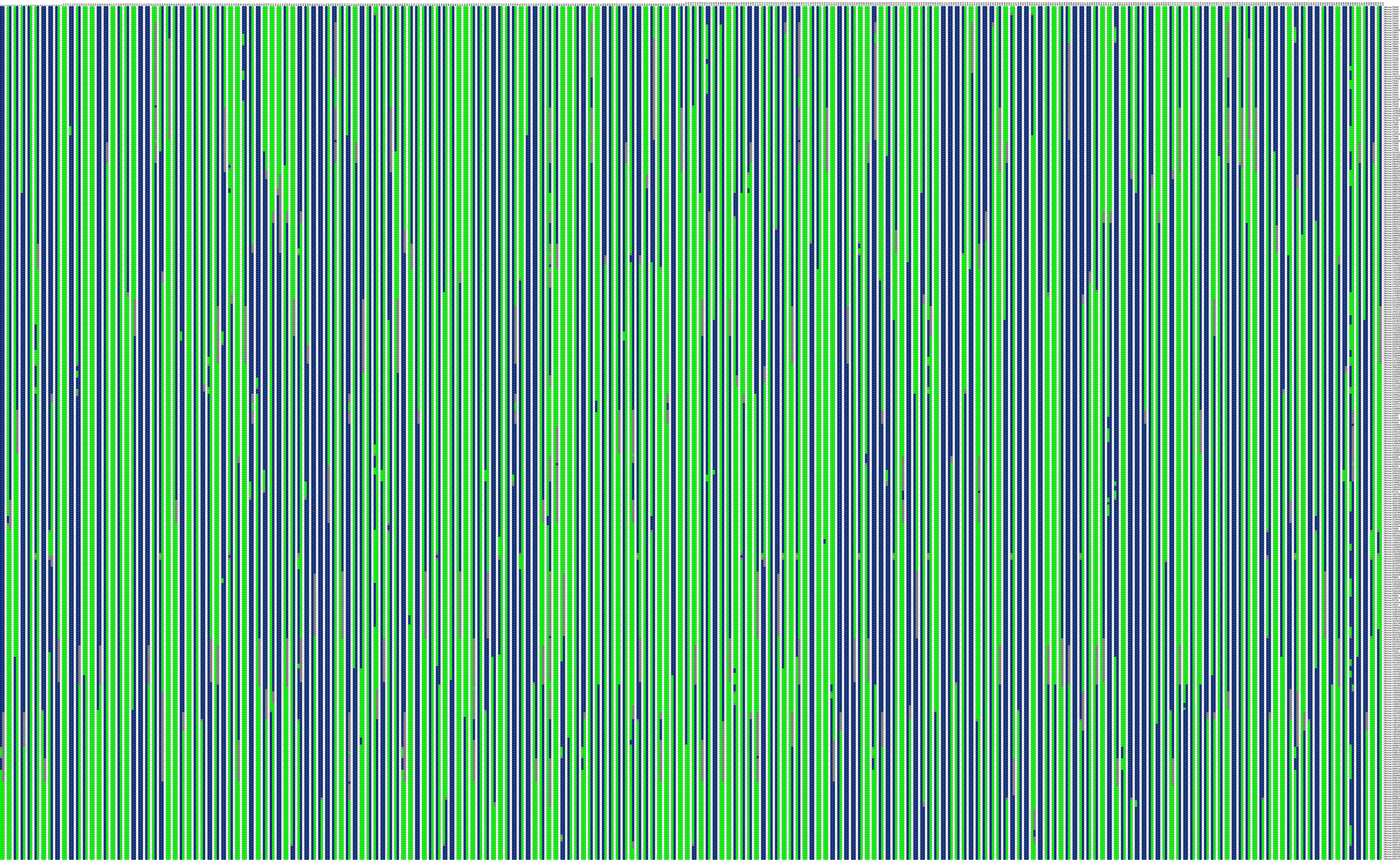

Supplement: Supplementary file 1 [file DataSheet_1.zip › Figure S5/male/LG24.male.haplo.png]

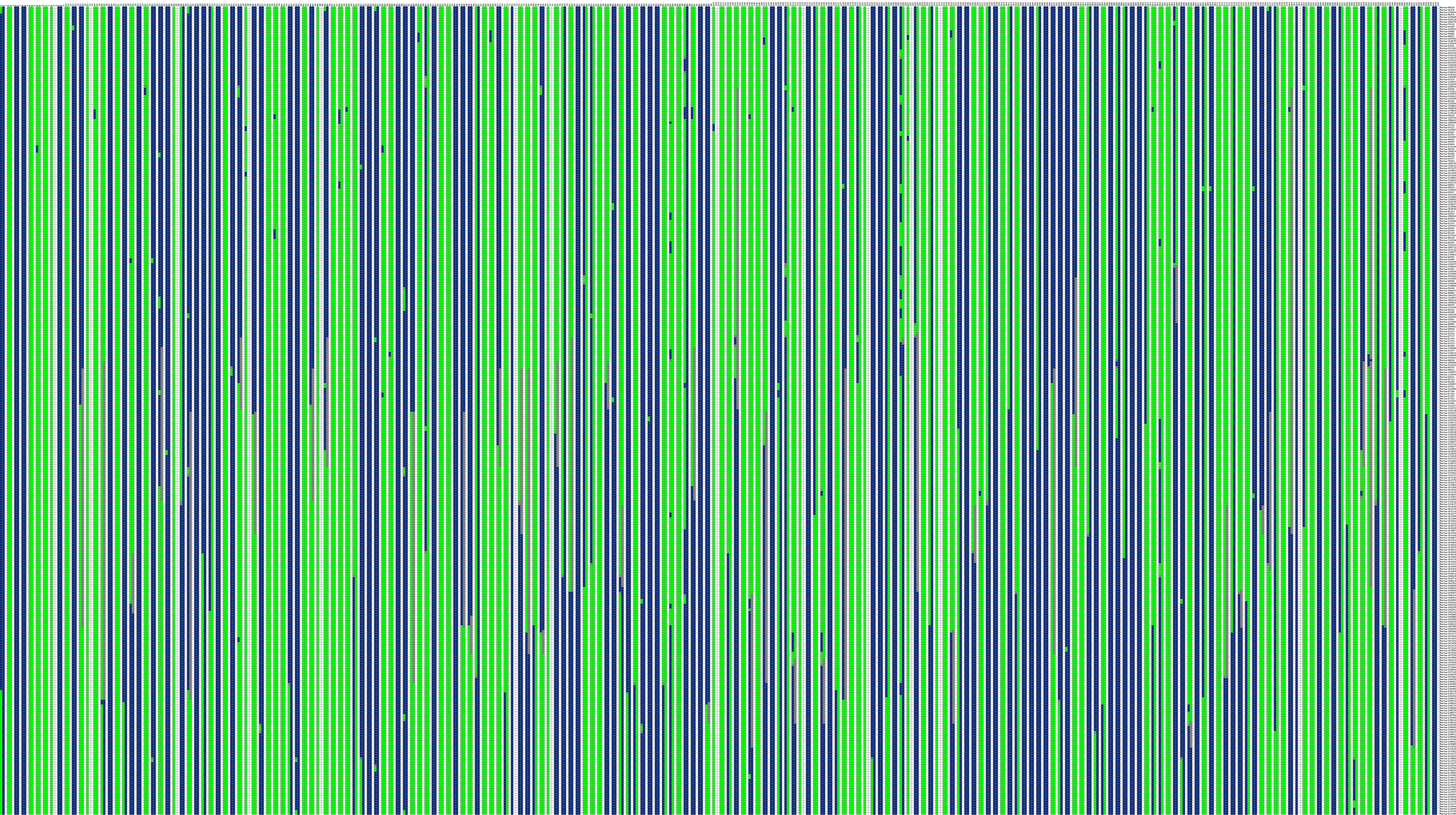

Supplement: Supplementary file 1 [file DataSheet_1.zip › Figure S5/male/LG3.male.haplo.png]

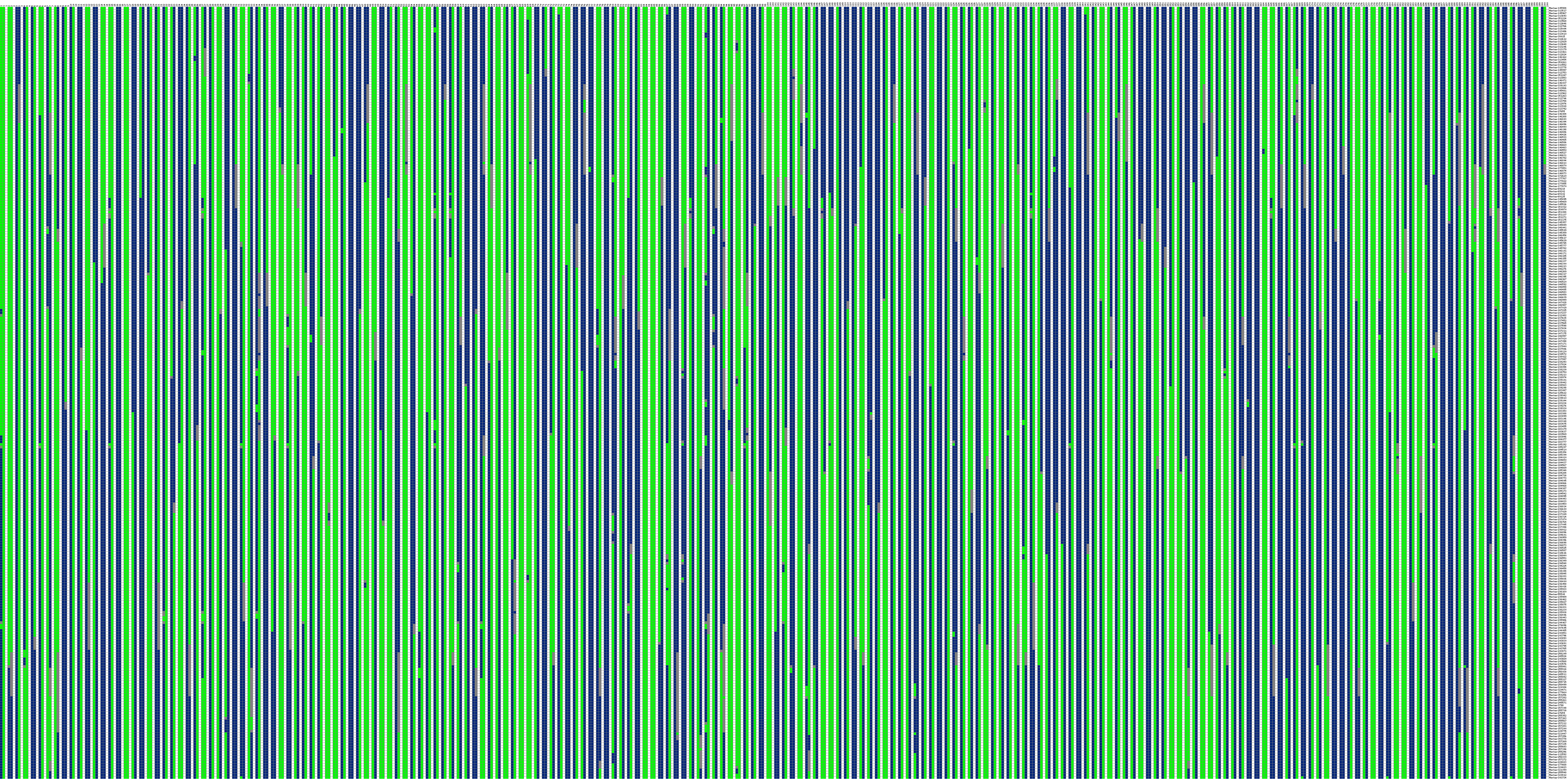

Supplement: Supplementary file 1 [file DataSheet_1.zip › Figure S5/male/LG4.male.haplo.png]

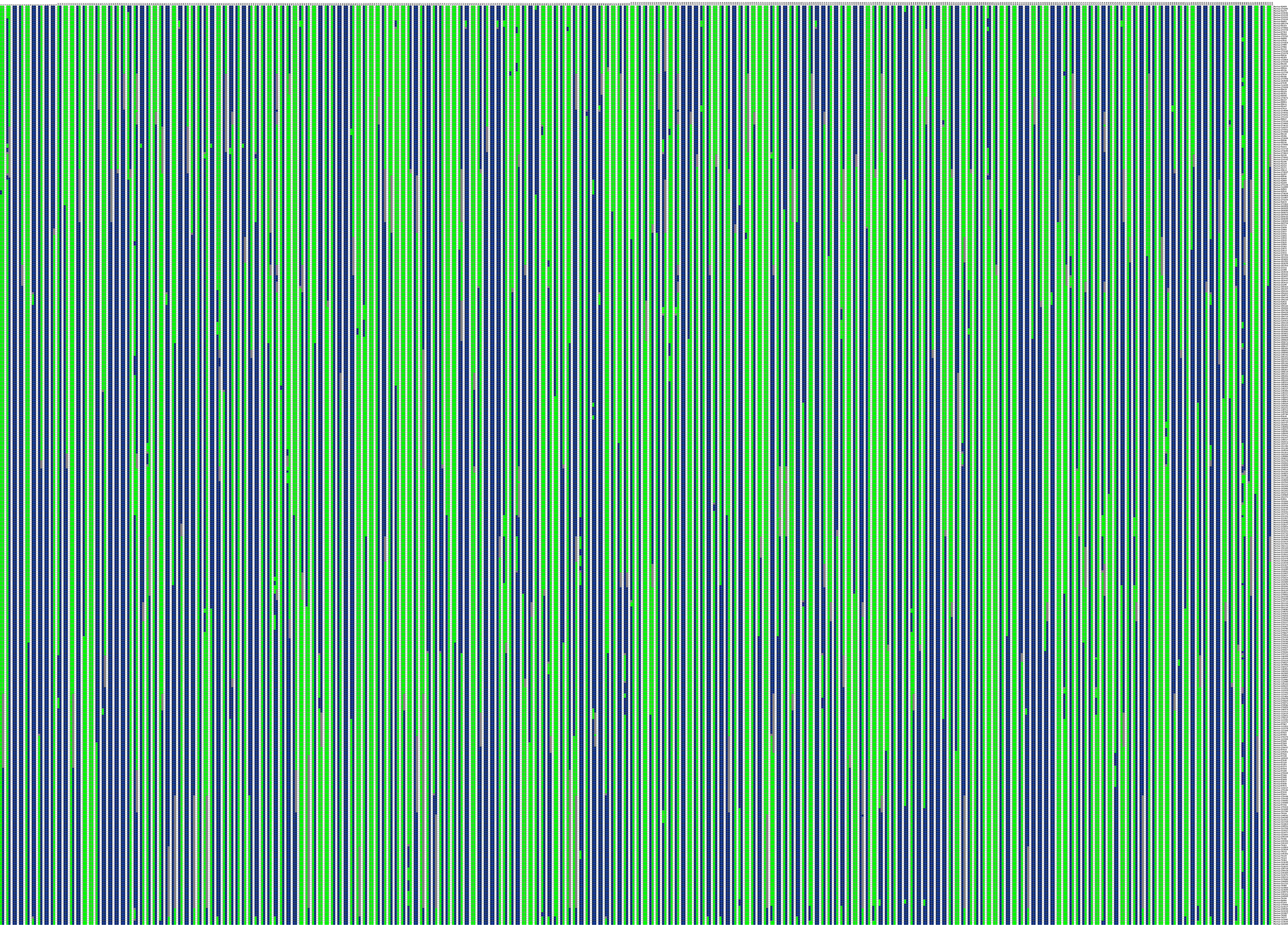

Supplement: Supplementary file 1 [file DataSheet_1.zip › Figure S5/male/LG5.male.haplo.png]

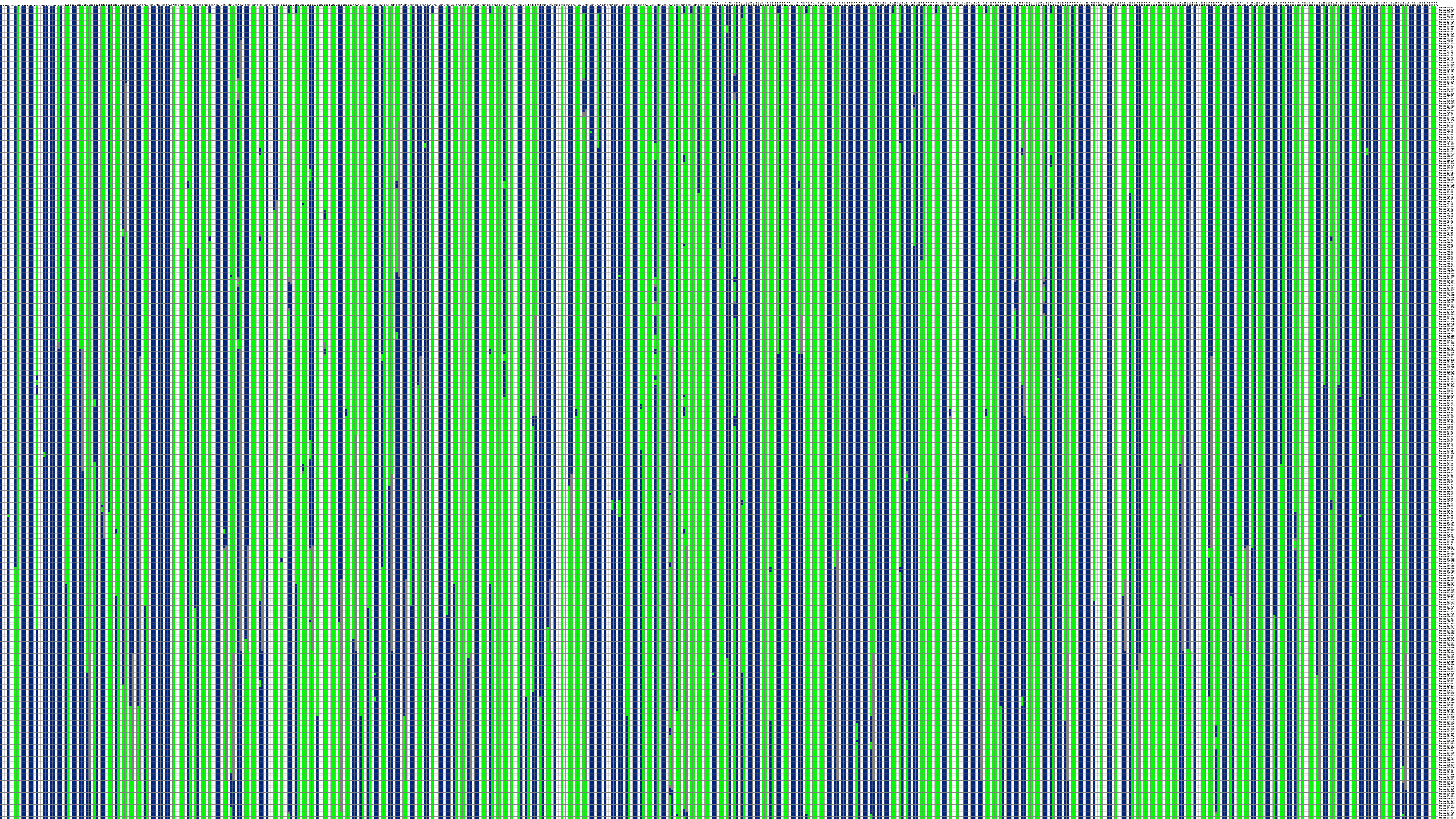

Supplement: Supplementary file 1 [file DataSheet_1.zip › Figure S5/male/LG6.male.haplo.png]

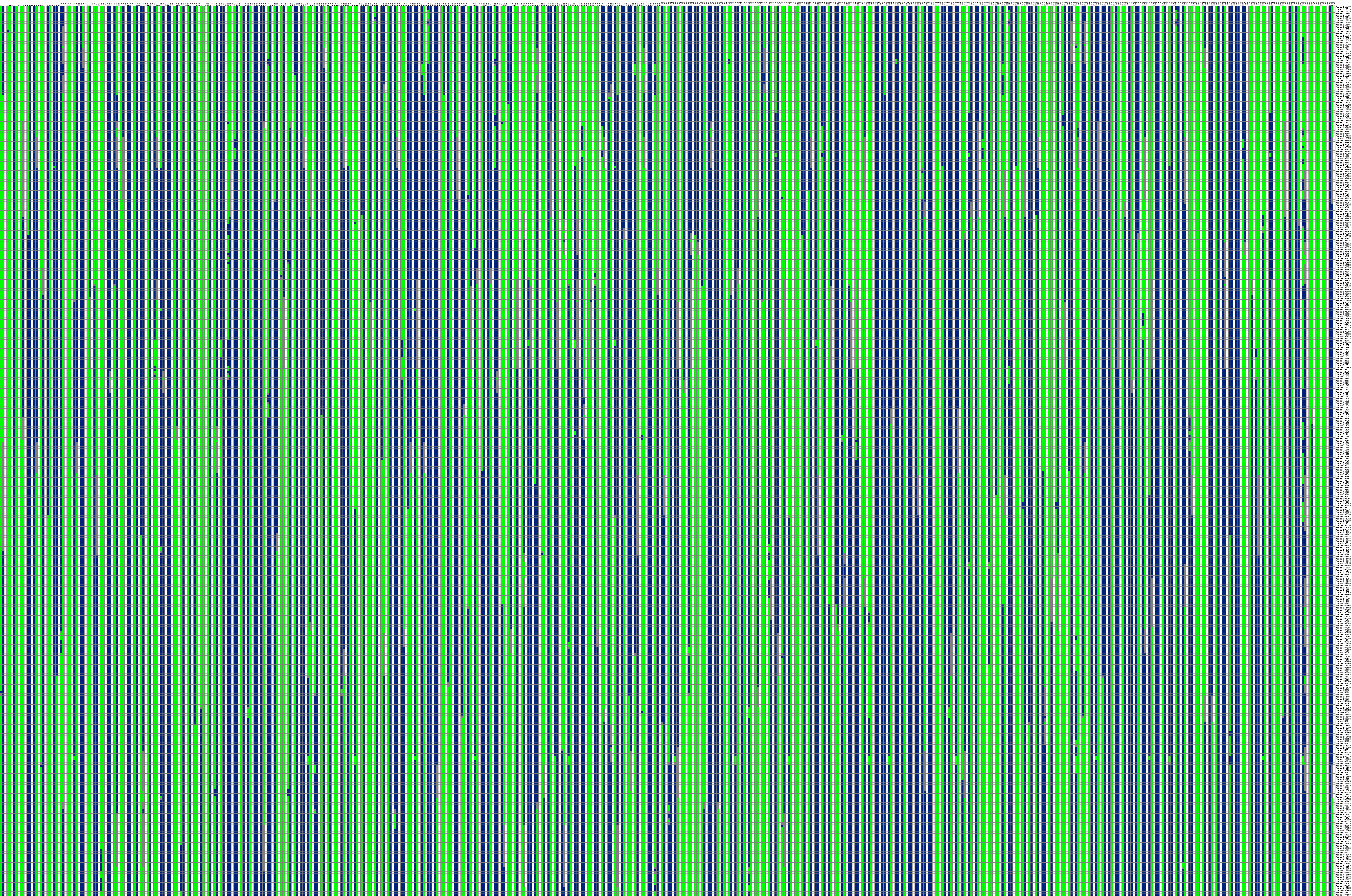

Supplement: Supplementary file 1 [file DataSheet_1.zip › Figure S5/male/LG7.male.haplo.png]

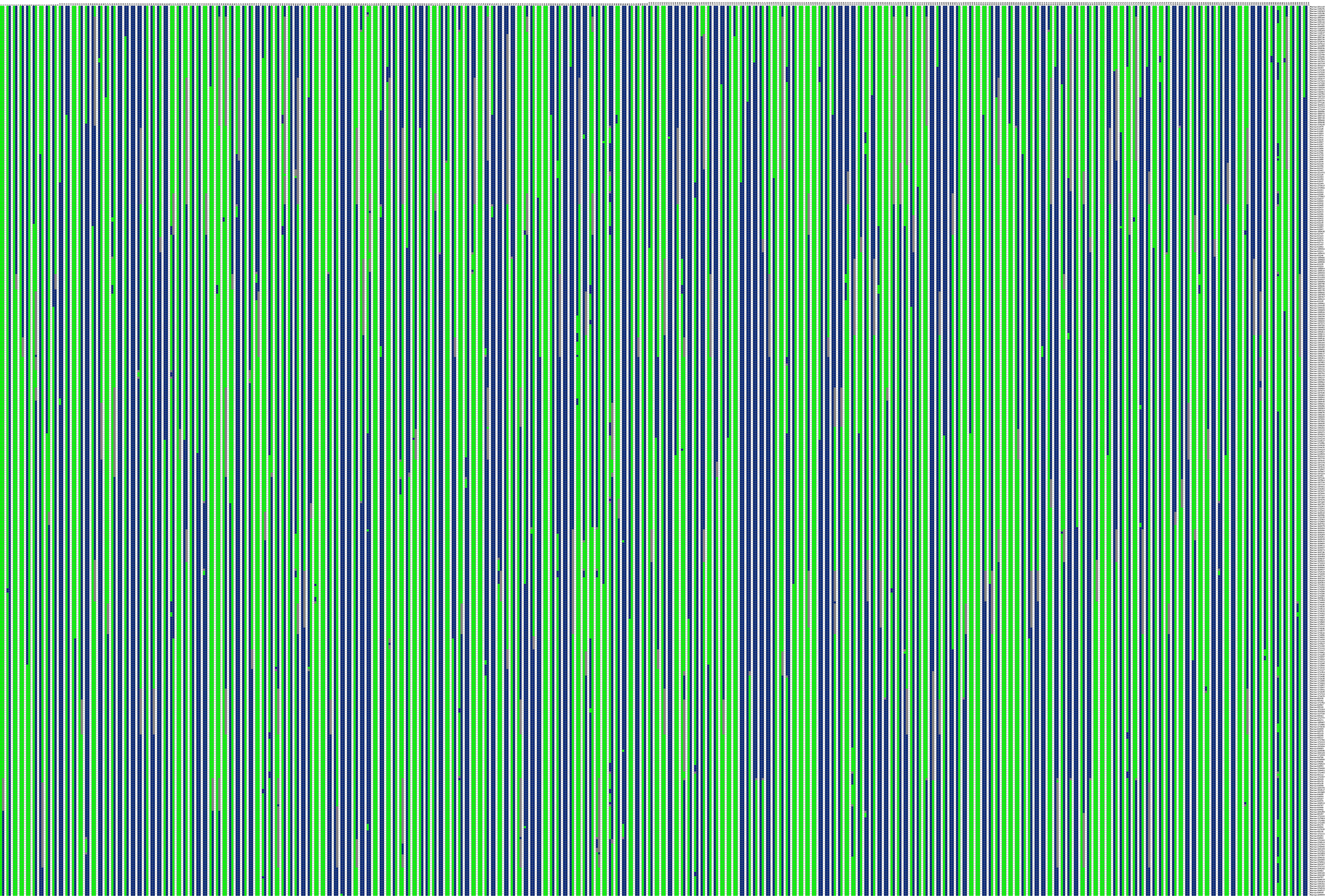

Supplement: Supplementary file 1 [file DataSheet_1.zip › Figure S5/male/LG8.male.haplo.png]

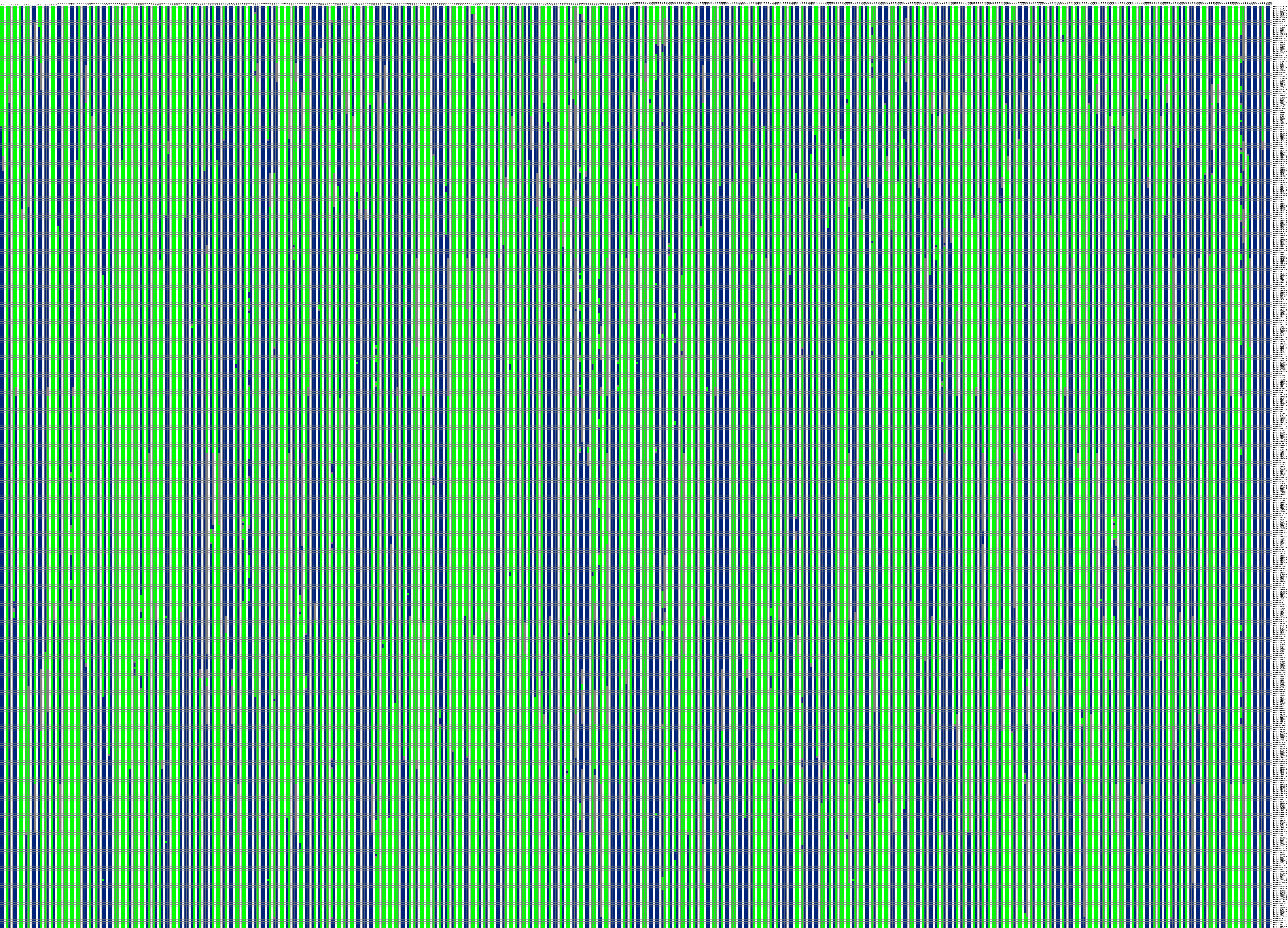

Supplement: Supplementary file 1 [file DataSheet_1.zip › Figure S5/male/LG9.male.haplo.png]

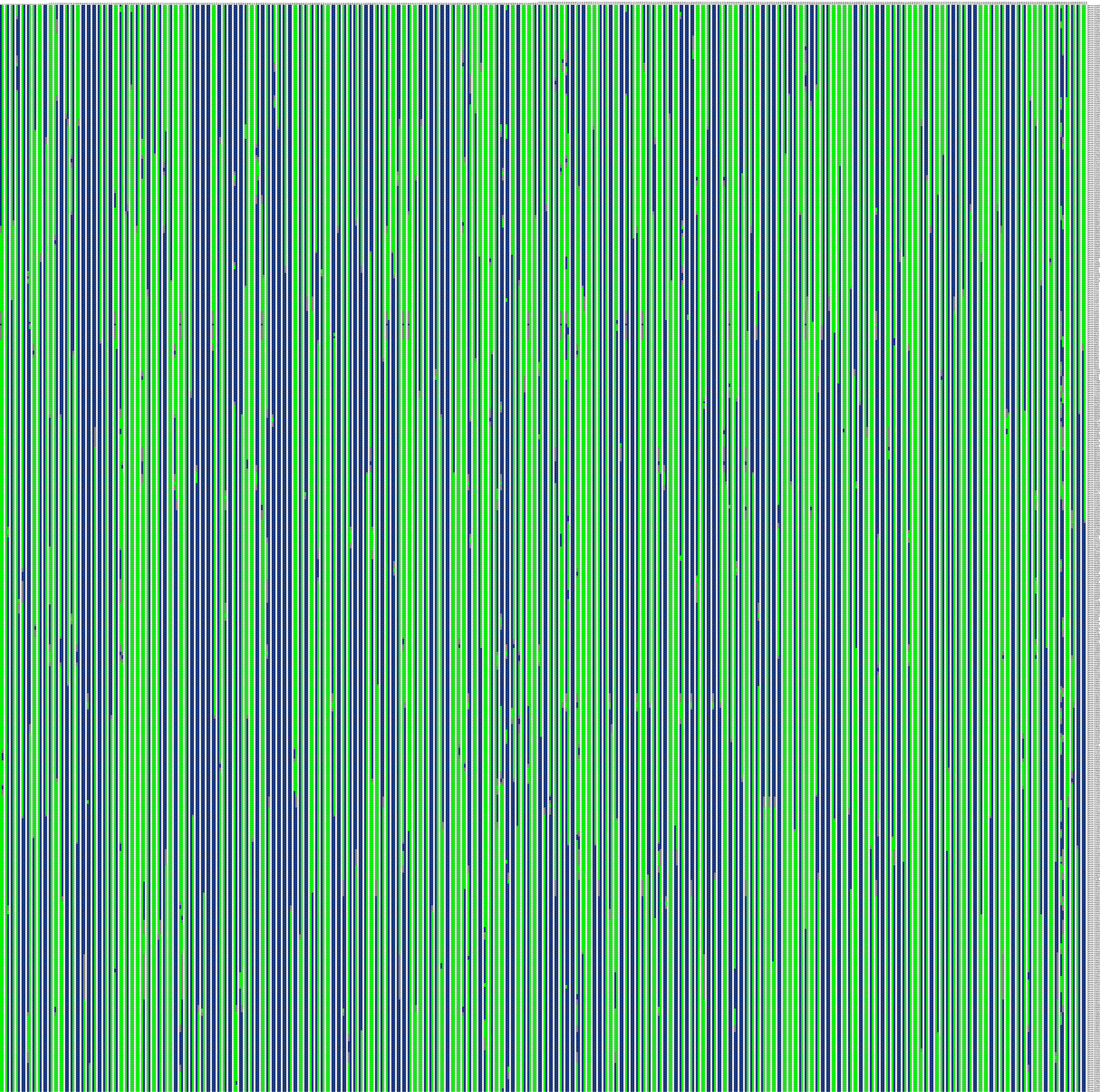

Supplement: Supplementary file 1 [file DataSheet_1.zip › Figure S5/sexAver/LG1.sexAver.haplo.png]

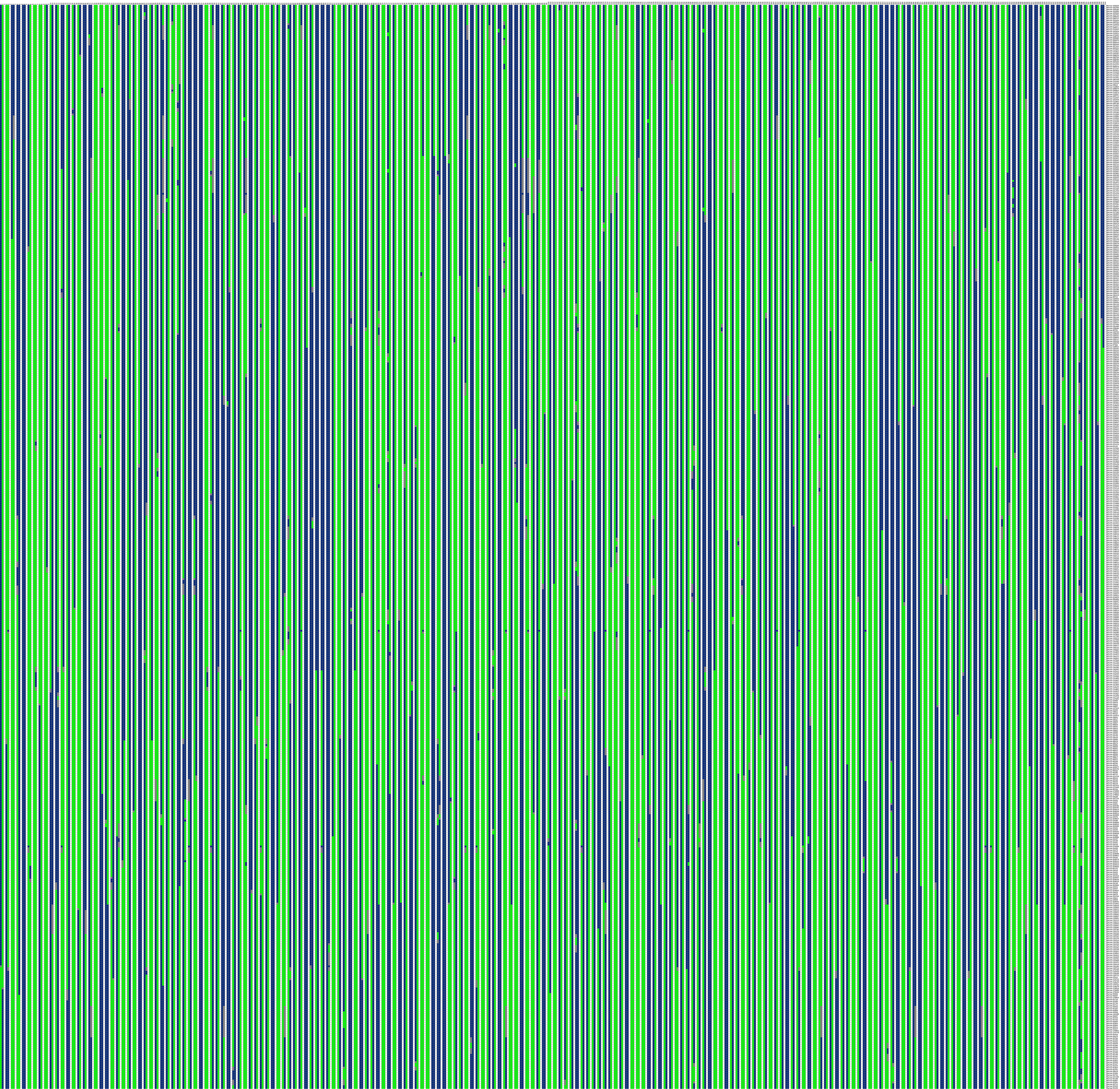

Supplement: Supplementary file 1 [file DataSheet_1.zip › Figure S5/sexAver/LG10.sexAver.haplo.png]

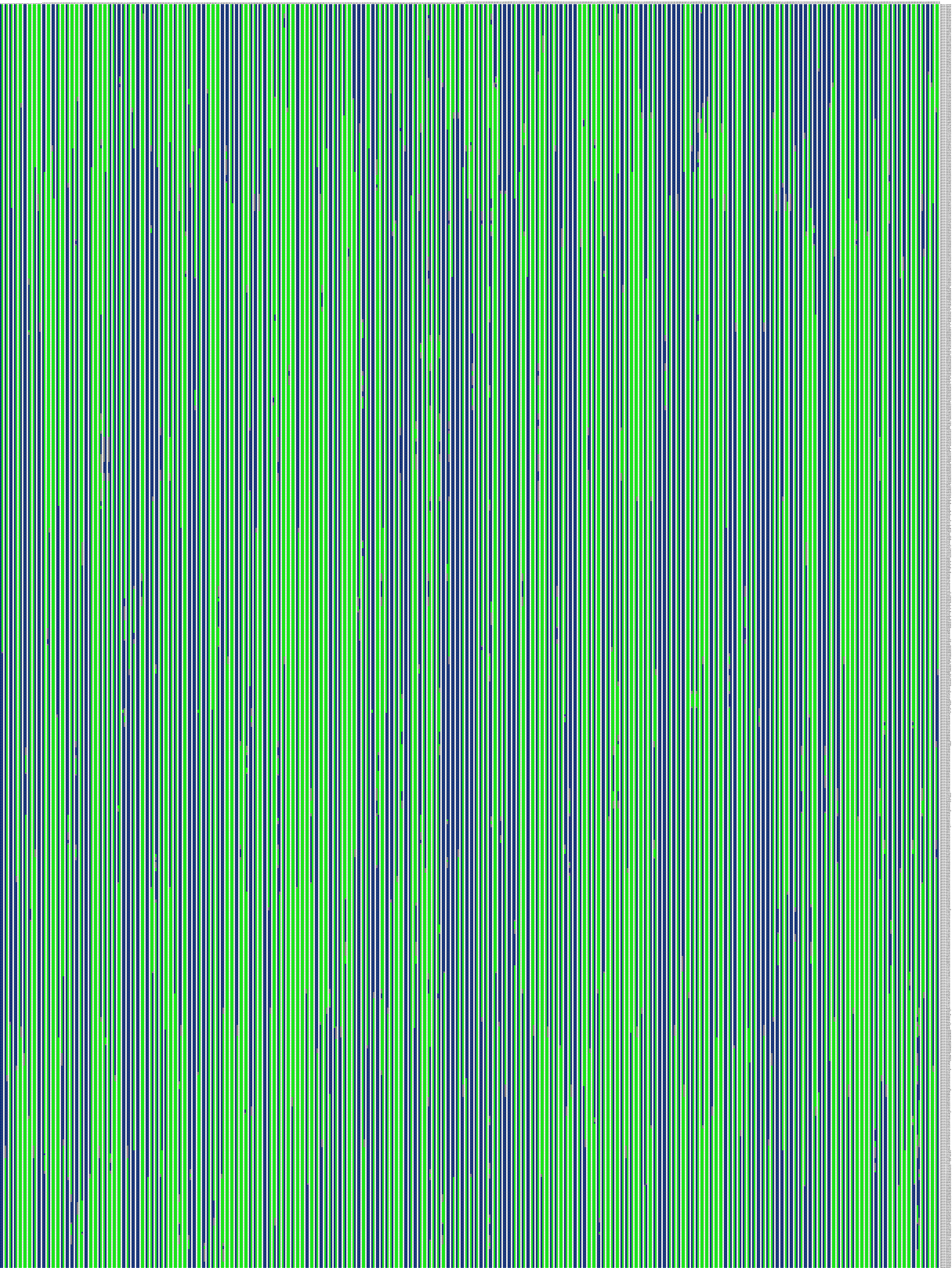

Supplement: Supplementary file 1 [file DataSheet_1.zip › Figure S5/sexAver/LG11.sexAver.haplo.png]

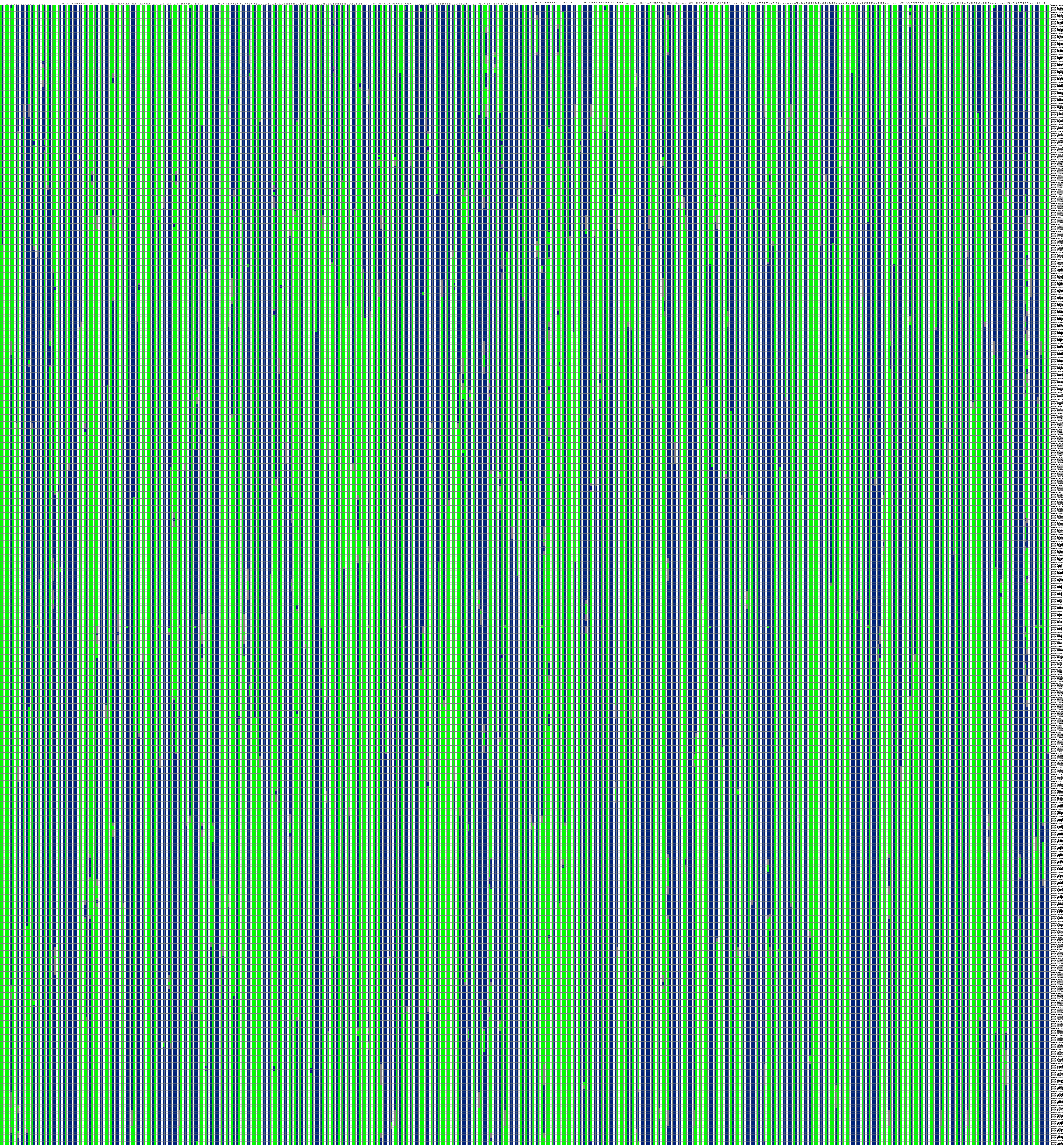

Supplement: Supplementary file 1 [file DataSheet_1.zip › Figure S5/sexAver/LG12.sexAver.haplo.png]

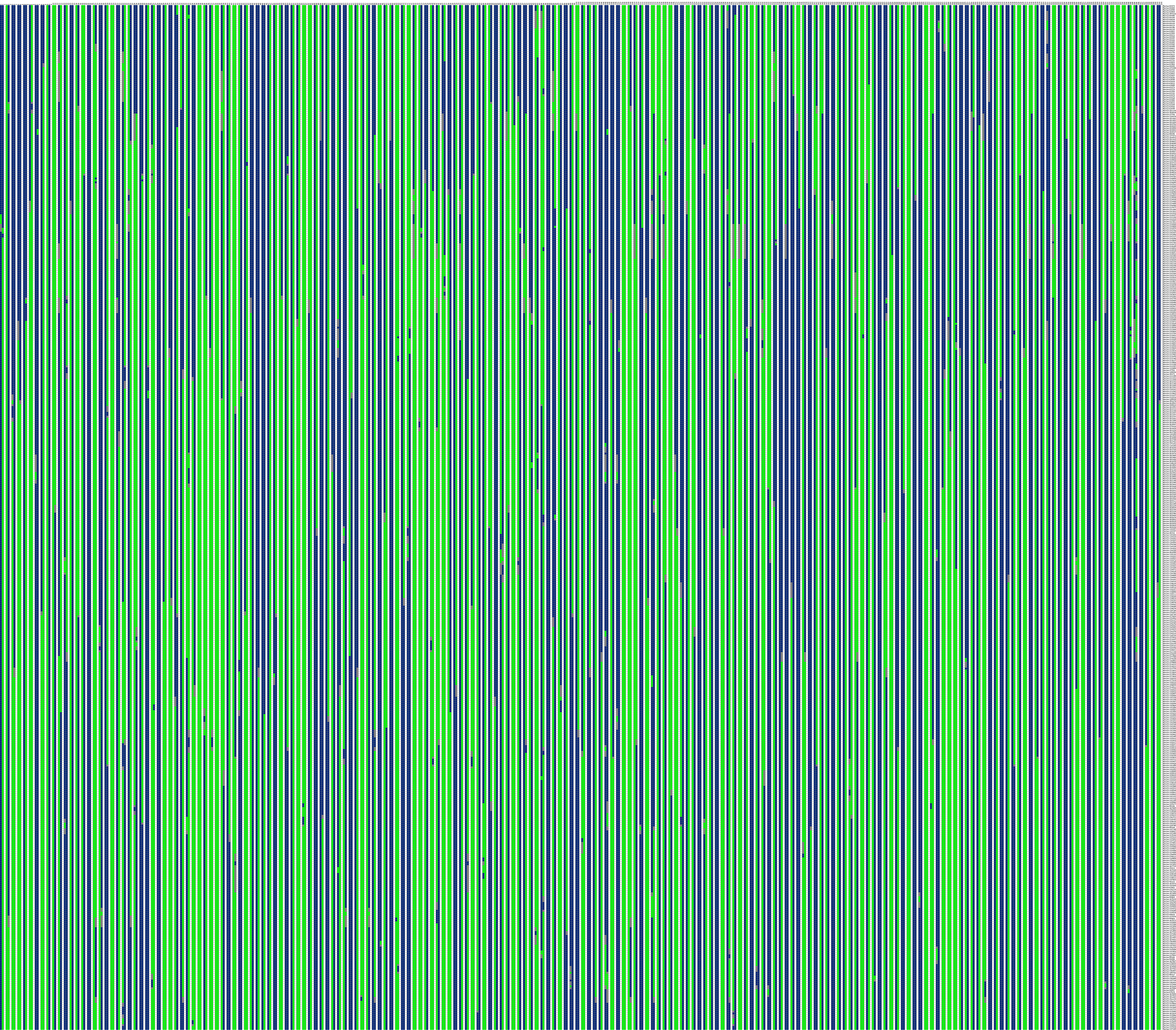

Supplement: Supplementary file 1 [file DataSheet_1.zip › Figure S5/sexAver/LG13.sexAver.haplo.png]

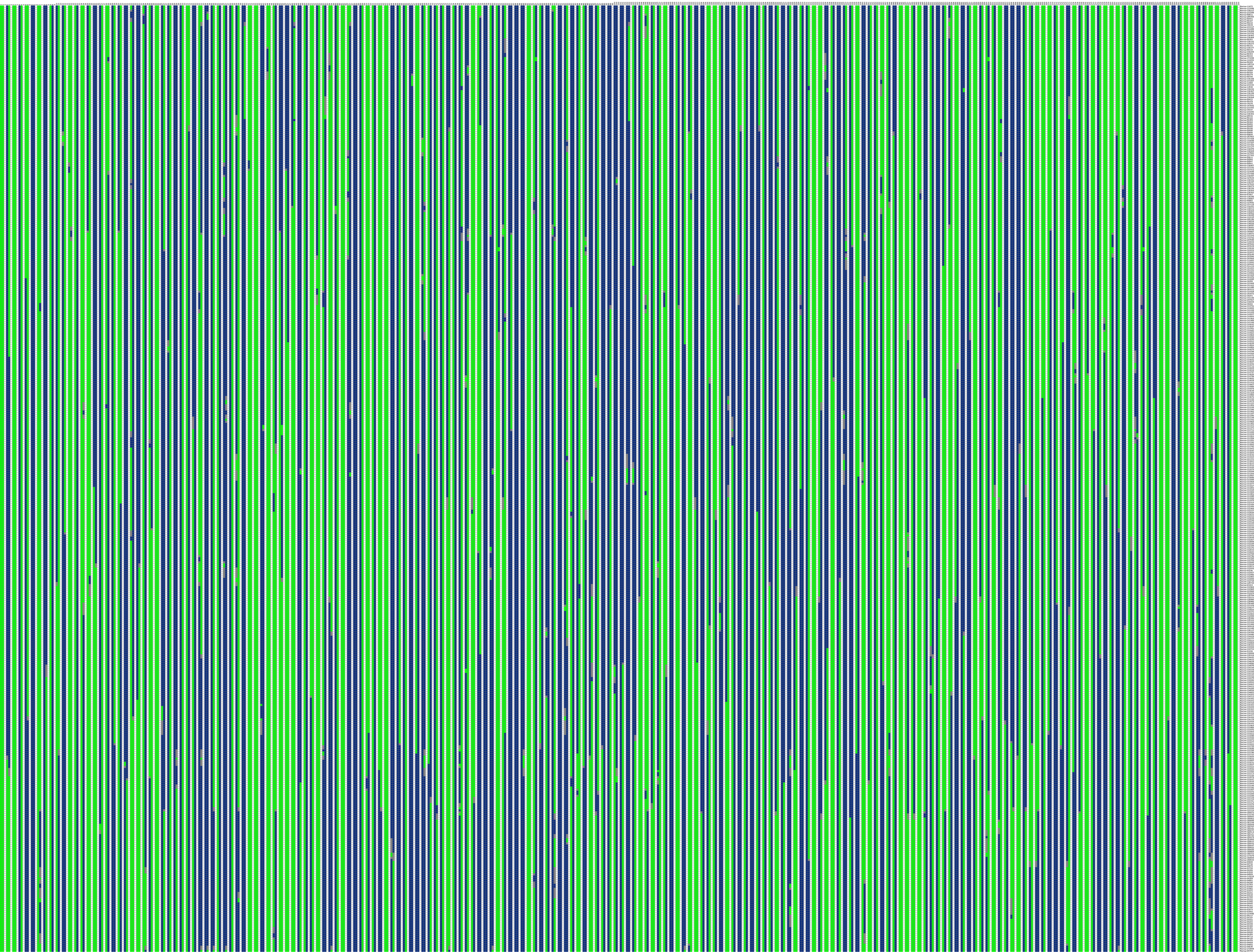

Supplement: Supplementary file 1 [file DataSheet_1.zip › Figure S5/sexAver/LG14.sexAver.haplo.png]

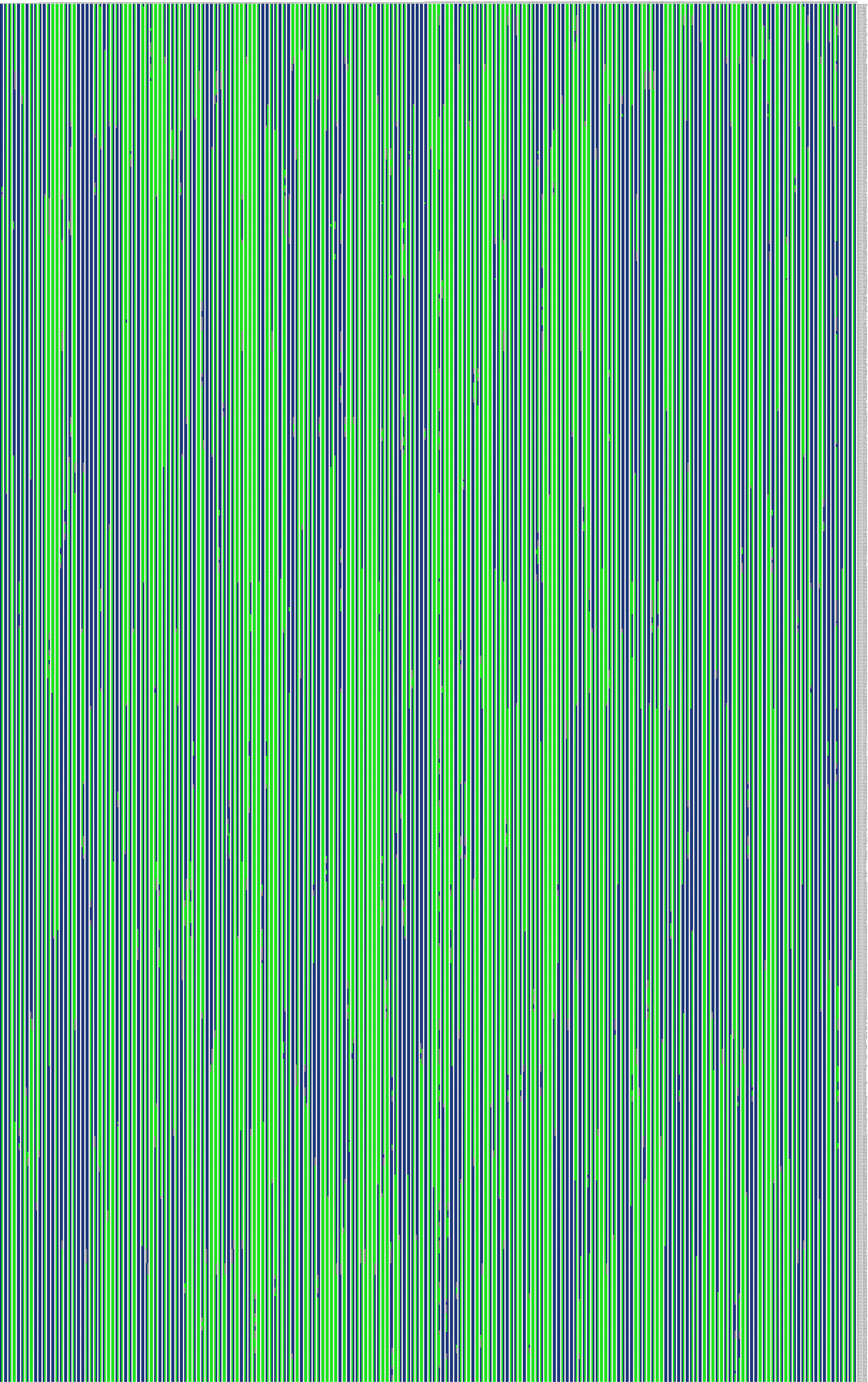

Supplement: Supplementary file 1 [file DataSheet_1.zip › Figure S5/sexAver/LG15.sexAver.haplo.png]

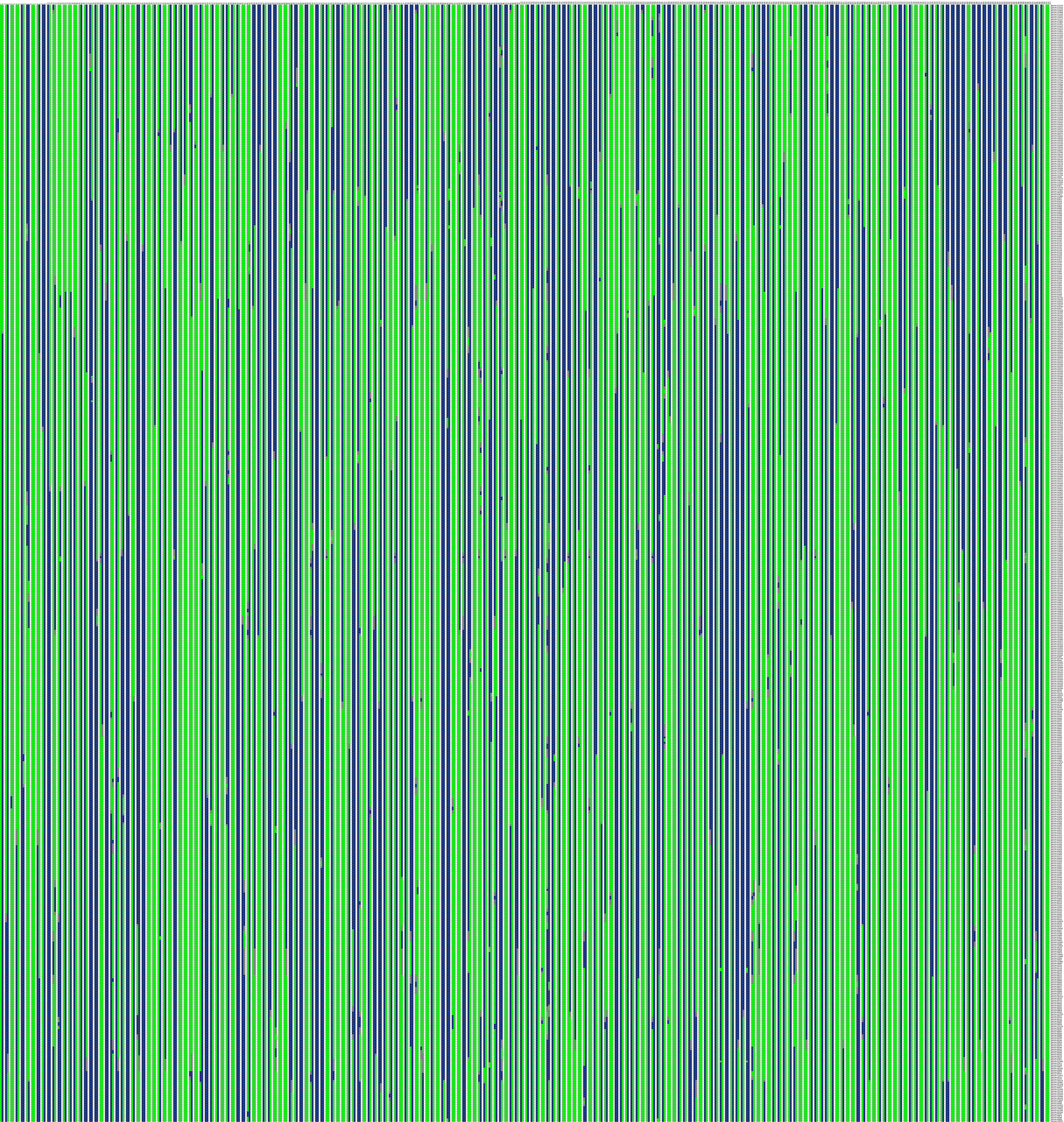

Supplement: Supplementary file 1 [file DataSheet_1.zip › Figure S5/sexAver/LG16.sexAver.haplo.png]

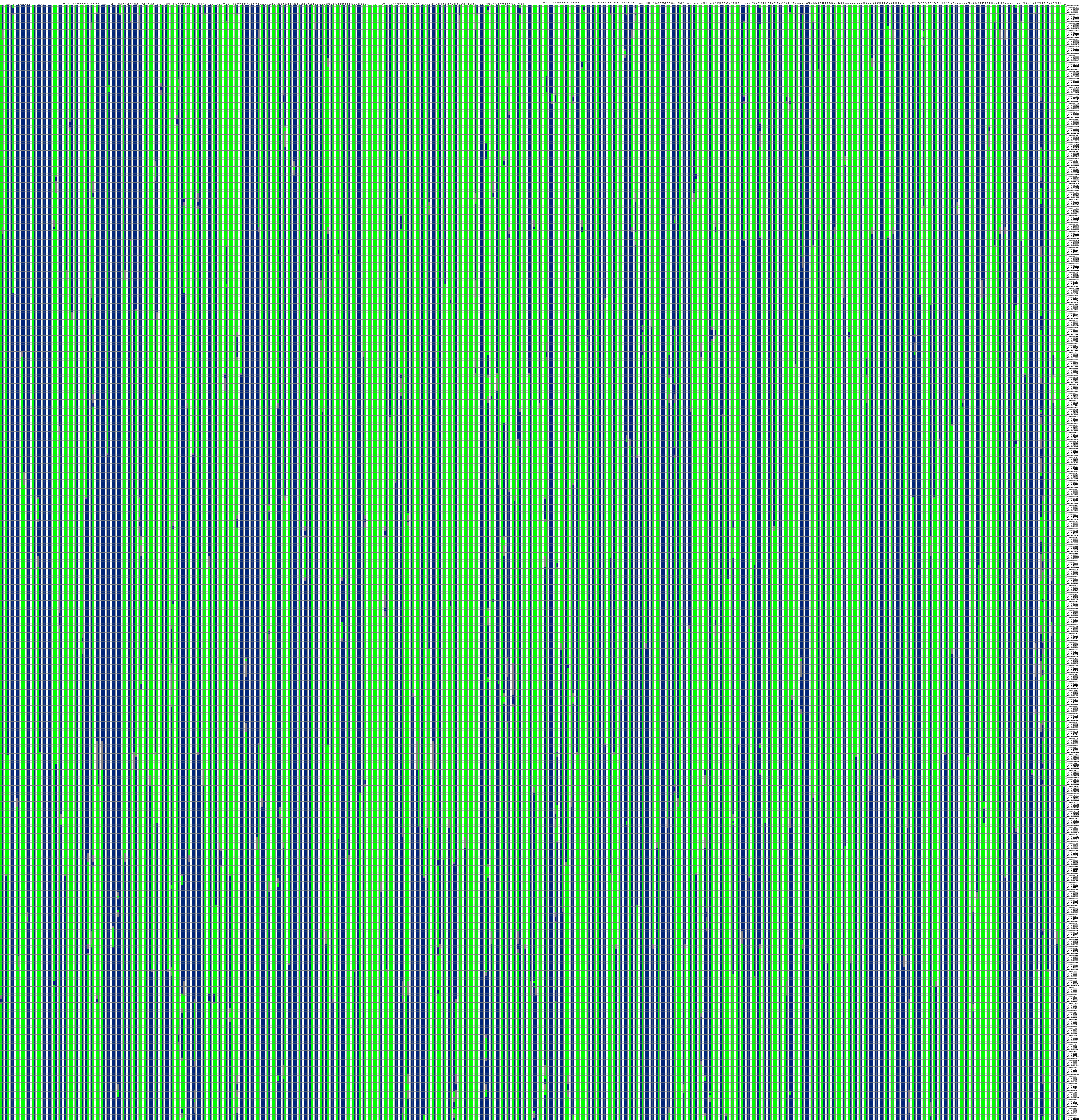

Supplement: Supplementary file 1 [file DataSheet_1.zip › Figure S5/sexAver/LG17.sexAver.haplo.png]

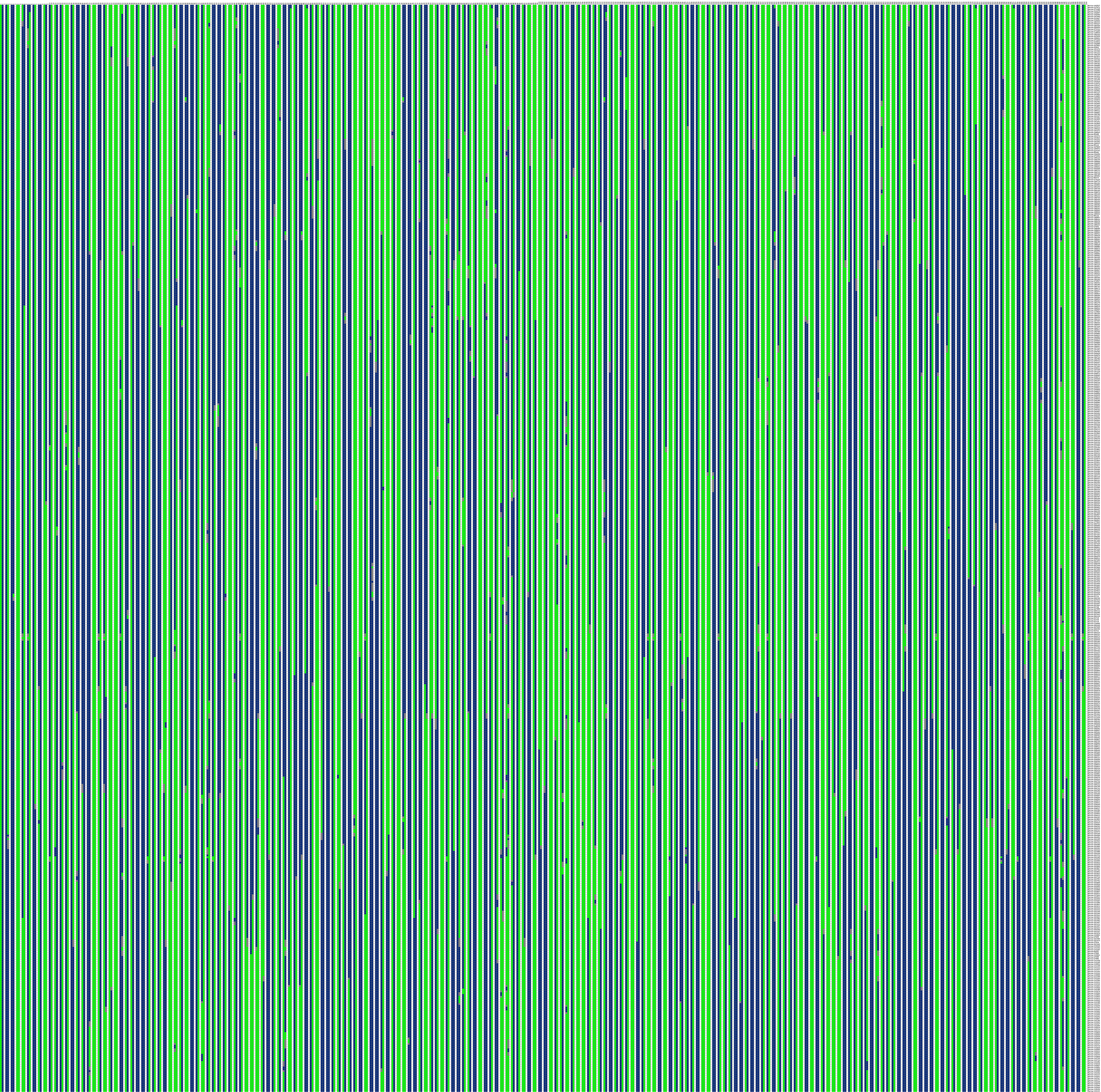

Supplement: Supplementary file 1 [file DataSheet_1.zip › Figure S5/sexAver/LG18.sexAver.haplo.png]

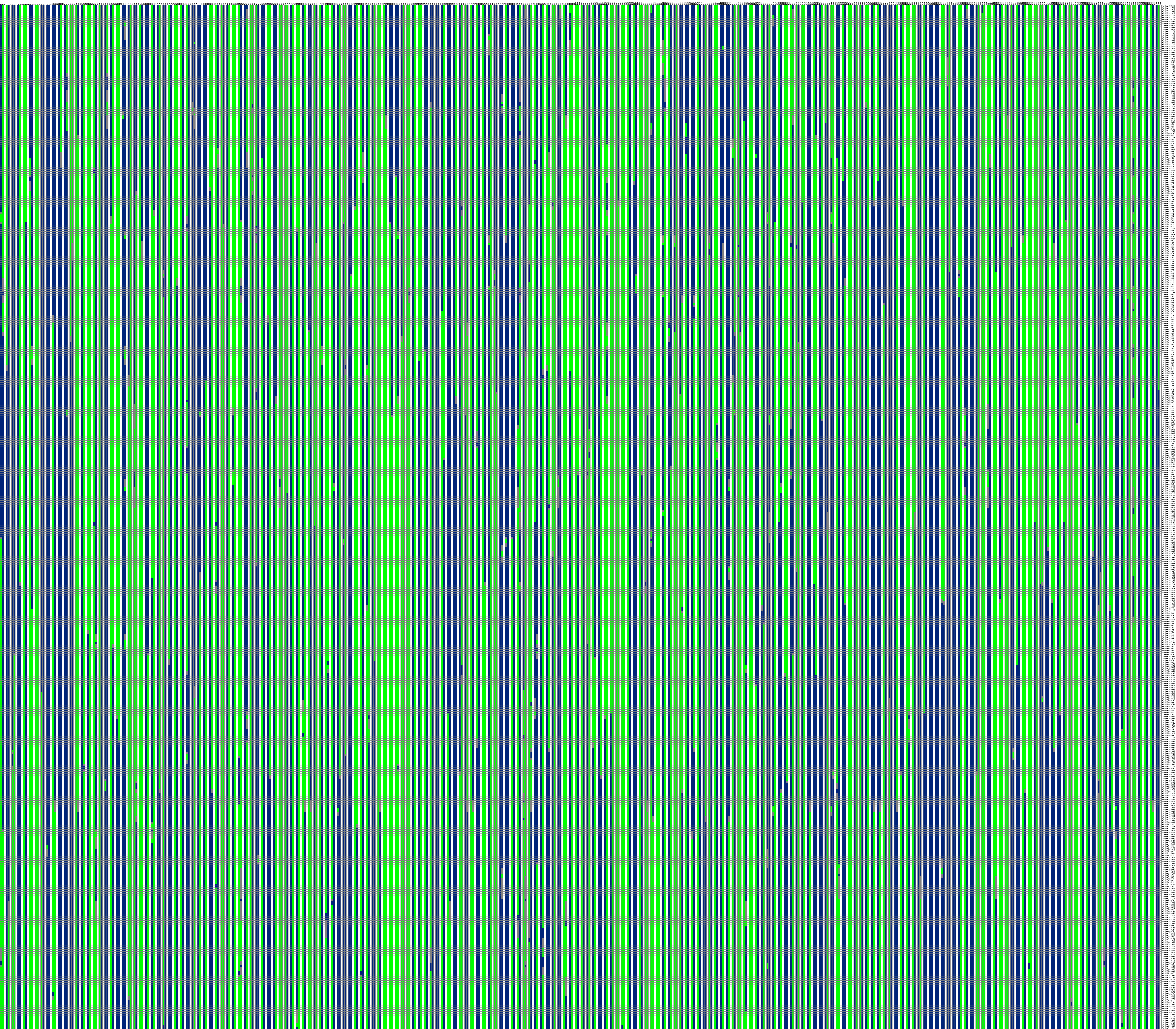

Supplement: Supplementary file 1 [file DataSheet_1.zip › Figure S5/sexAver/LG19.sexAver.haplo.png]

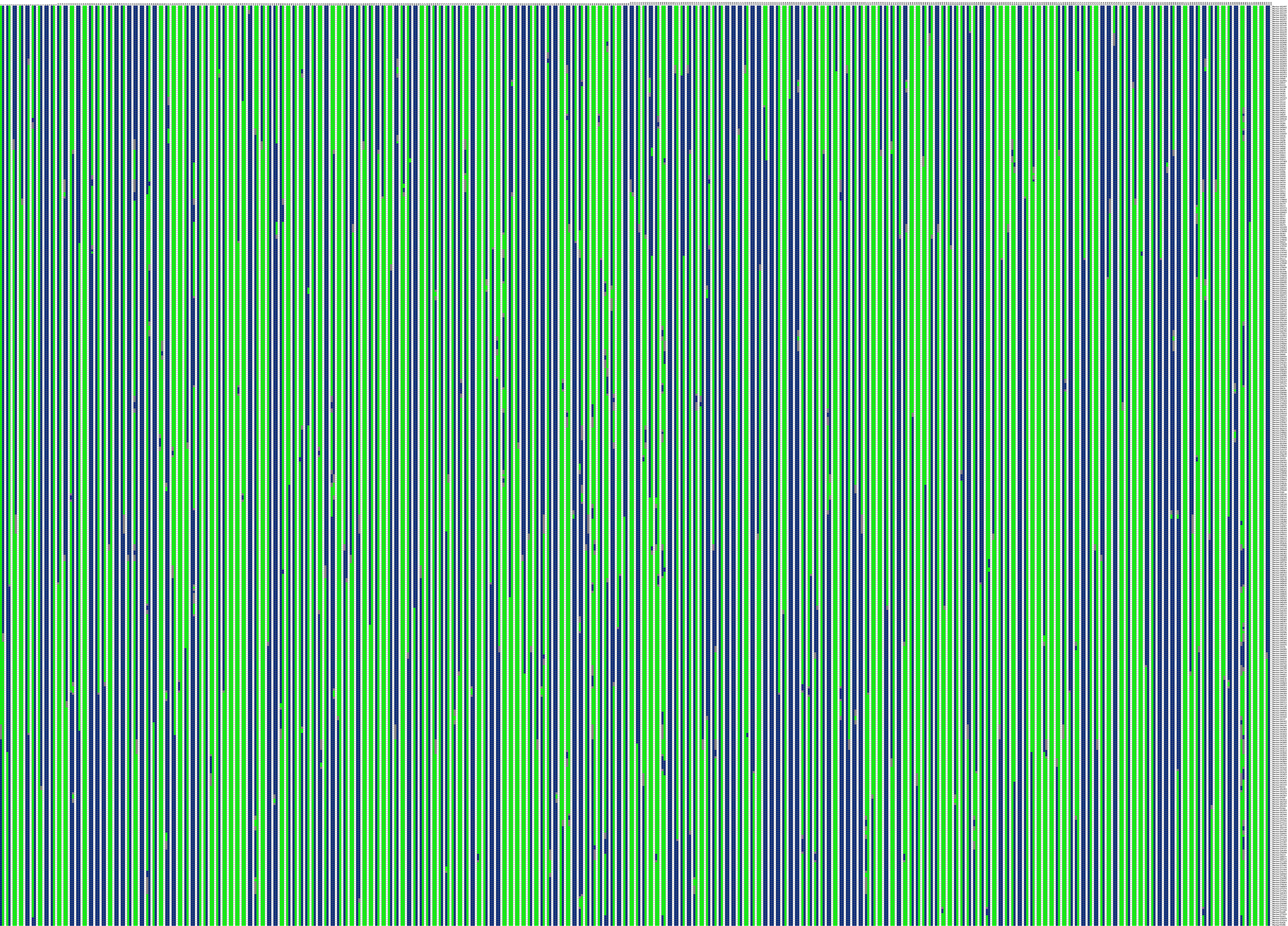

Supplement: Supplementary file 1 [file DataSheet_1.zip › Figure S5/sexAver/LG2.sexAver.haplo.png]

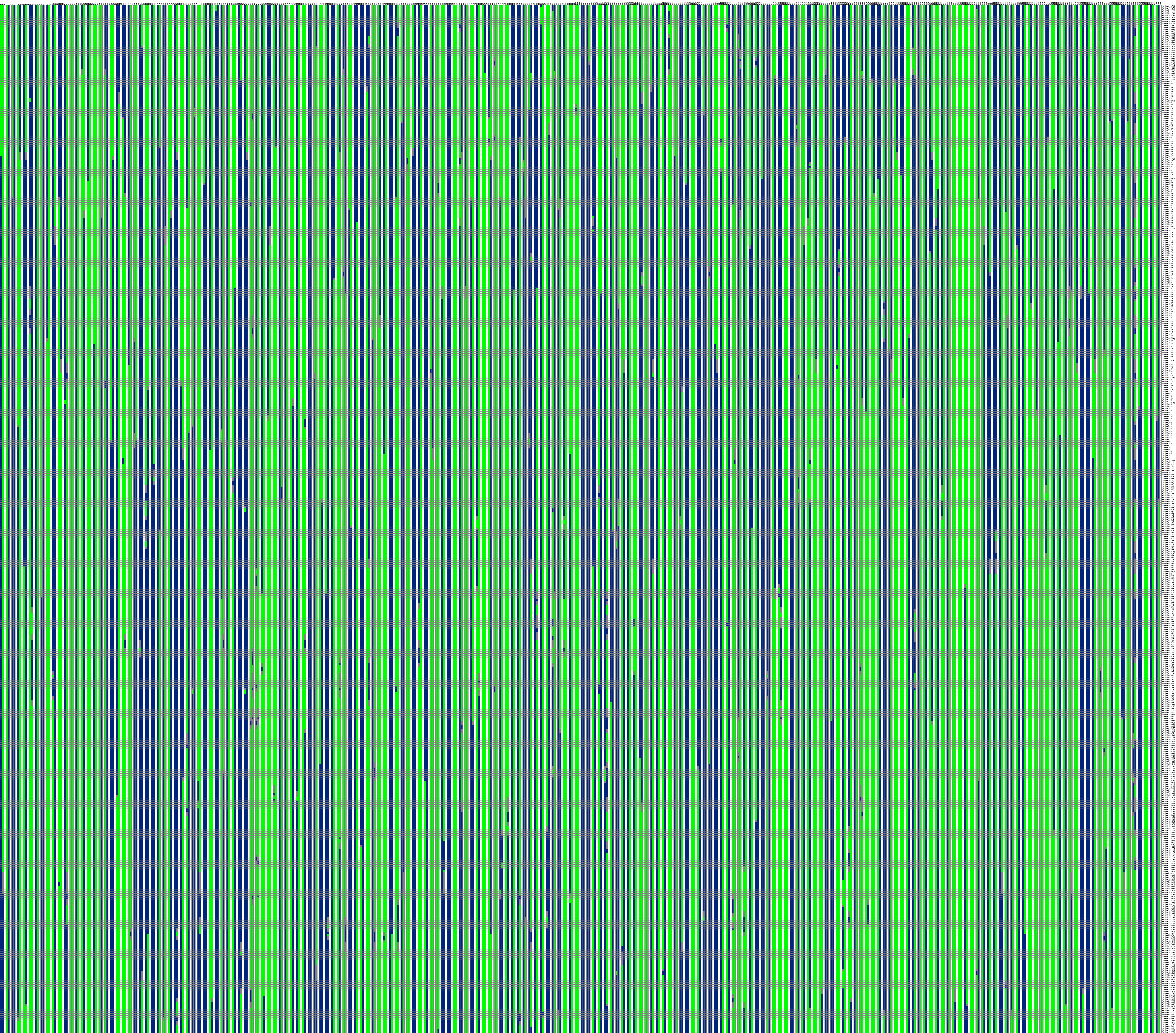

Supplement: Supplementary file 1 [file DataSheet_1.zip › Figure S5/sexAver/LG20.sexAver.haplo.png]

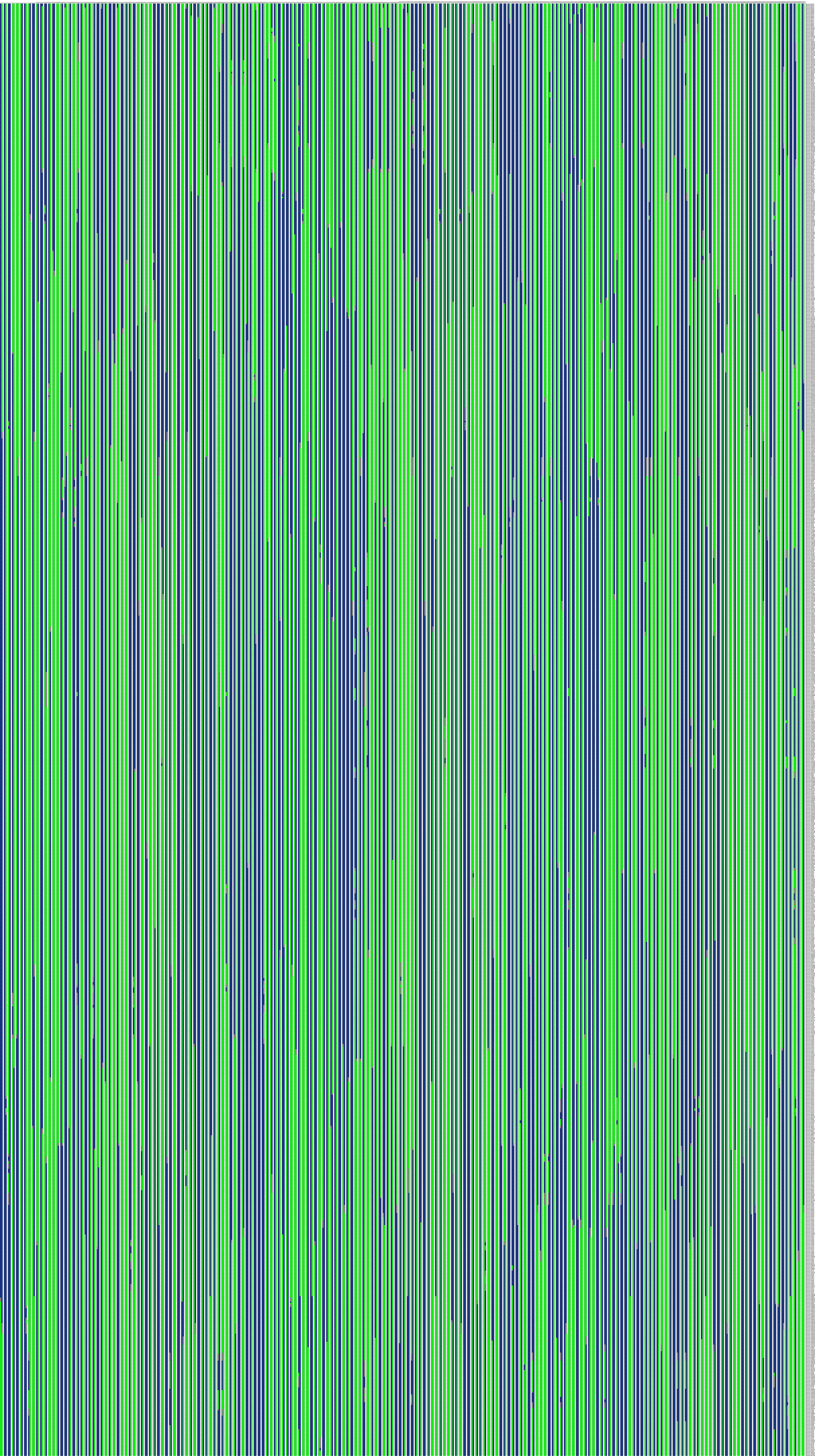

Supplement: Supplementary file 1 [file DataSheet_1.zip › Figure S5/sexAver/LG21.sexAver.haplo.png]

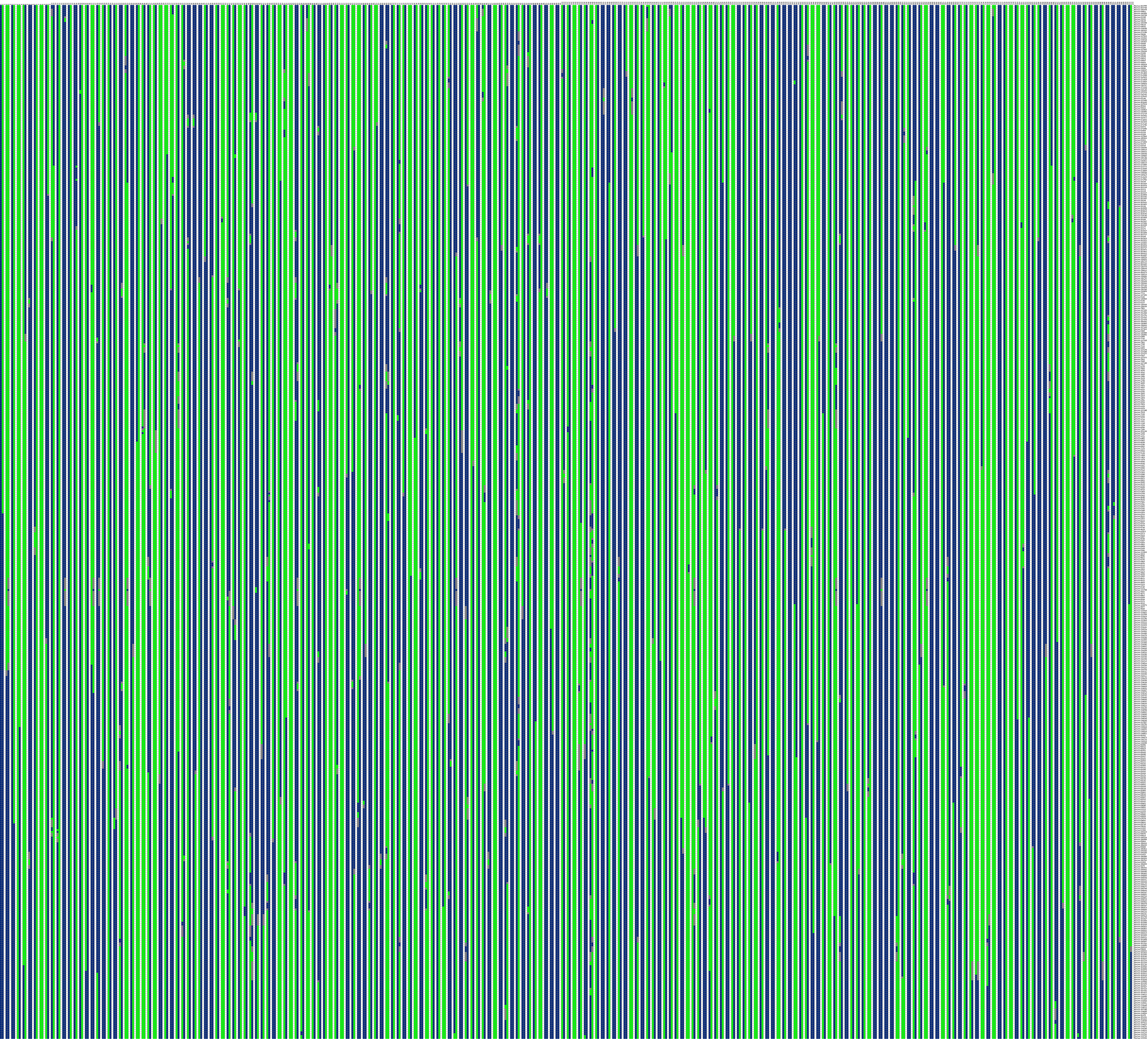

Supplement: Supplementary file 1 [file DataSheet_1.zip › Figure S5/sexAver/LG22.sexAver.haplo.png]

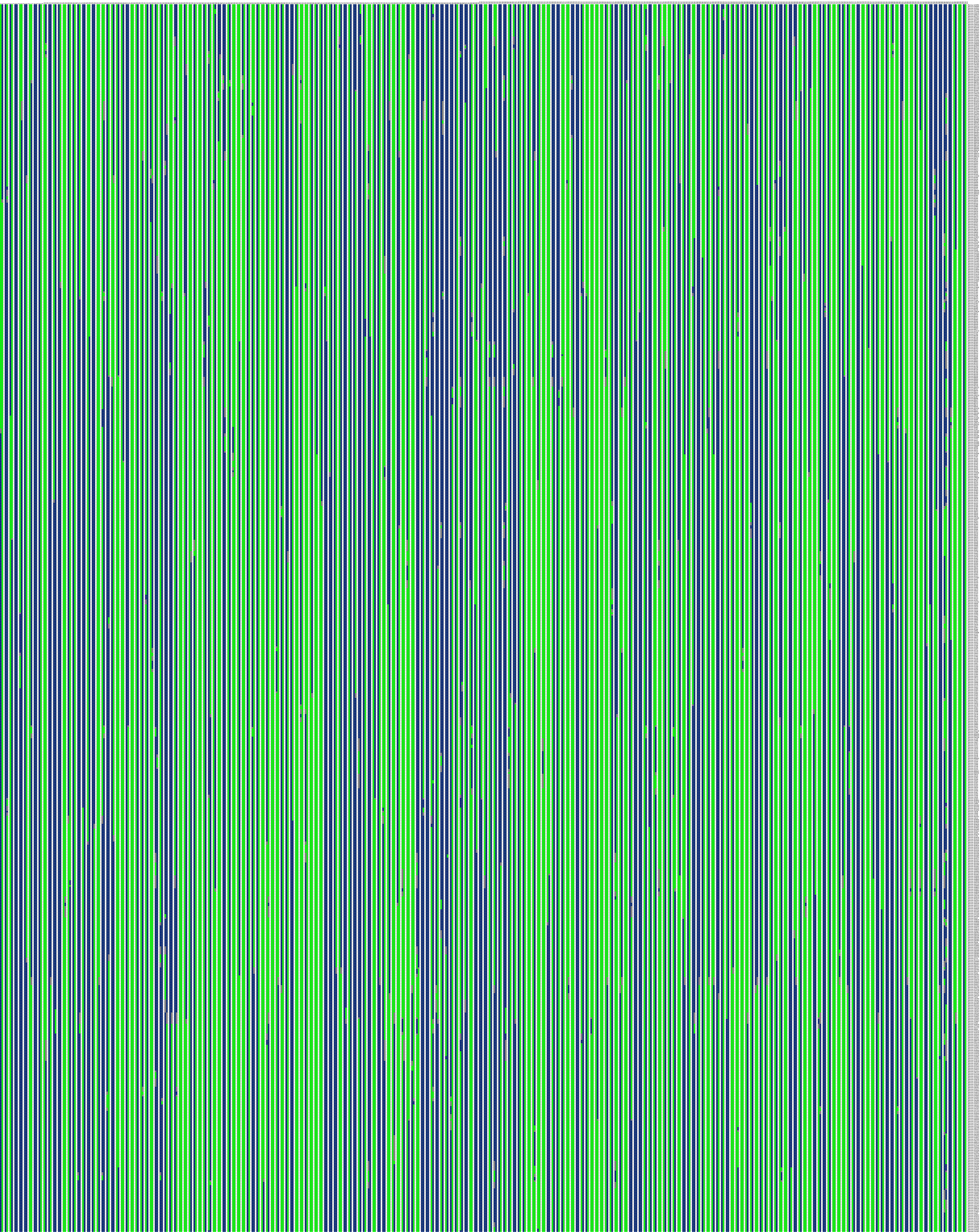

Supplement: Supplementary file 1 [file DataSheet_1.zip › Figure S5/sexAver/LG23.sexAver.haplo.png]

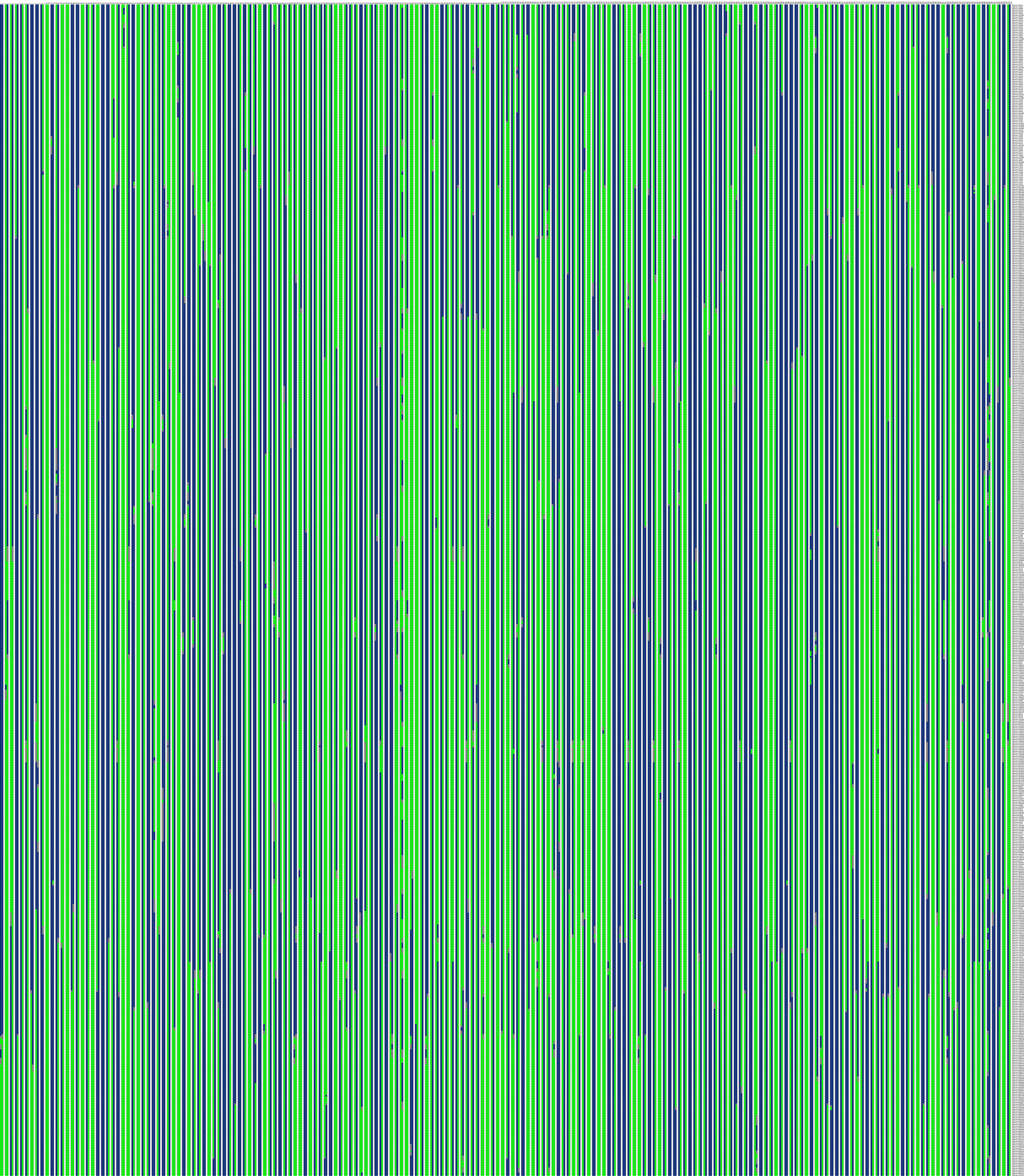

Supplement: Supplementary file 1 [file DataSheet_1.zip › Figure S5/sexAver/LG24.sexAver.haplo.png]

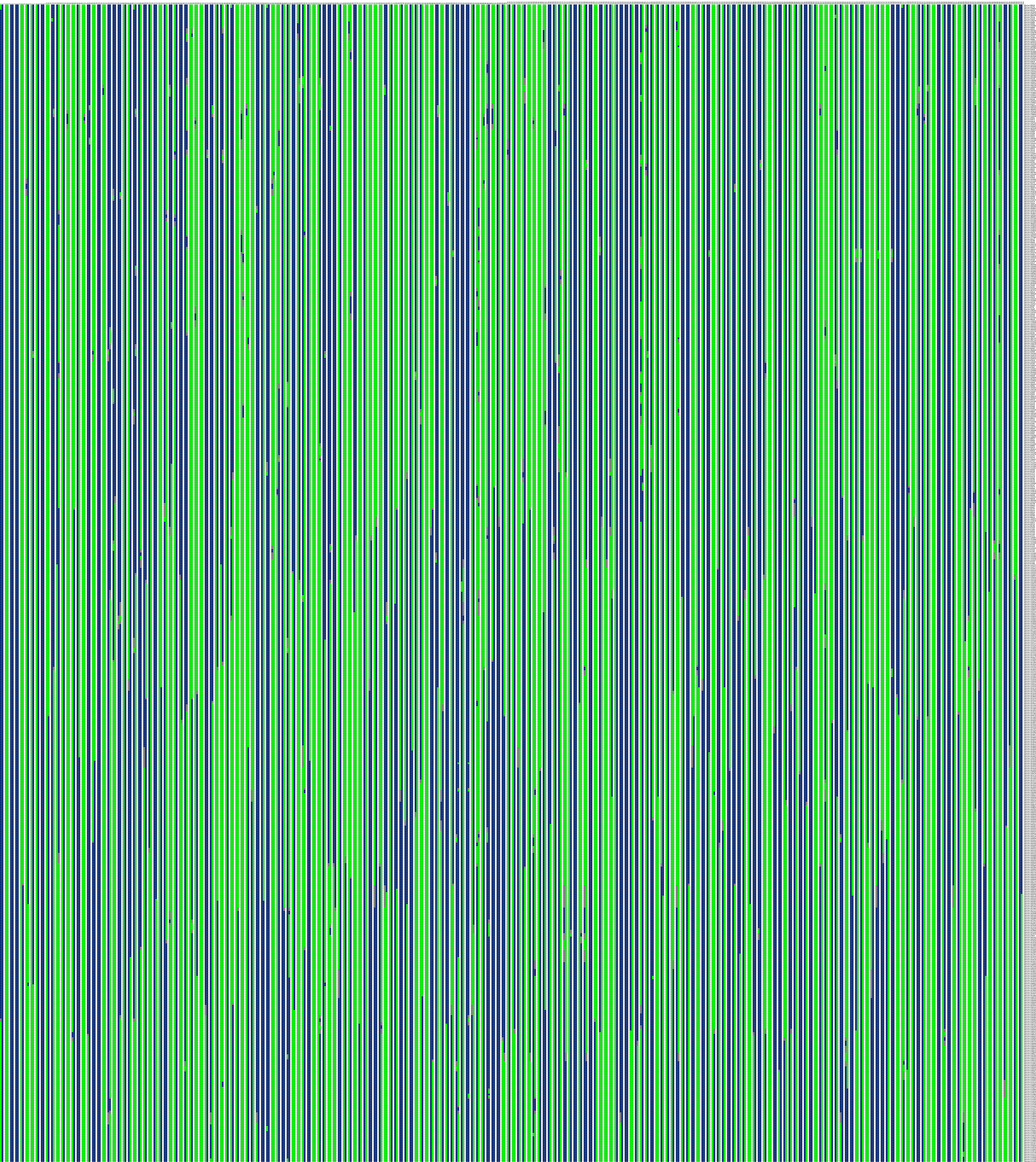

Supplement: Supplementary file 1 [file DataSheet_1.zip › Figure S5/sexAver/LG3.sexAver.haplo.png]

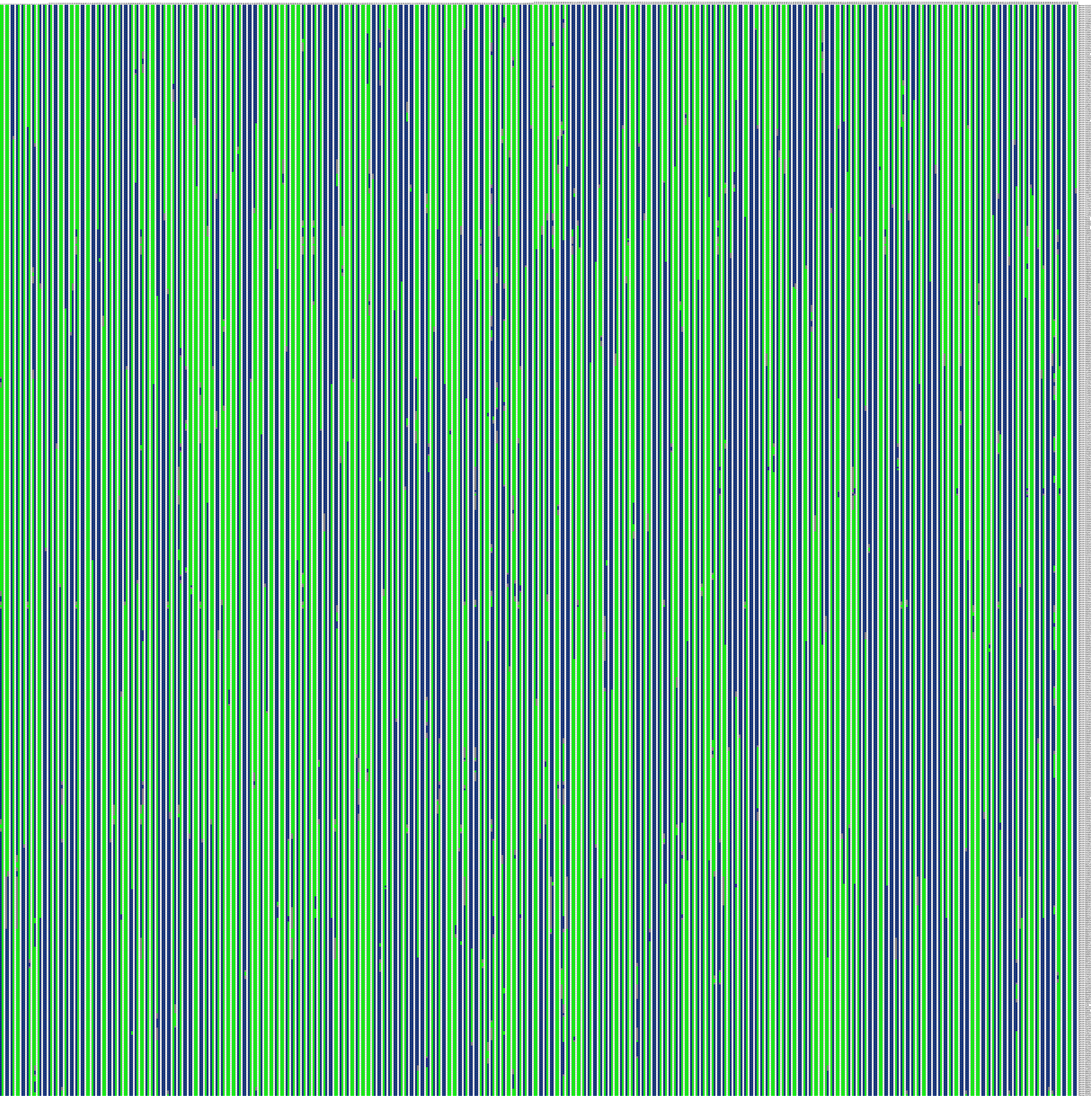

Supplement: Supplementary file 1 [file DataSheet_1.zip › Figure S5/sexAver/LG4.sexAver.haplo.png]

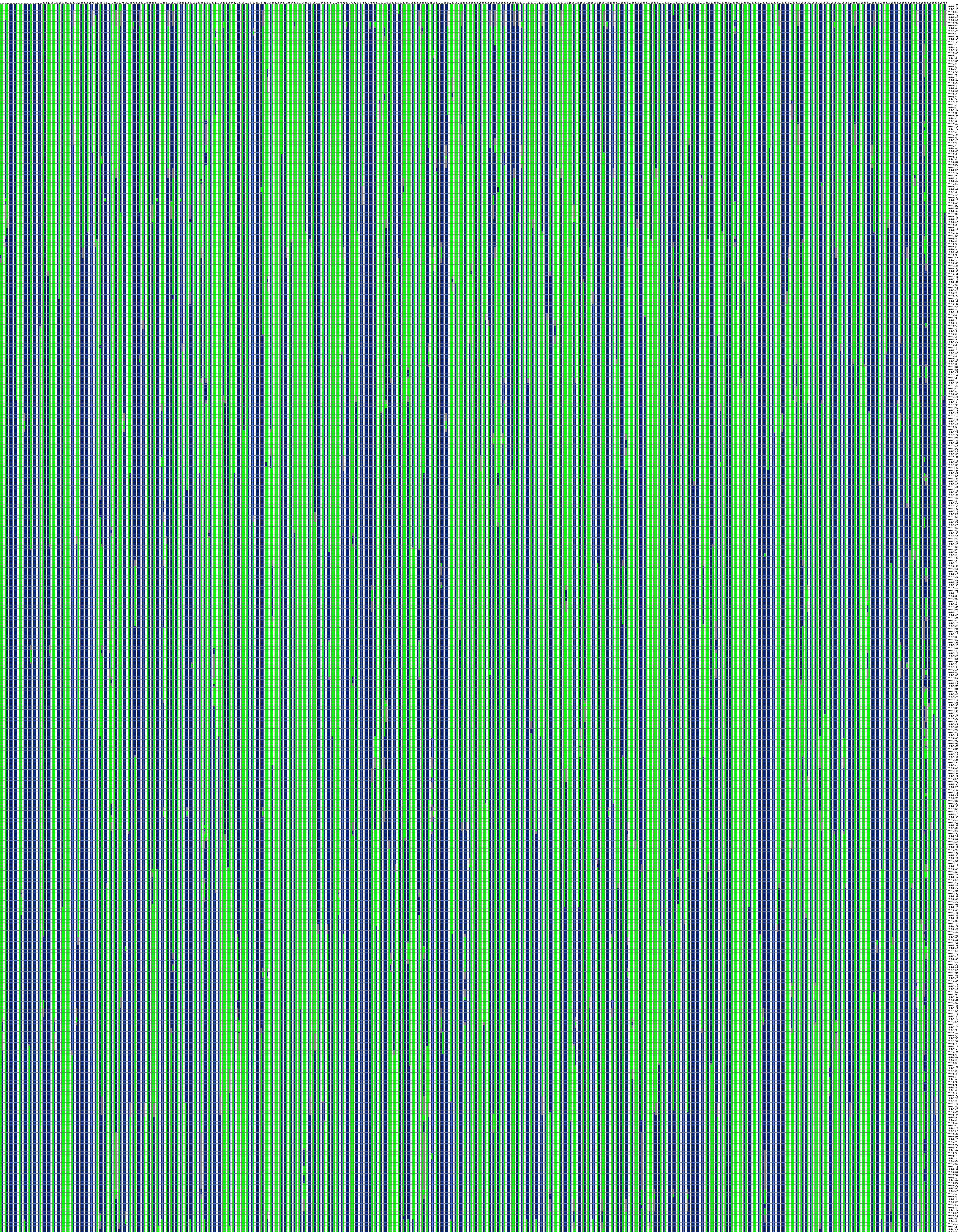

Supplement: Supplementary file 1 [file DataSheet_1.zip › Figure S5/sexAver/LG5.sexAver.haplo.png]

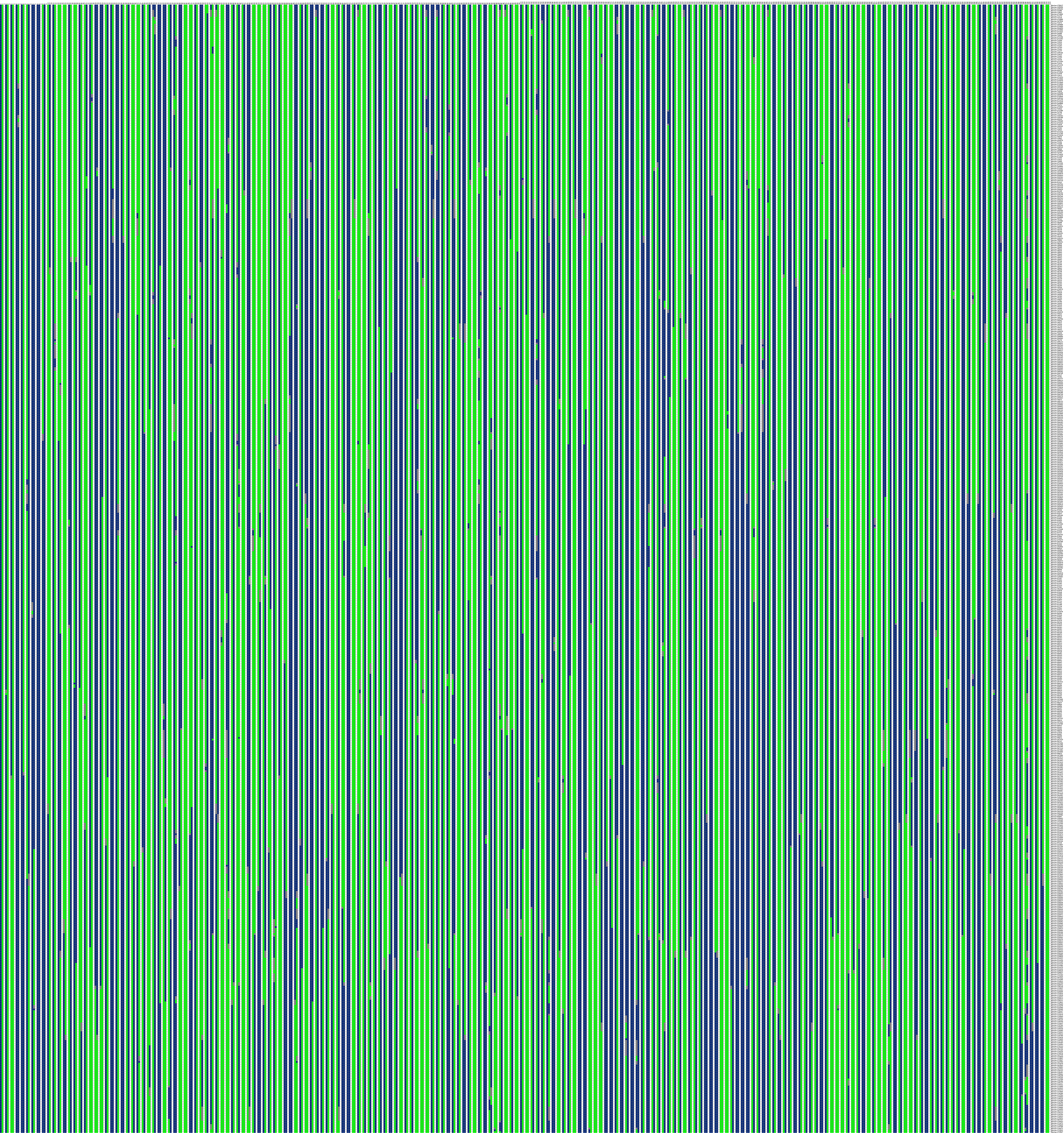

Supplement: Supplementary file 1 [file DataSheet_1.zip › Figure S5/sexAver/LG6.sexAver.haplo.png]

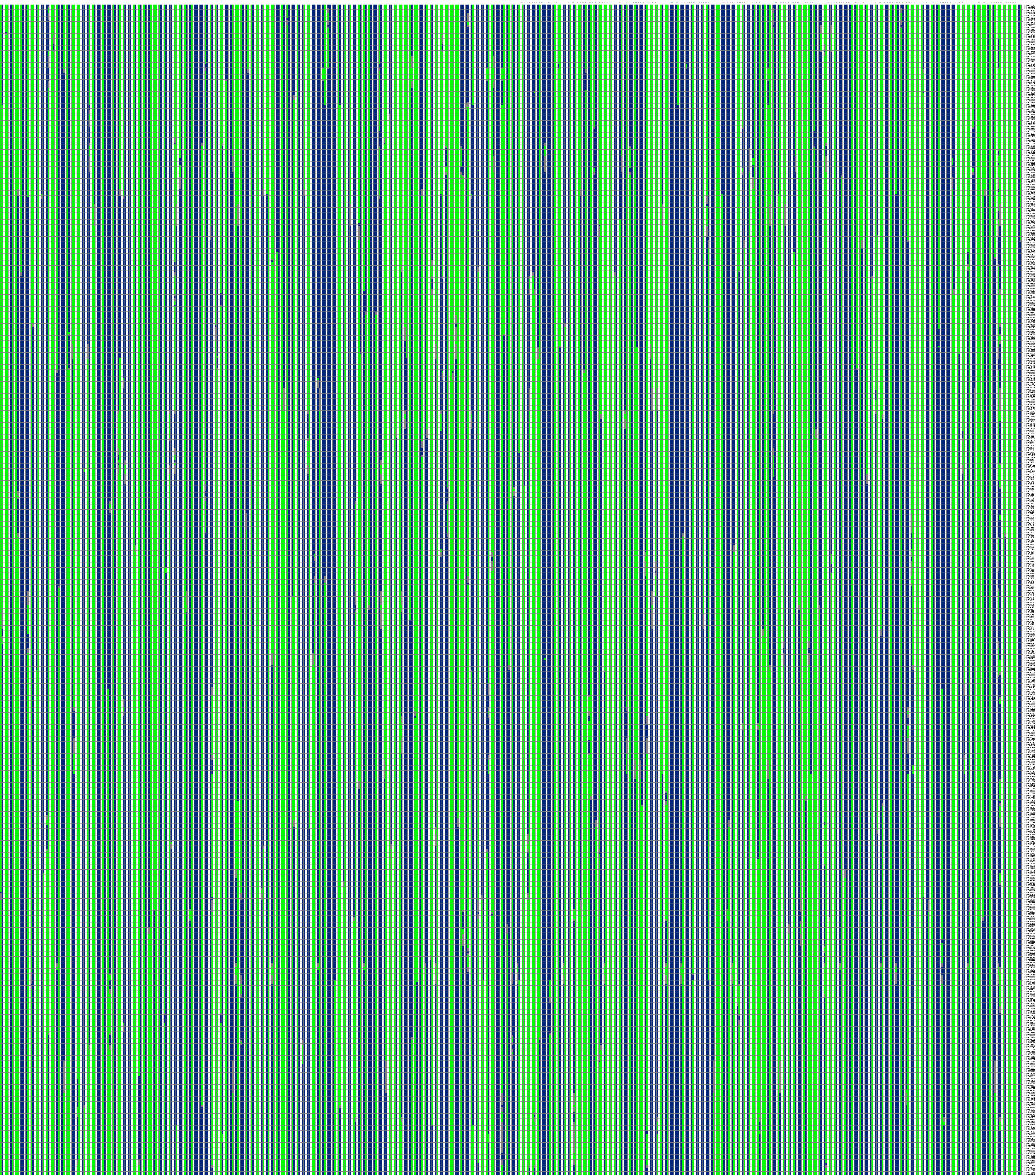

Supplement: Supplementary file 1 [file DataSheet_1.zip › Figure S5/sexAver/LG7.sexAver.haplo.png]

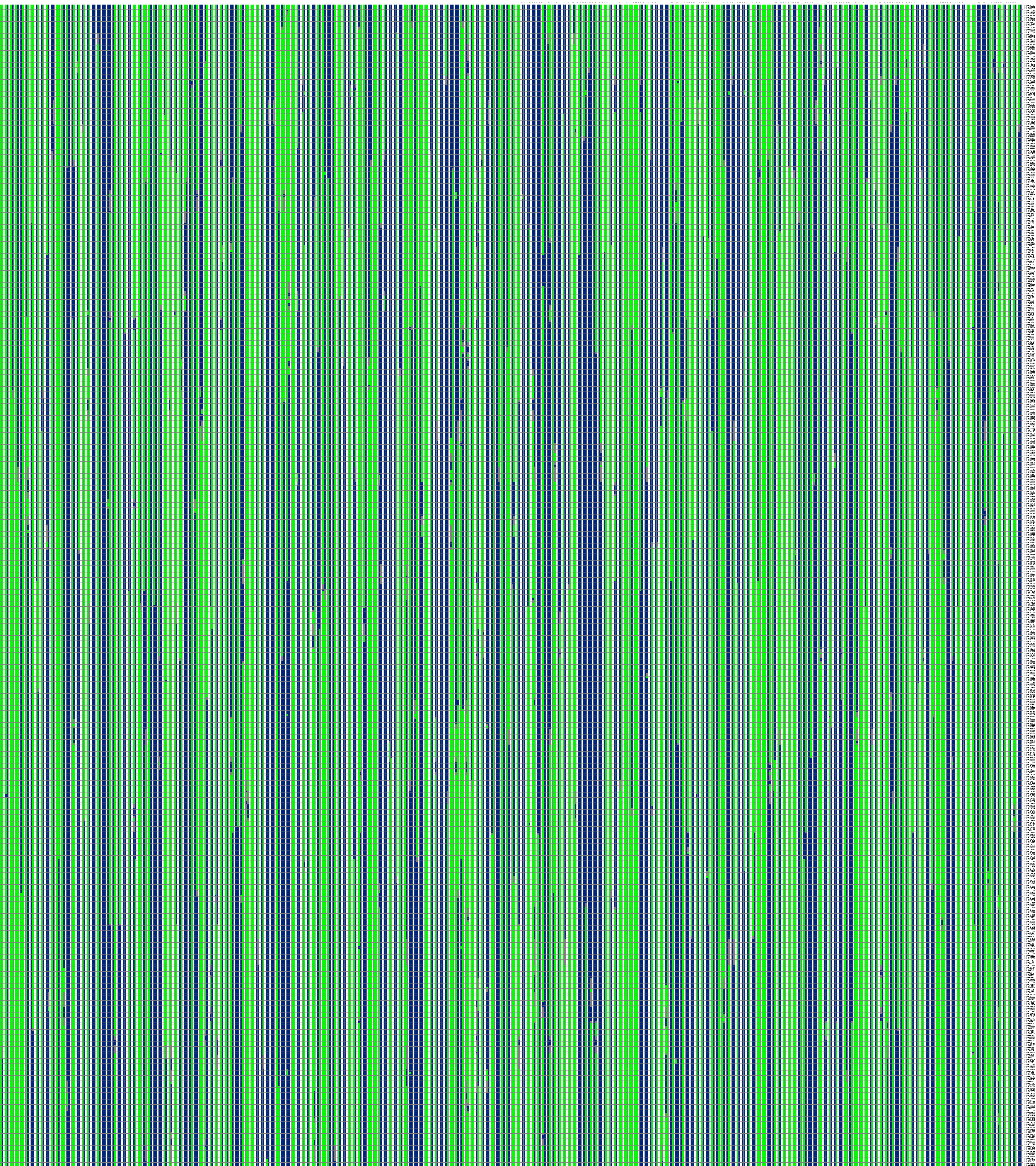

Supplement: Supplementary file 1 [file DataSheet_1.zip › Figure S5/sexAver/LG8.sexAver.haplo.png]

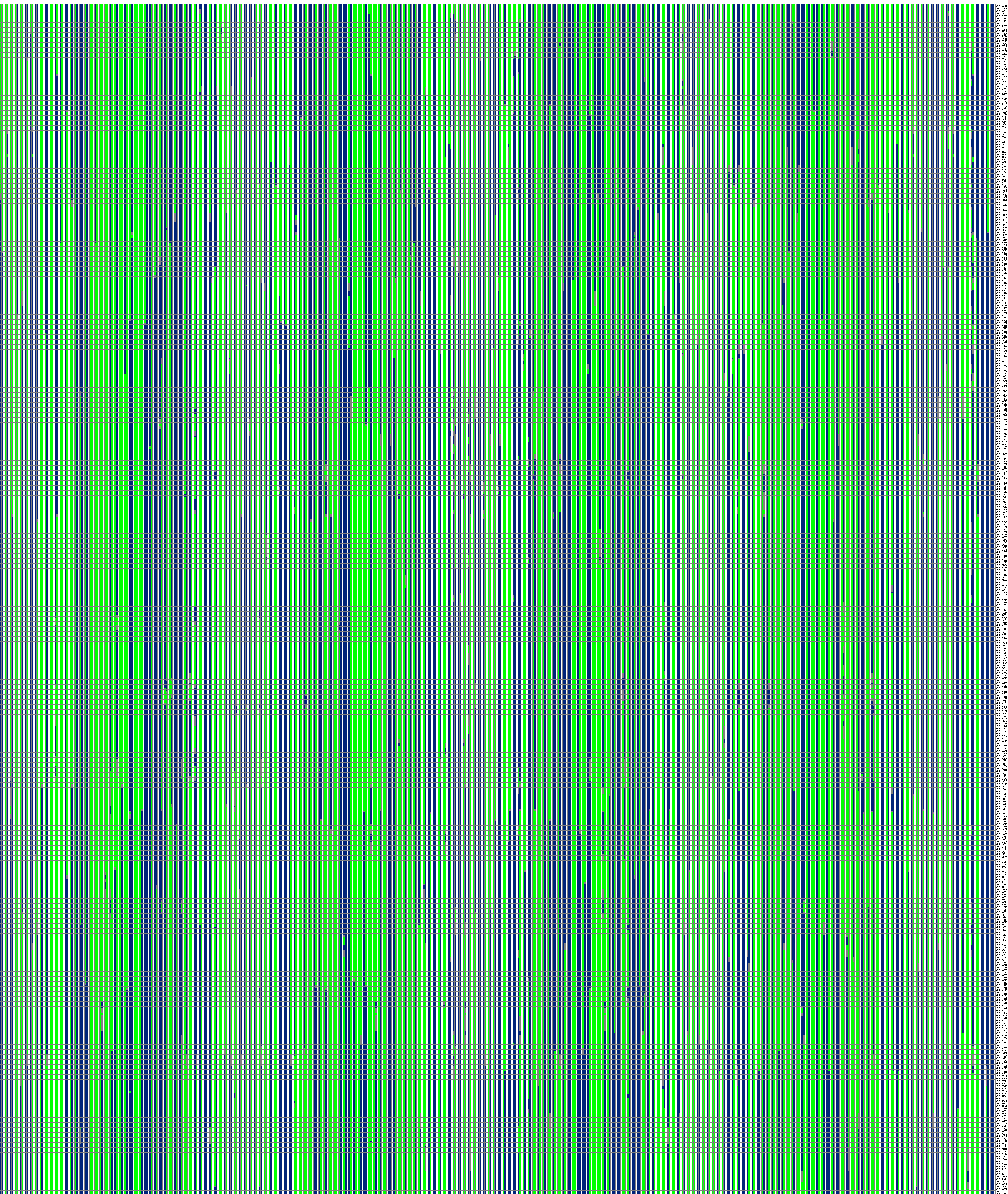

Supplement: Supplementary file 1 [file DataSheet_1.zip › Figure S5/sexAver/LG9.sexAver.haplo.png]

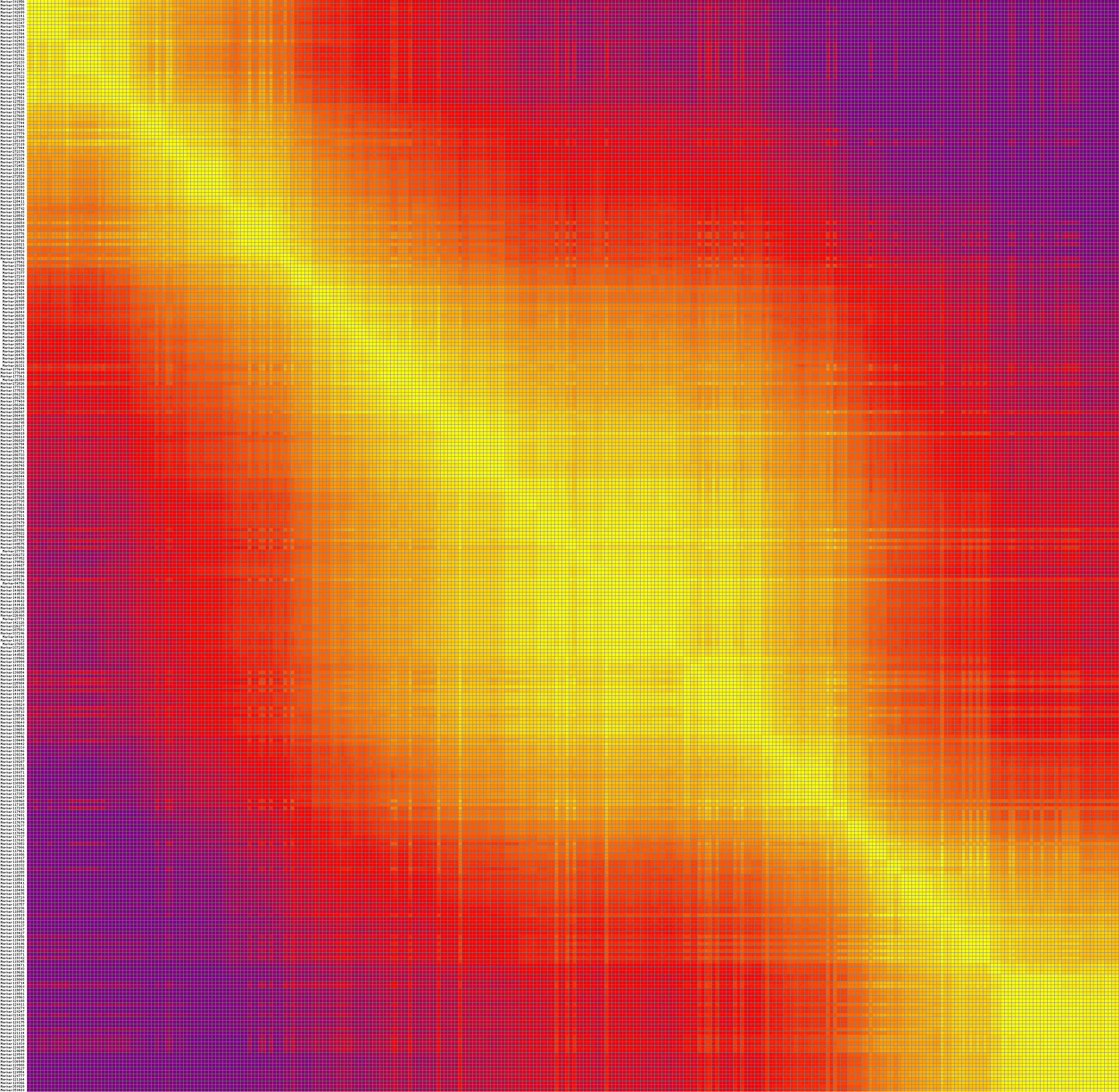

Supplement: Supplementary file 2 [file DataSheet_2.zip › Figure S6/female/LG1.female.heatMap.png]

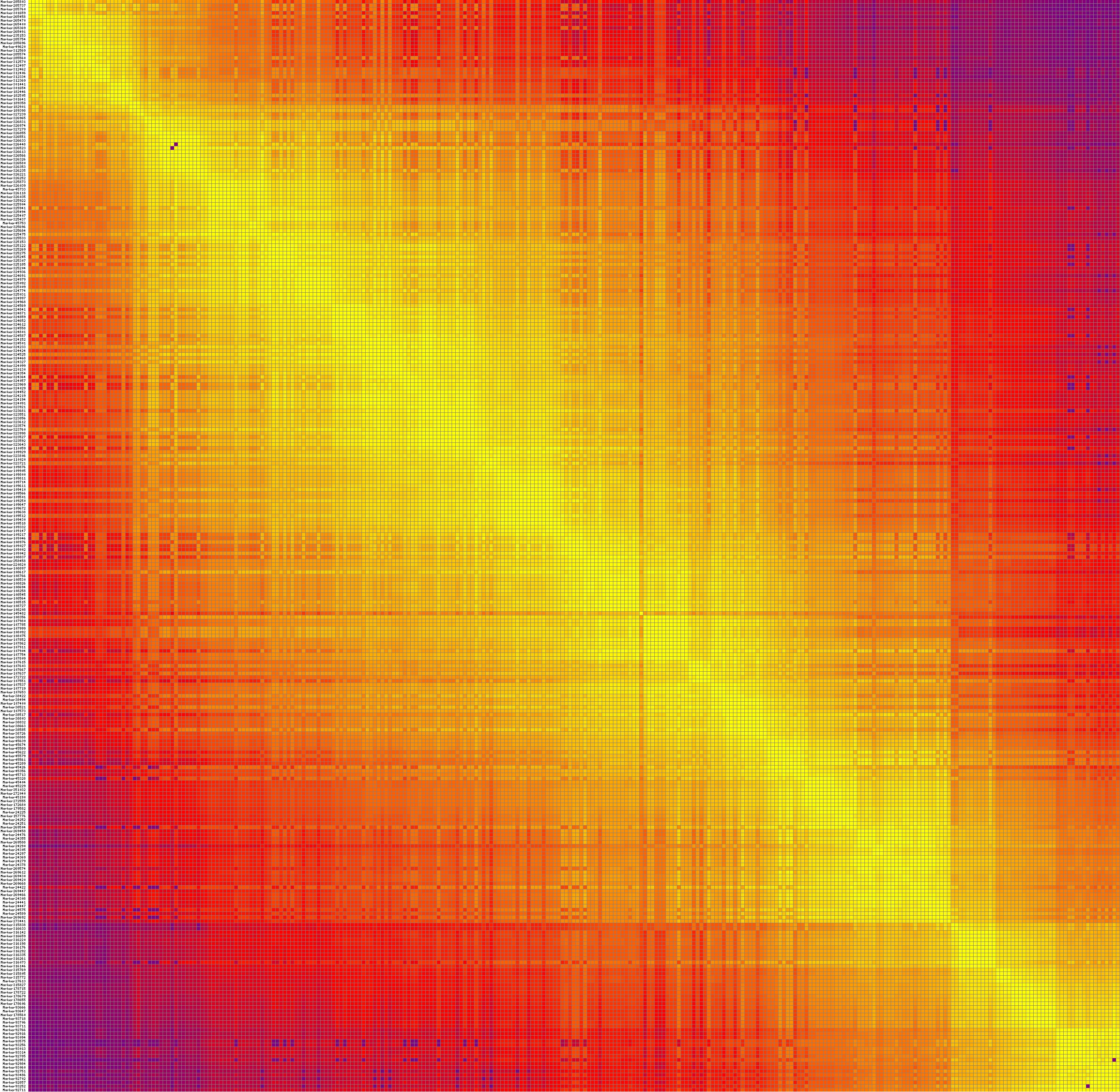

Supplement: Supplementary file 2 [file DataSheet_2.zip › Figure S6/female/LG10.female.heatMap.png]

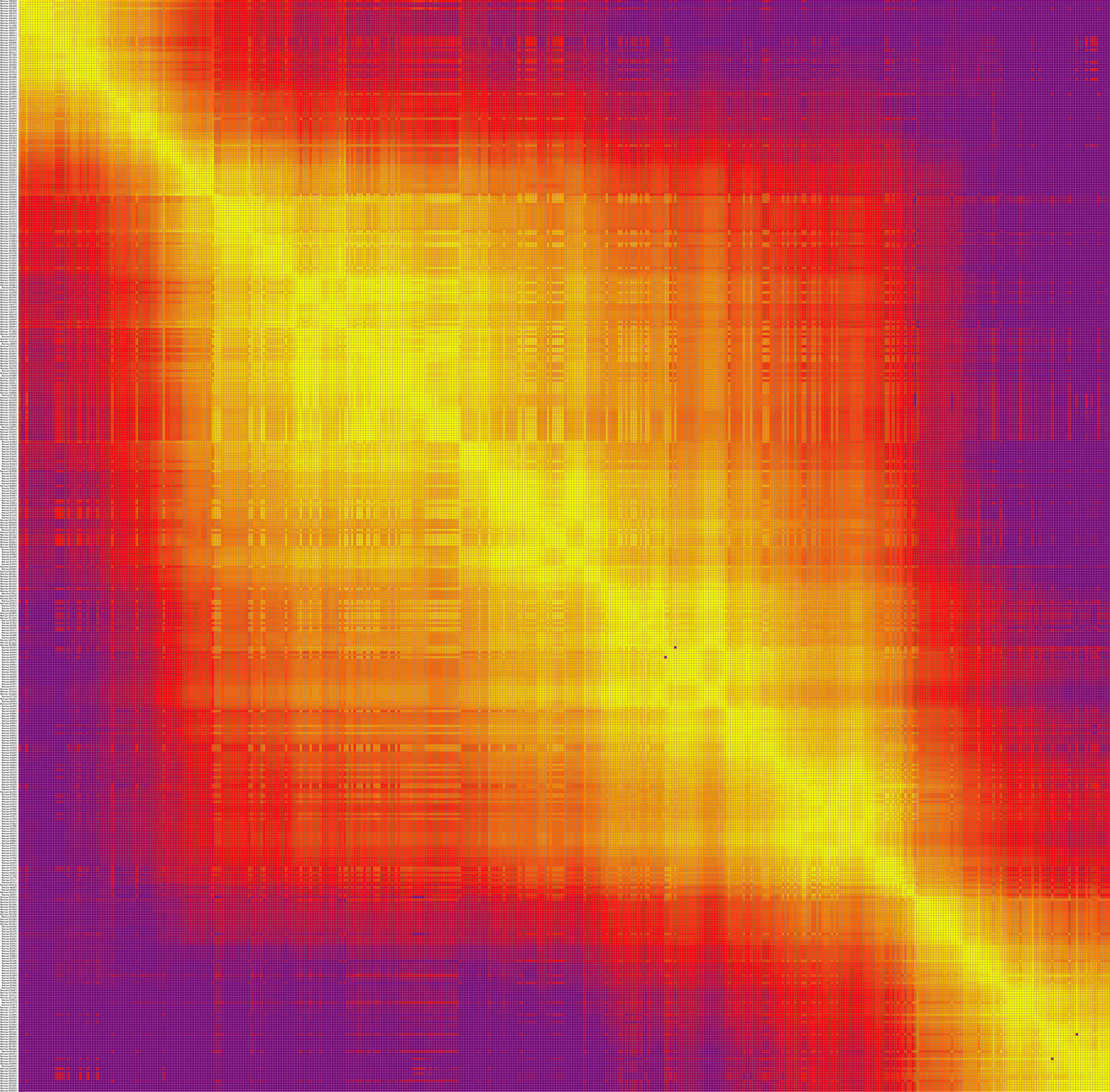

Supplement: Supplementary file 2 [file DataSheet_2.zip › Figure S6/female/LG11.female.heatMap.png]

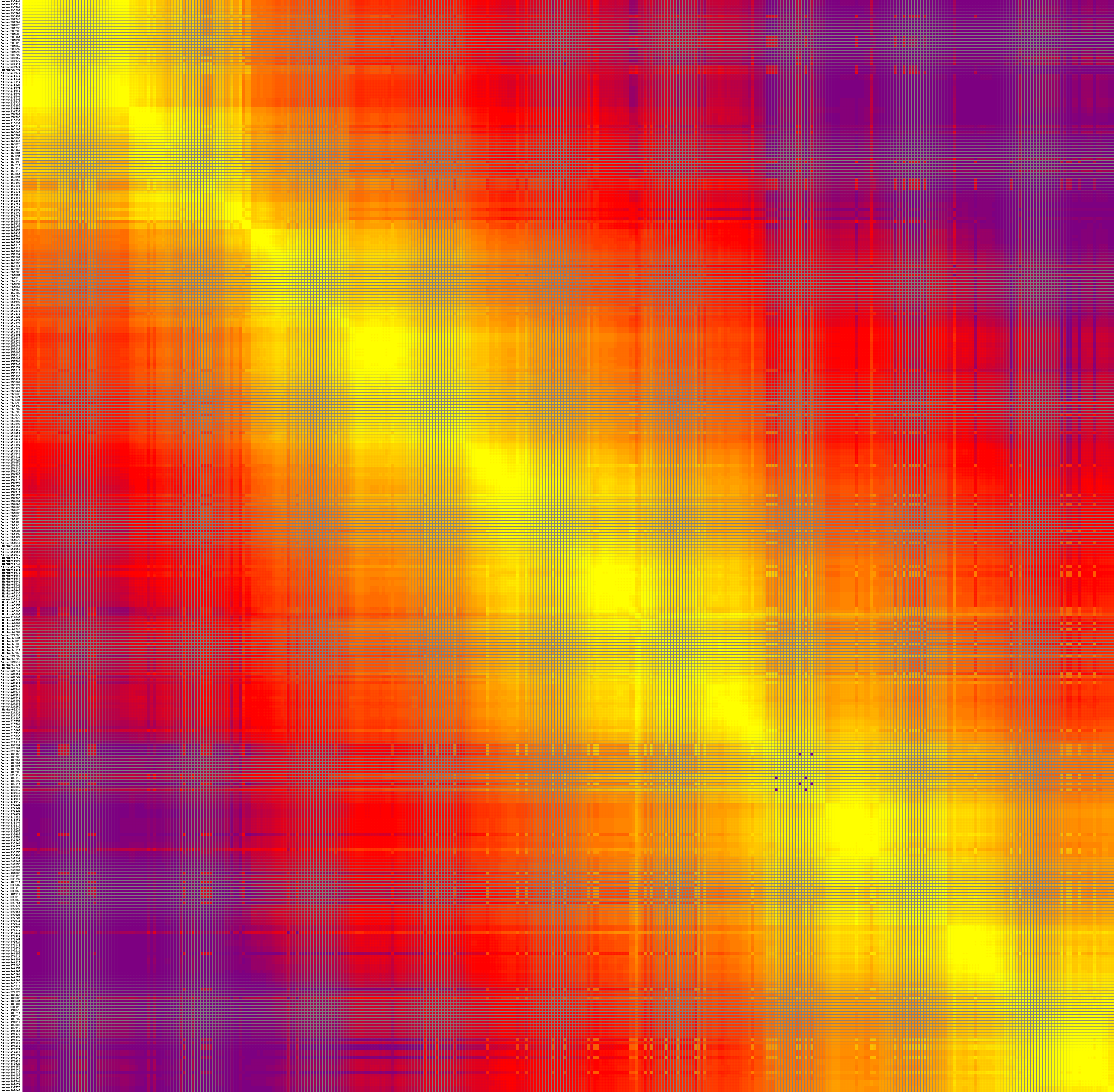

Supplement: Supplementary file 2 [file DataSheet_2.zip › Figure S6/female/LG12.female.heatMap.png]

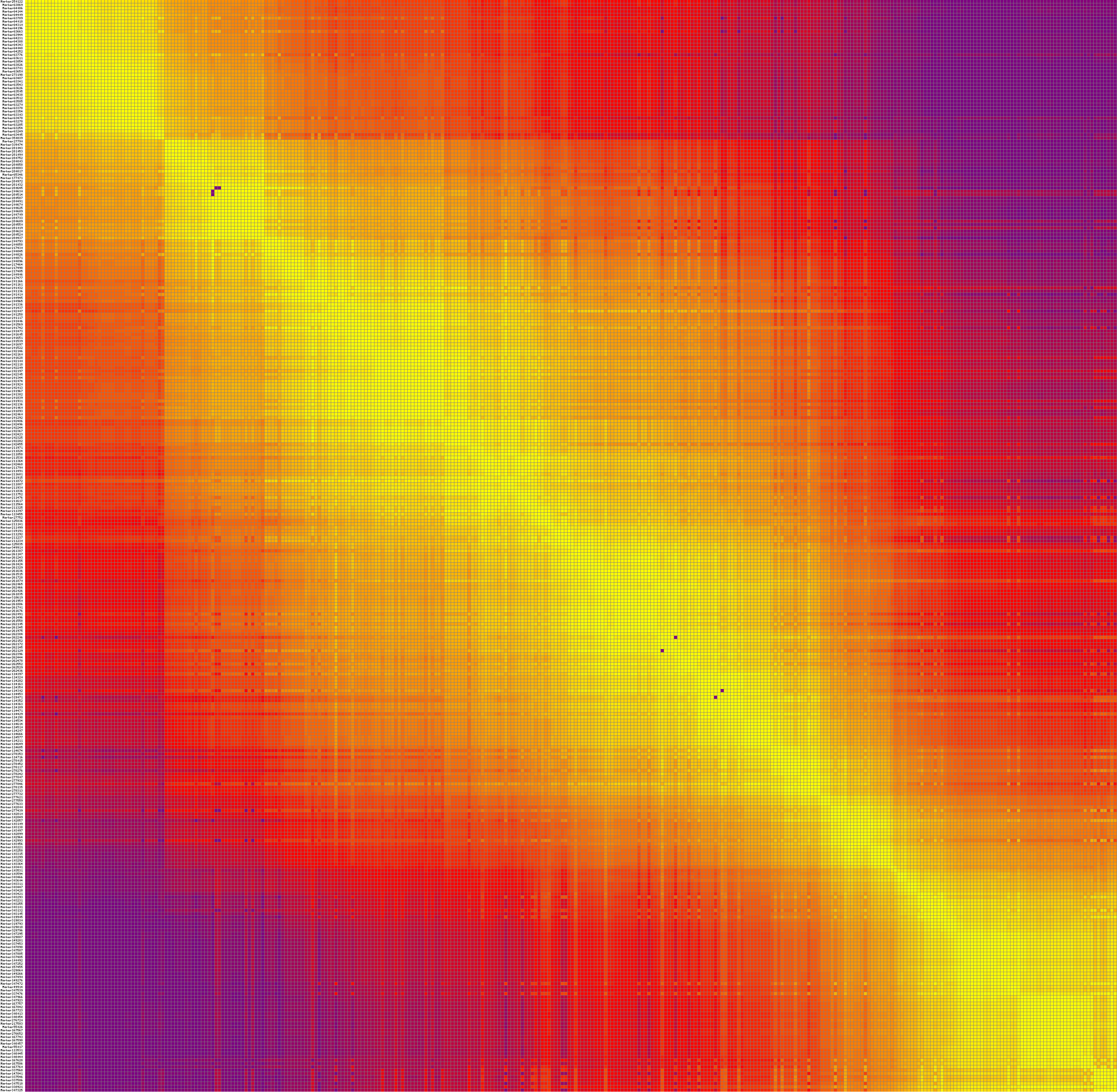

Supplement: Supplementary file 2 [file DataSheet_2.zip › Figure S6/female/LG13.female.heatMap.png]

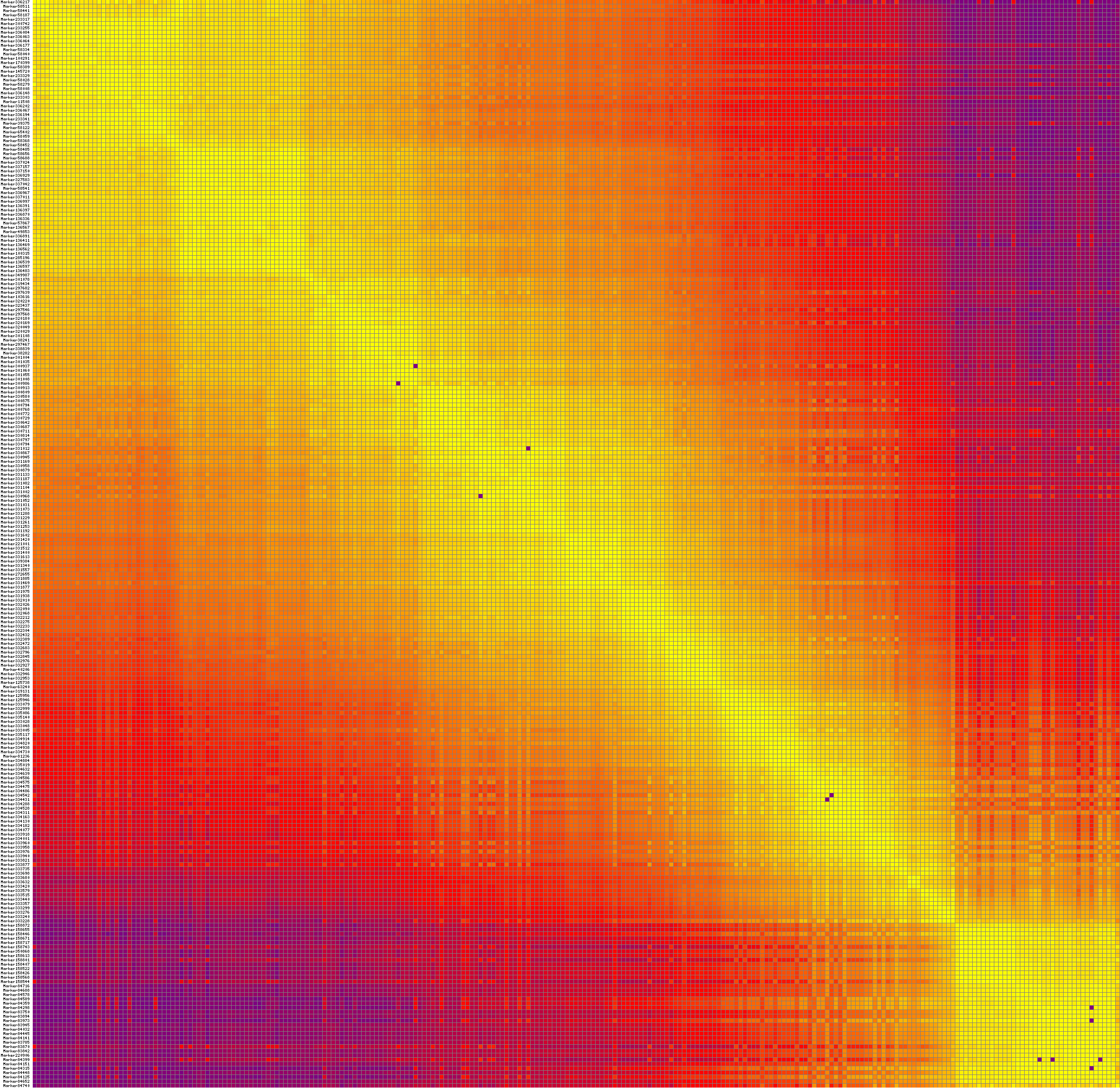

Supplement: Supplementary file 2 [file DataSheet_2.zip › Figure S6/female/LG14.female.heatMap.png]

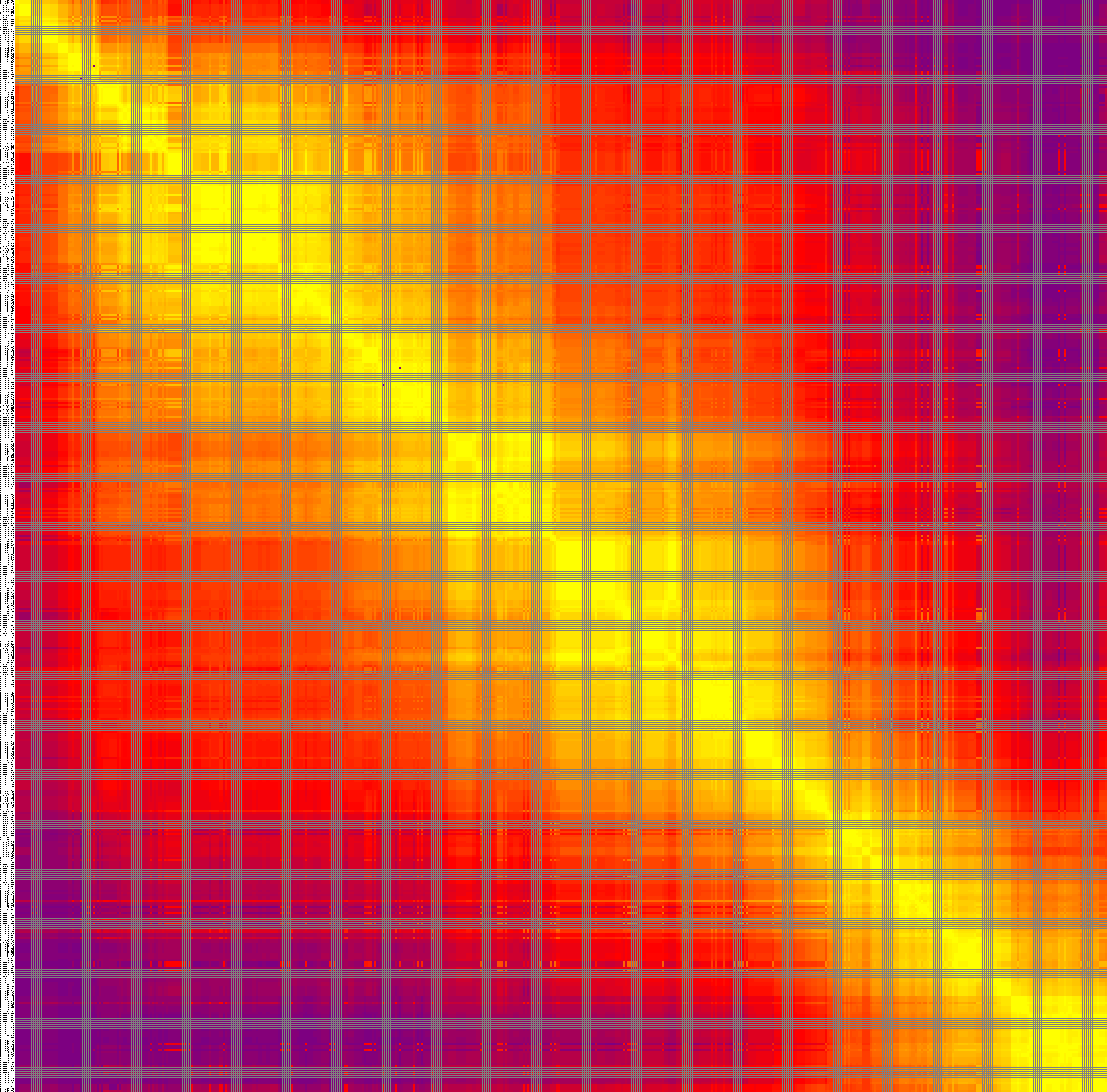

Supplement: Supplementary file 2 [file DataSheet_2.zip › Figure S6/female/LG15.female.heatMap.png]

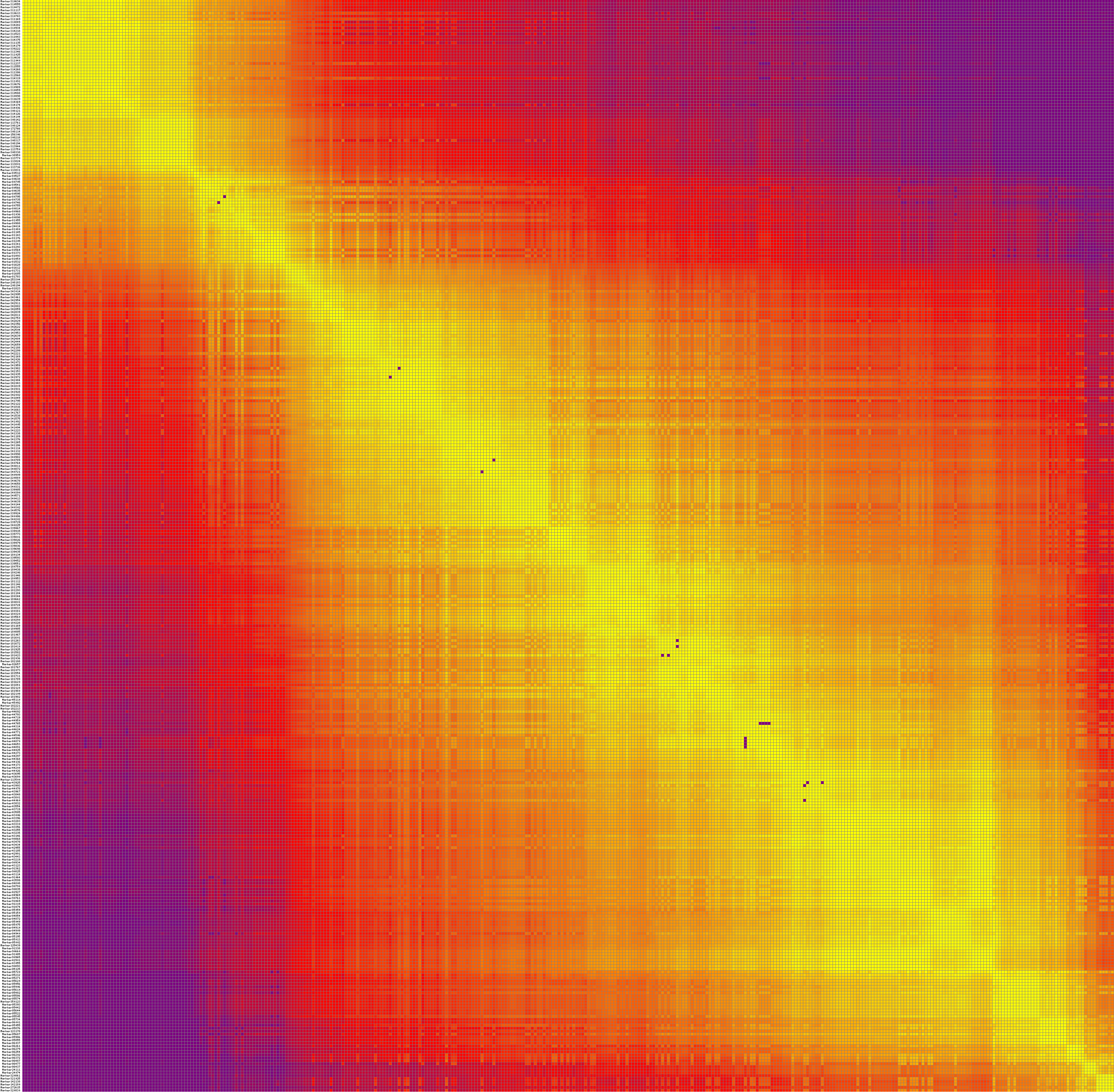

Supplement: Supplementary file 2 [file DataSheet_2.zip › Figure S6/female/LG16.female.heatMap.png]

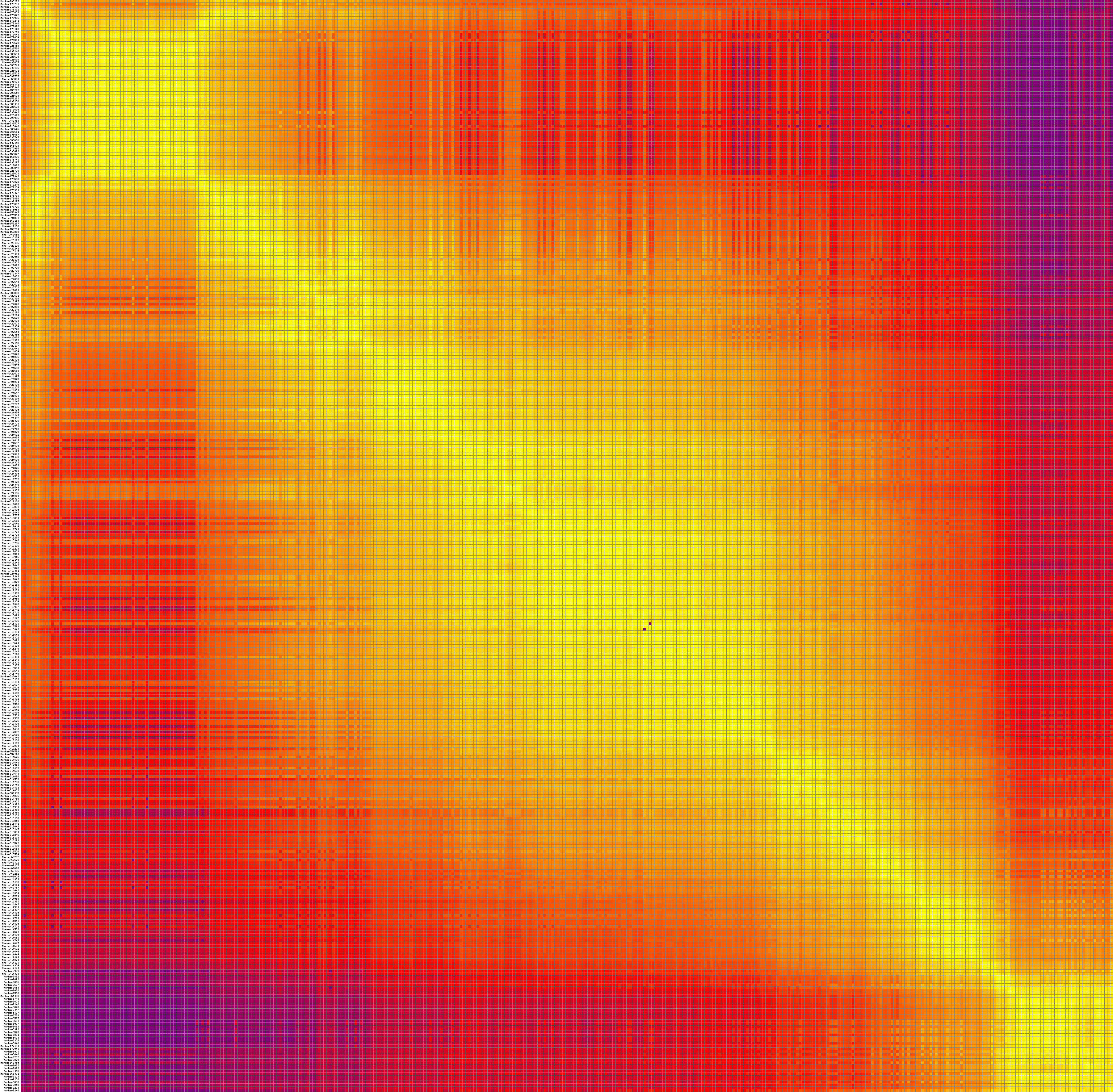

Supplement: Supplementary file 2 [file DataSheet_2.zip › Figure S6/female/LG17.female.heatMap.png]

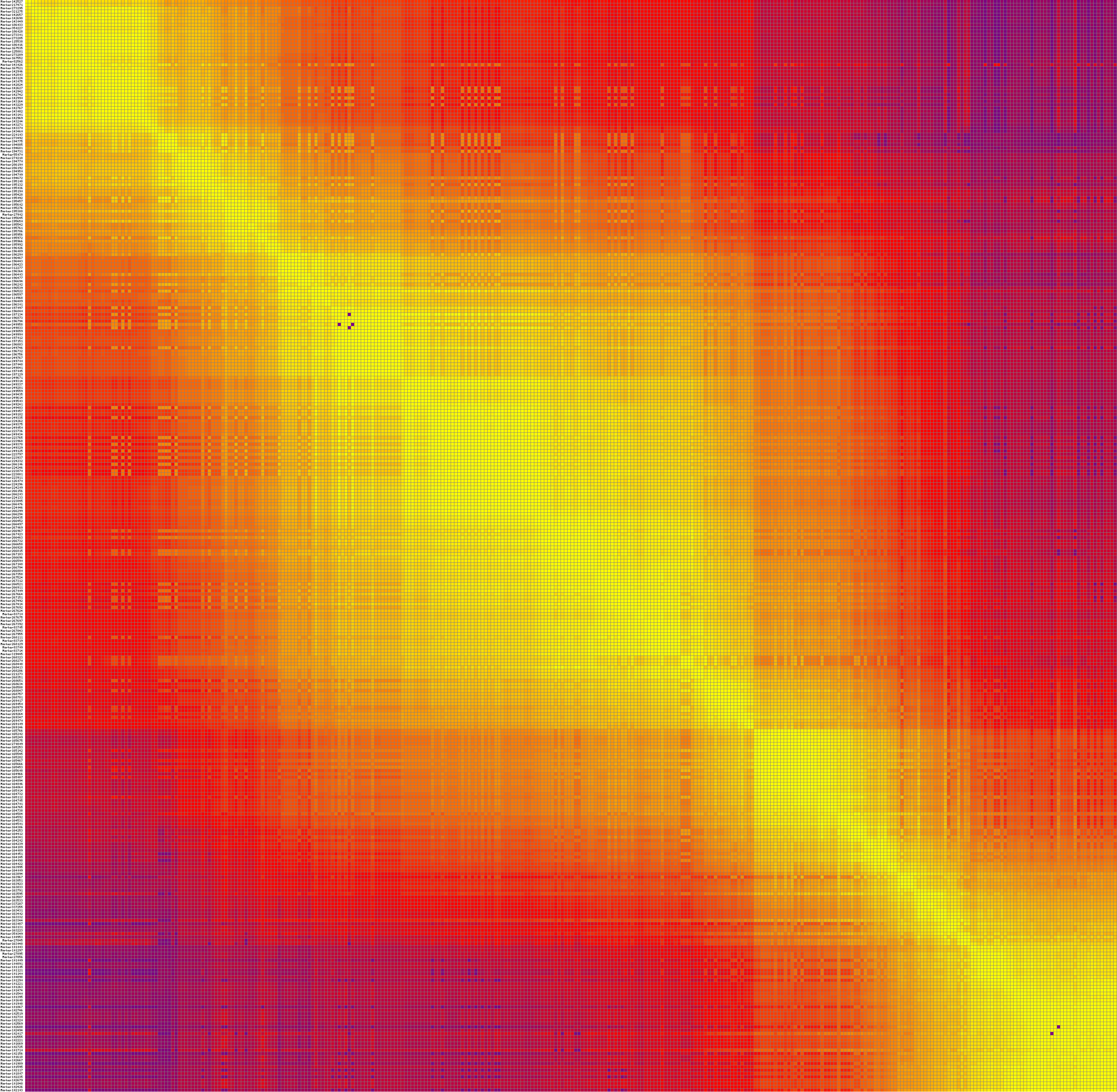

Supplement: Supplementary file 2 [file DataSheet_2.zip › Figure S6/female/LG18.female.heatMap.png]

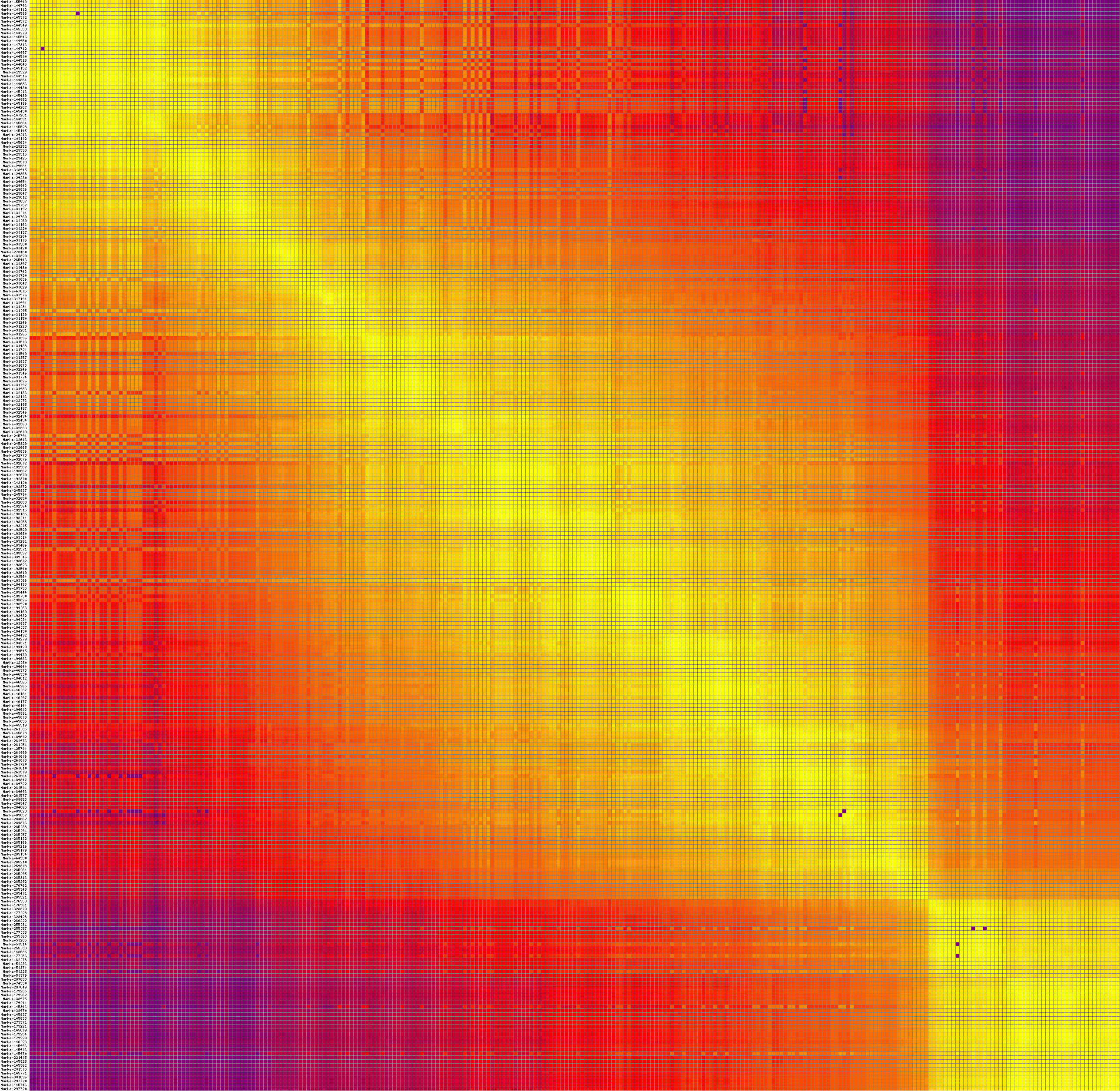

Supplement: Supplementary file 2 [file DataSheet_2.zip › Figure S6/female/LG19.female.heatMap.png]

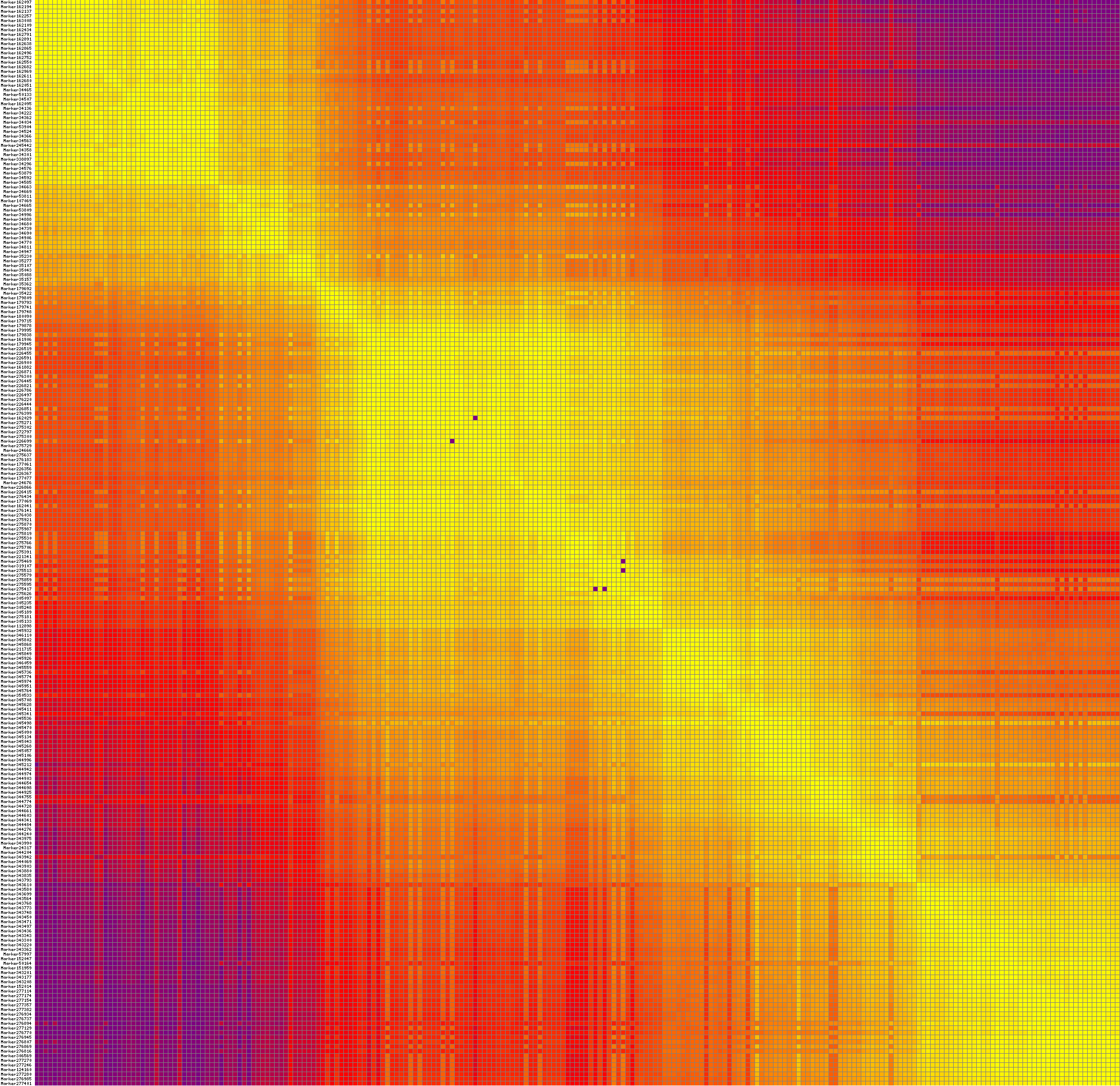

Supplement: Supplementary file 2 [file DataSheet_2.zip › Figure S6/female/LG2.female.heatMap.png]

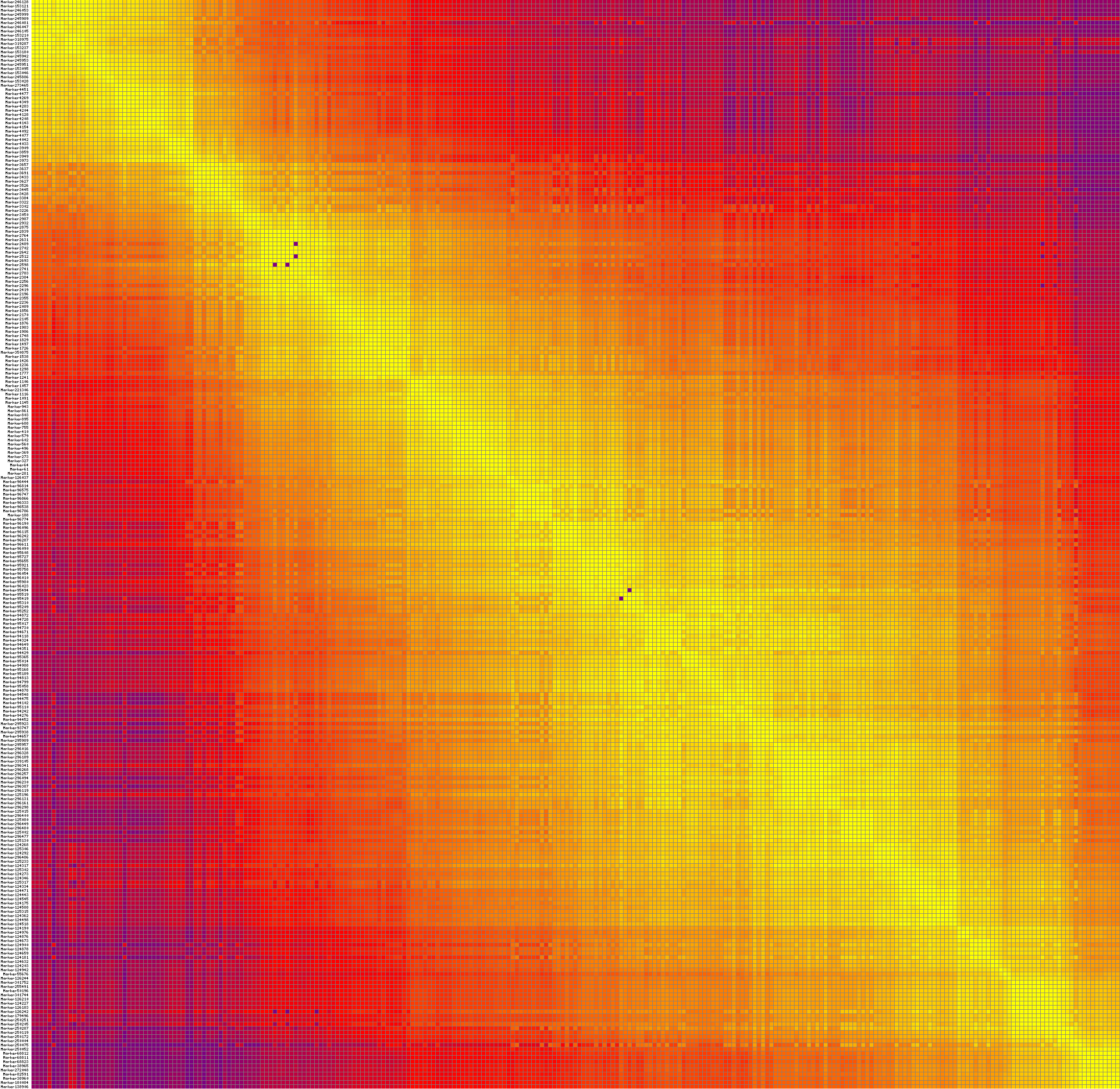

Supplement: Supplementary file 2 [file DataSheet_2.zip › Figure S6/female/LG20.female.heatMap.png]

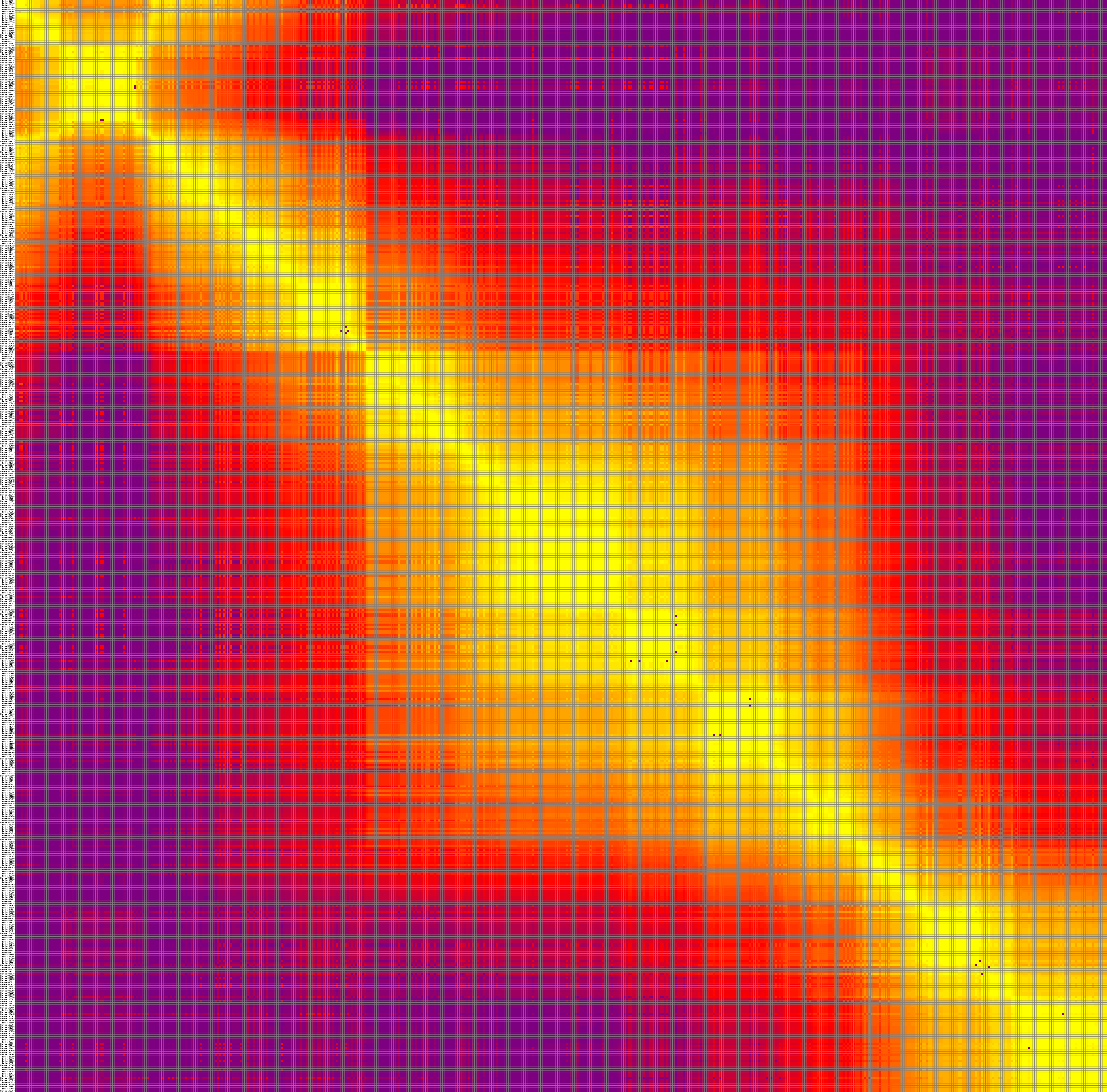

Supplement: Supplementary file 2 [file DataSheet_2.zip › Figure S6/female/LG21.female.heatMap.png]

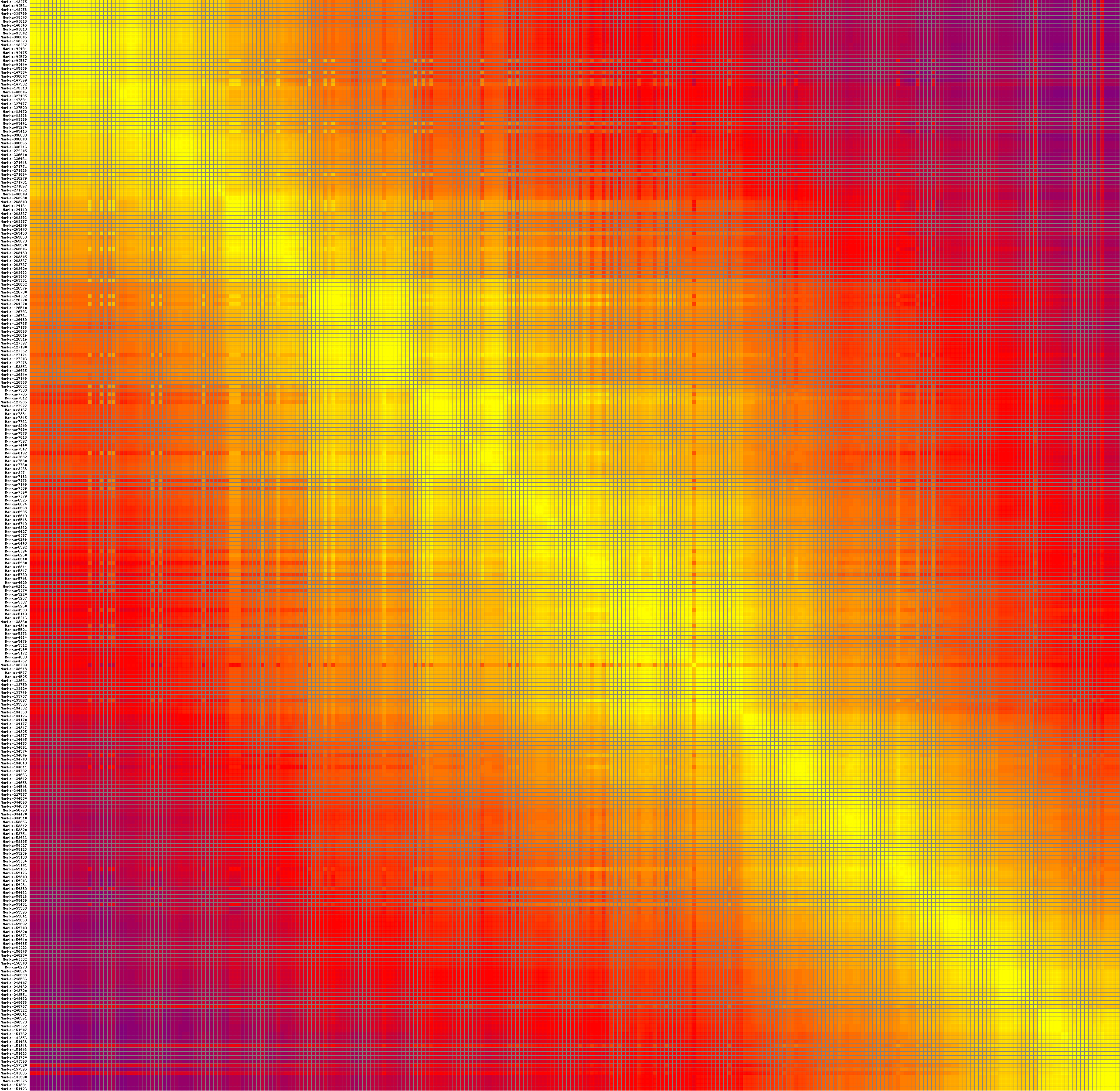

Supplement: Supplementary file 2 [file DataSheet_2.zip › Figure S6/female/LG22.female.heatMap.png]

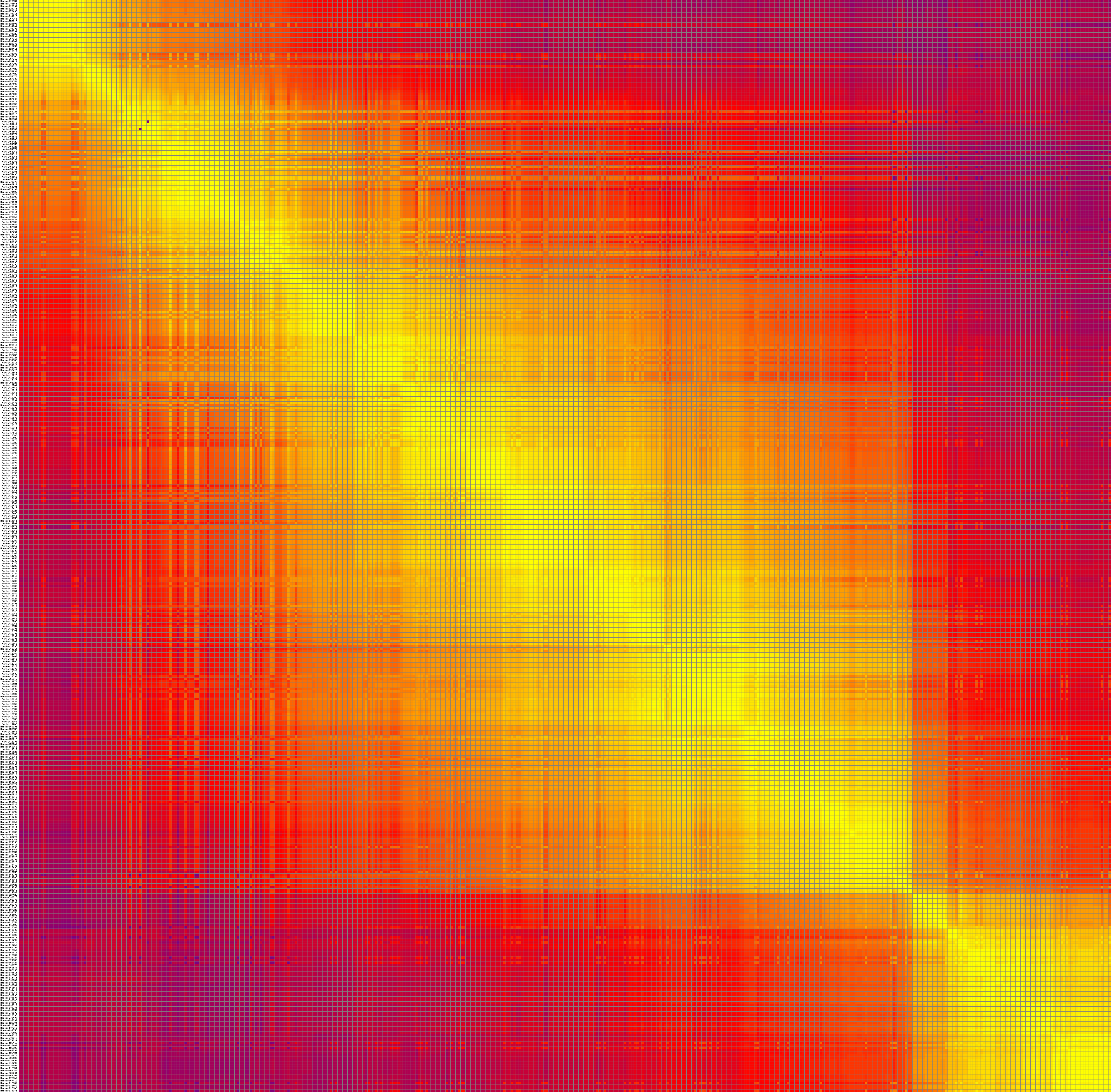

Supplement: Supplementary file 2 [file DataSheet_2.zip › Figure S6/female/LG23.female.heatMap.png]

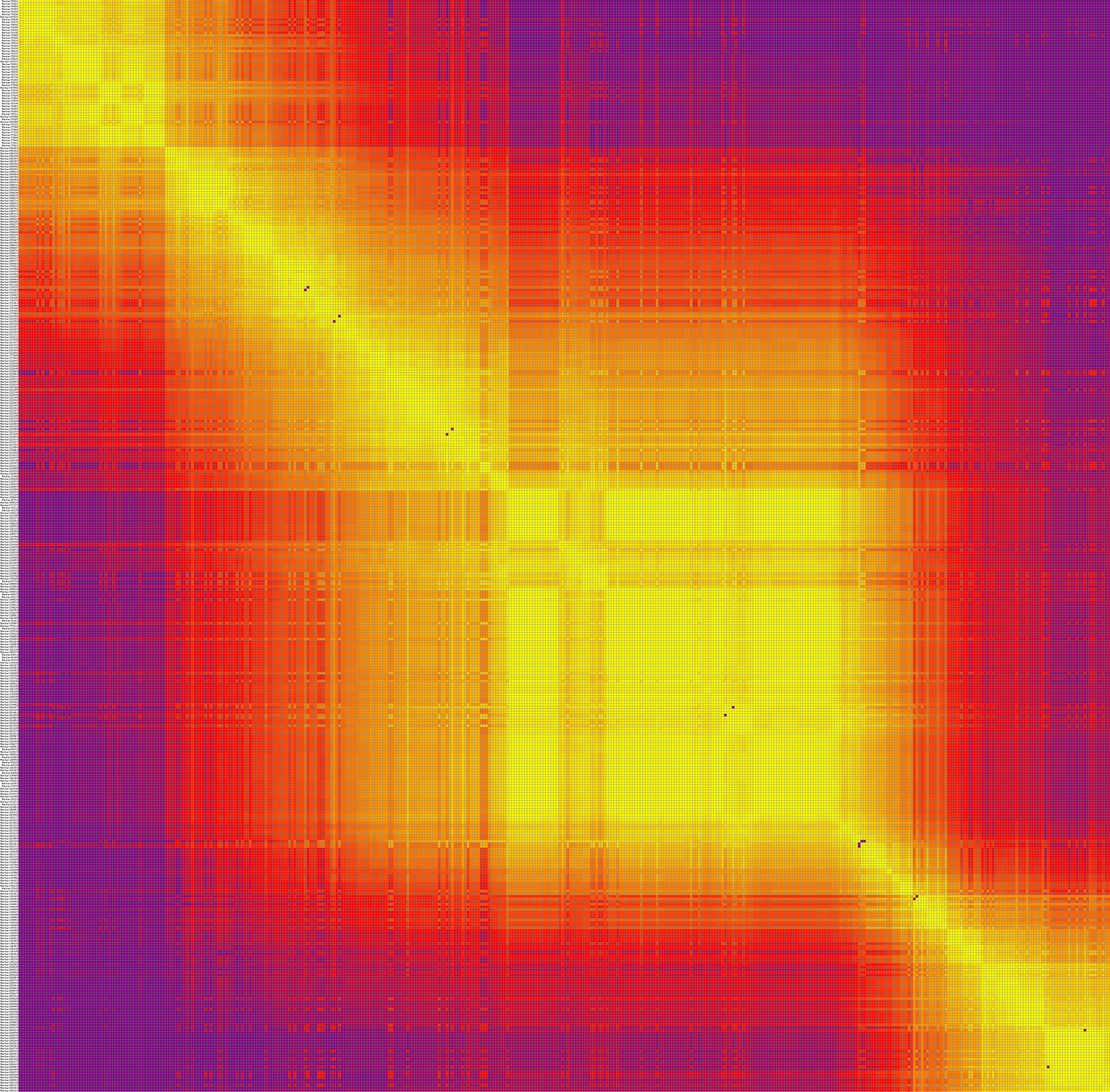

Supplement: Supplementary file 2 [file DataSheet_2.zip › Figure S6/female/LG24.female.heatMap.png]

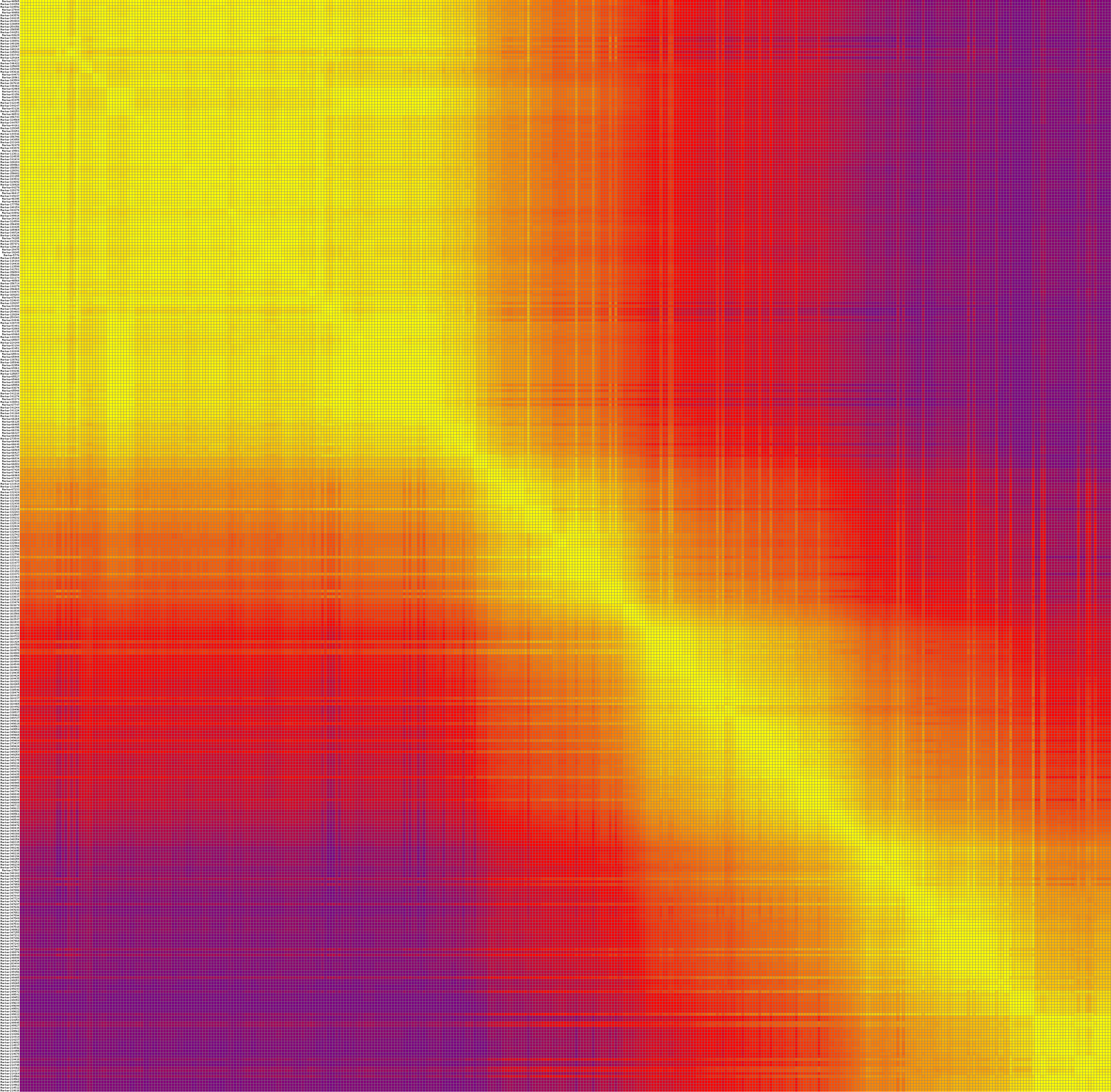

Supplement: Supplementary file 2 [file DataSheet_2.zip › Figure S6/female/LG3.female.heatMap.png]

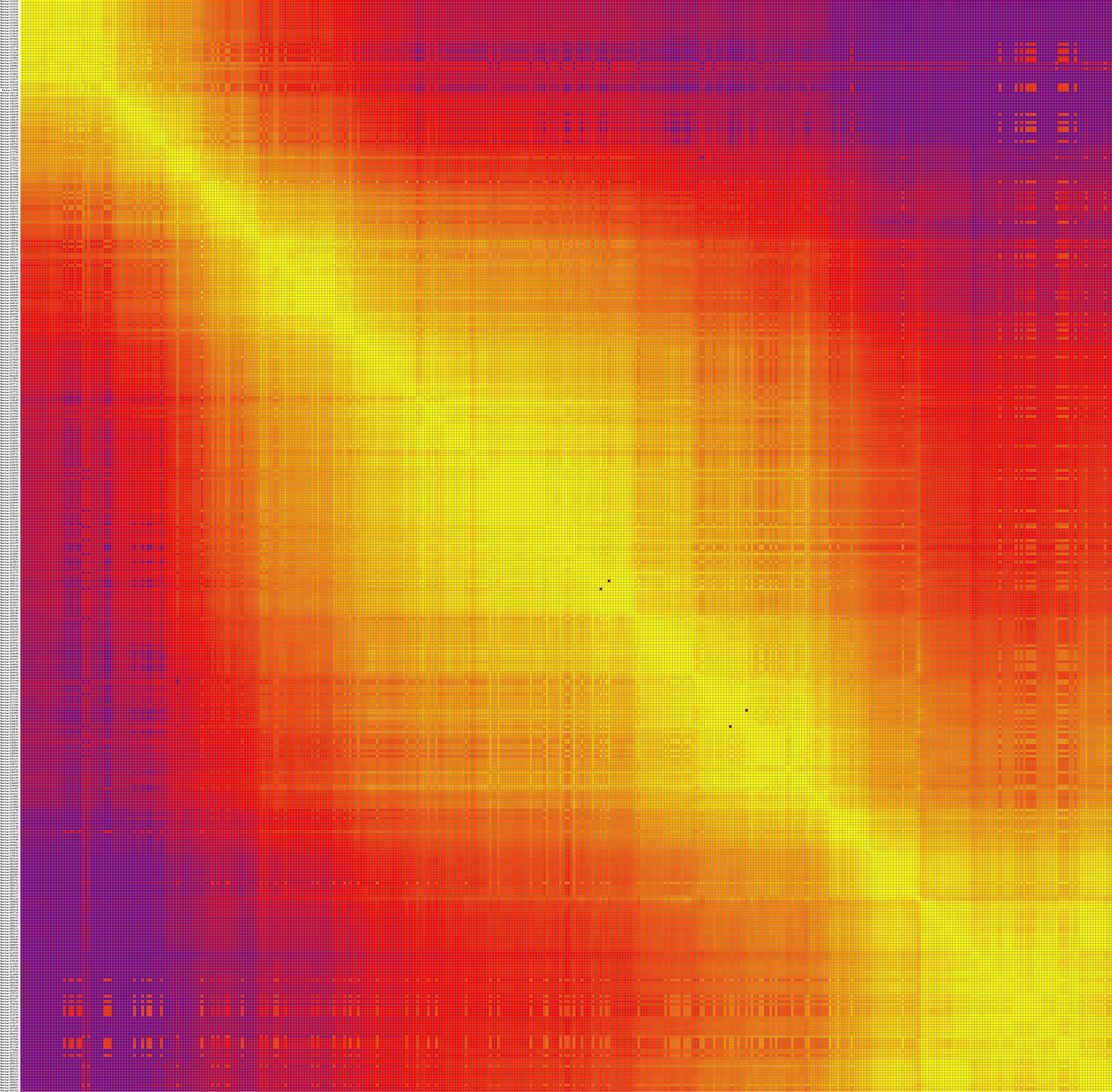

Supplement: Supplementary file 2 [file DataSheet_2.zip › Figure S6/female/LG4.female.heatMap.png]

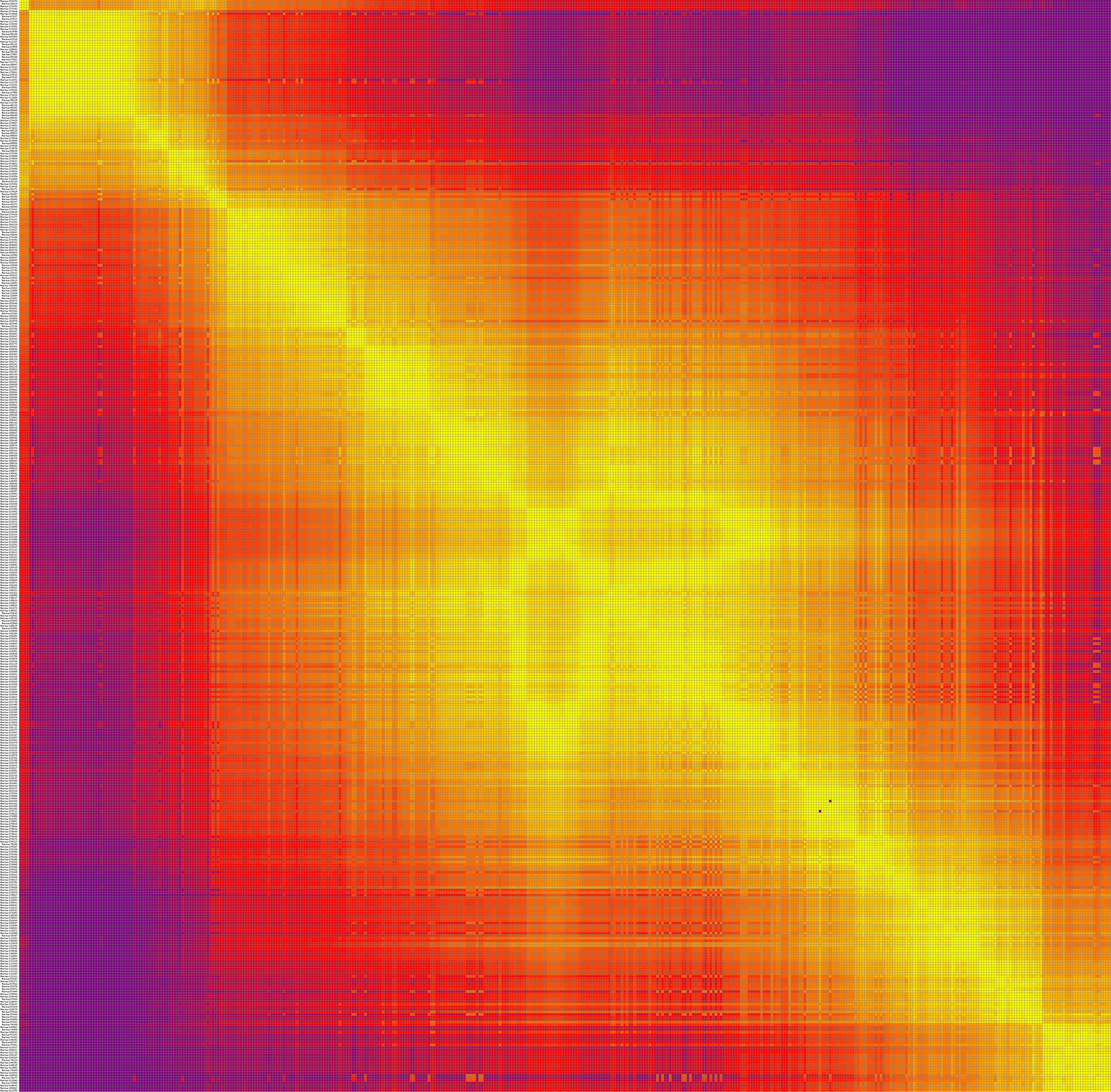

Supplement: Supplementary file 2 [file DataSheet_2.zip › Figure S6/female/LG5.female.heatMap.png]

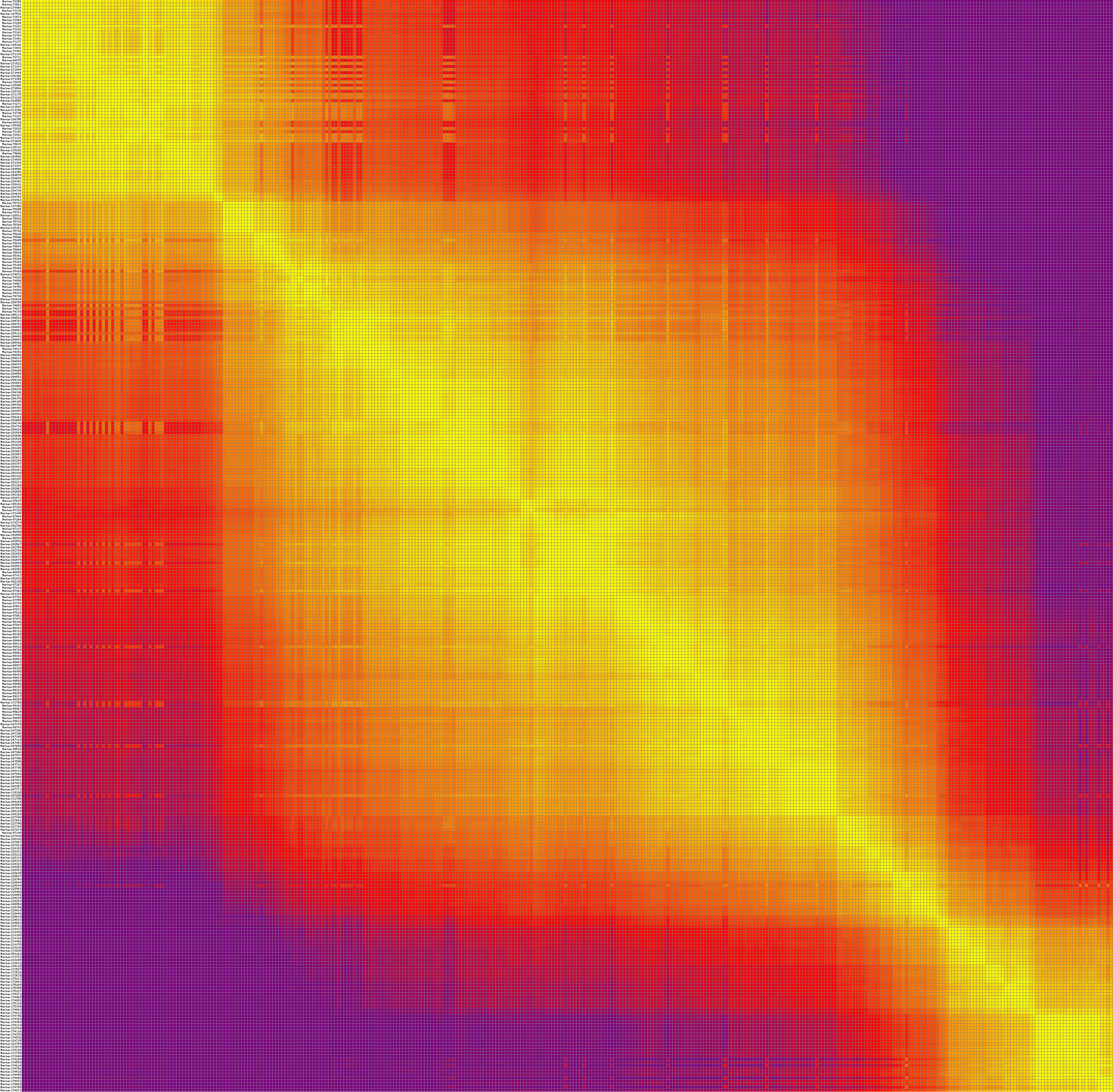

Supplement: Supplementary file 2 [file DataSheet_2.zip › Figure S6/female/LG6.female.heatMap.png]

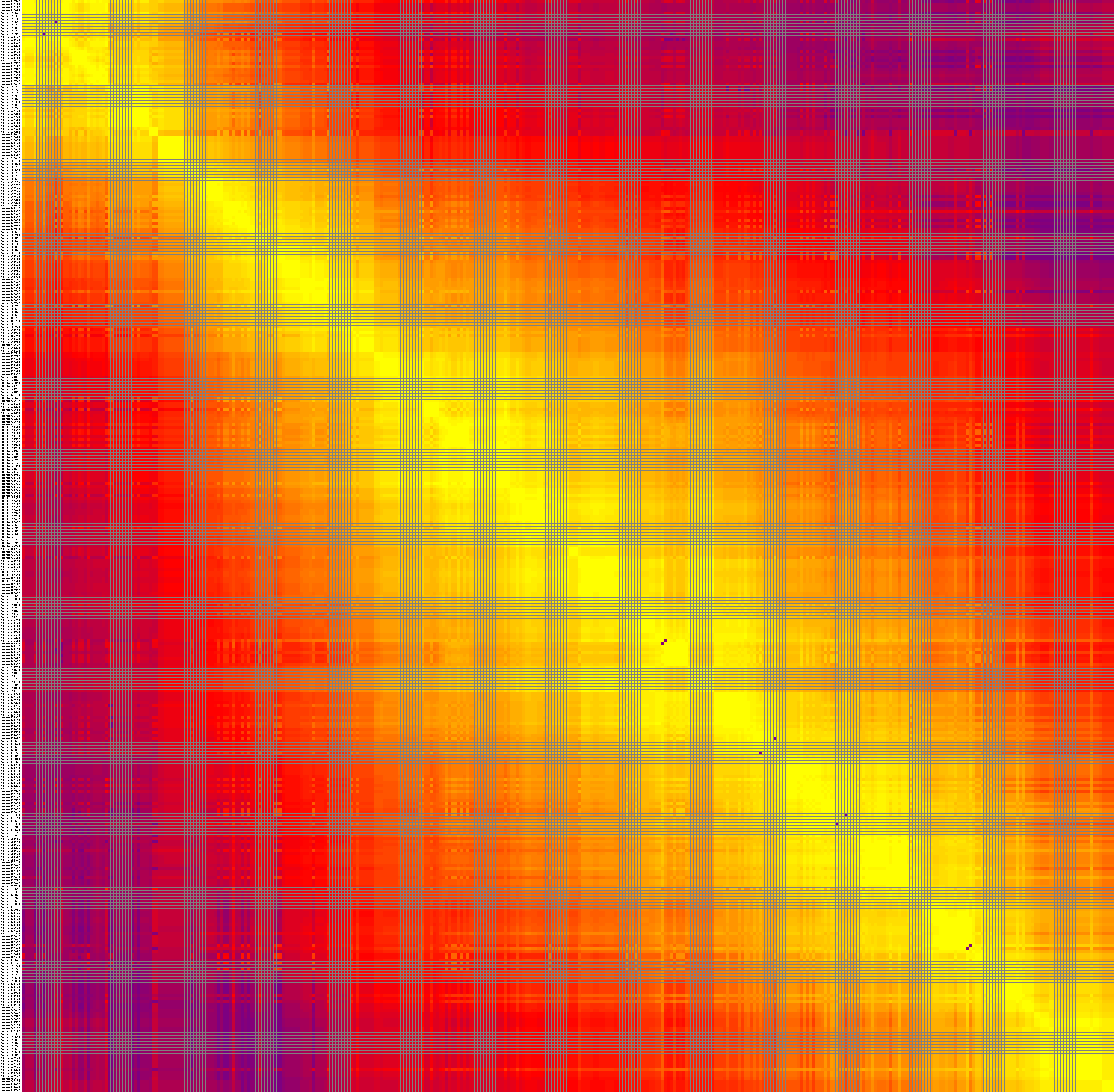

Supplement: Supplementary file 2 [file DataSheet_2.zip › Figure S6/female/LG7.female.heatMap.png]

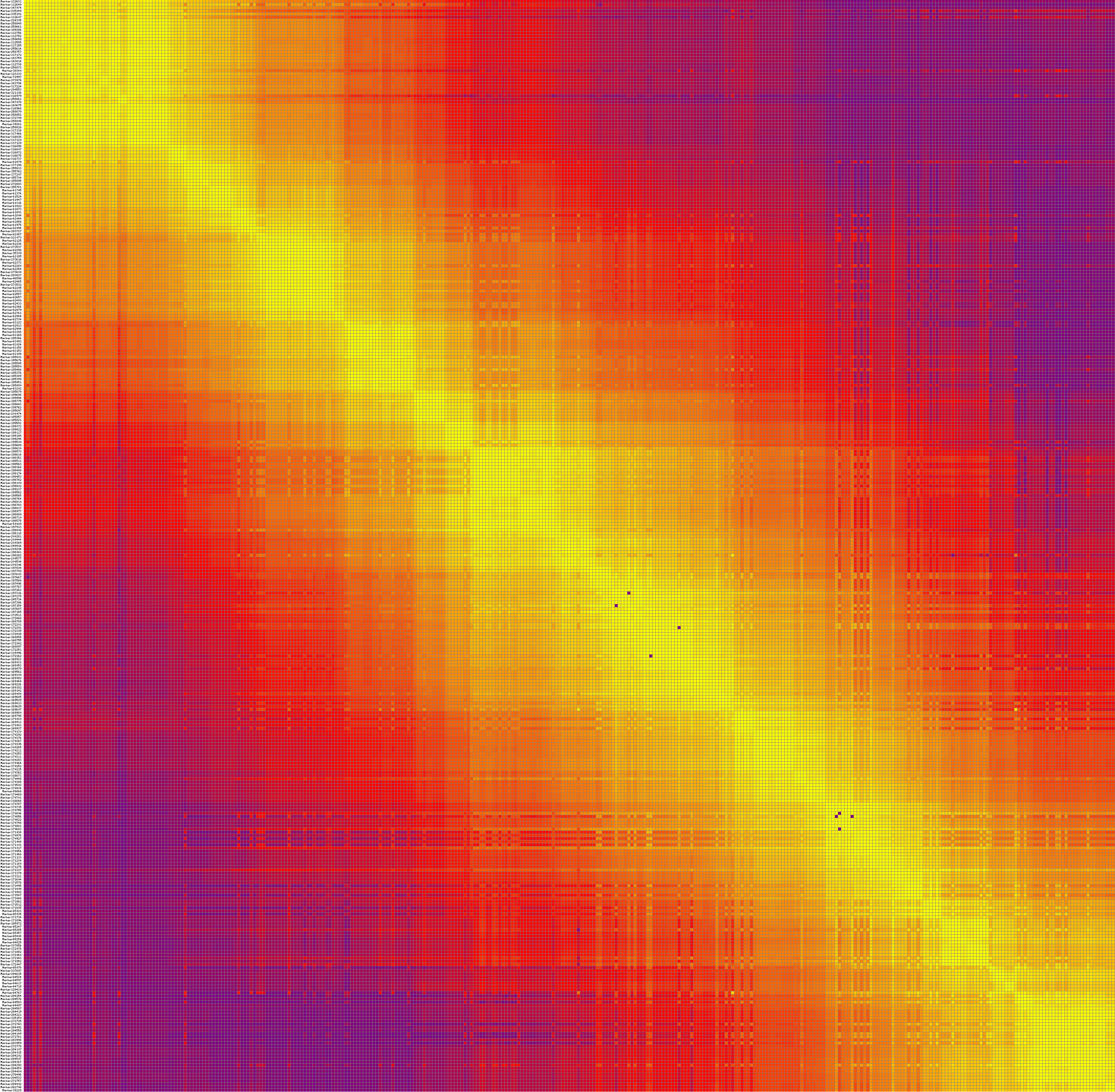

Supplement: Supplementary file 2 [file DataSheet_2.zip › Figure S6/female/LG8.female.heatMap.png]

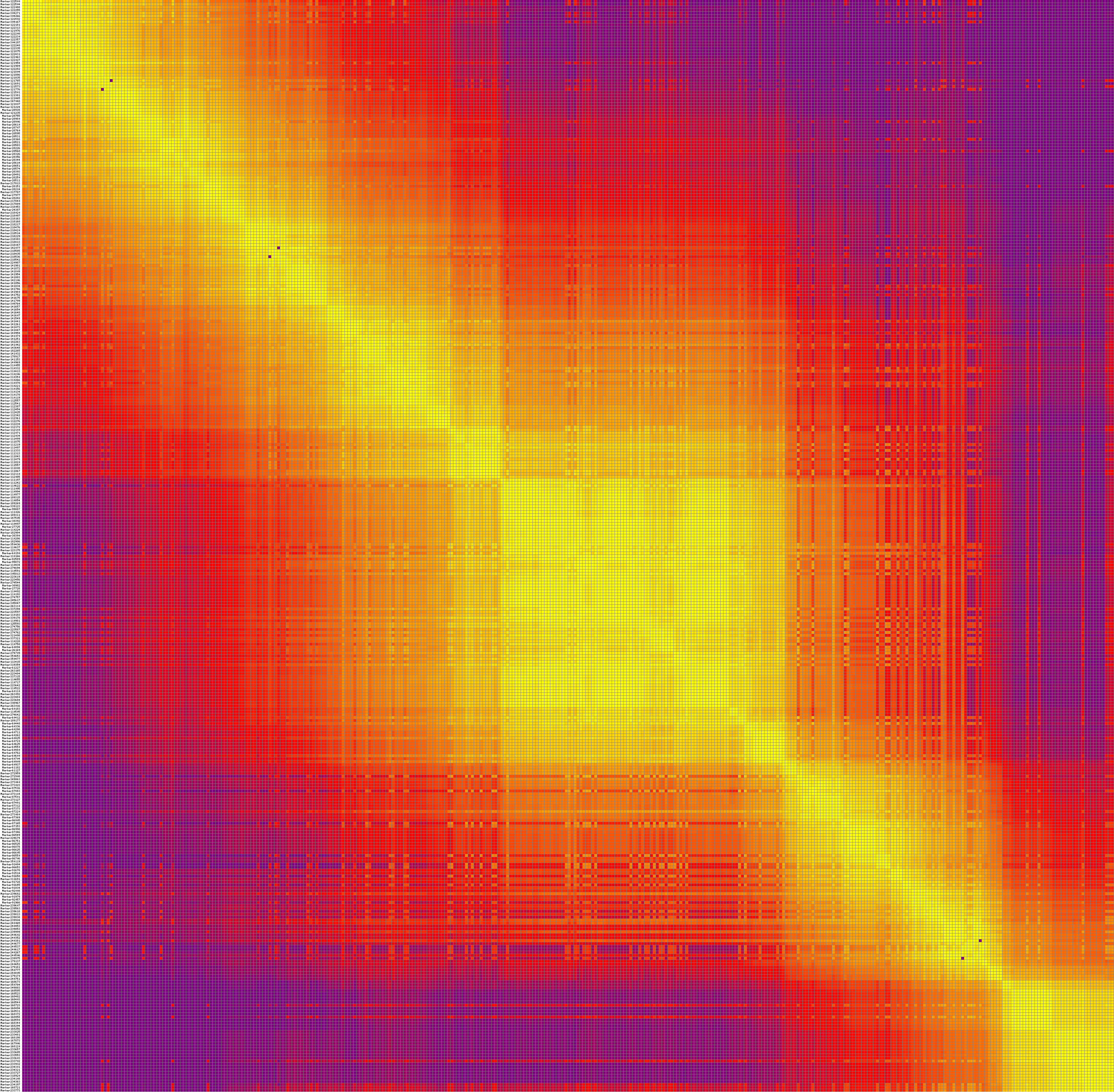

Supplement: Supplementary file 2 [file DataSheet_2.zip › Figure S6/female/LG9.female.heatMap.png]

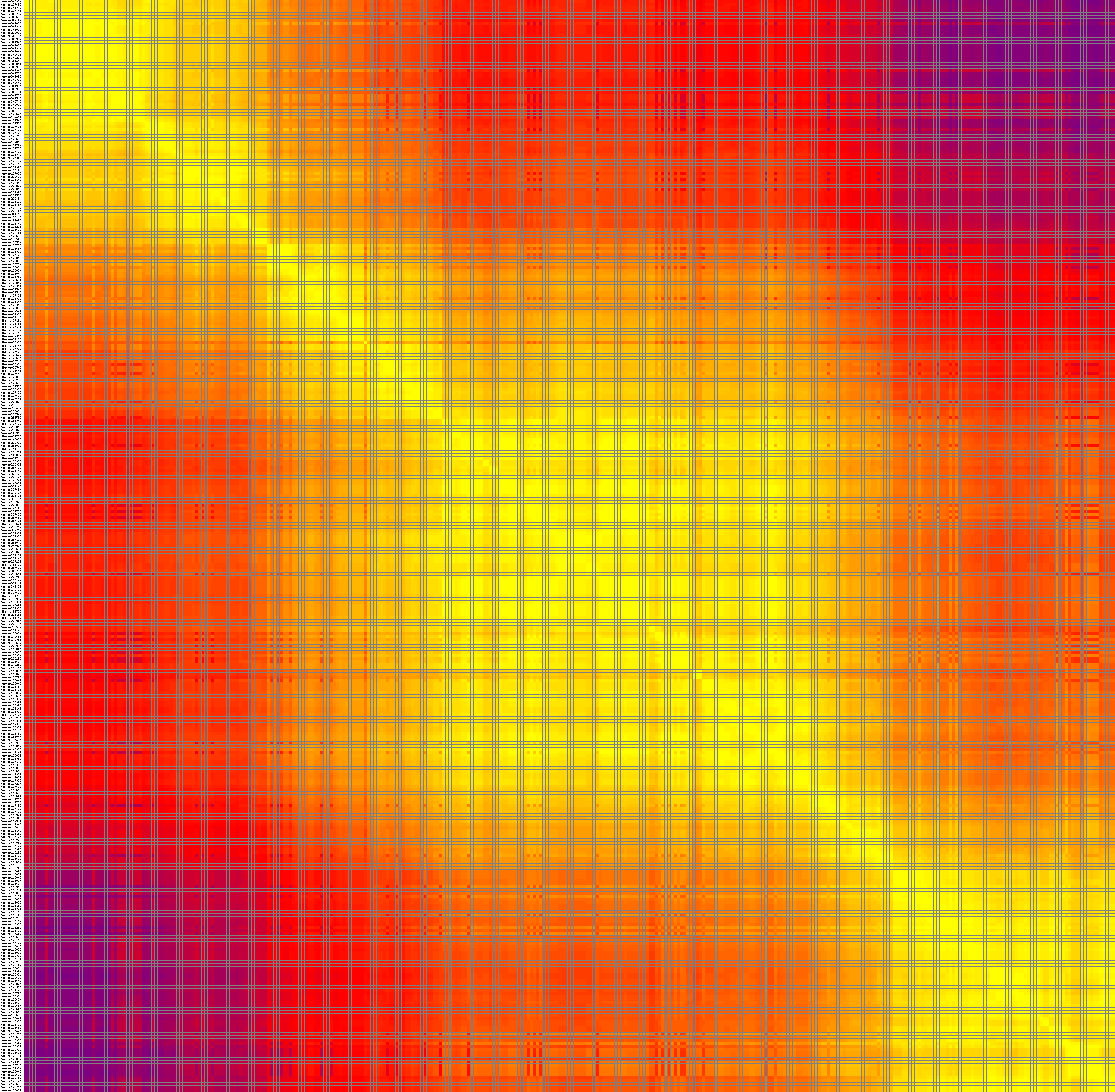

Supplement: Supplementary file 2 [file DataSheet_2.zip › Figure S6/male/LG1.male.heatMap.png]

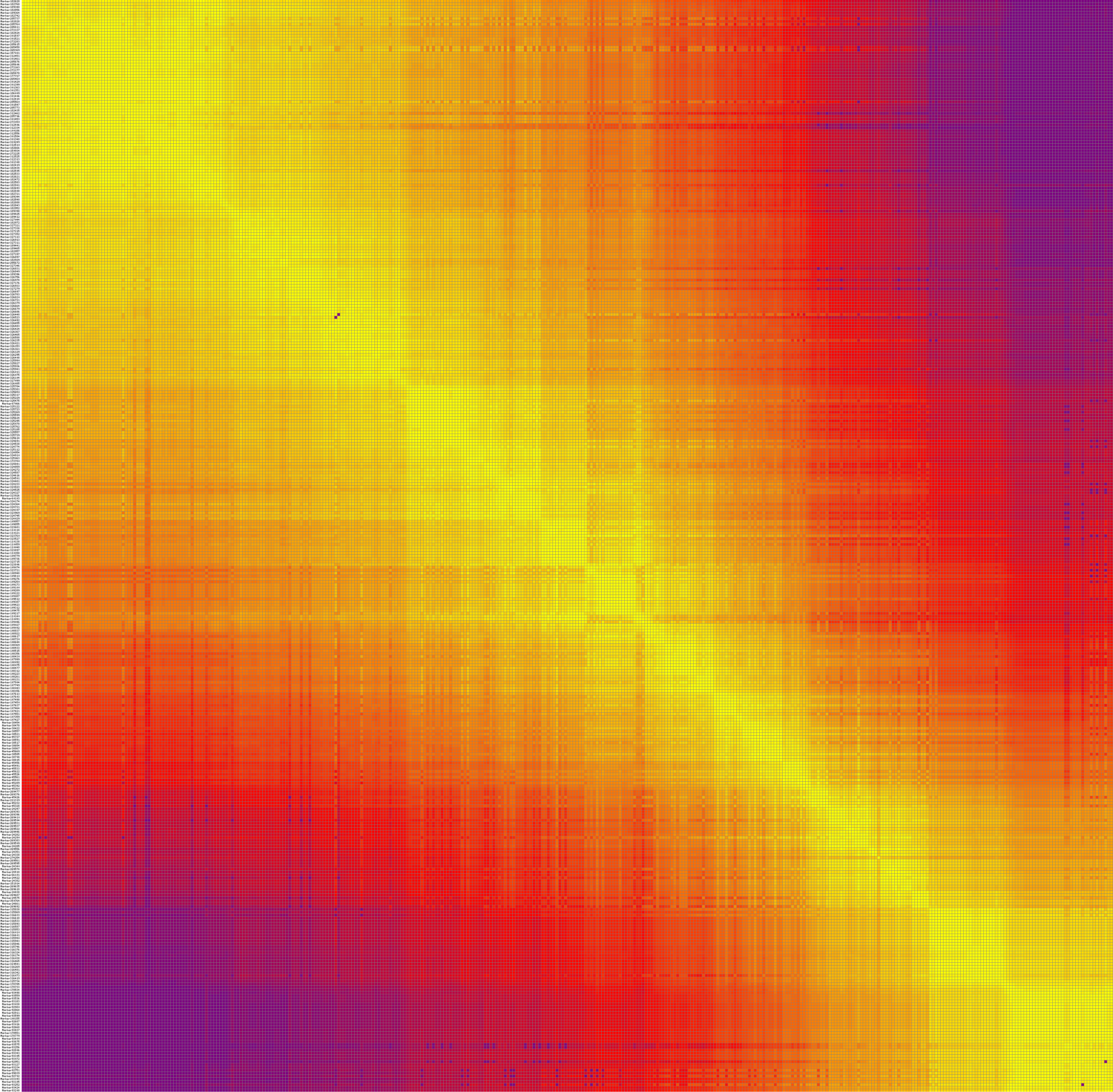

Supplement: Supplementary file 2 [file DataSheet_2.zip › Figure S6/male/LG10.male.heatMap.png]

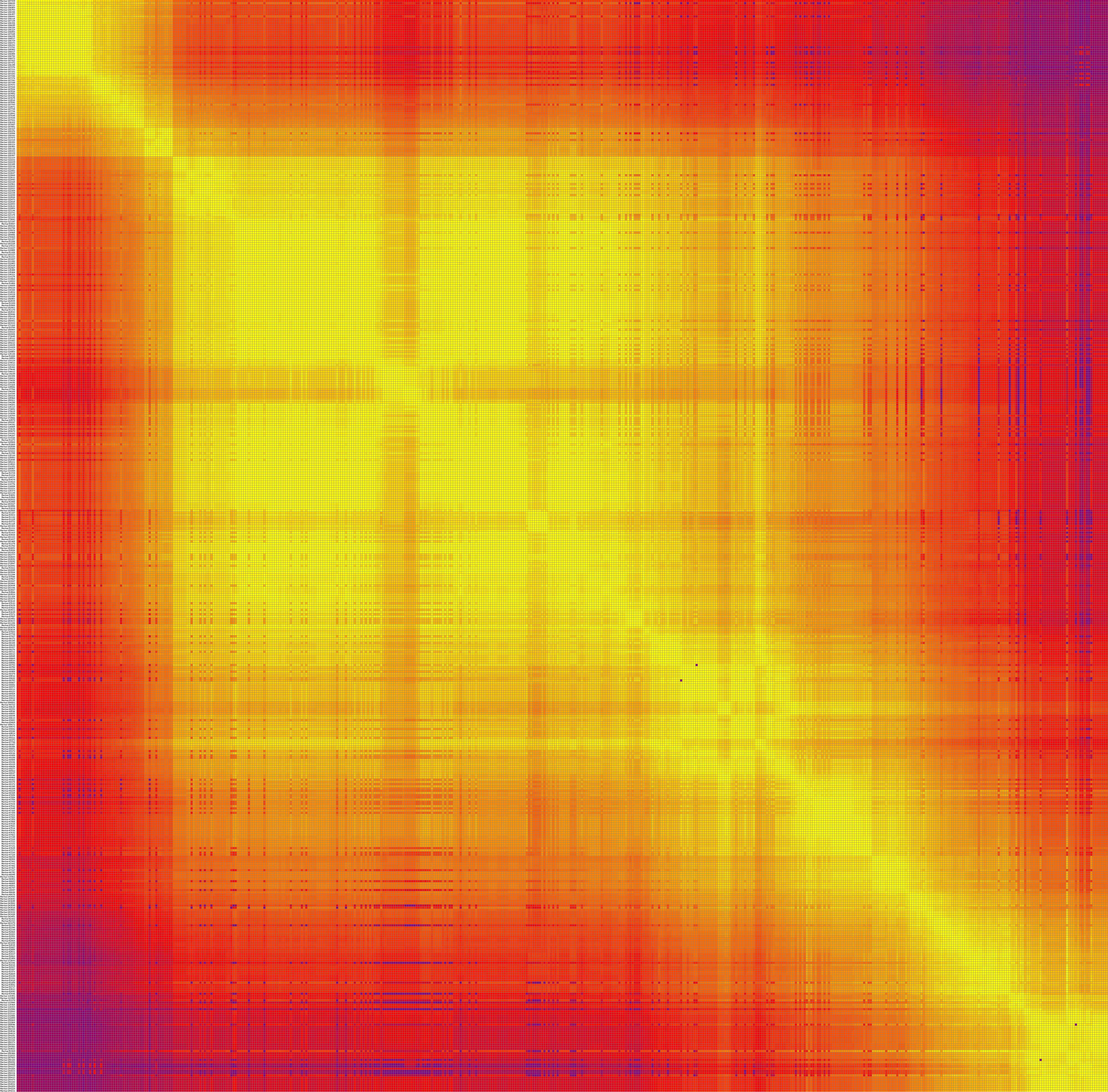

Supplement: Supplementary file 2 [file DataSheet_2.zip › Figure S6/male/LG11.male.heatMap.png]

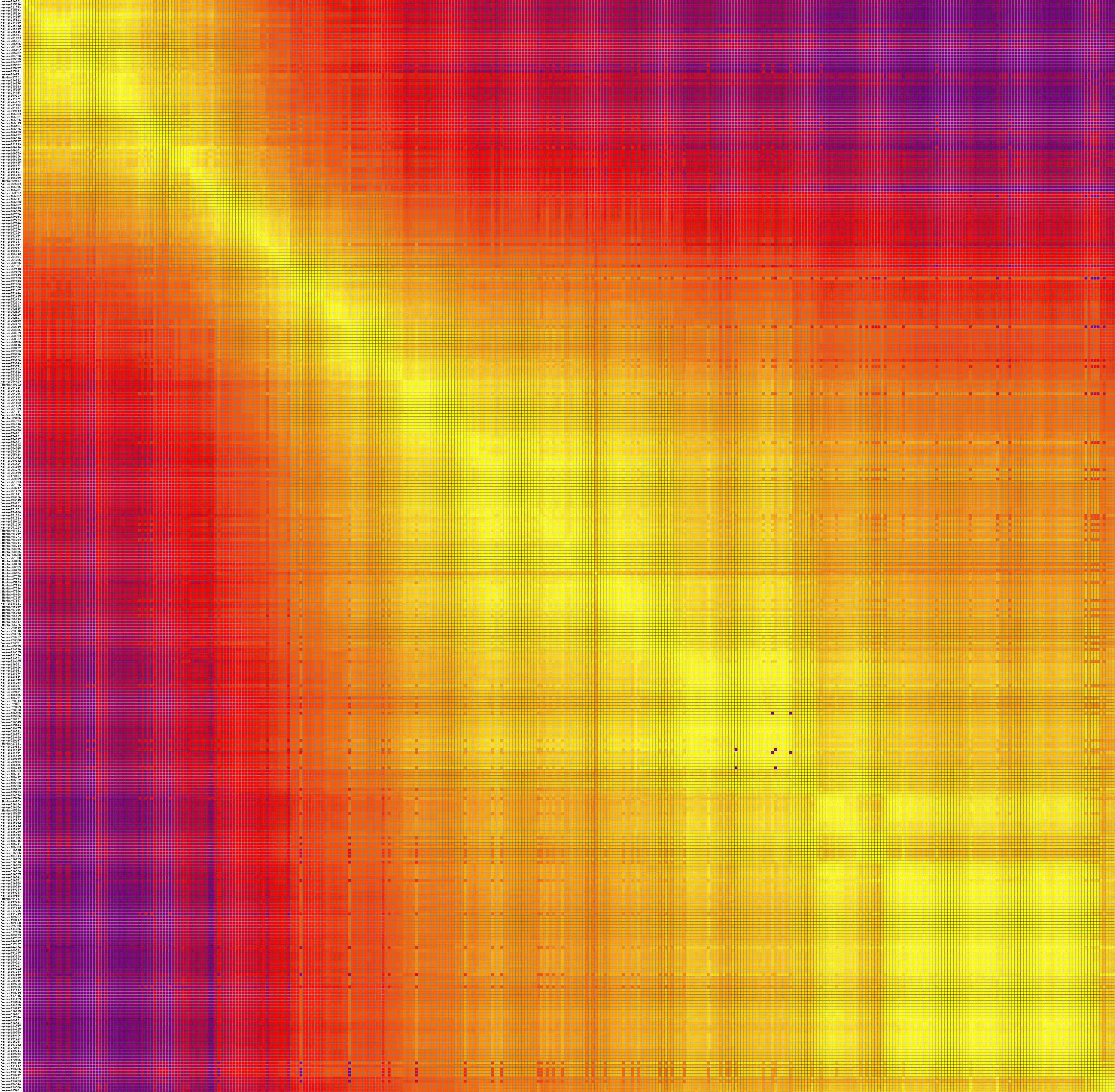

Supplement: Supplementary file 2 [file DataSheet_2.zip › Figure S6/male/LG12.male.heatMap.png]
