# Supplementary material for: A High-Density Genetic Linkage Map and QTL Mapping for Sex and Growth-Related Traits of Large-Scale Loach (Paramisgurnus dabryanus)
Source: Front Genet. 2019 Oct 25;10:1023. doi: 10.3389/fgene.2019.01023 (PMC6823184; doi:10.3389/fgene.2019.01023)
Supplement: Supplementary file 2 [file DataSheet_2.zip › Caption and Description of Figure S6.docx]

**File name:** Figure S6

**File format:** ZIP

**Caption of data:** Heat map showing a matrix of pair-wise recombination values for markers along LGs.

**Description of data:** The X-axis and Y-axis represent markers. The markers of each row and column were ordered according to the genetic map. The colors represent the strength of linkage in recombination values between all pairs of markers. The purple color indicates the highest recombination scores, which suggest no linkage between markers. The yellow color indicates the lowest recombination scores, which suggest a strong linkage between markers.
