# Supplementary figures and images for: A High-Density Genetic Linkage Map and QTL Mapping for Sex and Growth-Related Traits of Large-Scale Loach (Paramisgurnus dabryanus) (part 2 of 2)
Source: Front Genet. 2019 Oct 25;10:1023. doi: 10.3389/fgene.2019.01023 (PMC6823184; doi:10.3389/fgene.2019.01023)

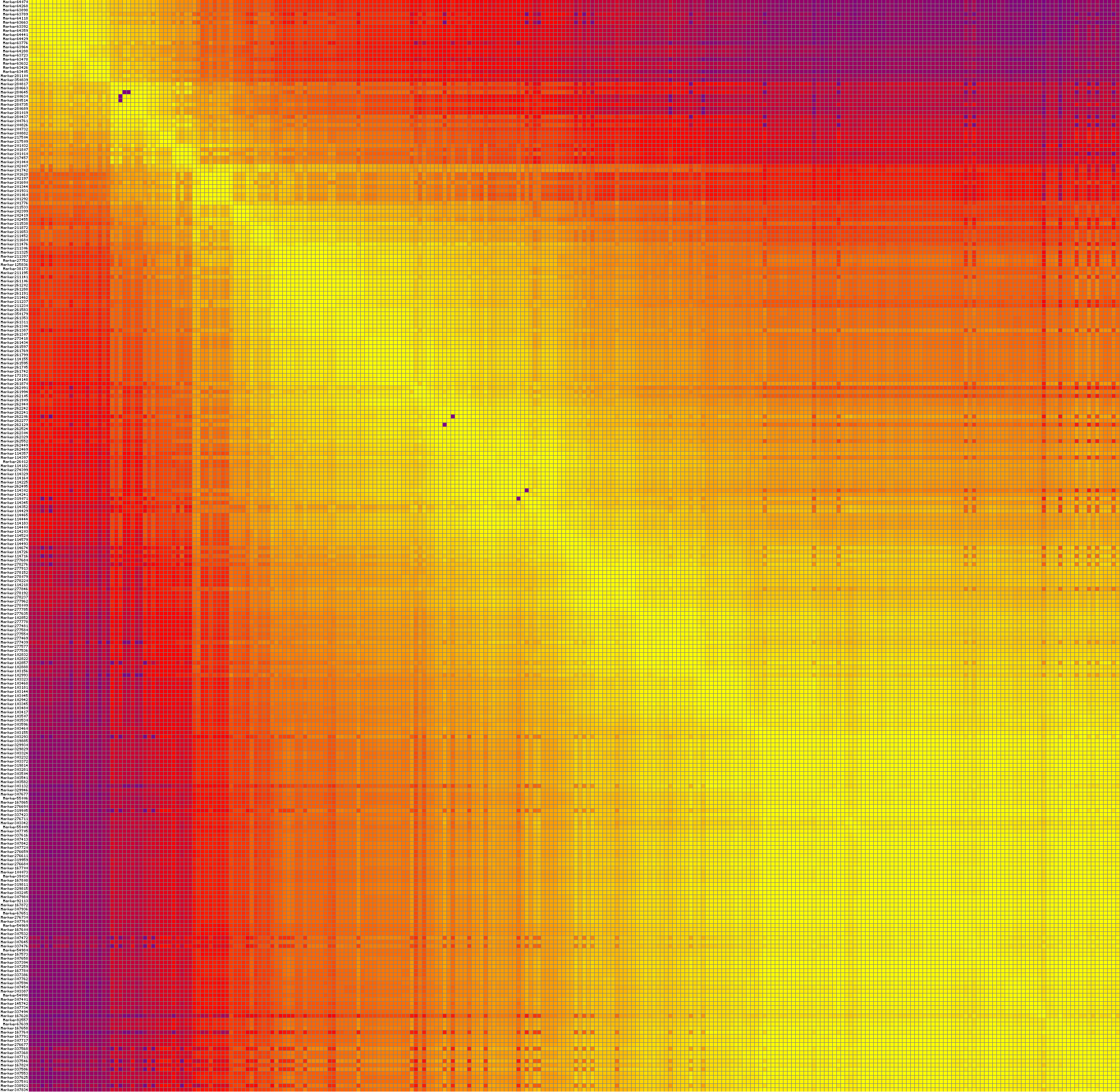

Supplement: Supplementary file 2 [file DataSheet_2.zip › Figure S6/male/LG13.male.heatMap.png]

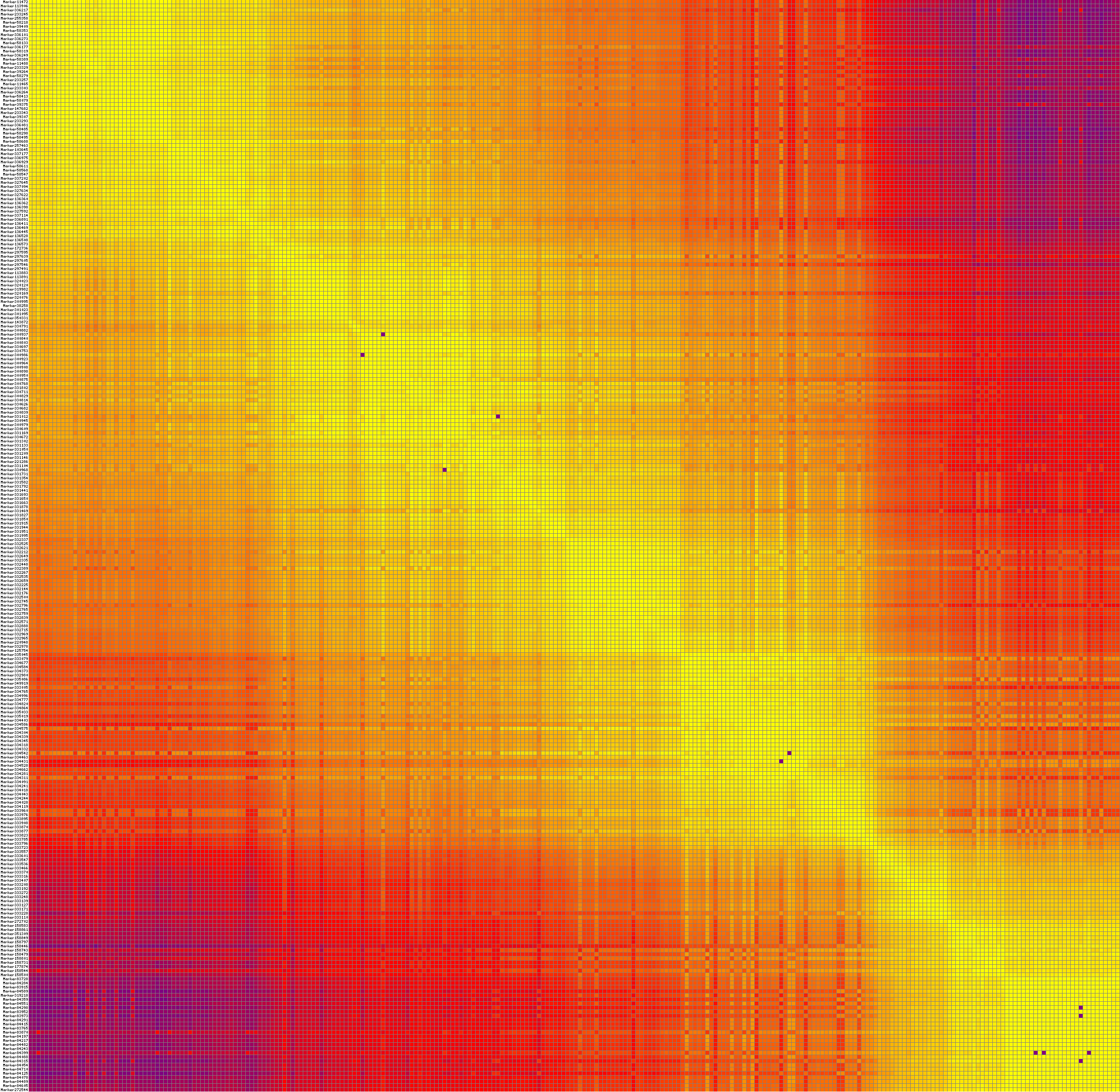

Supplement: Supplementary file 2 [file DataSheet_2.zip › Figure S6/male/LG14.male.heatMap.png]

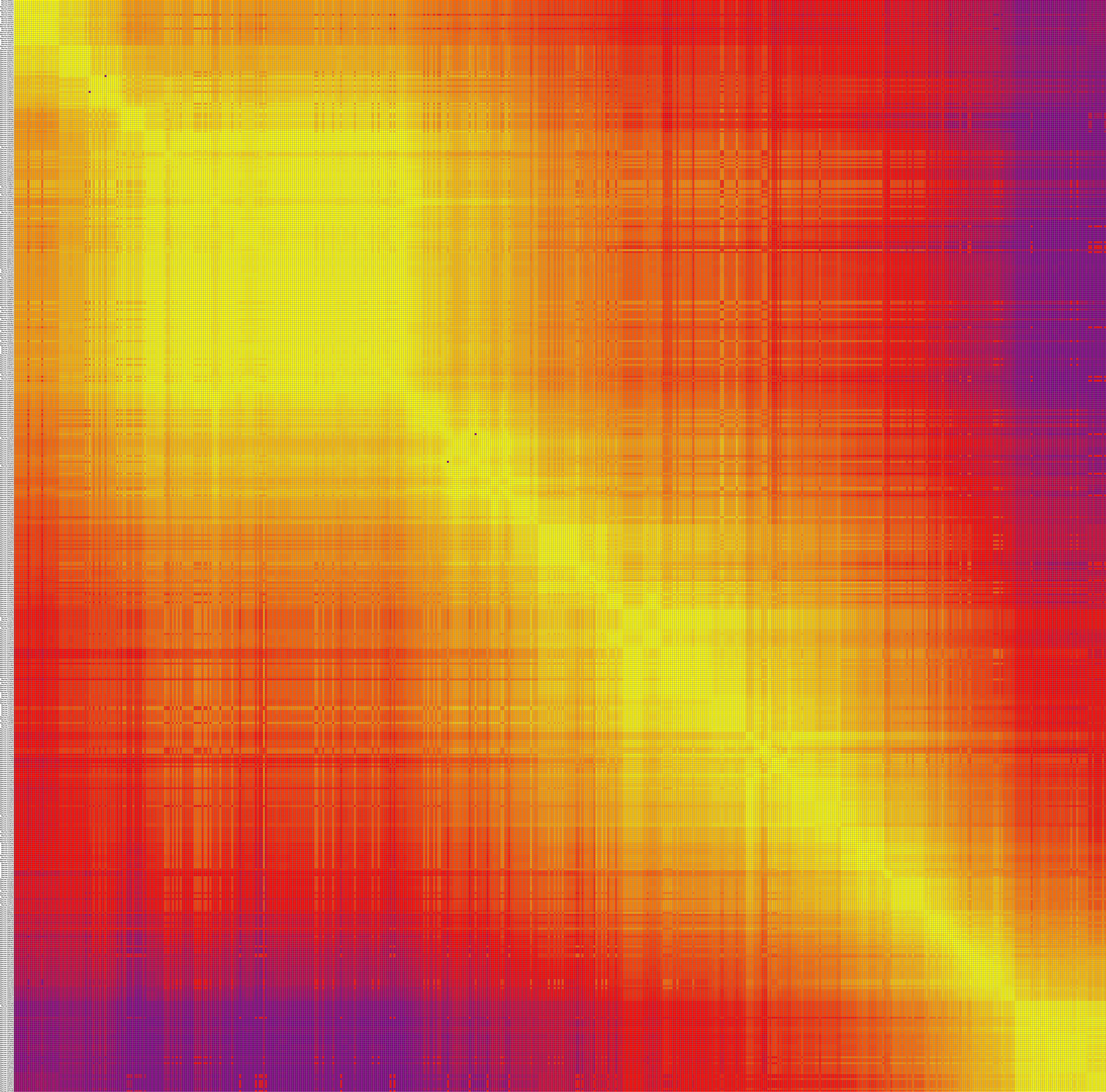

Supplement: Supplementary file 2 [file DataSheet_2.zip › Figure S6/male/LG15.male.heatMap.png]

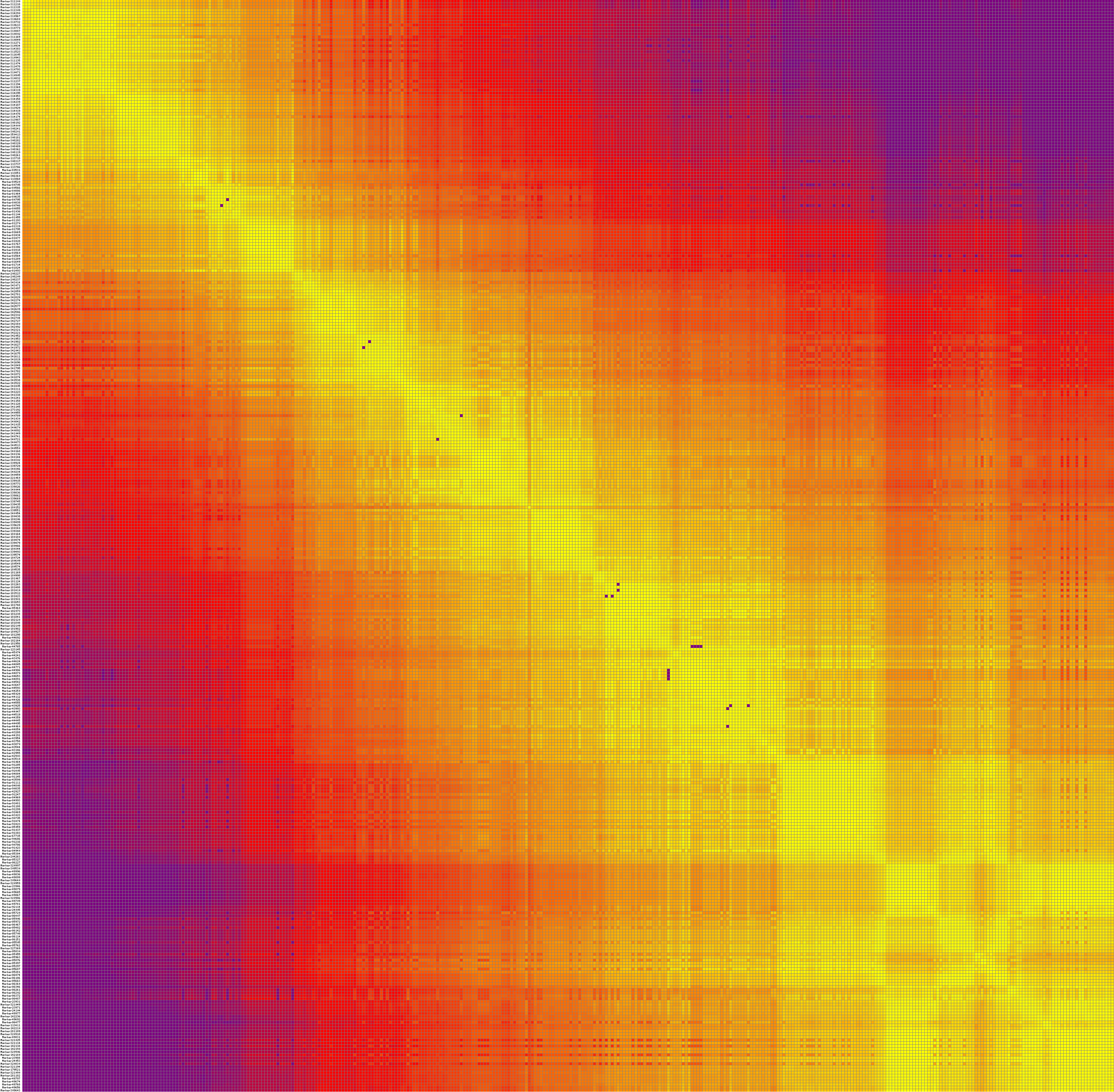

Supplement: Supplementary file 2 [file DataSheet_2.zip › Figure S6/male/LG16.male.heatMap.png]

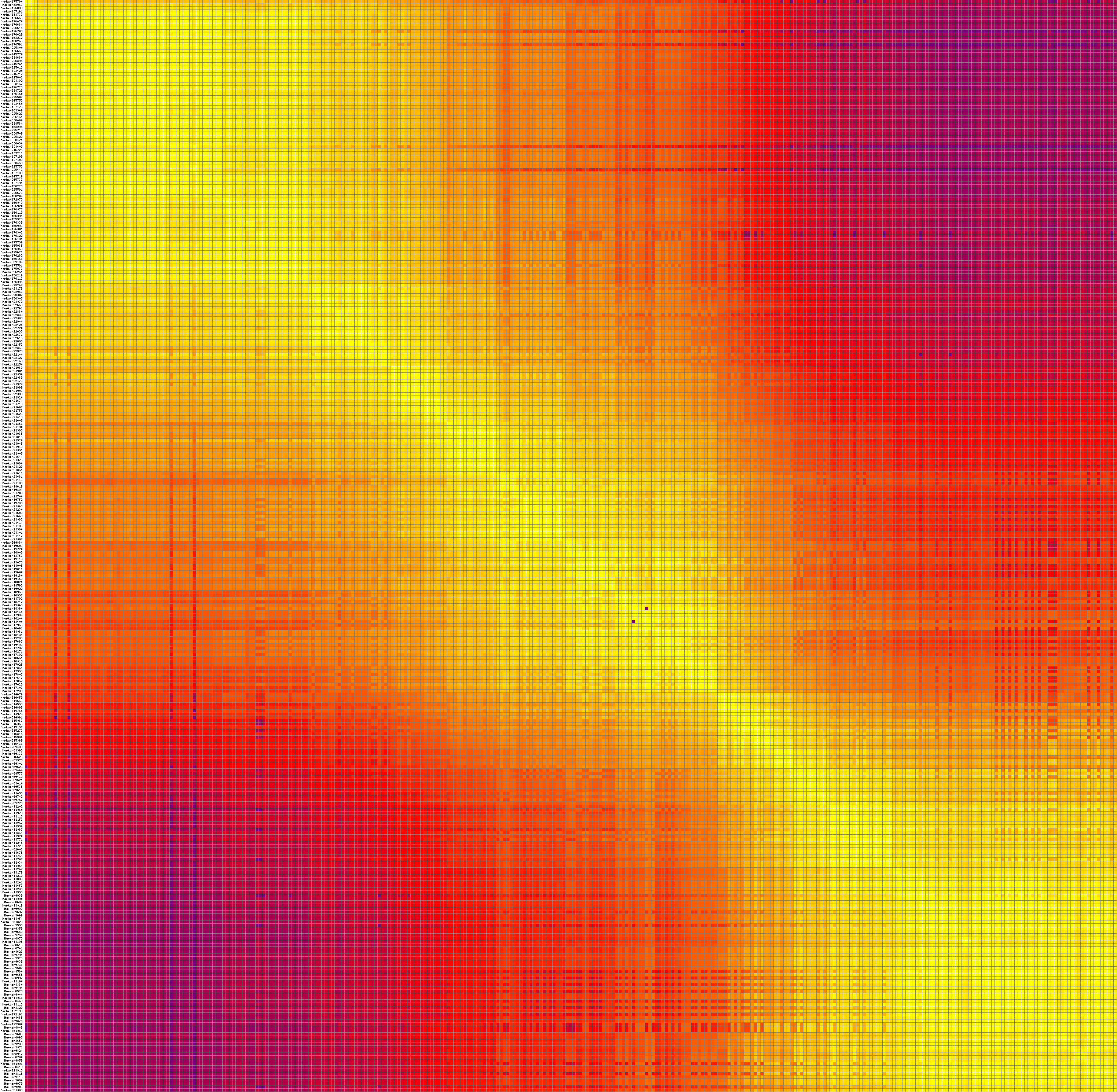

Supplement: Supplementary file 2 [file DataSheet_2.zip › Figure S6/male/LG17.male.heatMap.png]

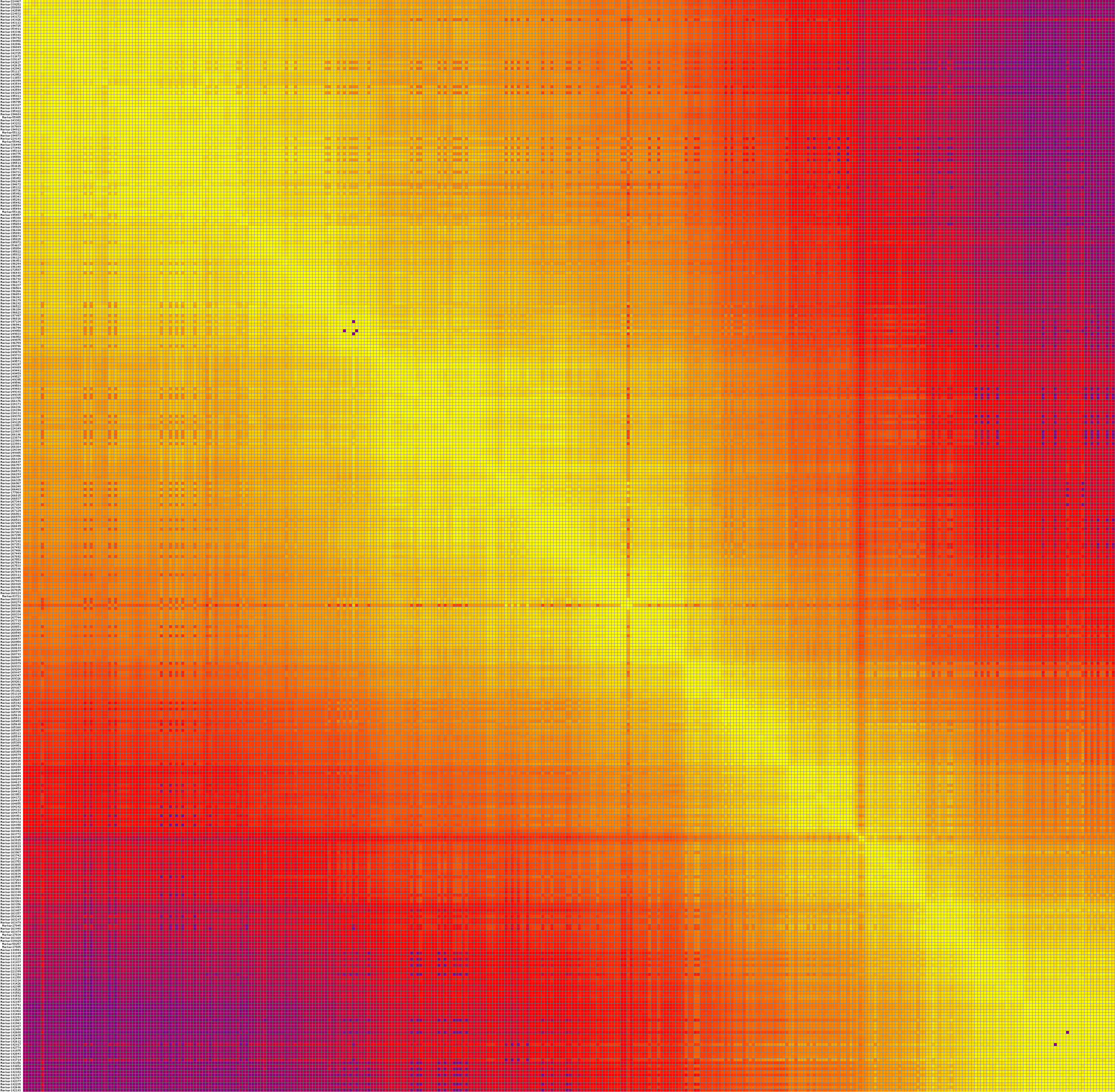

Supplement: Supplementary file 2 [file DataSheet_2.zip › Figure S6/male/LG18.male.heatMap.png]

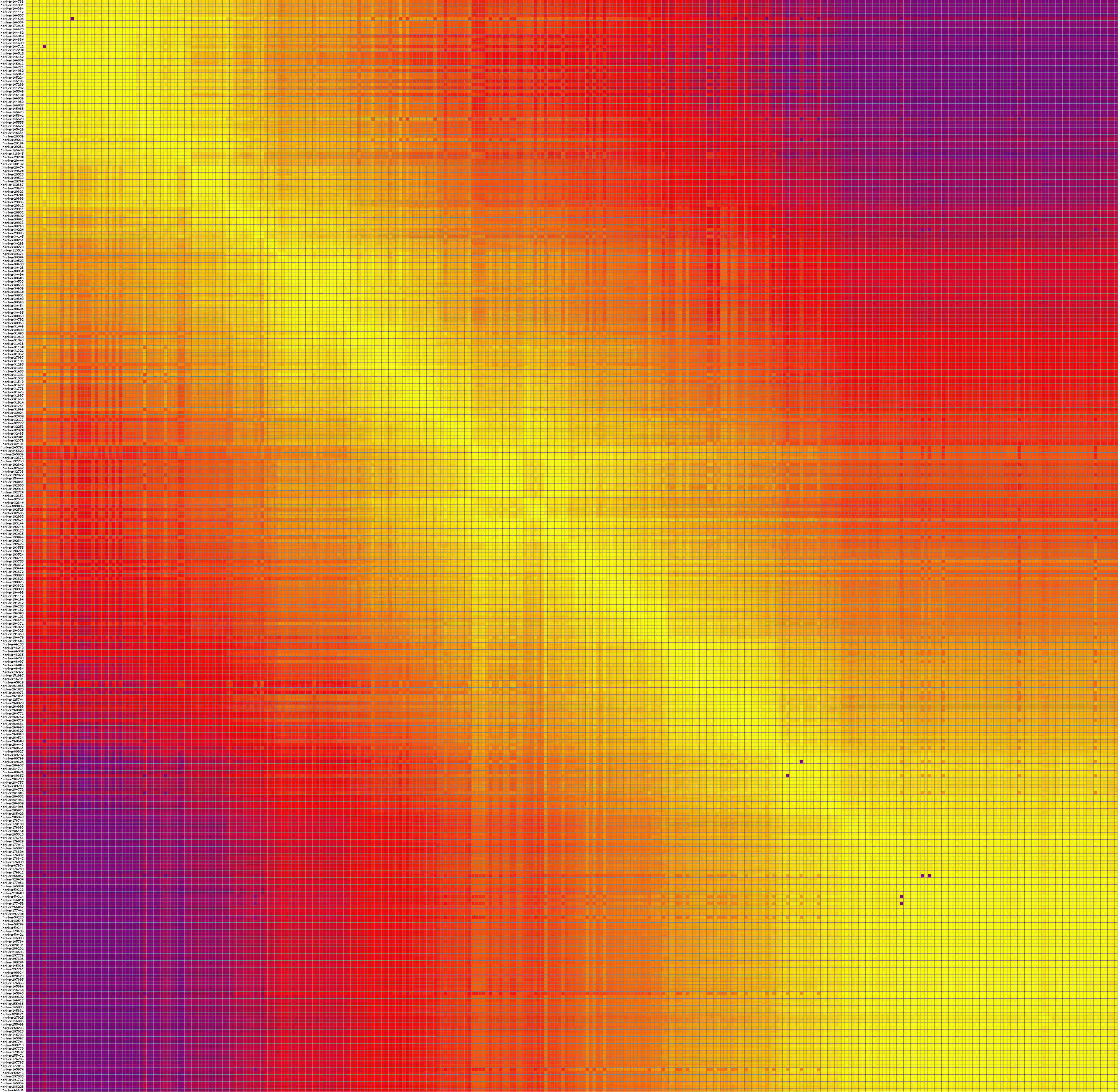

Supplement: Supplementary file 2 [file DataSheet_2.zip › Figure S6/male/LG19.male.heatMap.png]

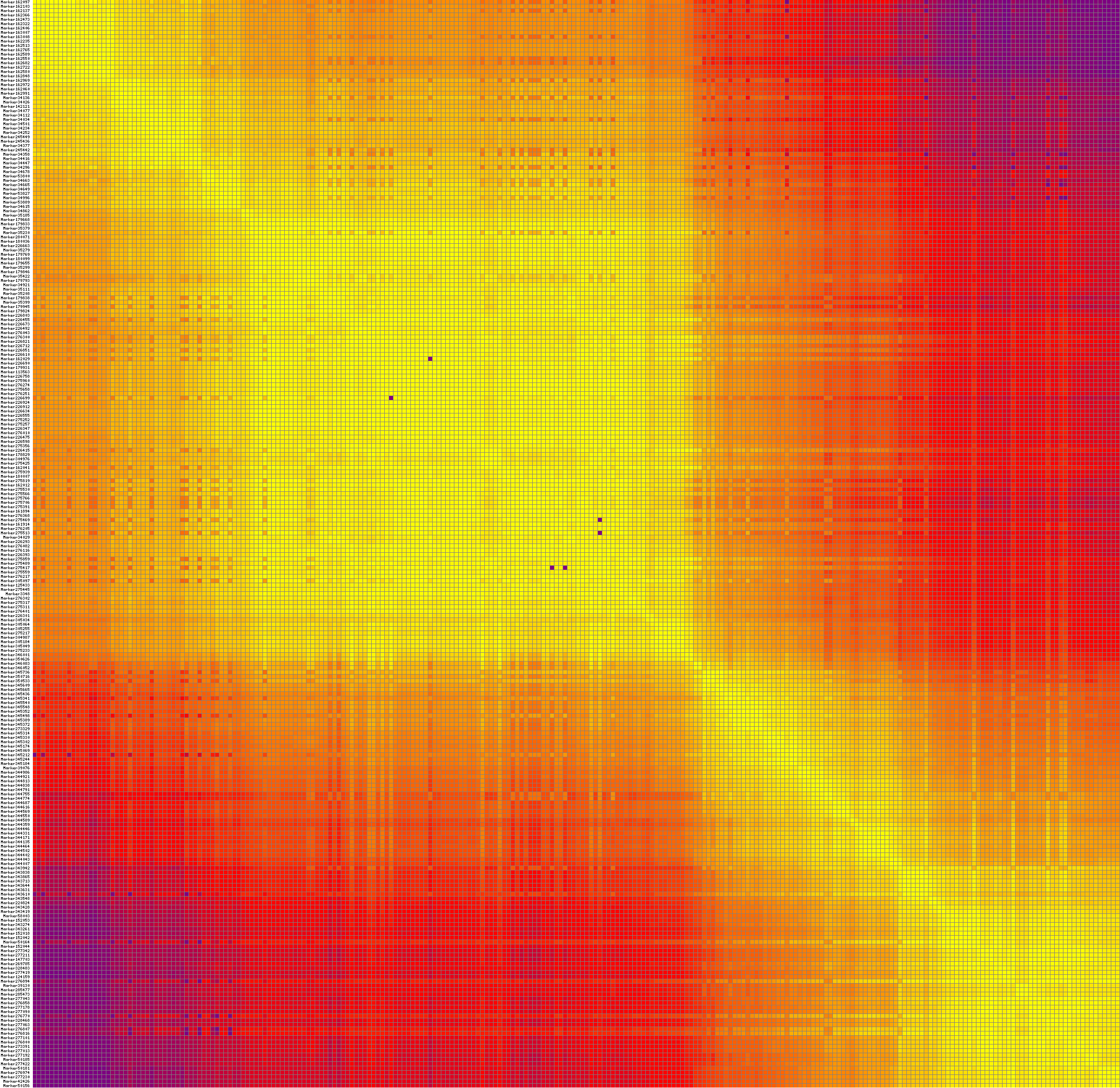

Supplement: Supplementary file 2 [file DataSheet_2.zip › Figure S6/male/LG2.male.heatMap.png]

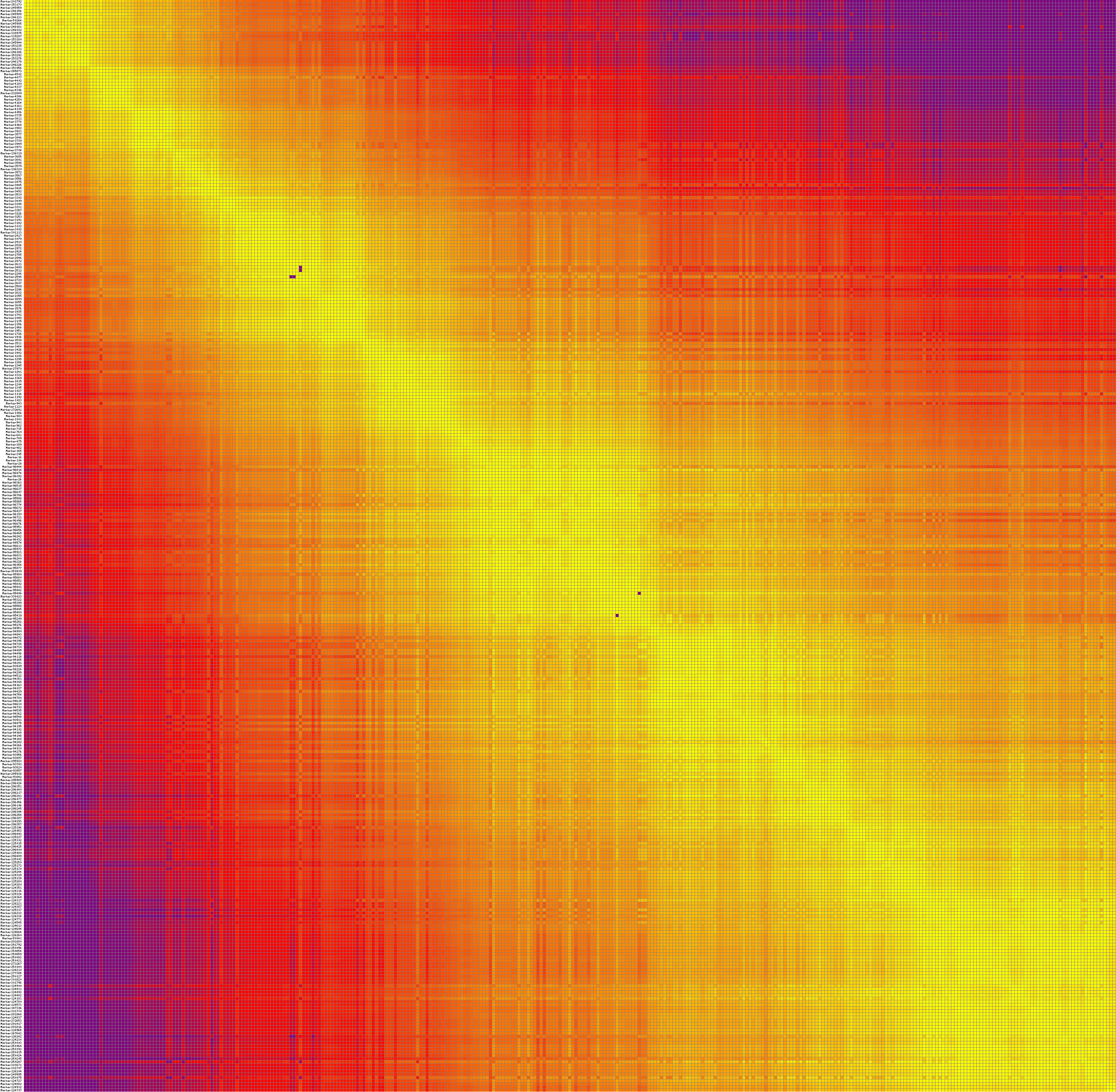

Supplement: Supplementary file 2 [file DataSheet_2.zip › Figure S6/male/LG20.male.heatMap.png]

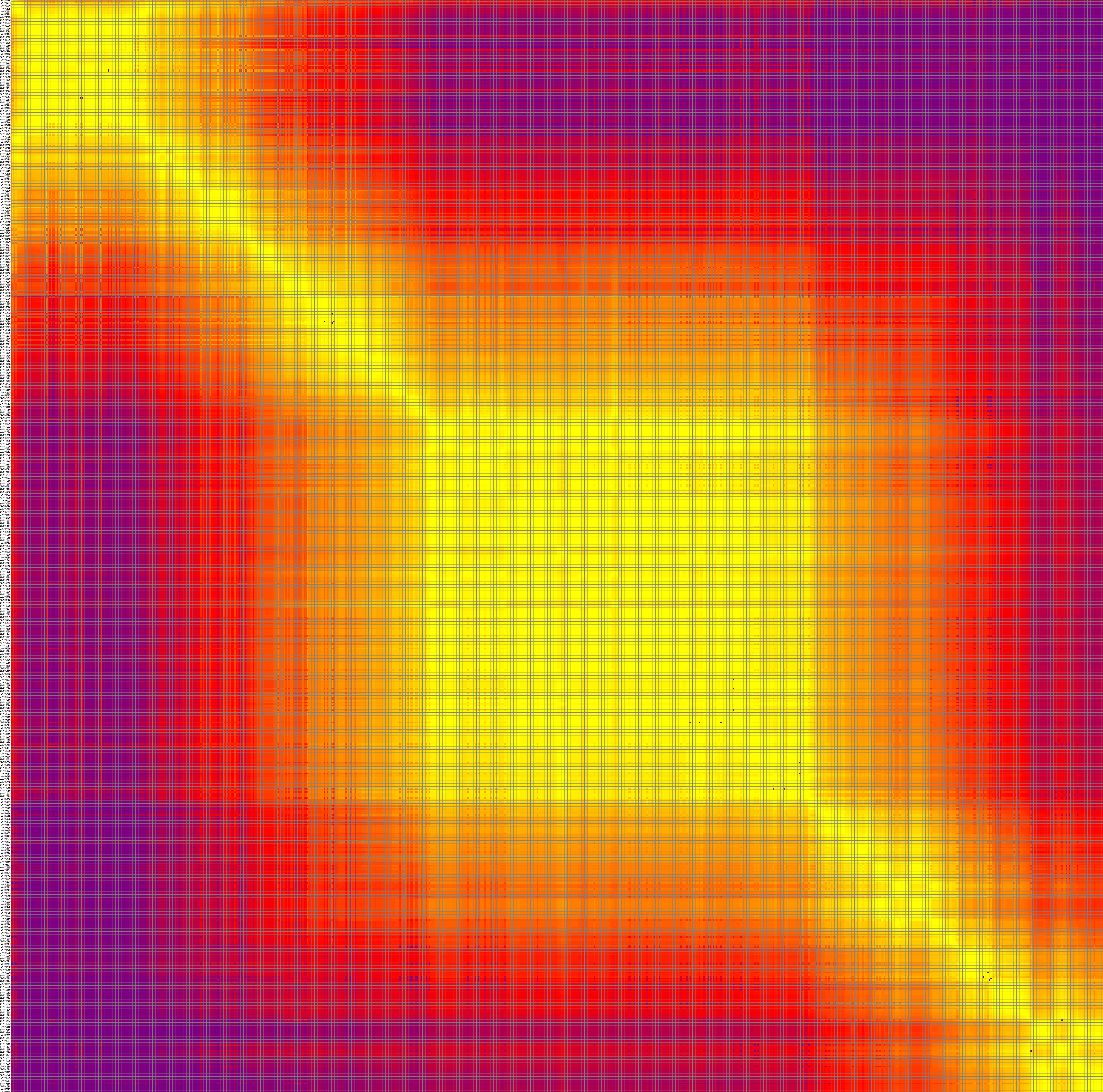

Supplement: Supplementary file 2 [file DataSheet_2.zip › Figure S6/male/LG21.male.heatMap.png]

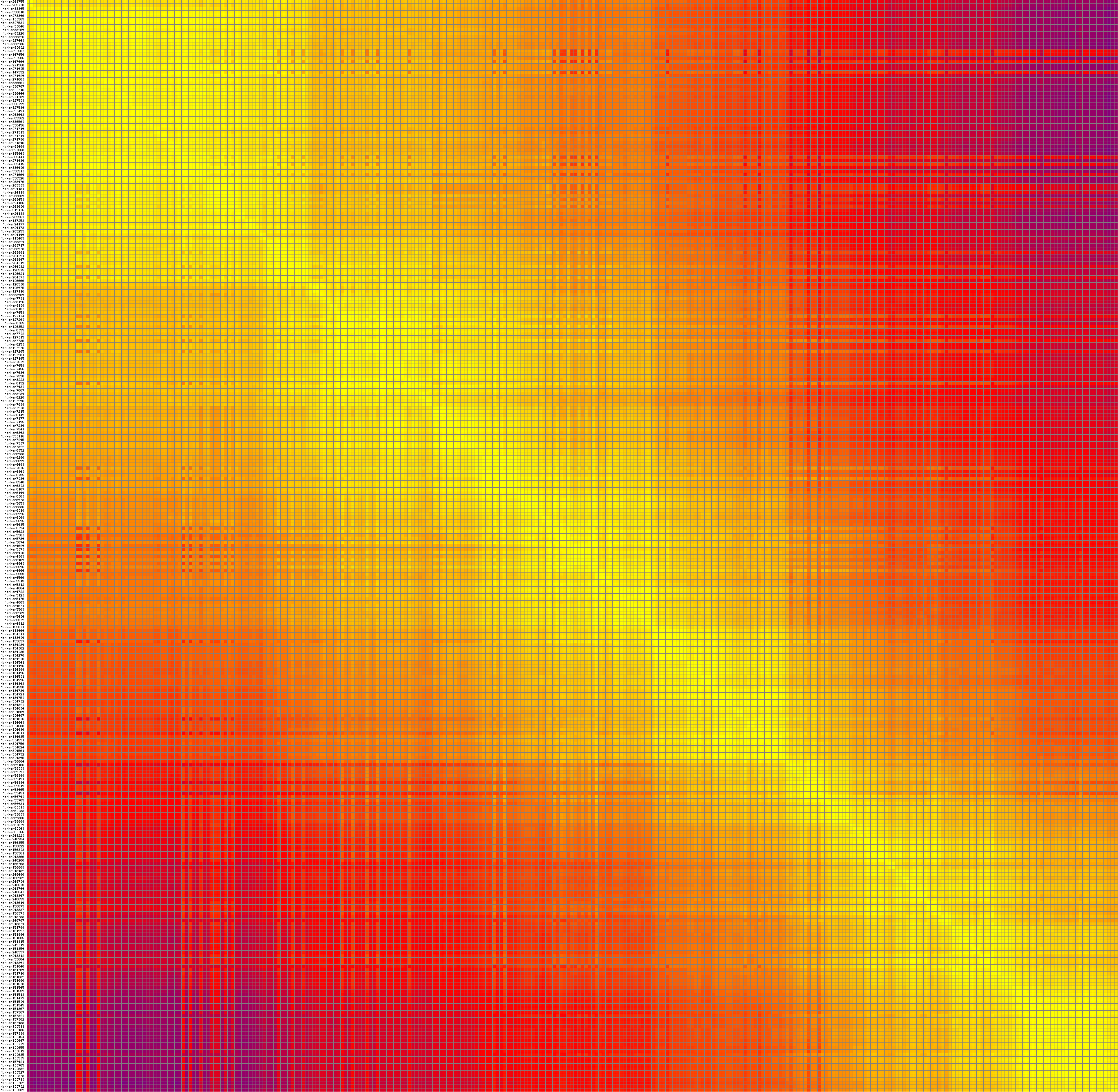

Supplement: Supplementary file 2 [file DataSheet_2.zip › Figure S6/male/LG22.male.heatMap.png]

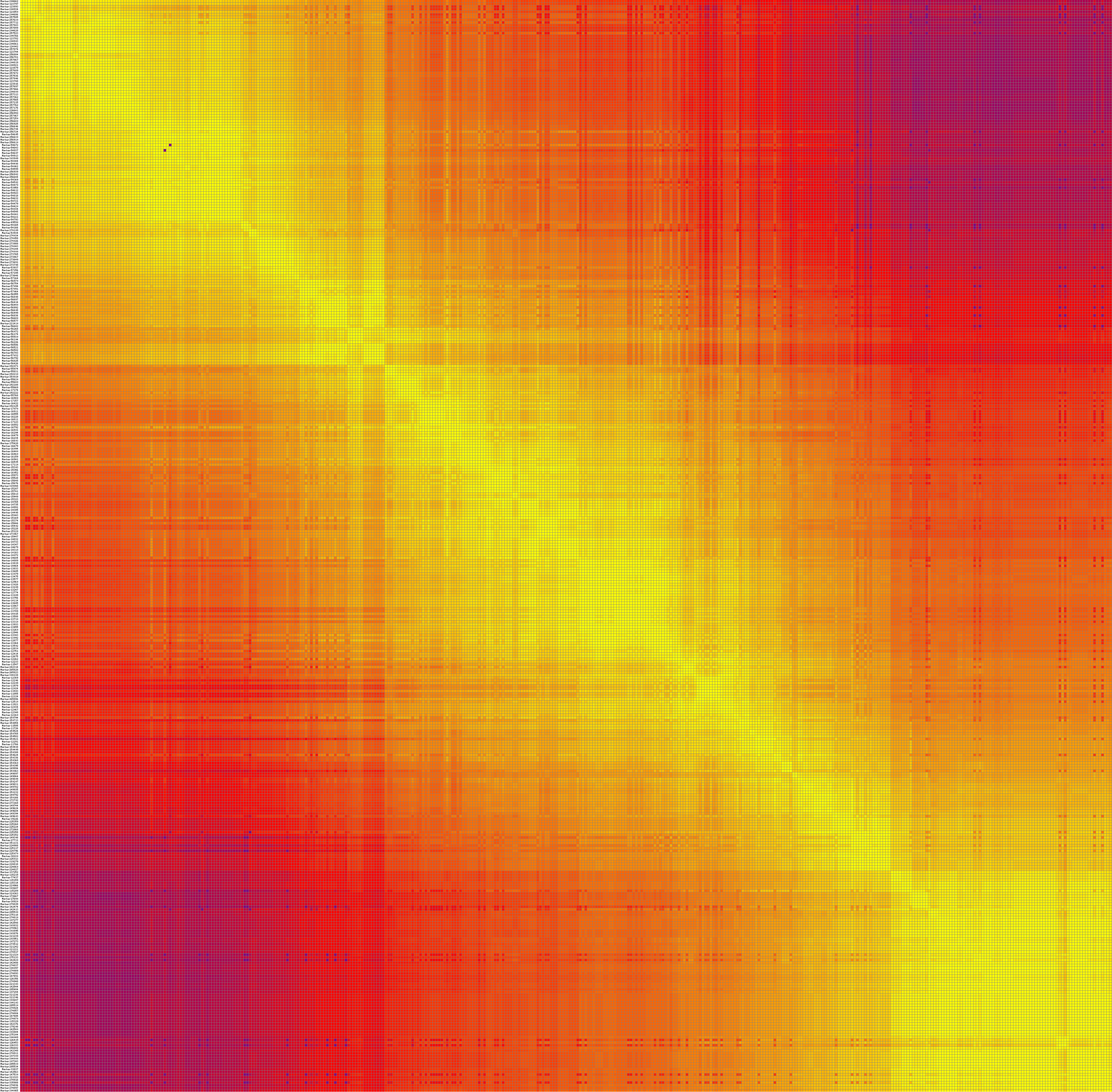

Supplement: Supplementary file 2 [file DataSheet_2.zip › Figure S6/male/LG23.male.heatMap.png]

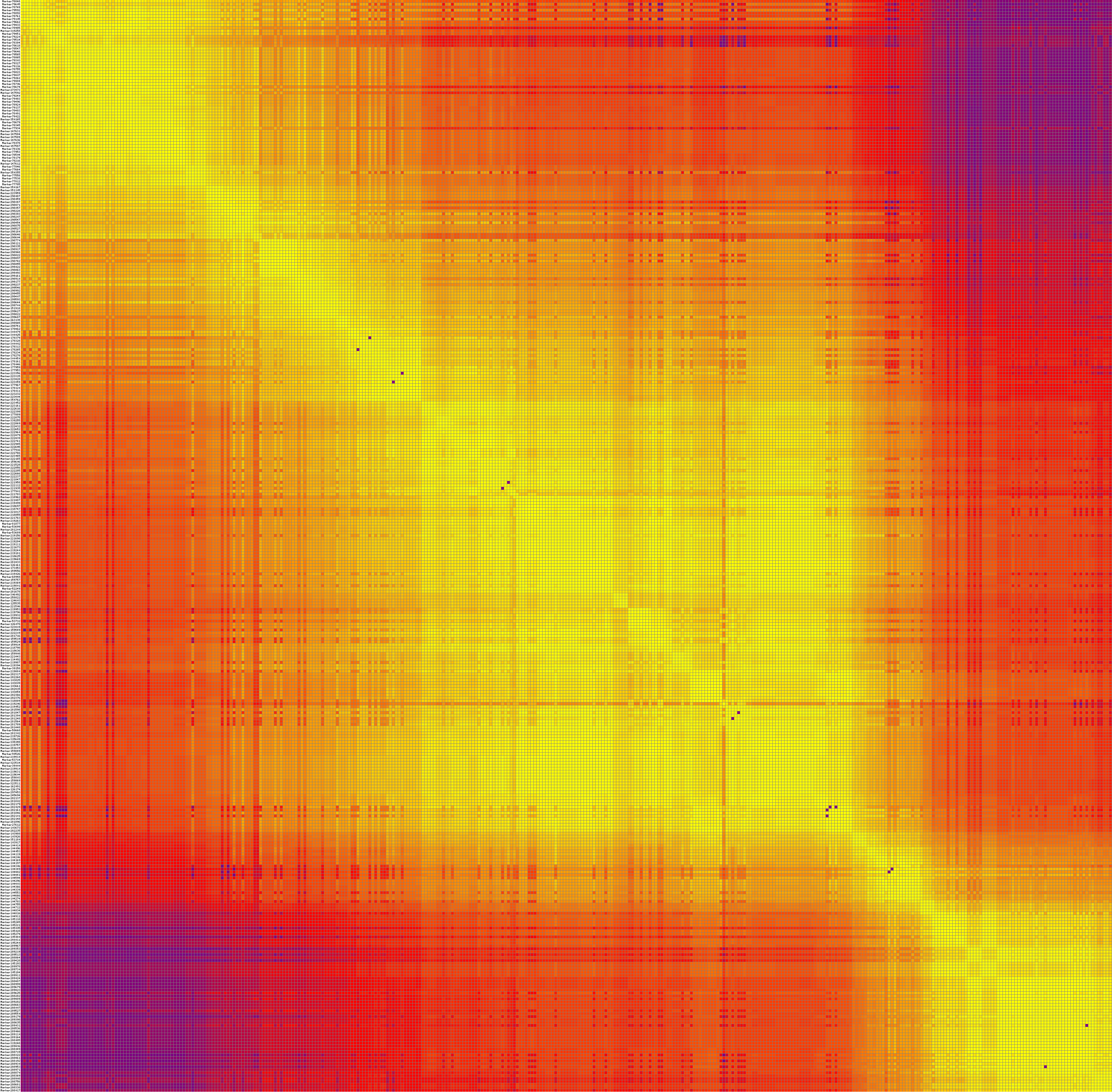

Supplement: Supplementary file 2 [file DataSheet_2.zip › Figure S6/male/LG24.male.heatMap.png]

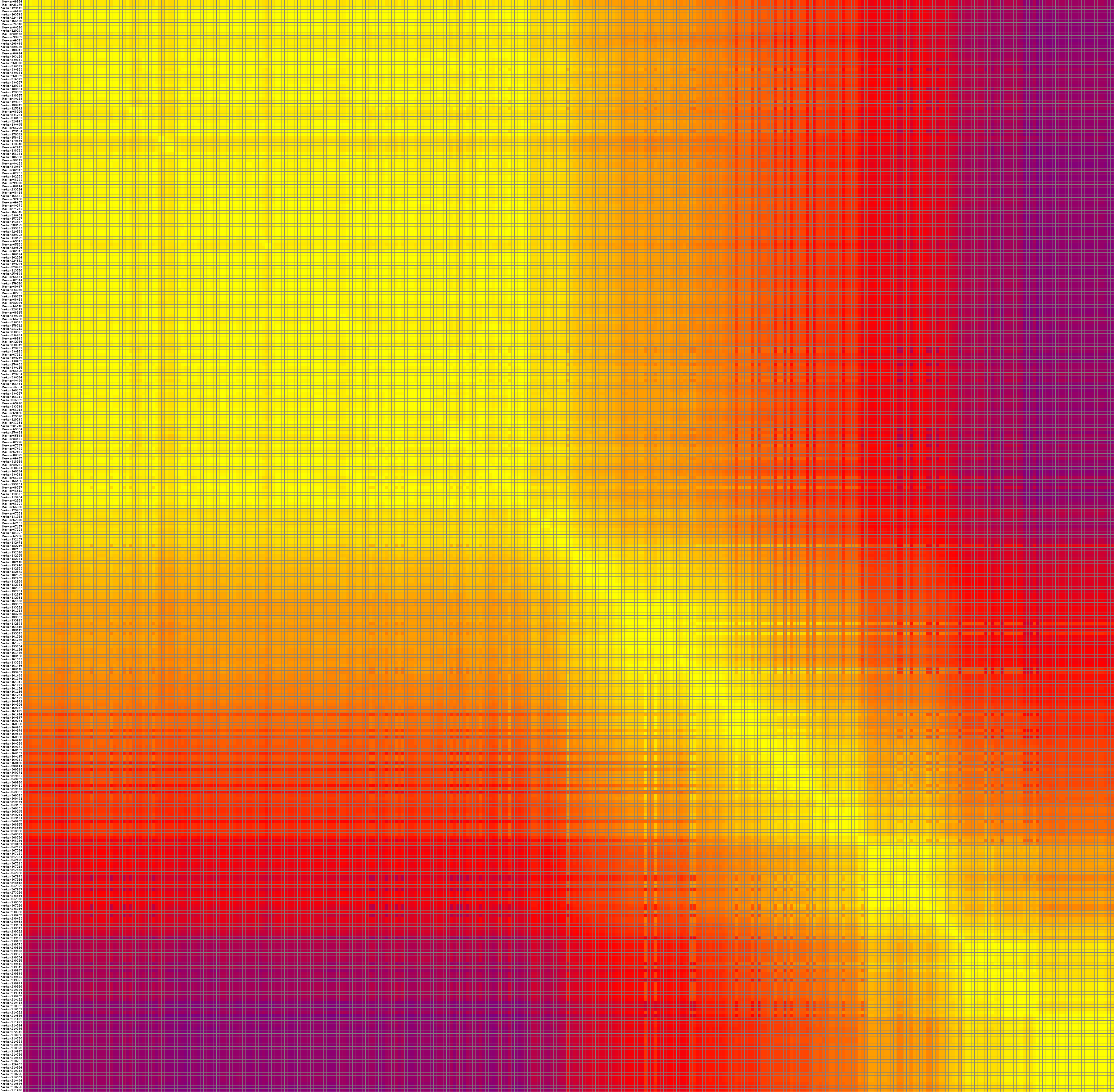

Supplement: Supplementary file 2 [file DataSheet_2.zip › Figure S6/male/LG3.male.heatMap.png]

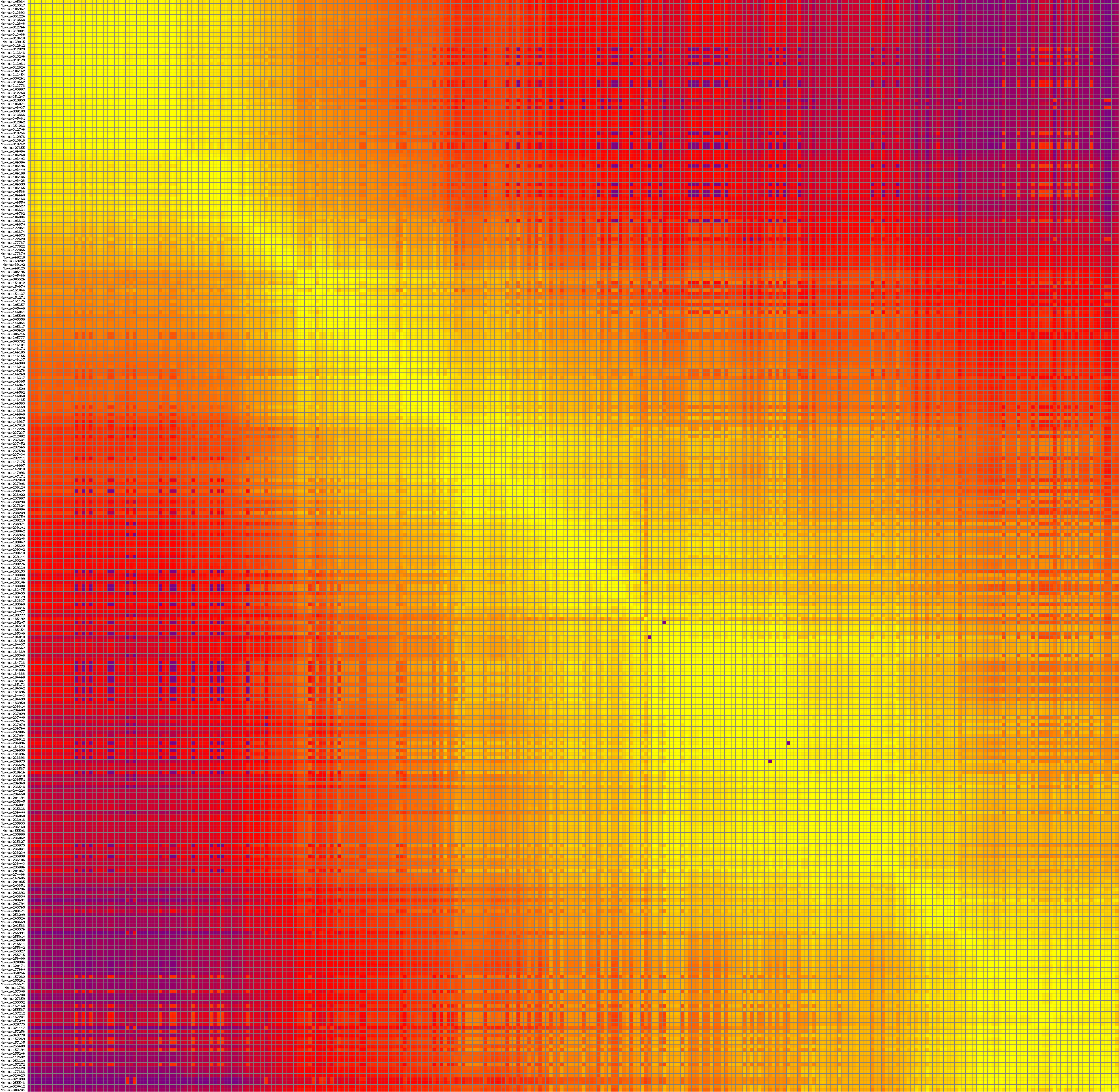

Supplement: Supplementary file 2 [file DataSheet_2.zip › Figure S6/male/LG4.male.heatMap.png]

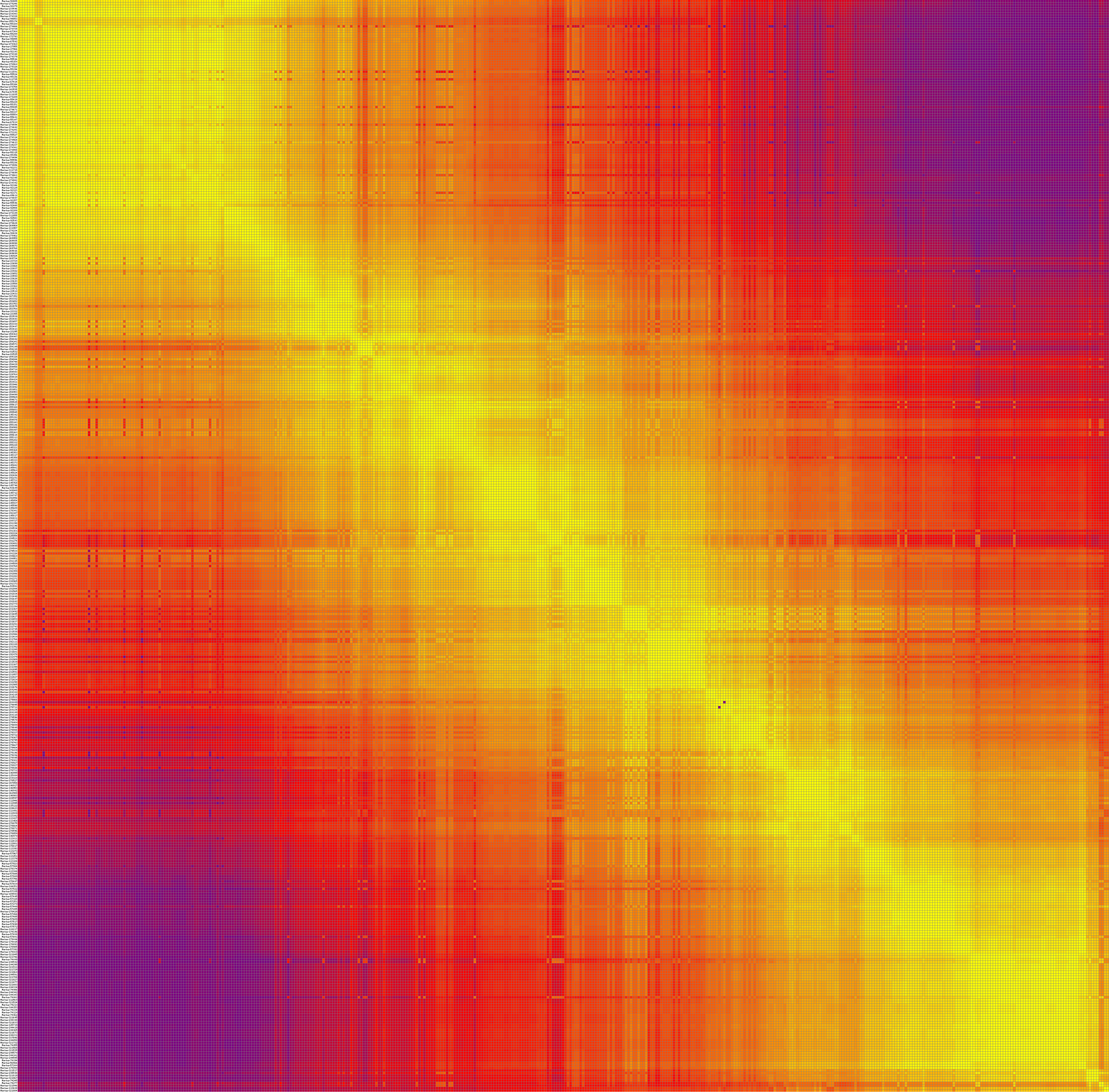

Supplement: Supplementary file 2 [file DataSheet_2.zip › Figure S6/male/LG5.male.heatMap.png]

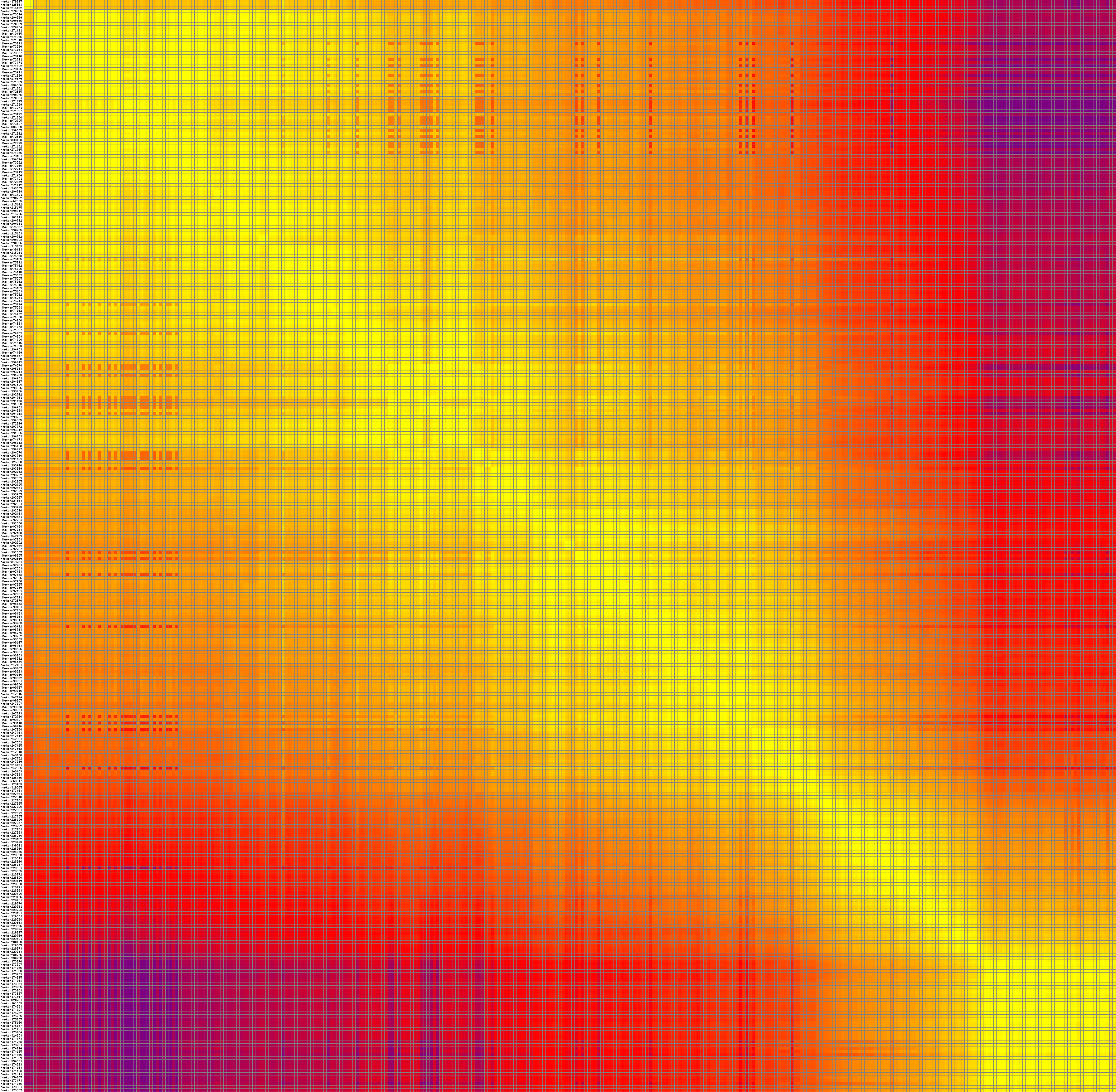

Supplement: Supplementary file 2 [file DataSheet_2.zip › Figure S6/male/LG6.male.heatMap.png]

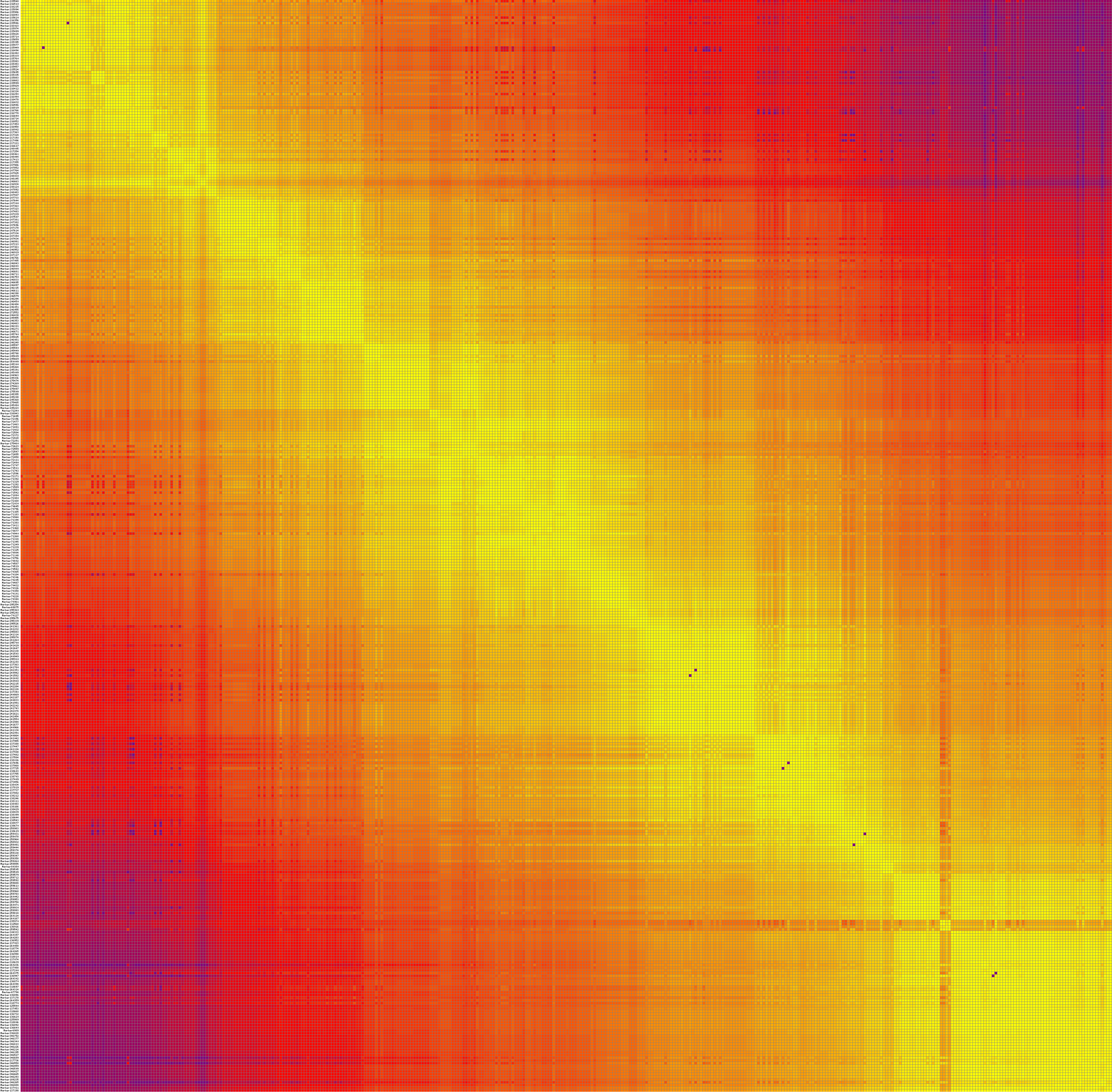

Supplement: Supplementary file 2 [file DataSheet_2.zip › Figure S6/male/LG7.male.heatMap.png]

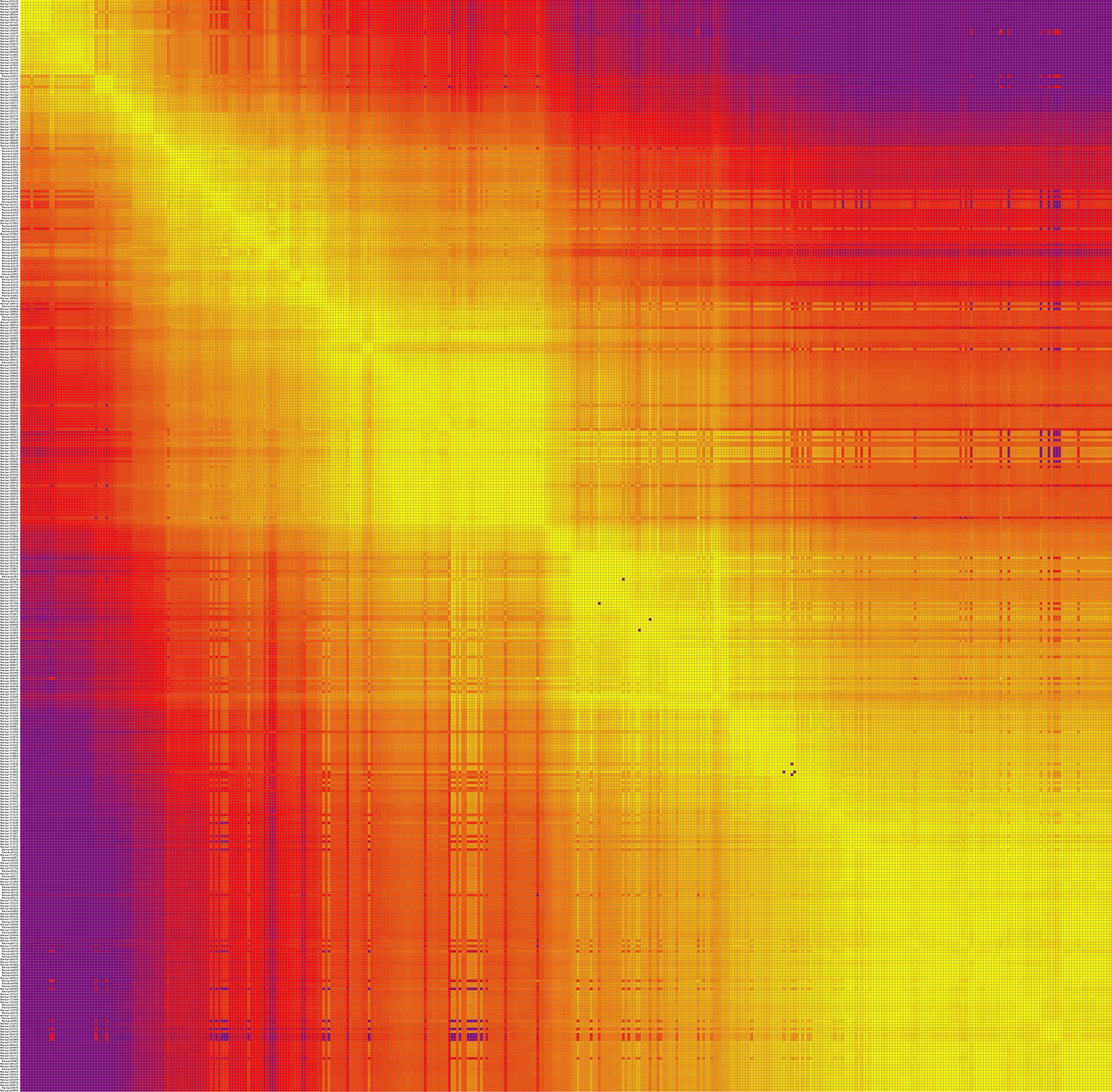

Supplement: Supplementary file 2 [file DataSheet_2.zip › Figure S6/male/LG8.male.heatMap.png]

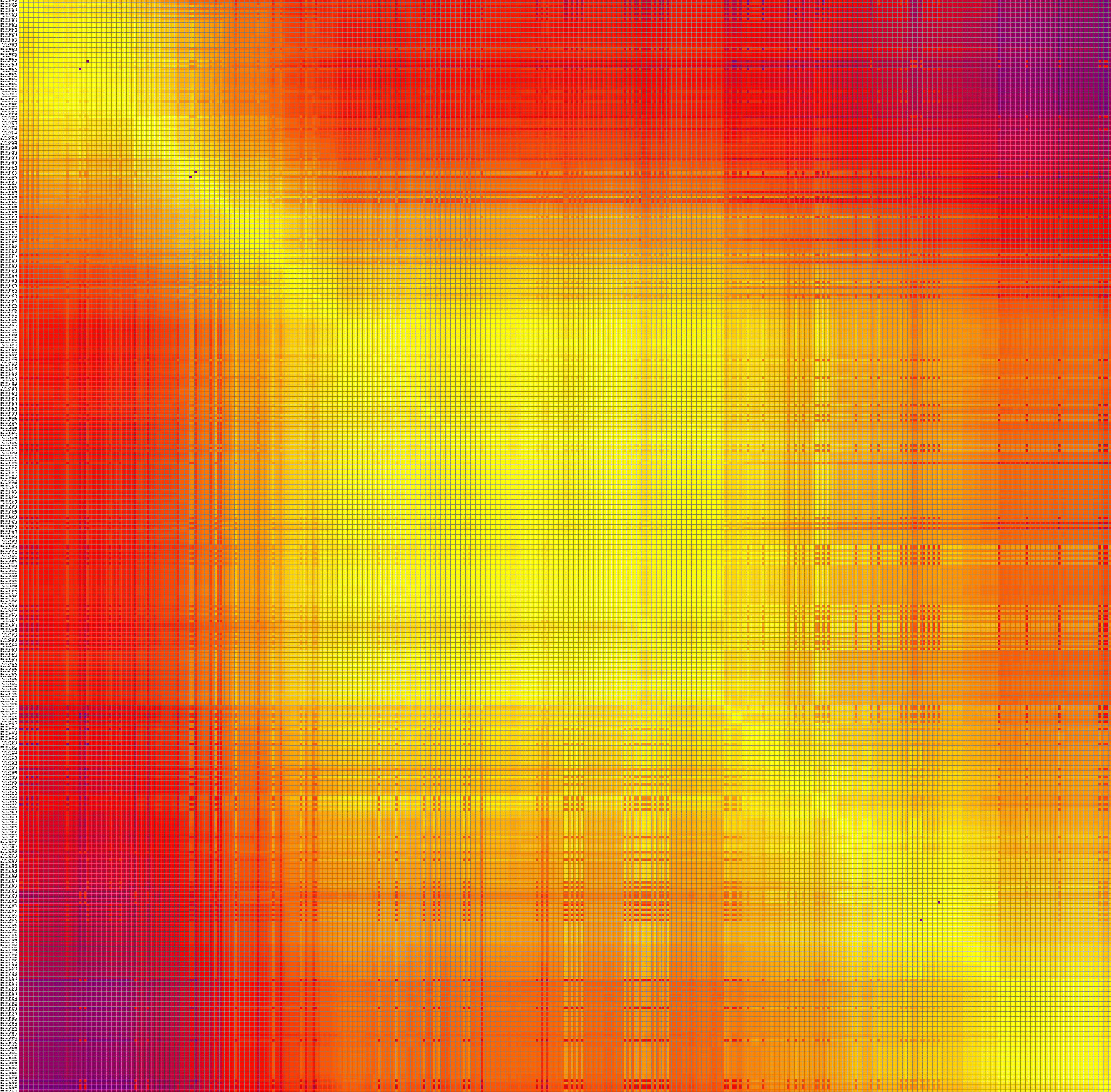

Supplement: Supplementary file 2 [file DataSheet_2.zip › Figure S6/male/LG9.male.heatMap.png]

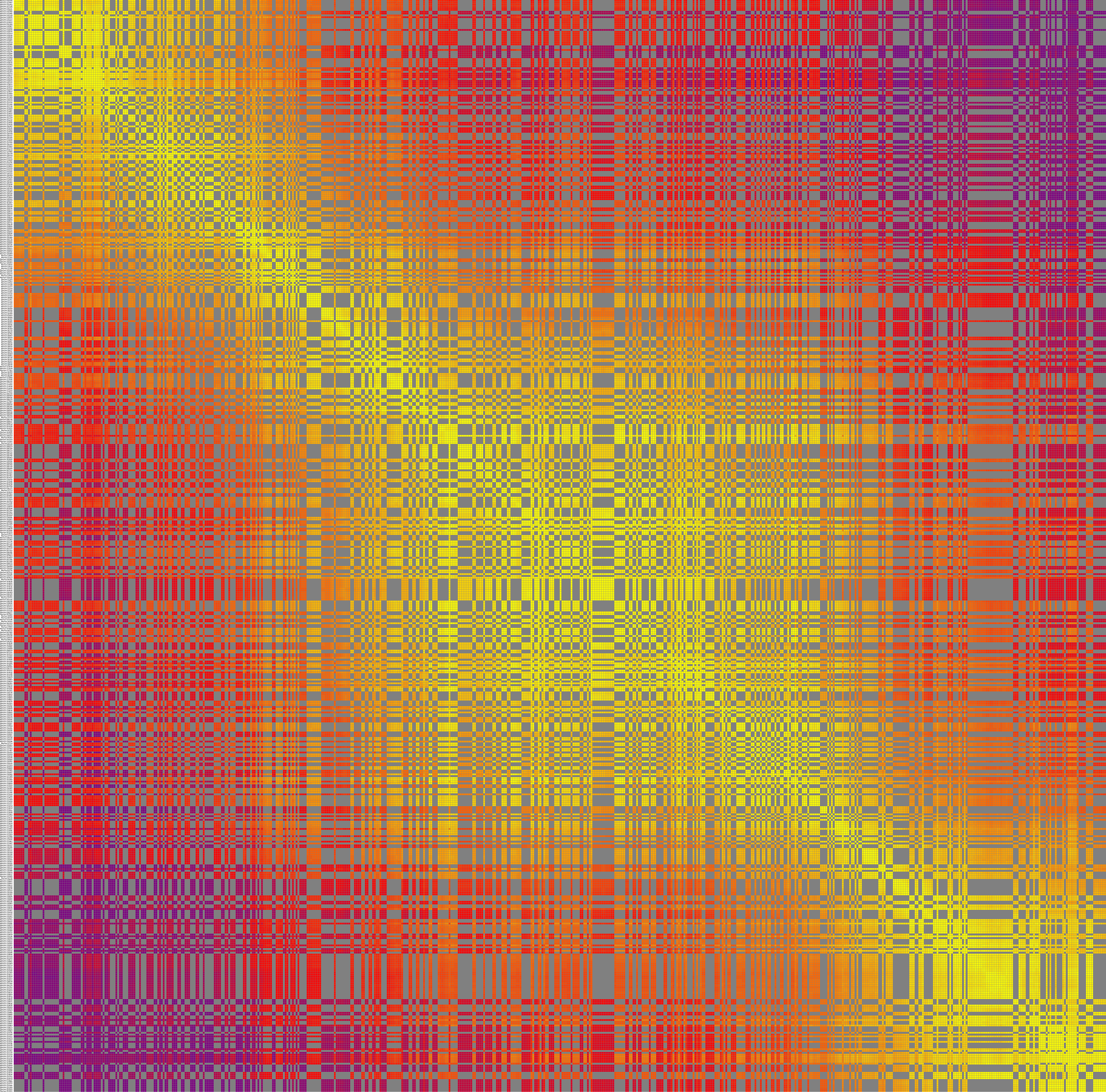

Supplement: Supplementary file 2 [file DataSheet_2.zip › Figure S6/sexAver/LG1.sexAver.heatMap.png]

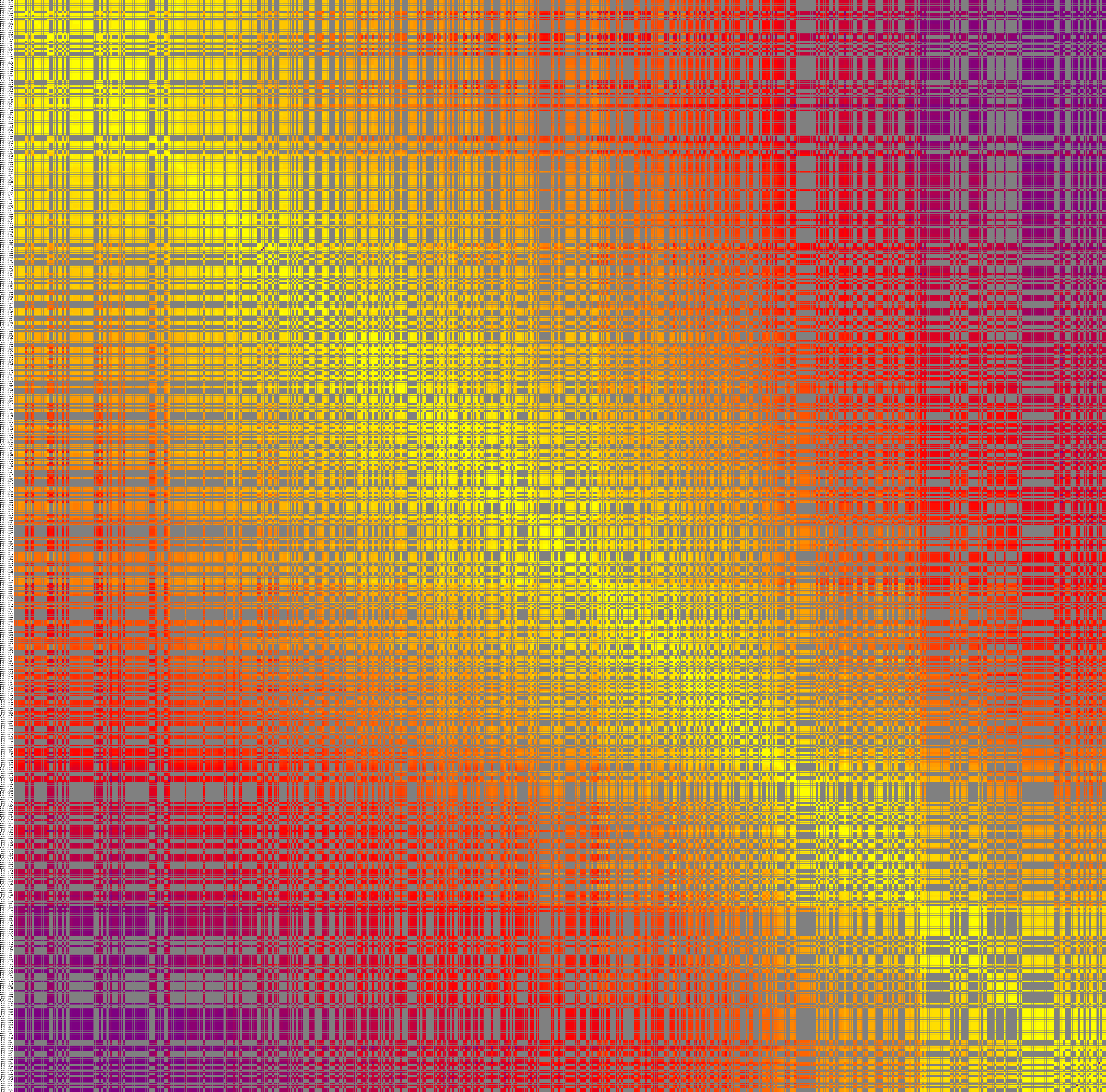

Supplement: Supplementary file 2 [file DataSheet_2.zip › Figure S6/sexAver/LG10.sexAver.heatMap.png]

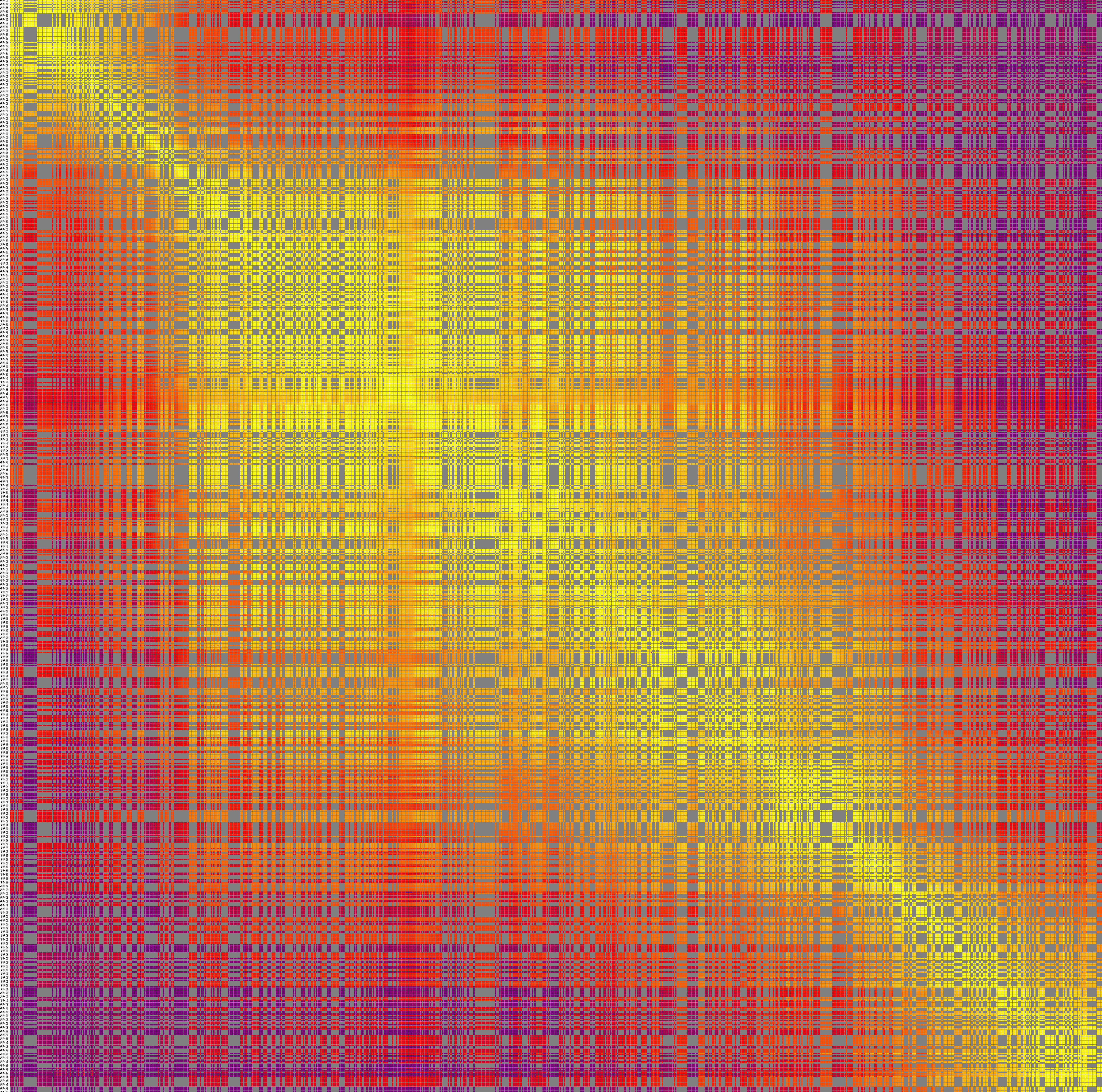

Supplement: Supplementary file 2 [file DataSheet_2.zip › Figure S6/sexAver/LG11.sexAver.heatMap.png]

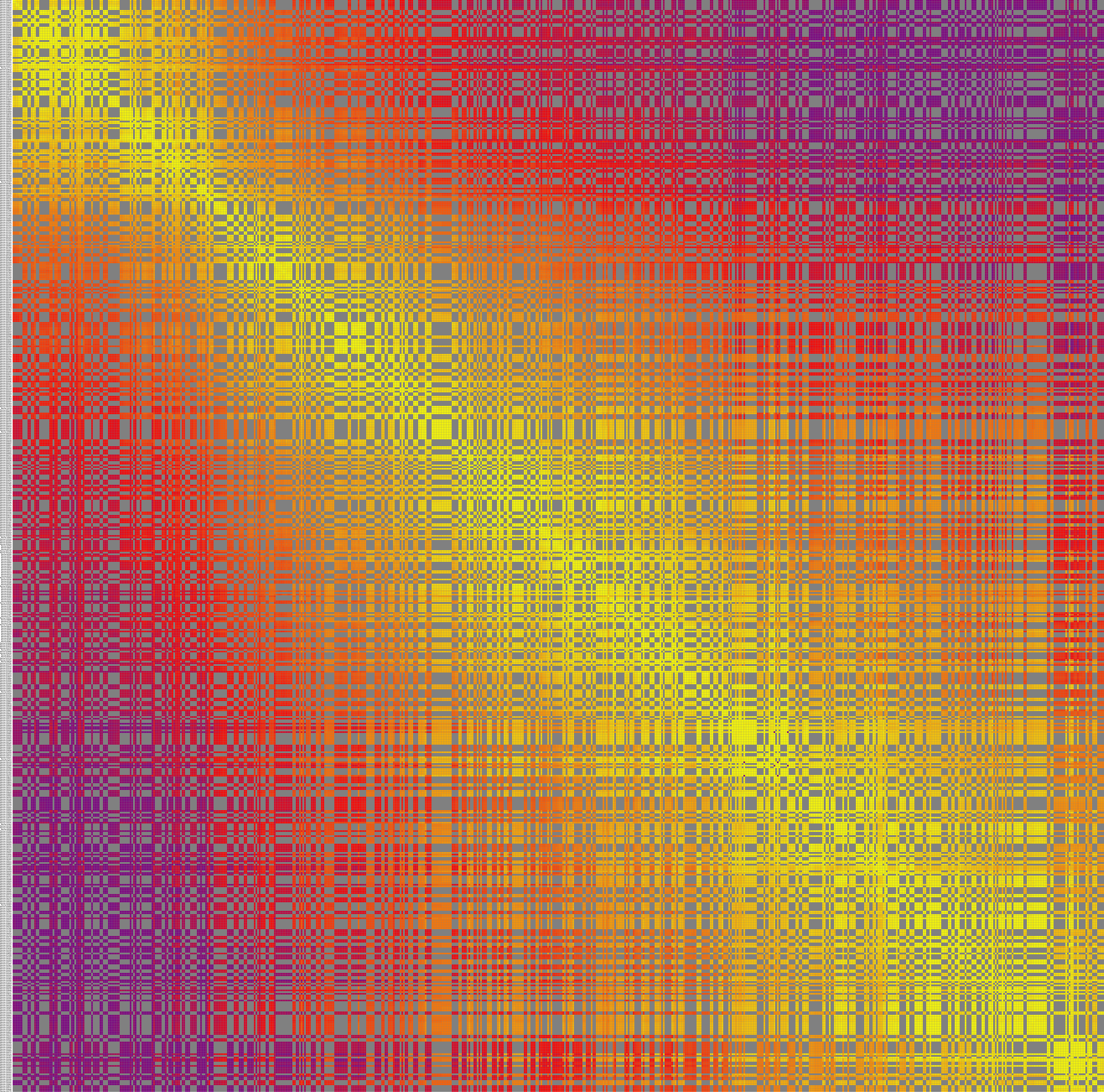

Supplement: Supplementary file 2 [file DataSheet_2.zip › Figure S6/sexAver/LG12.sexAver.heatMap.png]

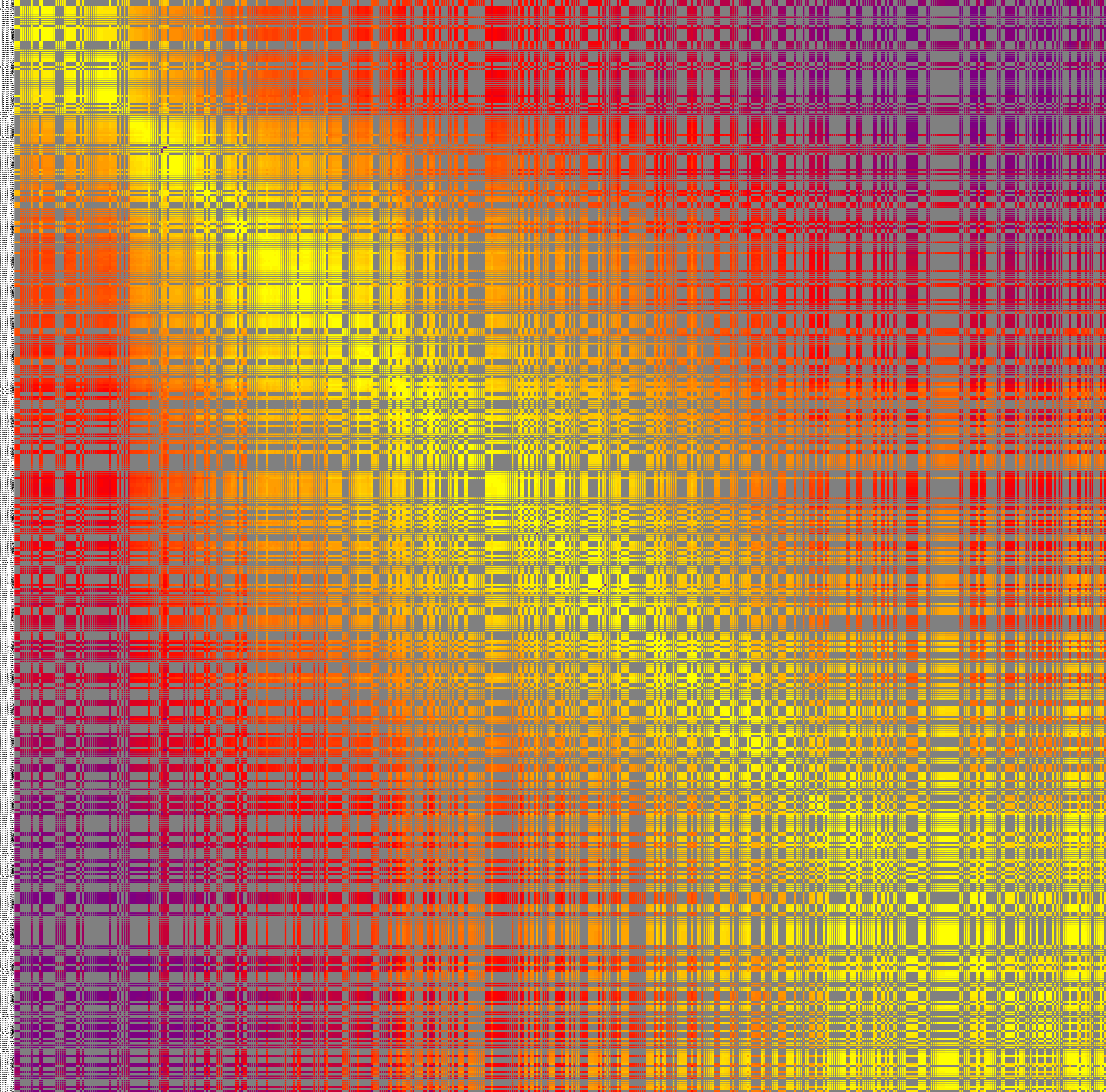

Supplement: Supplementary file 2 [file DataSheet_2.zip › Figure S6/sexAver/LG13.sexAver.heatMap.png]

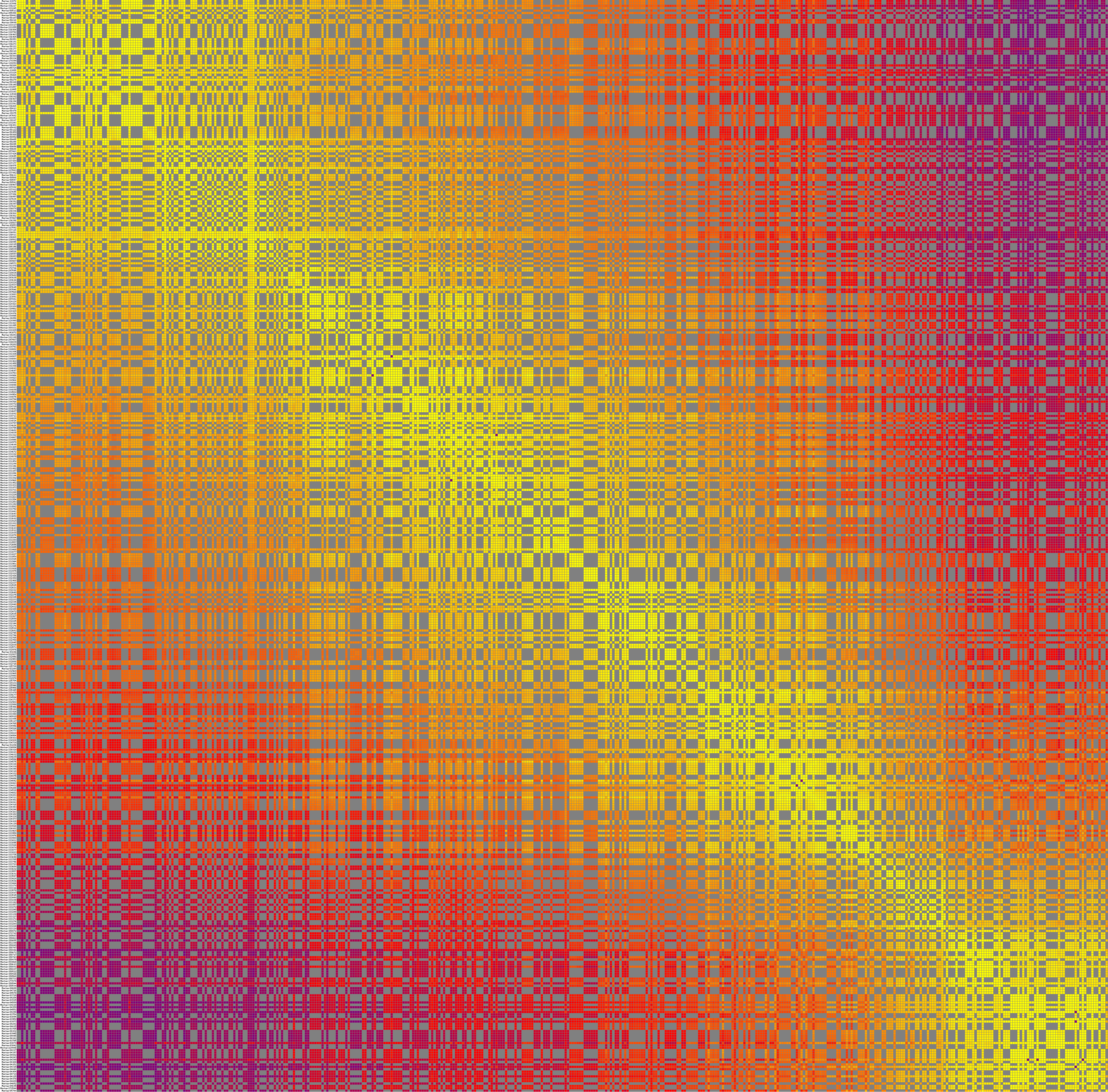

Supplement: Supplementary file 2 [file DataSheet_2.zip › Figure S6/sexAver/LG14.sexAver.heatMap.png]

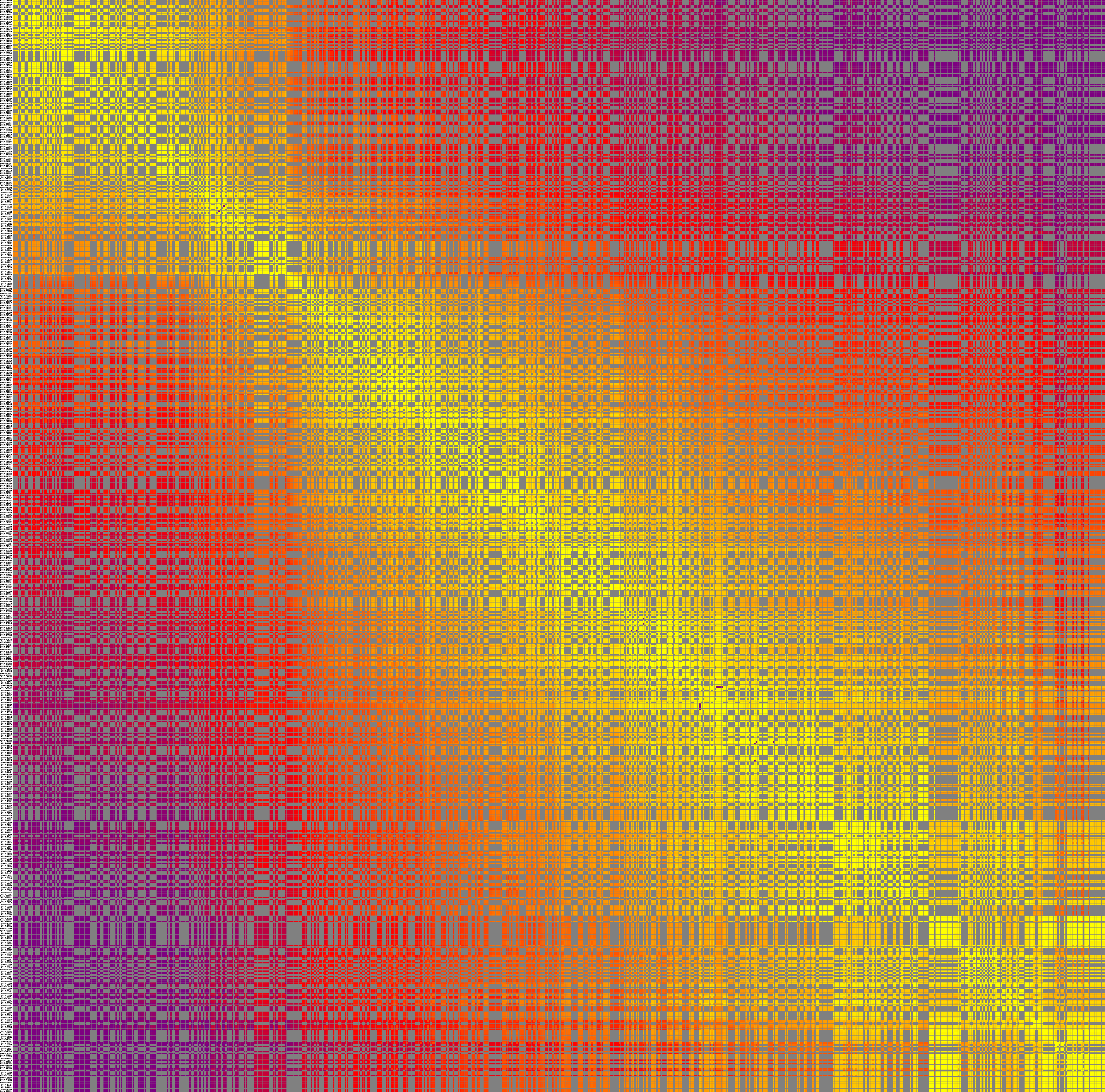

Supplement: Supplementary file 2 [file DataSheet_2.zip › Figure S6/sexAver/LG16.sexAver.heatMap.png]

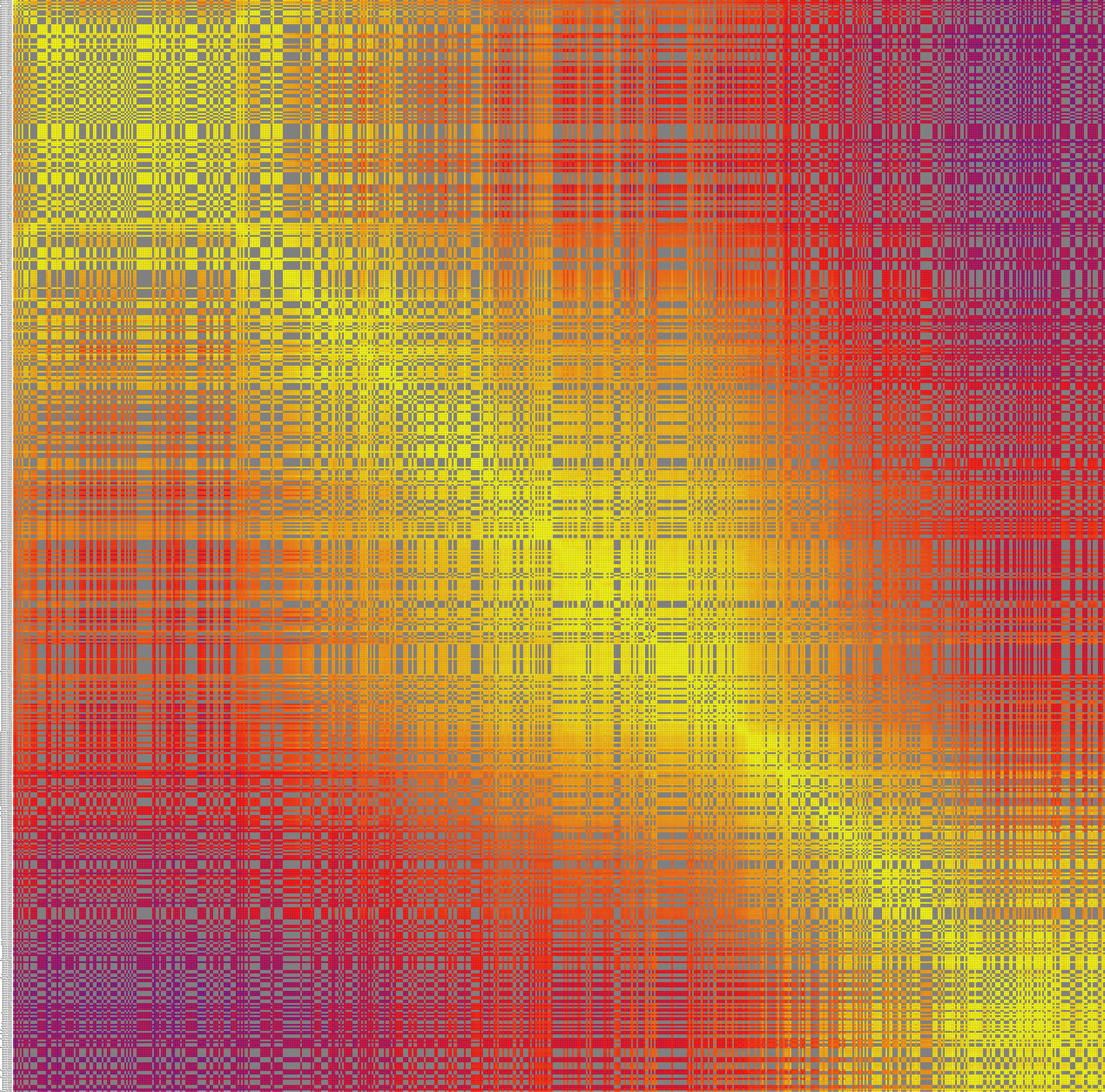

Supplement: Supplementary file 2 [file DataSheet_2.zip › Figure S6/sexAver/LG17.sexAver.heatMap.png]

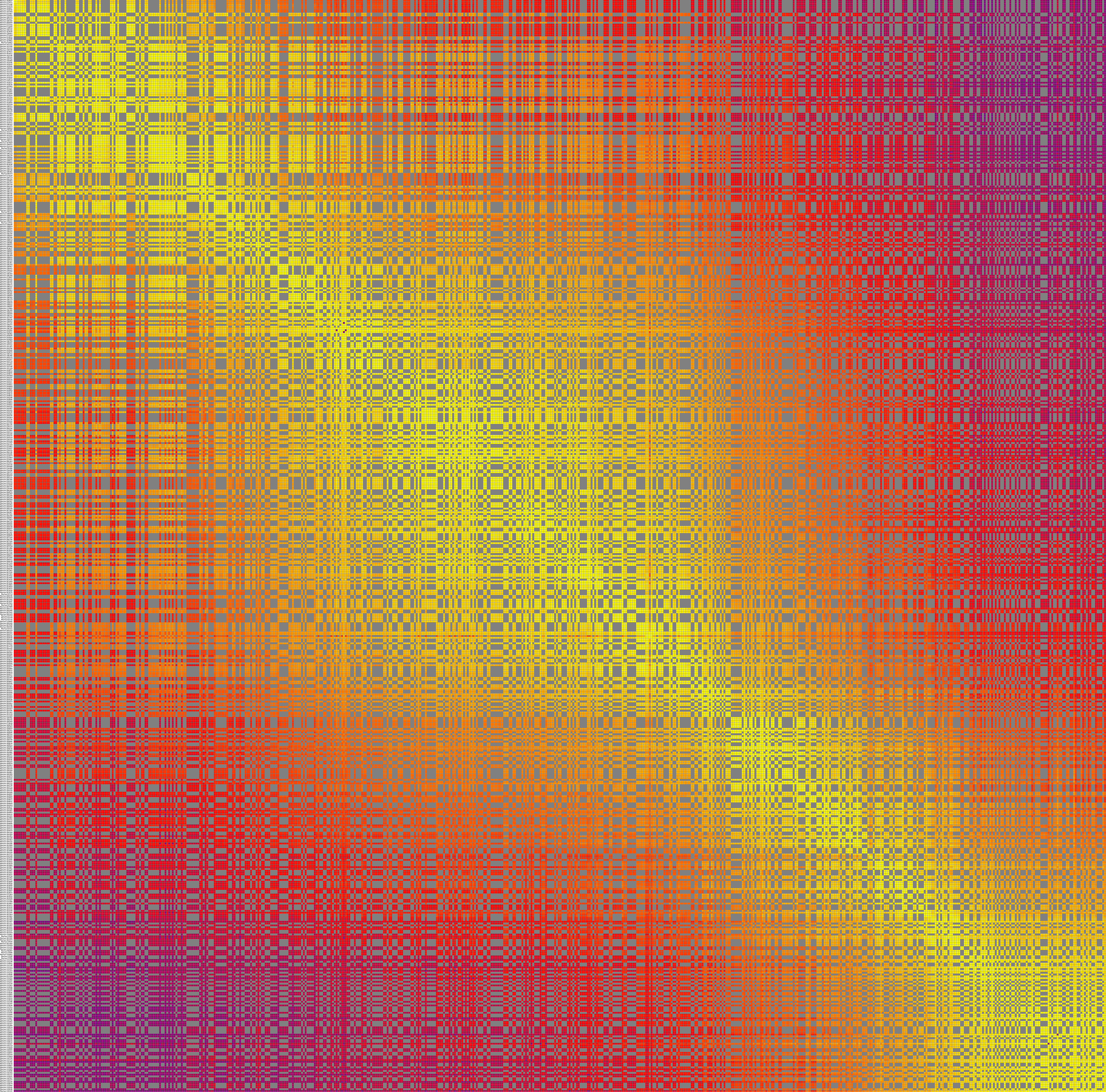

Supplement: Supplementary file 2 [file DataSheet_2.zip › Figure S6/sexAver/LG18.sexAver.heatMap.png]

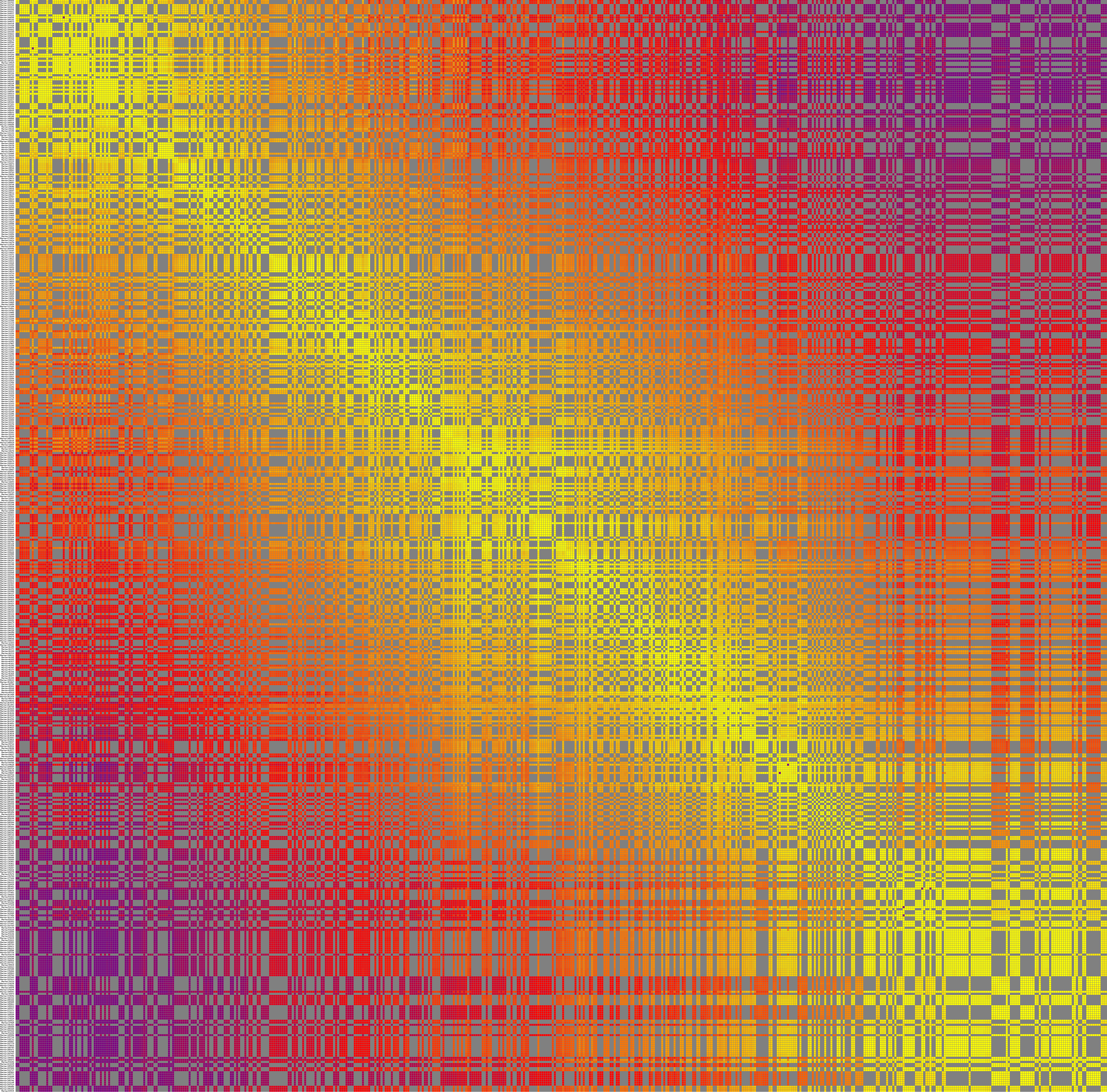

Supplement: Supplementary file 2 [file DataSheet_2.zip › Figure S6/sexAver/LG19.sexAver.heatMap.png]

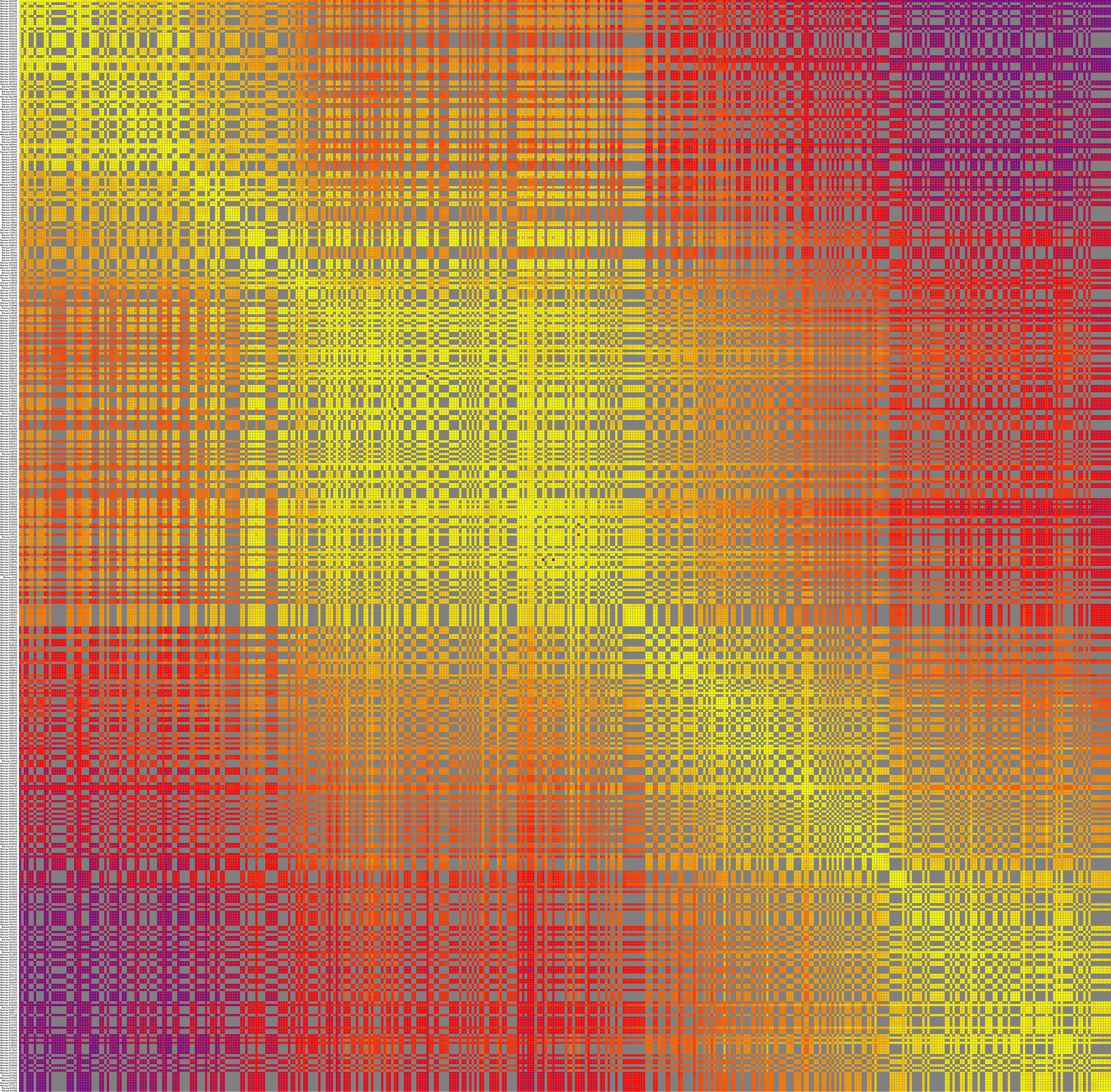

Supplement: Supplementary file 2 [file DataSheet_2.zip › Figure S6/sexAver/LG2.sexAver.heatMap.png]

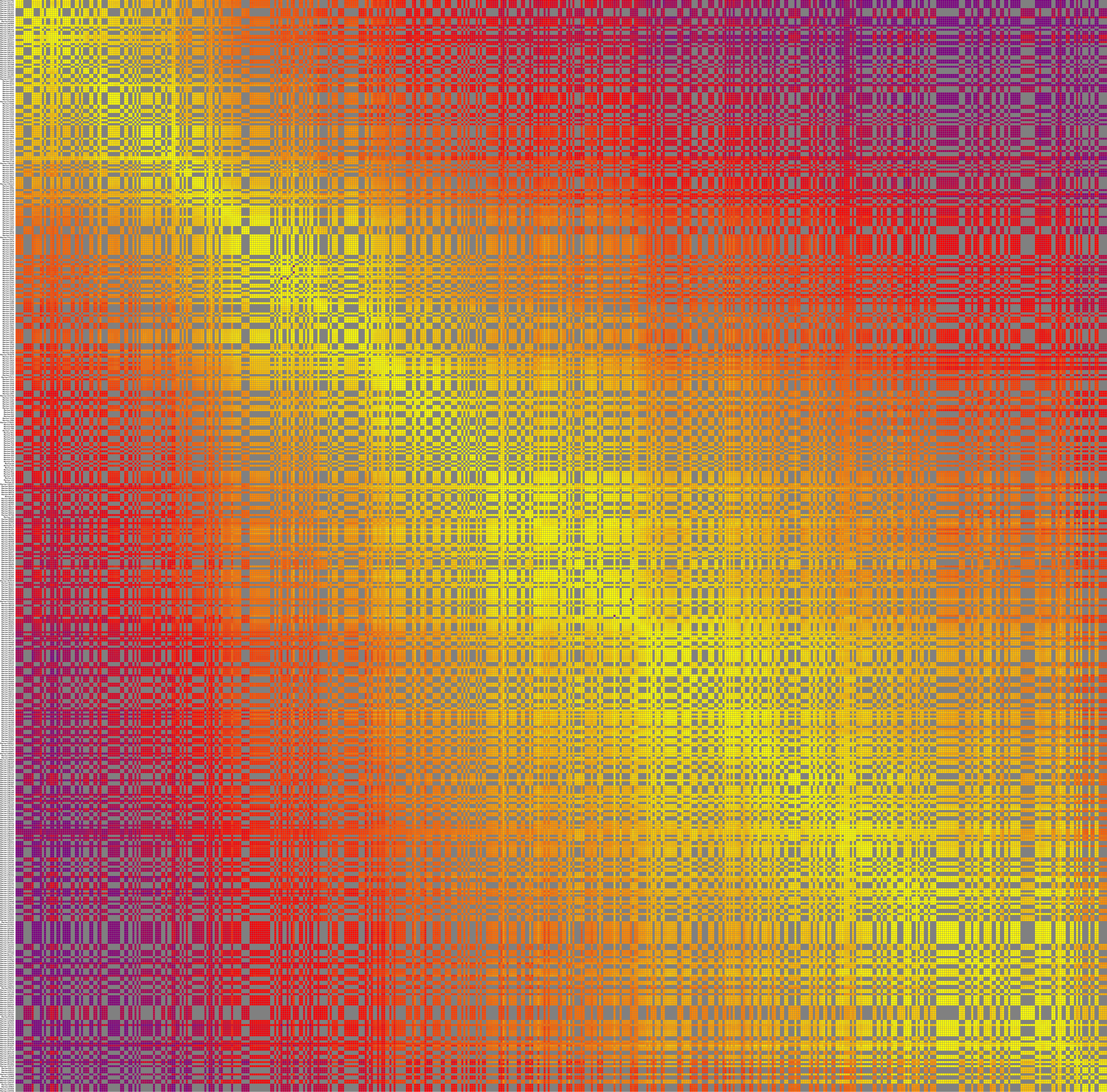

Supplement: Supplementary file 2 [file DataSheet_2.zip › Figure S6/sexAver/LG20.sexAver.heatMap.png]

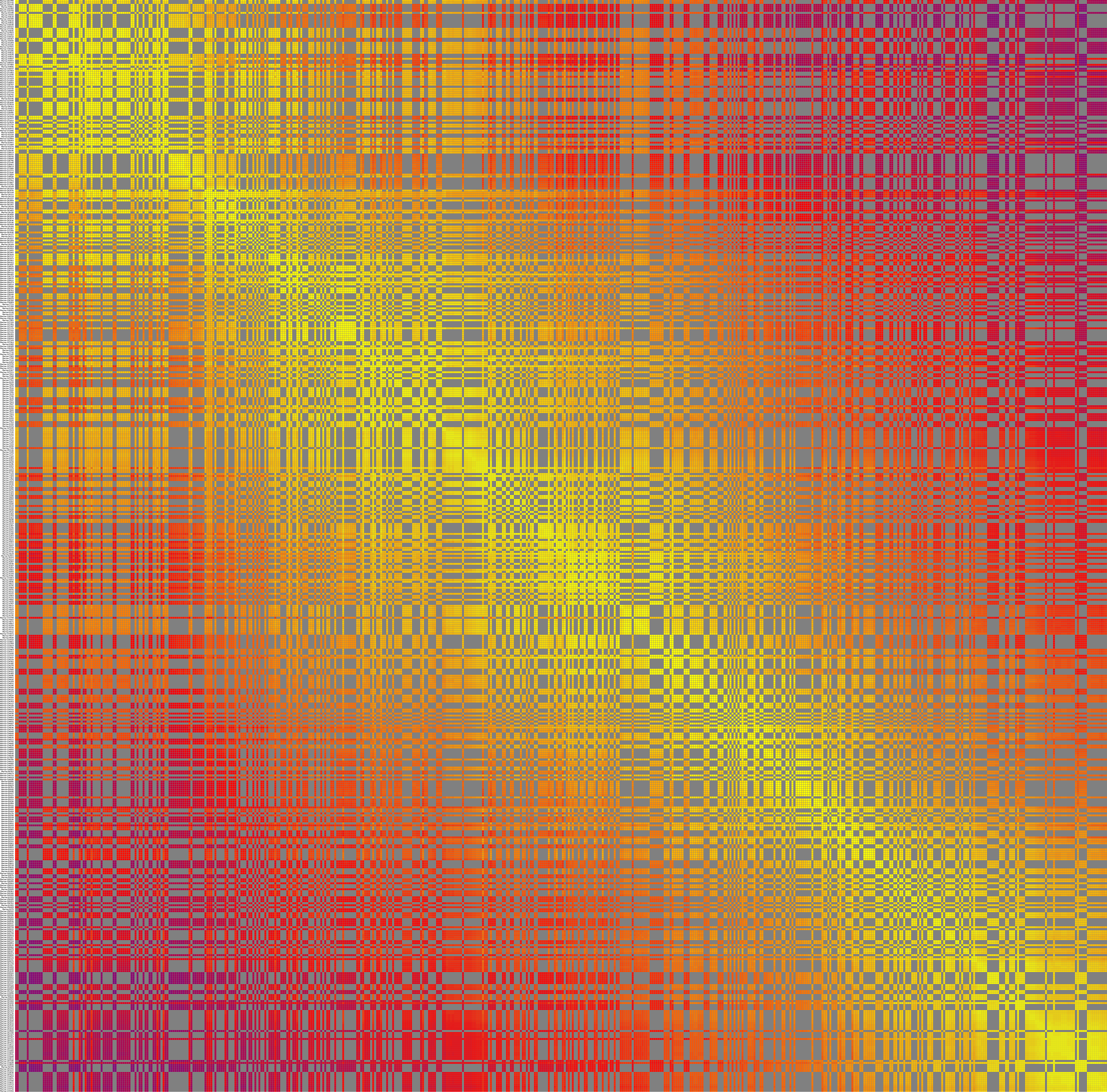

Supplement: Supplementary file 2 [file DataSheet_2.zip › Figure S6/sexAver/LG22.sexAver.heatMap.png]

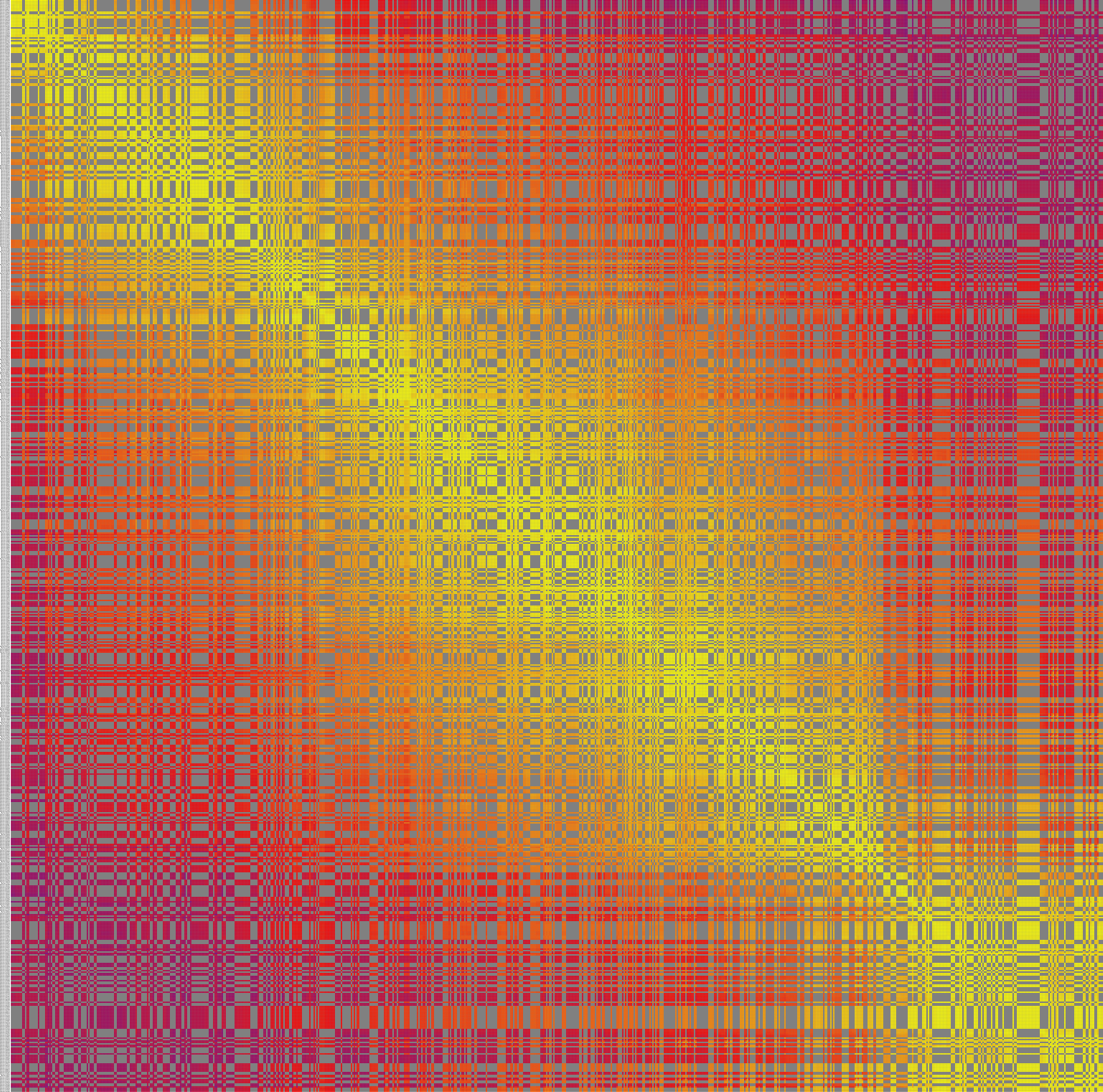

Supplement: Supplementary file 2 [file DataSheet_2.zip › Figure S6/sexAver/LG23.sexAver.heatMap.png]

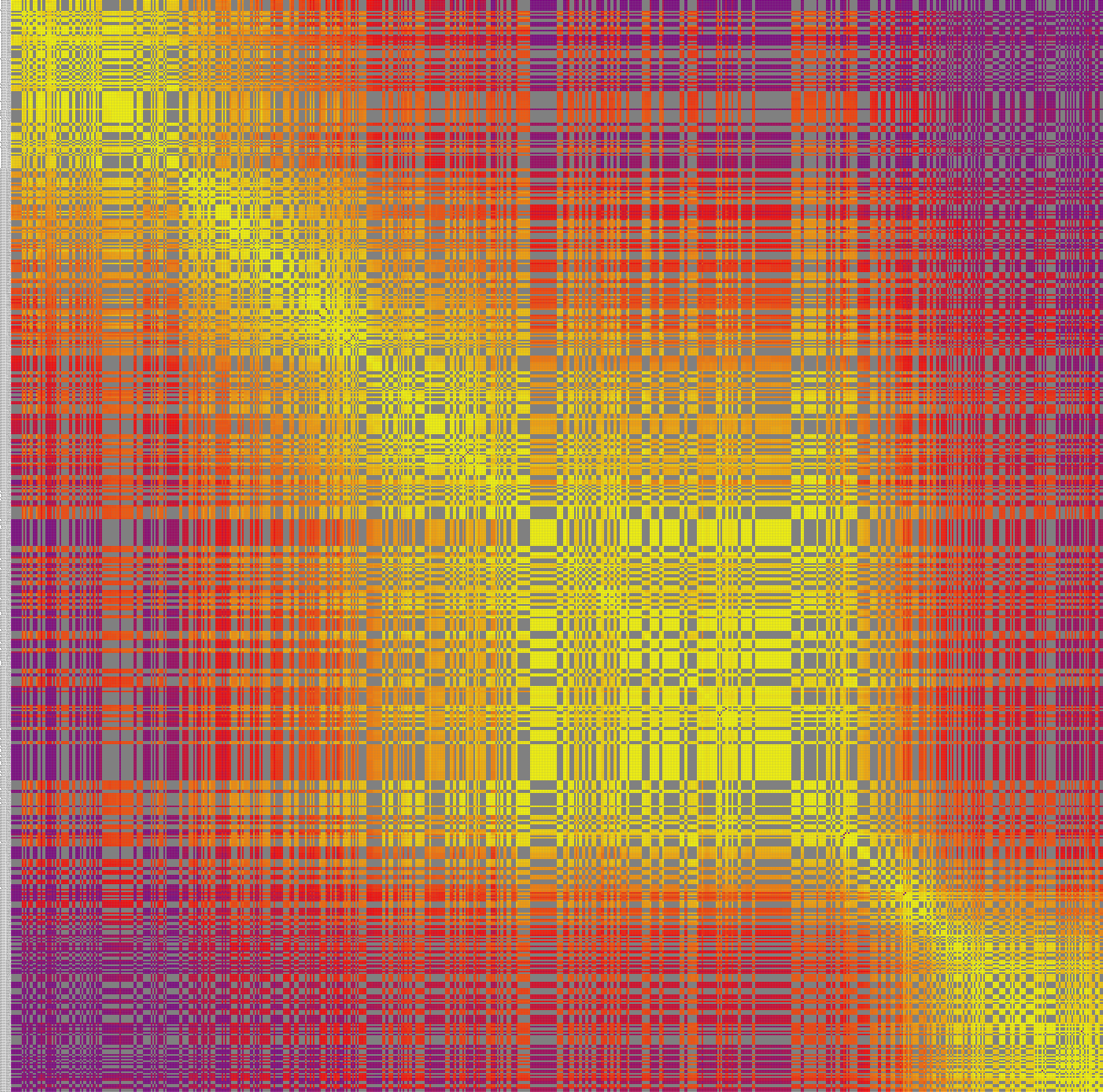

Supplement: Supplementary file 2 [file DataSheet_2.zip › Figure S6/sexAver/LG24.sexAver.heatMap.png]

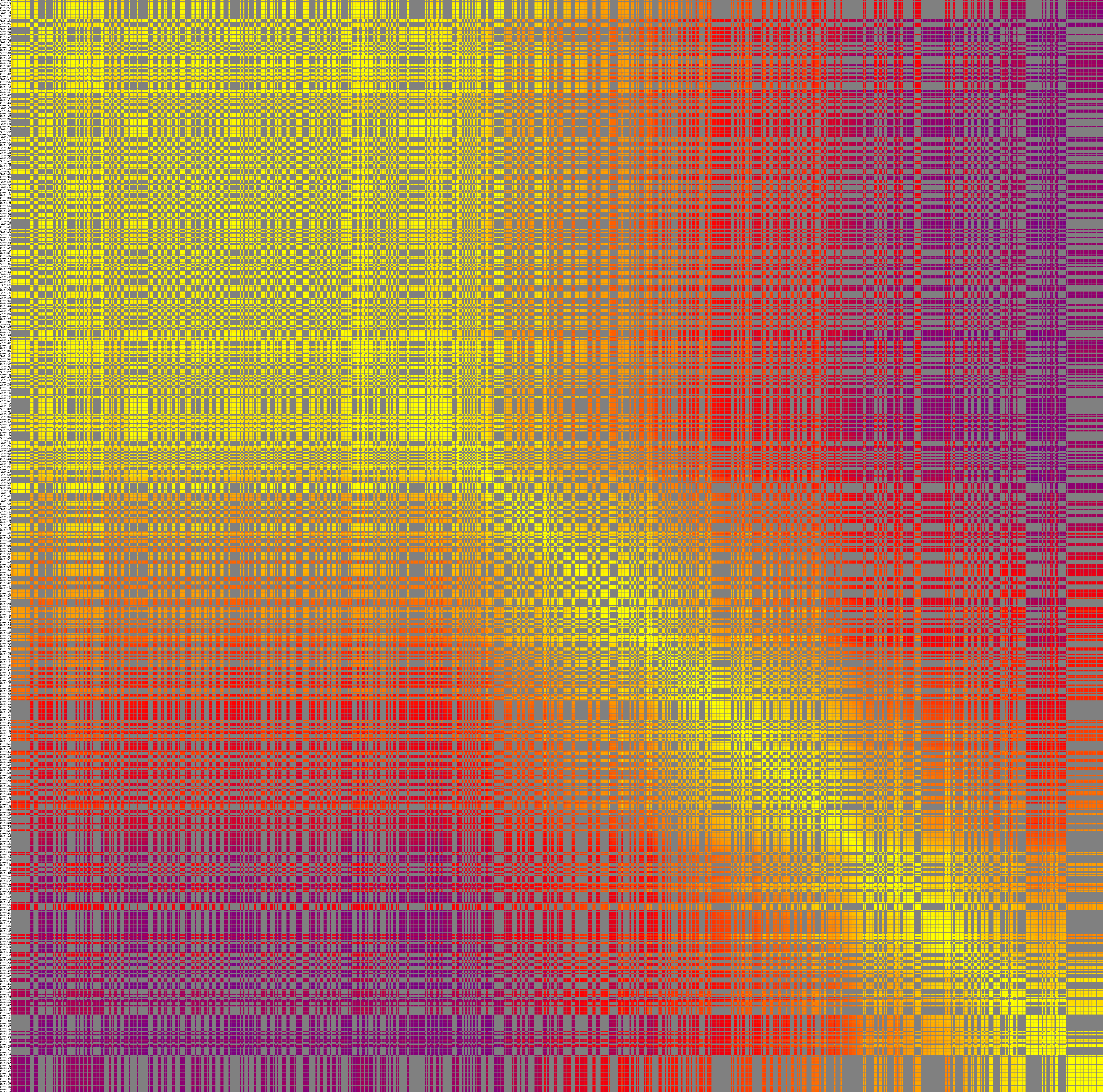

Supplement: Supplementary file 2 [file DataSheet_2.zip › Figure S6/sexAver/LG3.sexAver.heatMap.png]

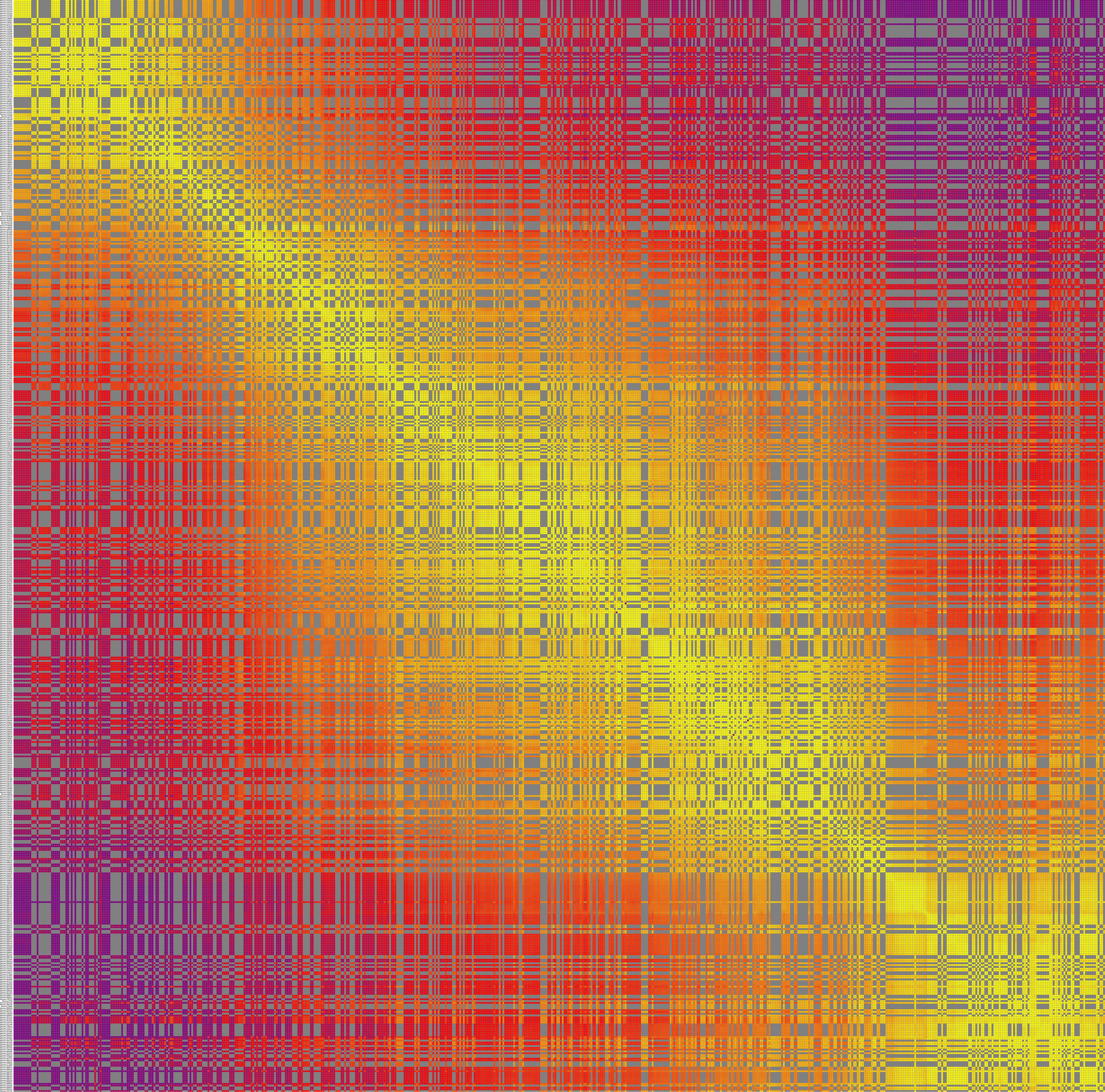

Supplement: Supplementary file 2 [file DataSheet_2.zip › Figure S6/sexAver/LG4.sexAver.heatMap.png]

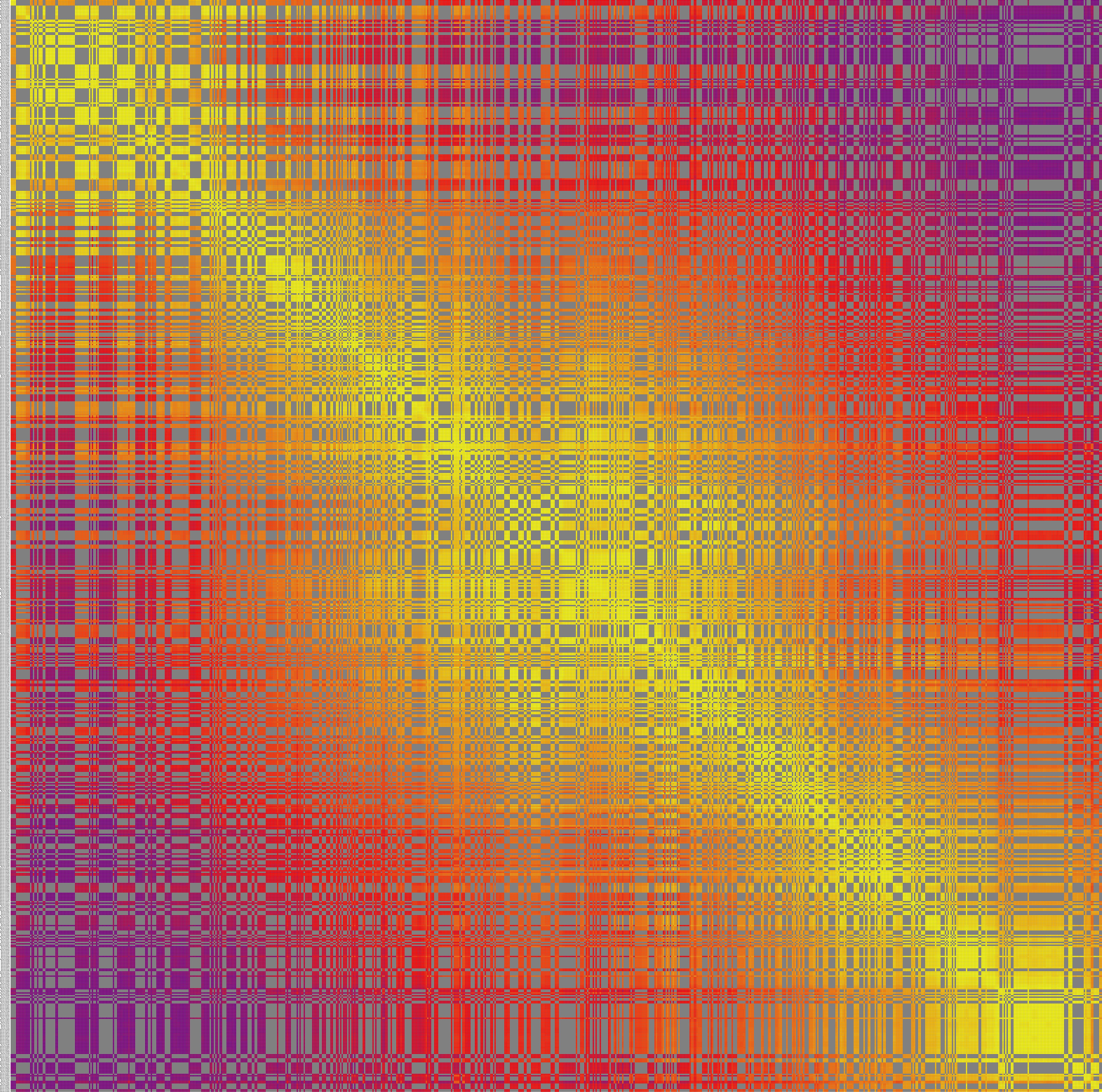

Supplement: Supplementary file 2 [file DataSheet_2.zip › Figure S6/sexAver/LG5.sexAver.heatMap.png]

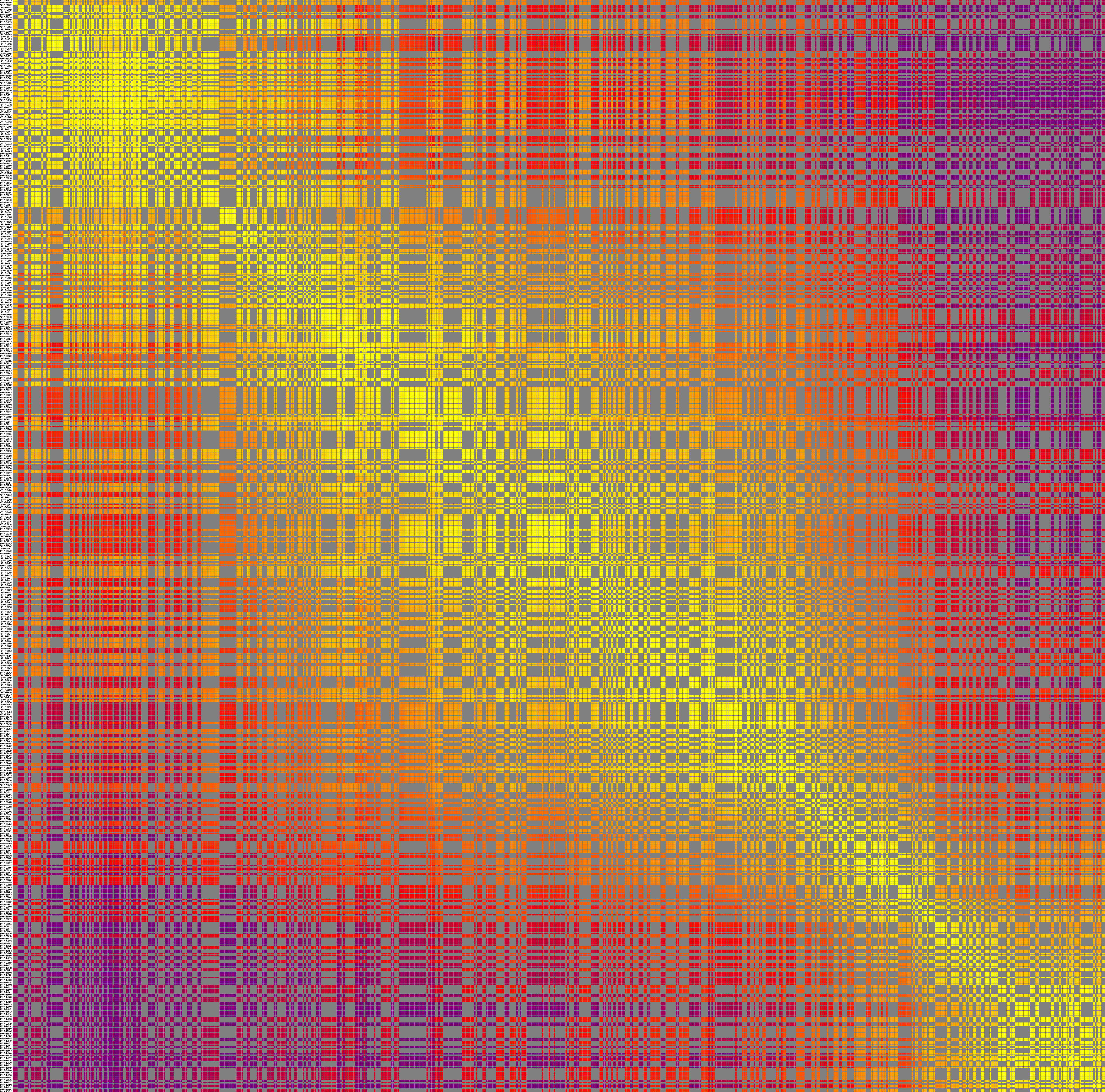

Supplement: Supplementary file 2 [file DataSheet_2.zip › Figure S6/sexAver/LG6.sexAver.heatMap.png]

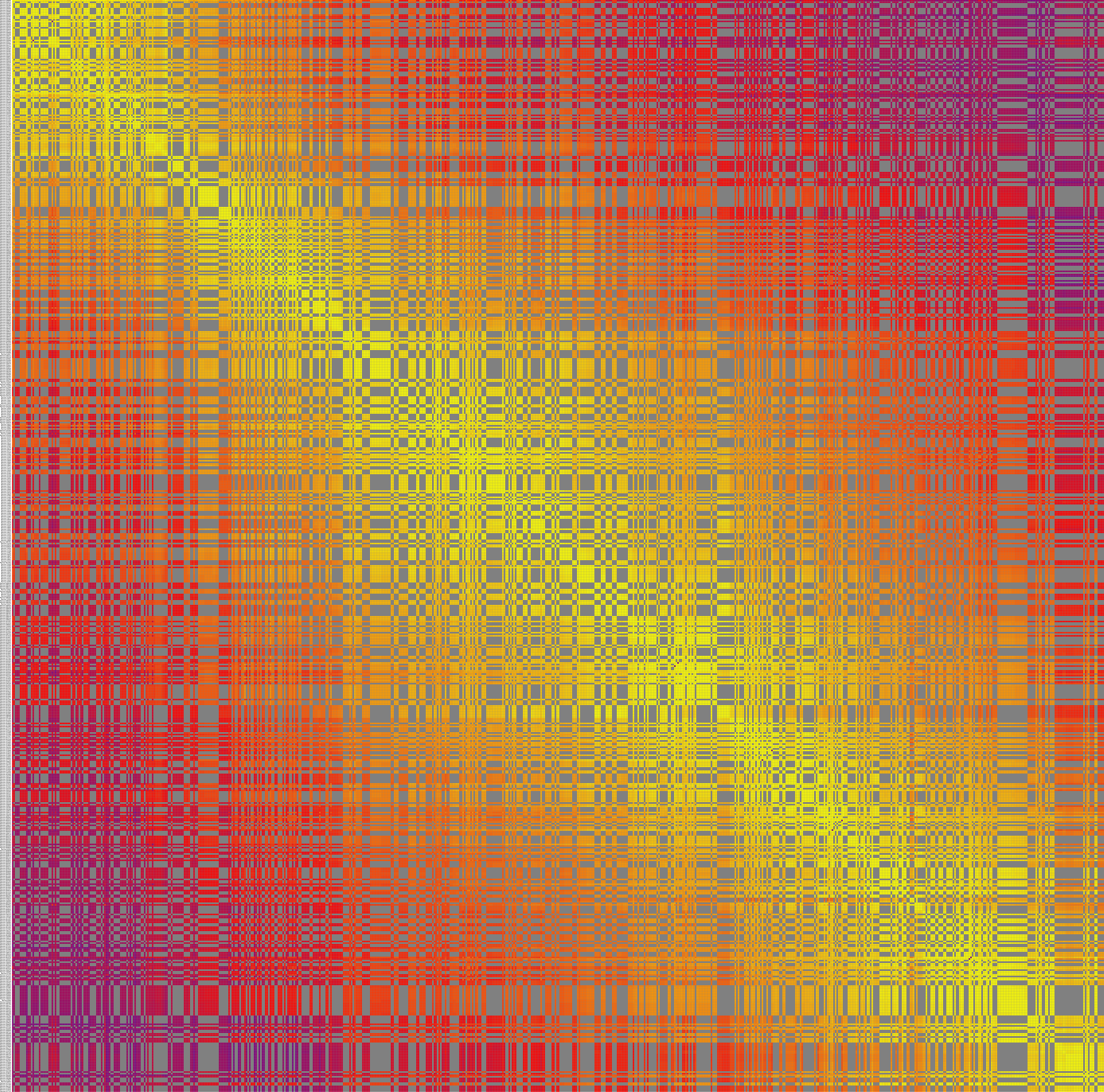

Supplement: Supplementary file 2 [file DataSheet_2.zip › Figure S6/sexAver/LG7.sexAver.heatMap.png]

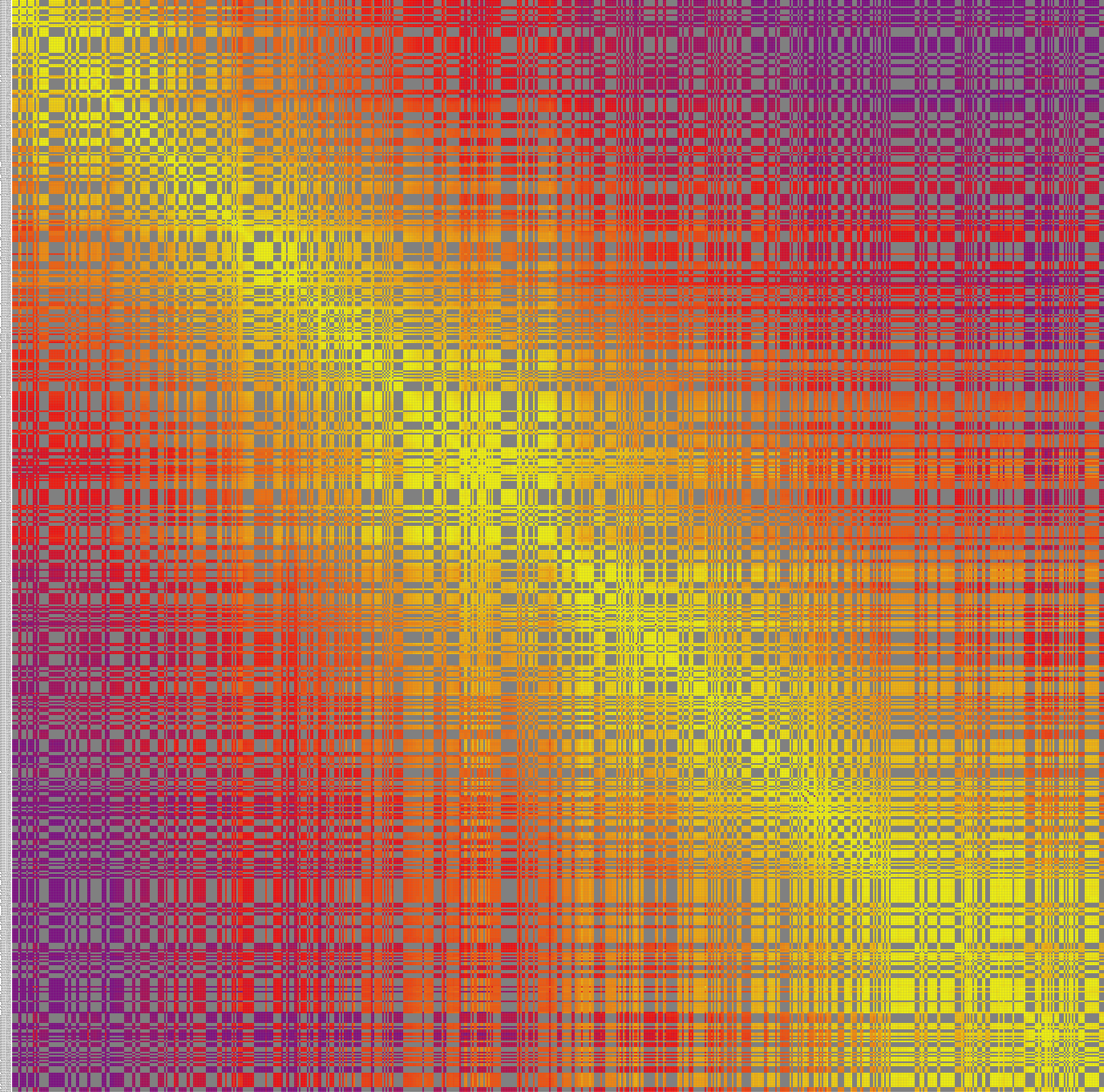

Supplement: Supplementary file 2 [file DataSheet_2.zip › Figure S6/sexAver/LG8.sexAver.heatMap.png]

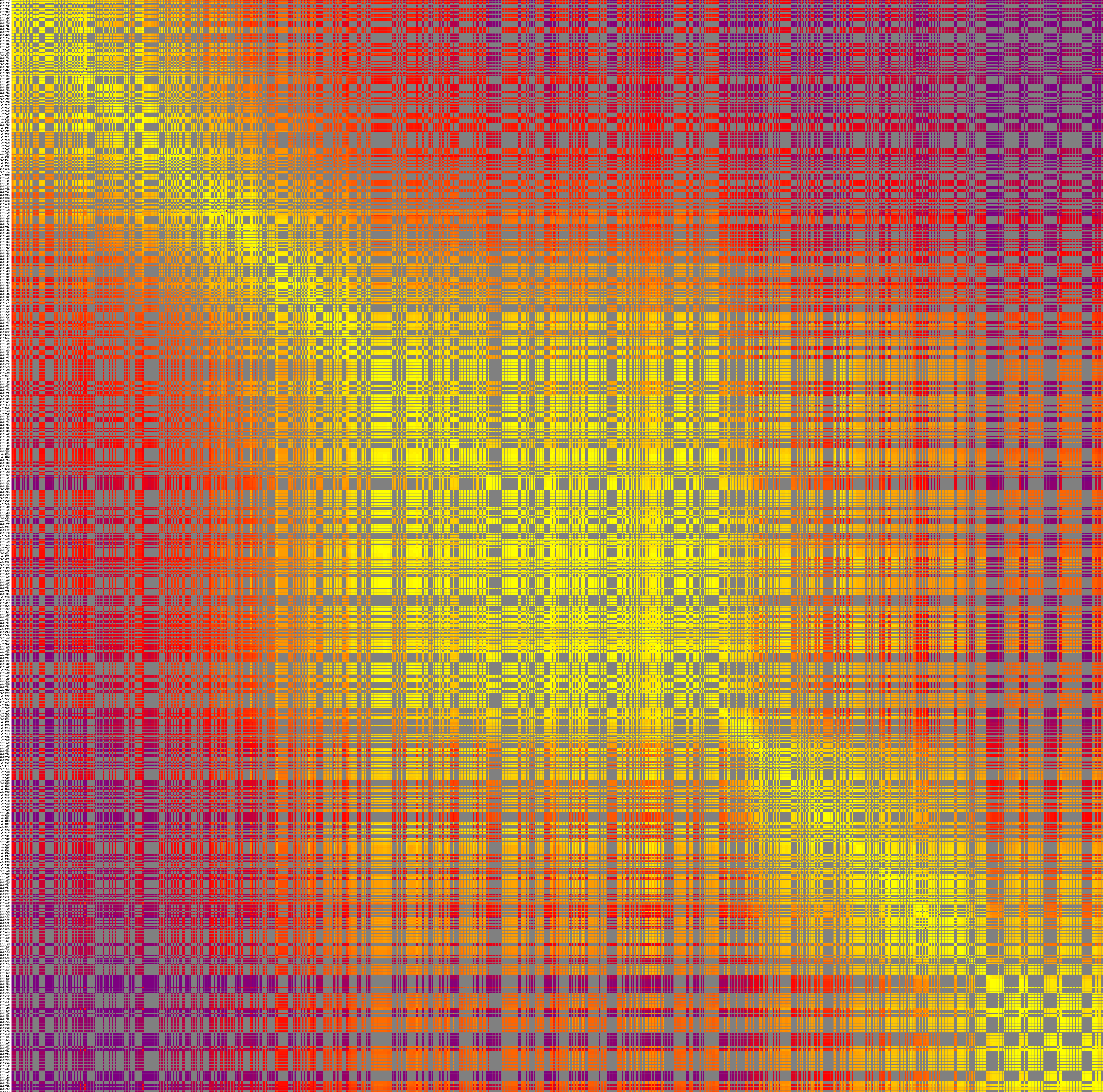

Supplement: Supplementary file 2 [file DataSheet_2.zip › Figure S6/sexAver/LG9.sexAver.heatMap.png]

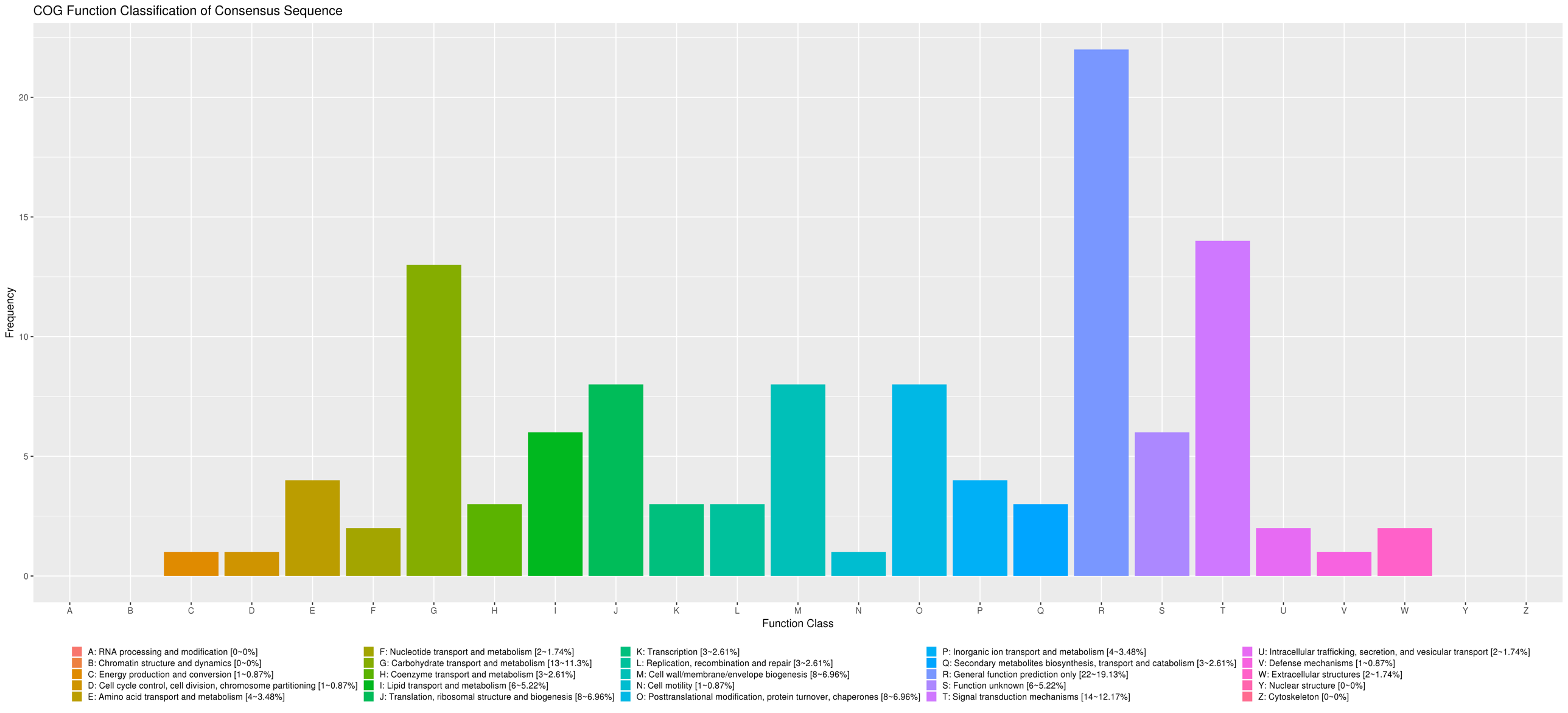

Supplement: Supplementary file 3 [file DataSheet_3.zip › Figure S7/Figure S7/GD/GD.Cog.classify.png]

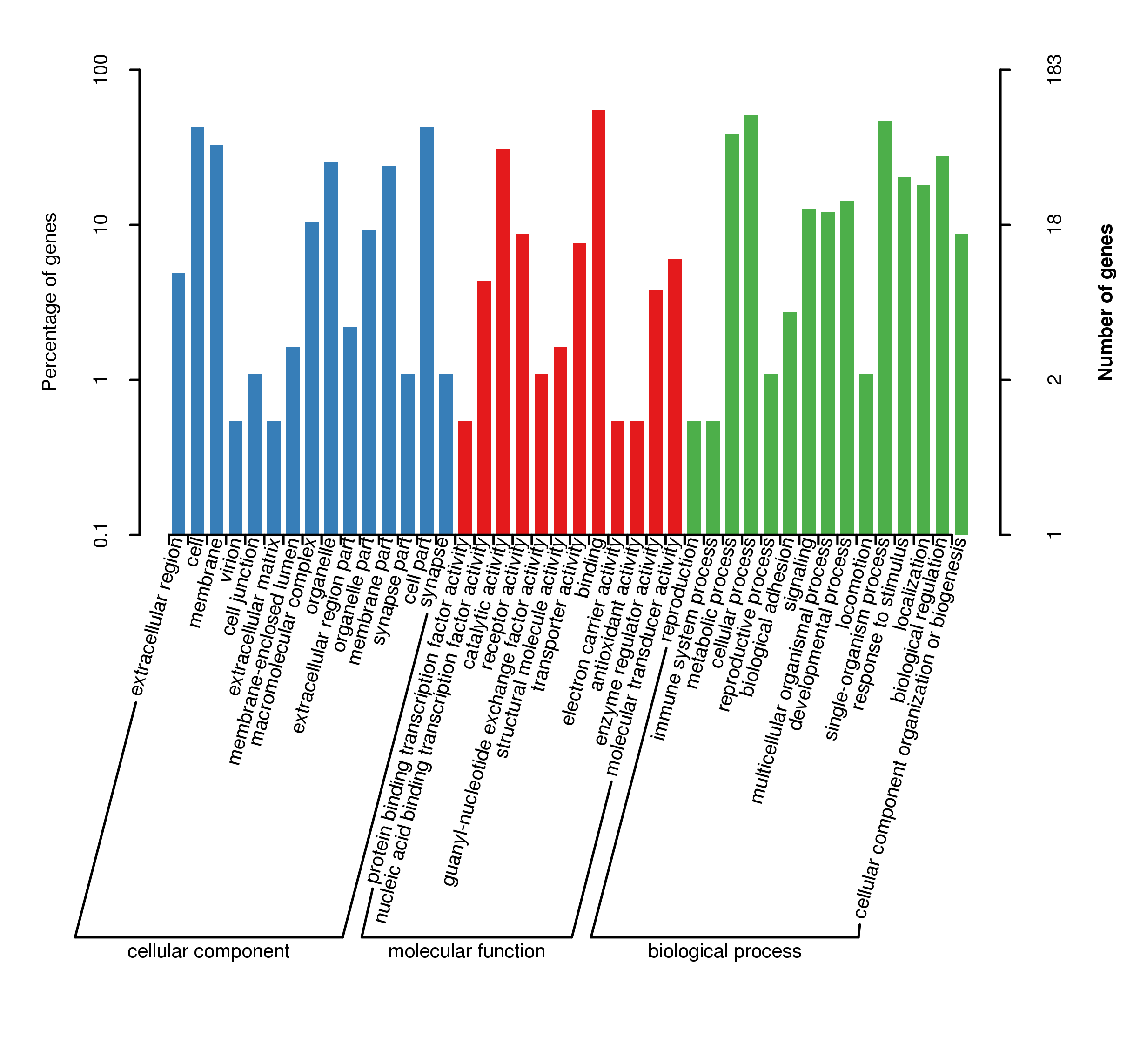

Supplement: Supplementary file 3 [file DataSheet_3.zip › Figure S7/Figure S7/GD/GD.GO.png]

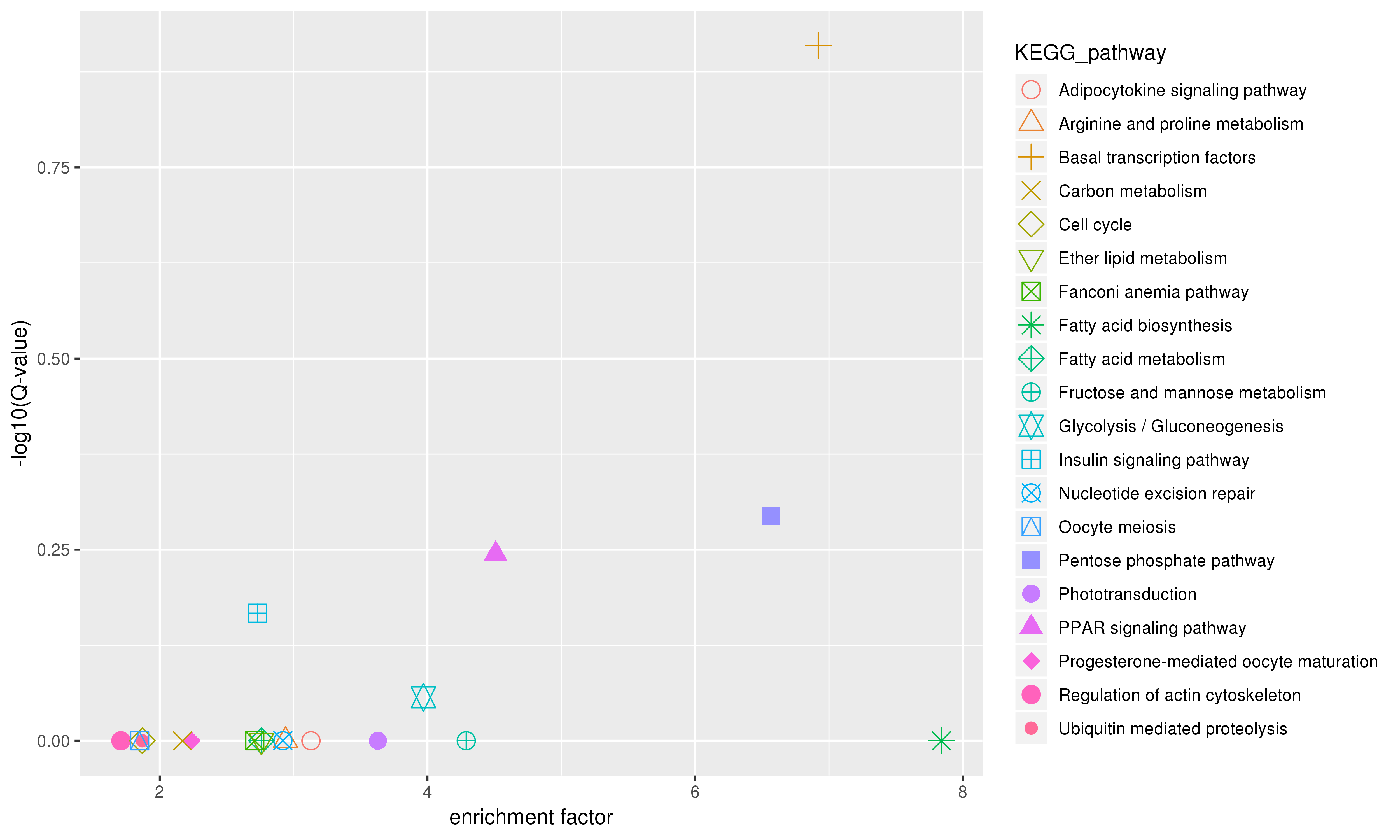

Supplement: Supplementary file 3 [file DataSheet_3.zip › Figure S7/Figure S7/GD/GD.KEGG.Phase.png]

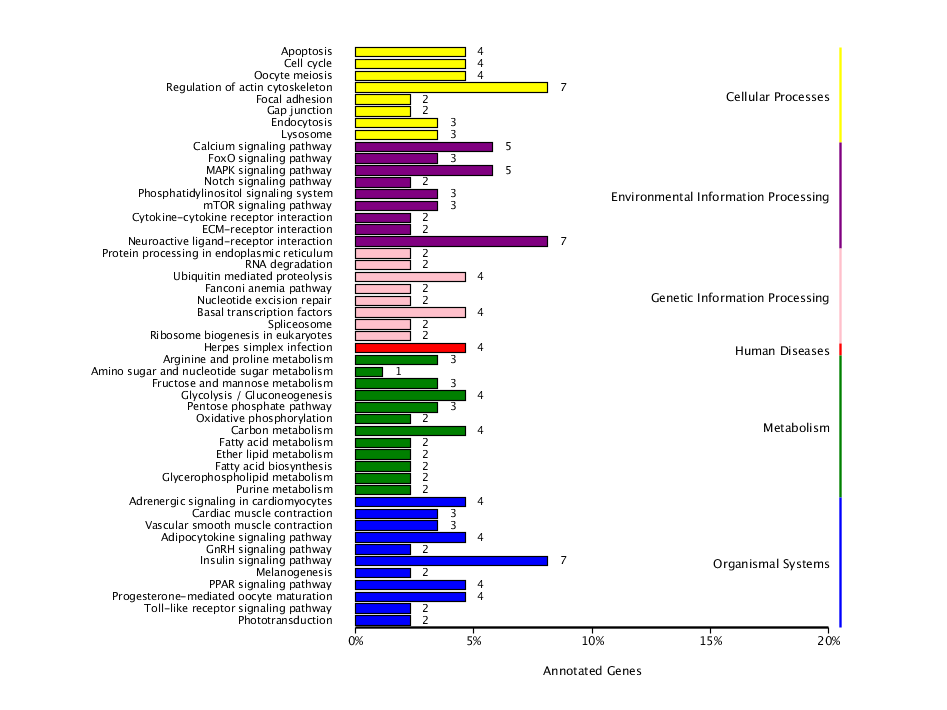

Supplement: Supplementary file 3 [file DataSheet_3.zip › Figure S7/Figure S7/GD/GD.KEGG.png]

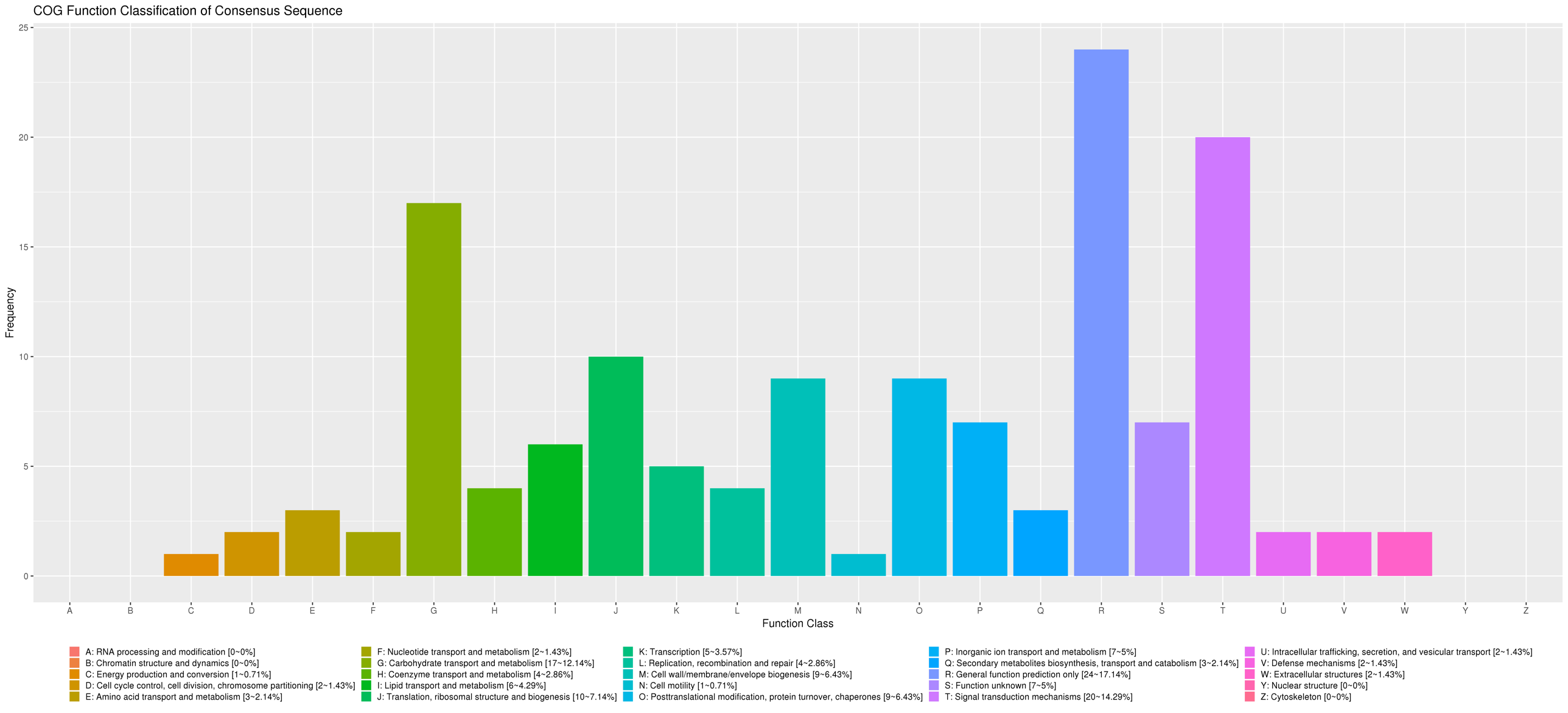

Supplement: Supplementary file 3 [file DataSheet_3.zip › Figure S7/Figure S7/MC/MC.Cog.classify.png]

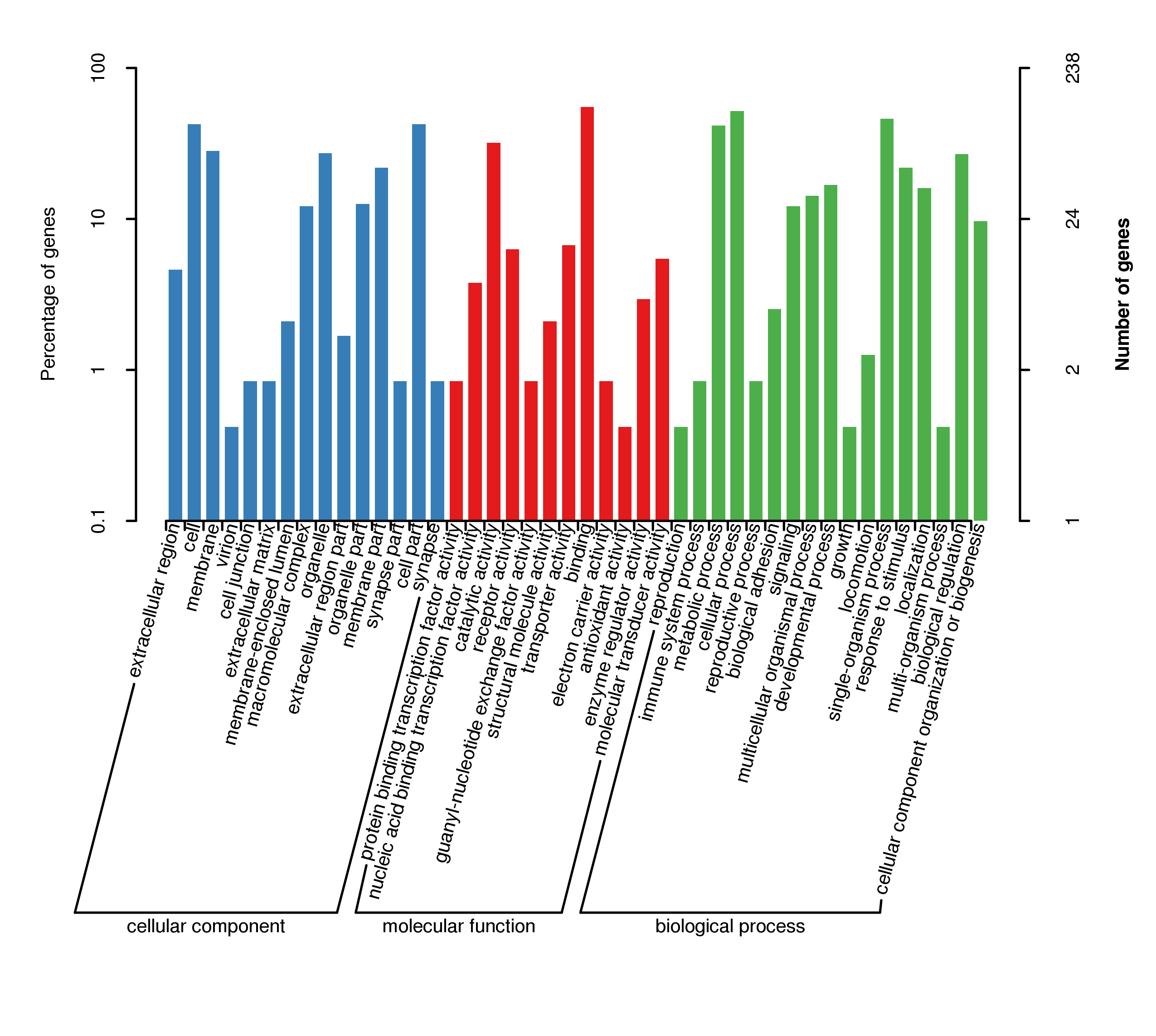

Supplement: Supplementary file 3 [file DataSheet_3.zip › Figure S7/Figure S7/MC/MC.GO.png]

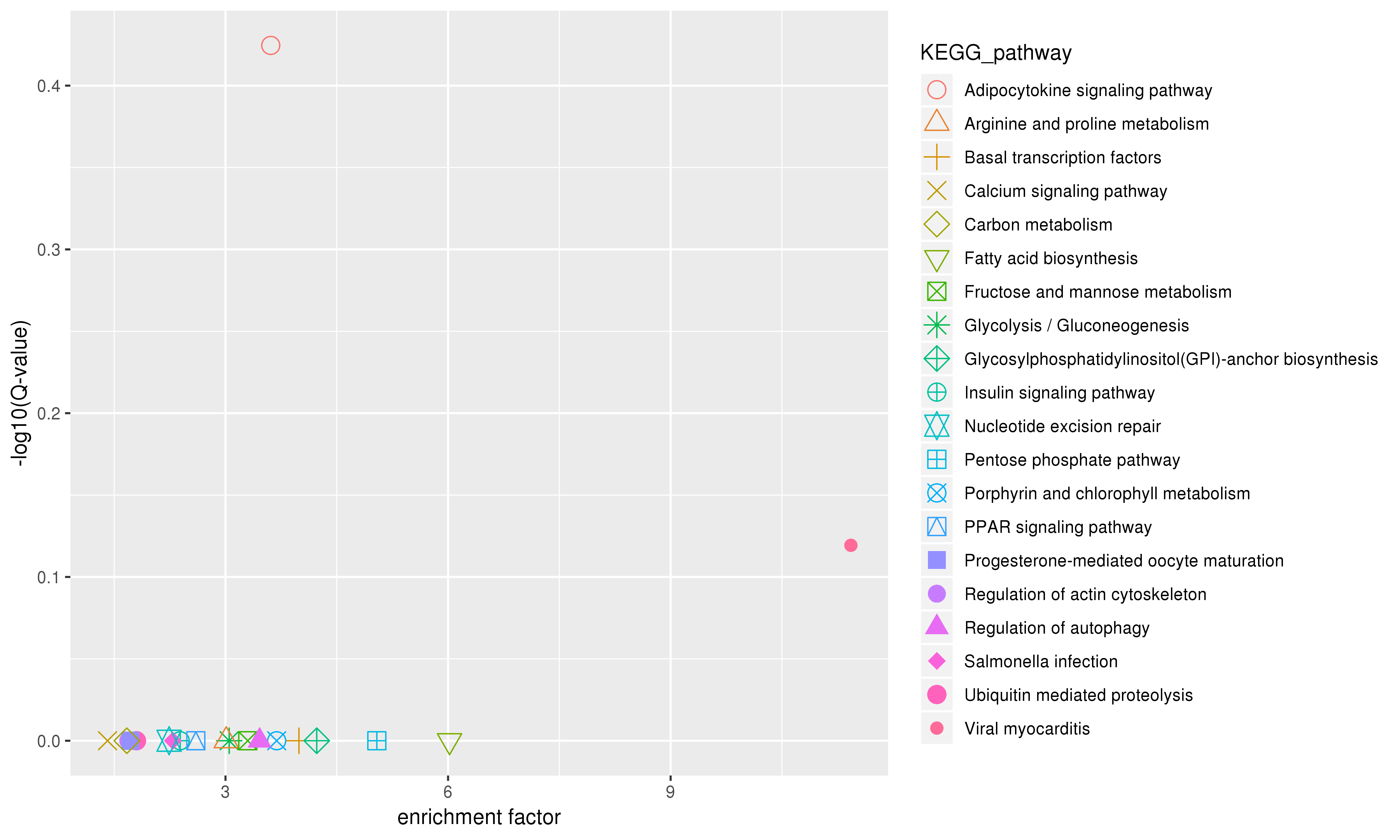

Supplement: Supplementary file 3 [file DataSheet_3.zip › Figure S7/Figure S7/MC/MC.KEGG.Phase.png]

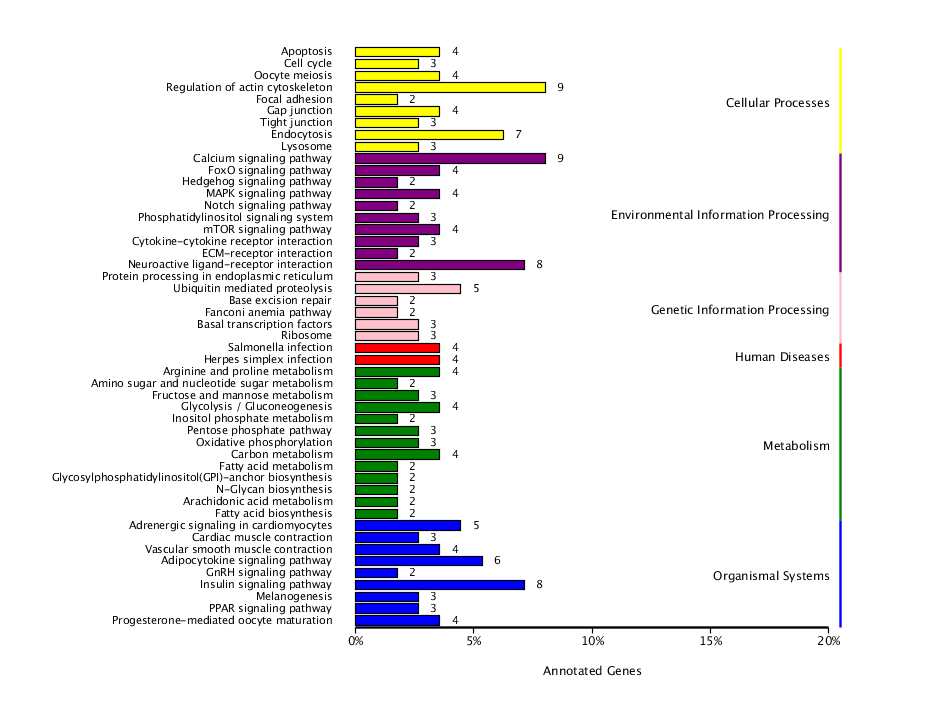

Supplement: Supplementary file 3 [file DataSheet_3.zip › Figure S7/Figure S7/MC/MC.KEGG.png]

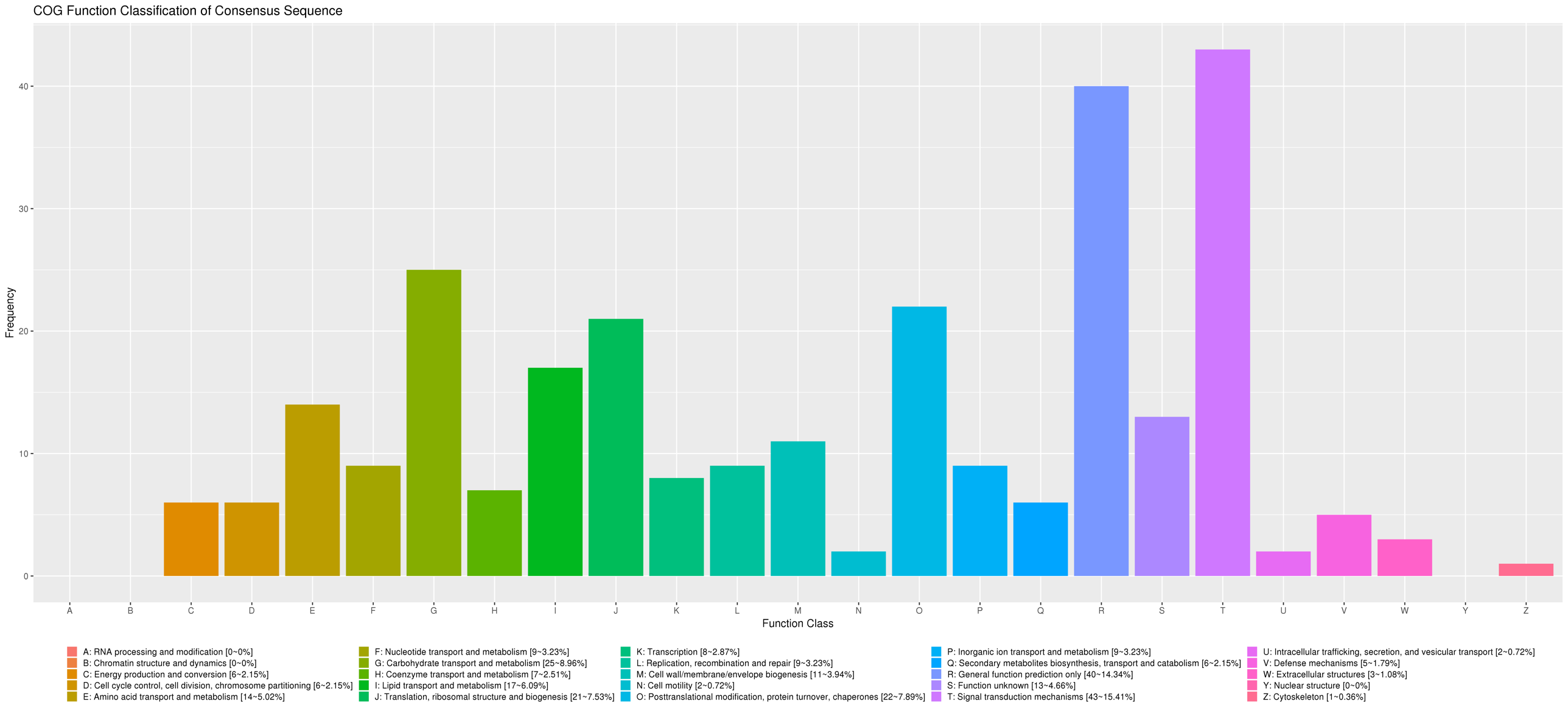

Supplement: Supplementary file 3 [file DataSheet_3.zip › Figure S7/Figure S7/SGD/SGD.Cog.classify.png]

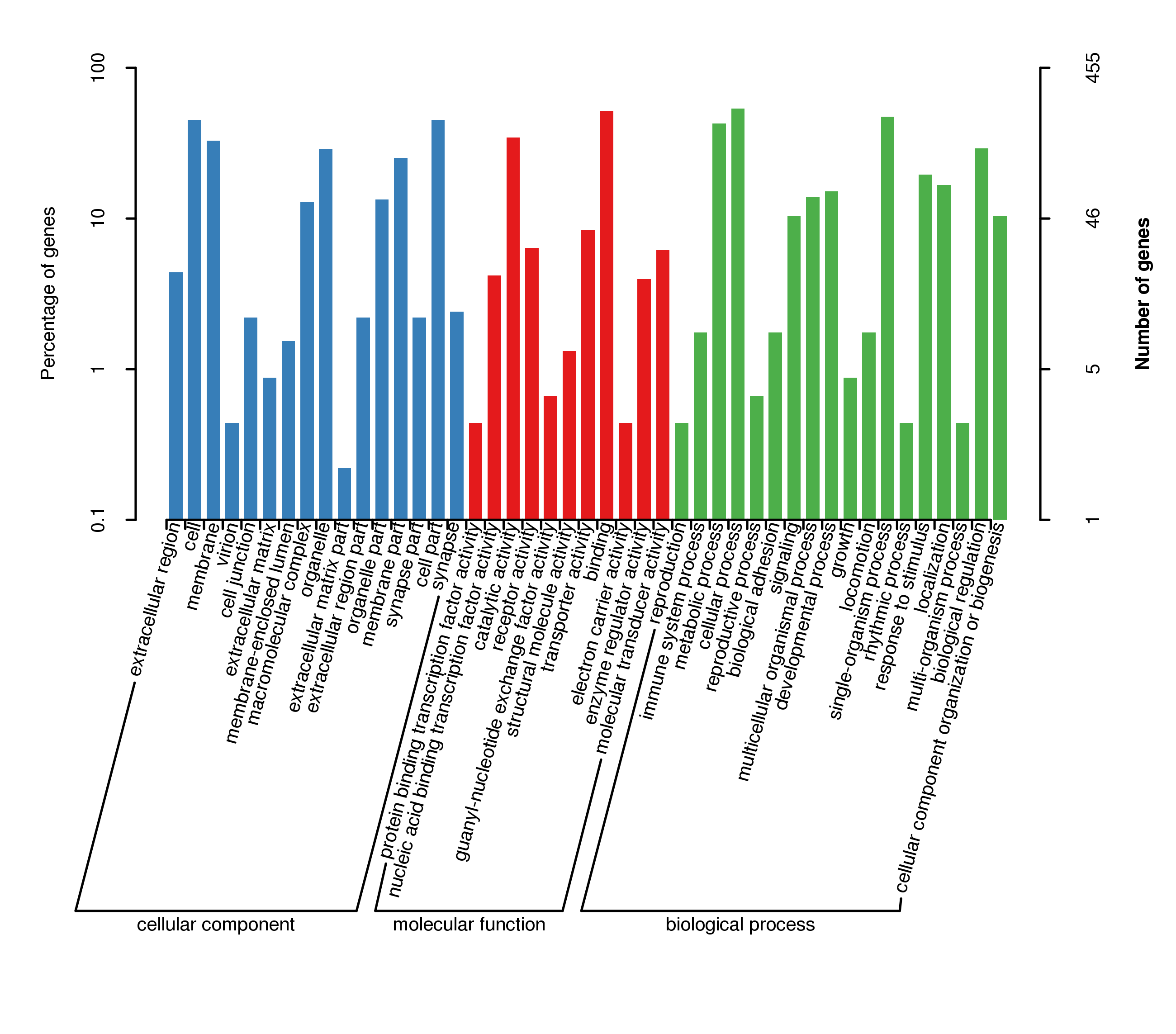

Supplement: Supplementary file 3 [file DataSheet_3.zip › Figure S7/Figure S7/SGD/SGD.GO.png]

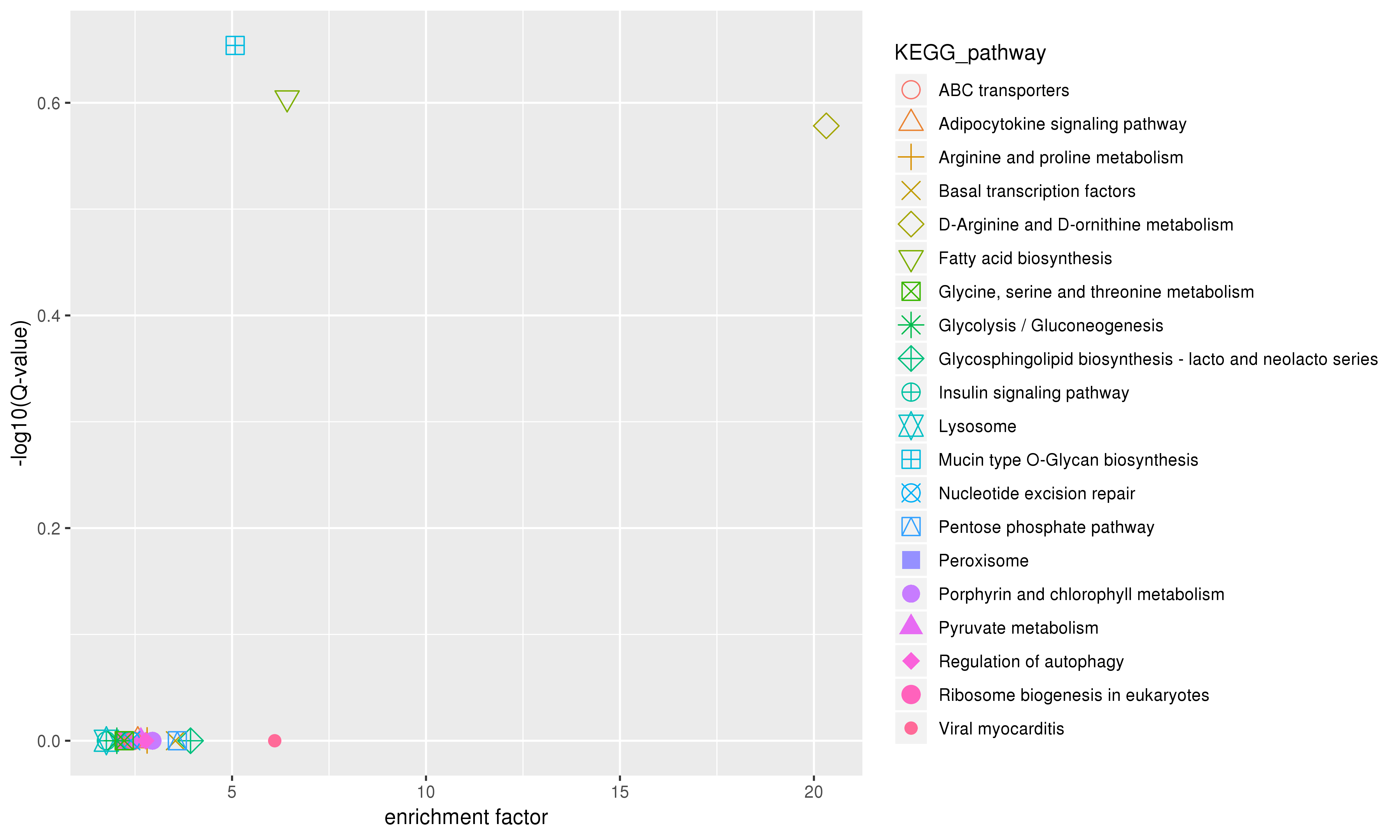

Supplement: Supplementary file 3 [file DataSheet_3.zip › Figure S7/Figure S7/SGD/SGD.KEGG.Phase.png]

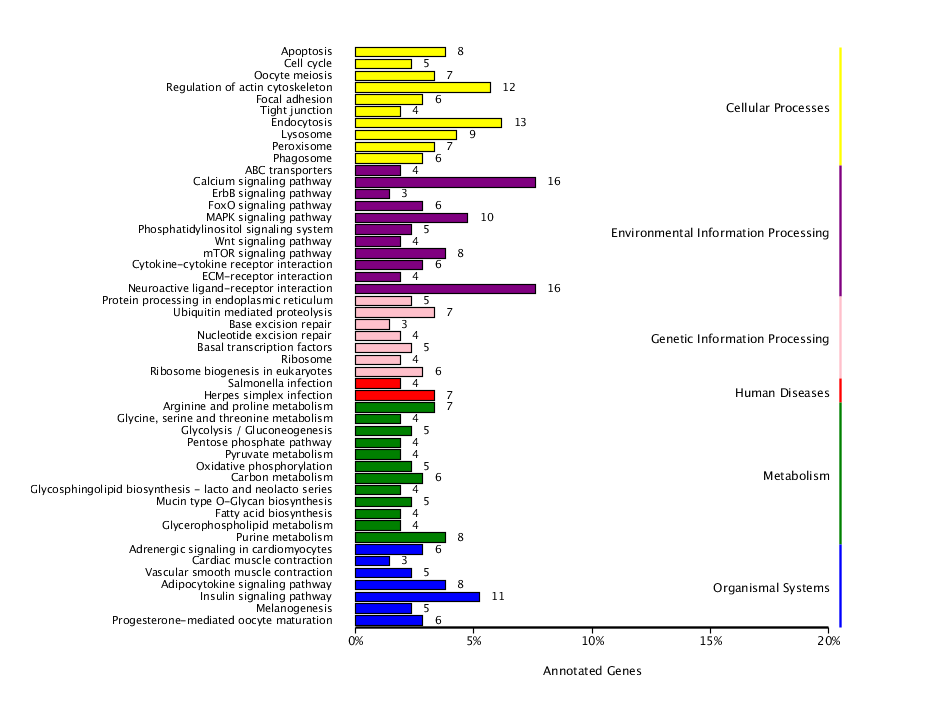

Supplement: Supplementary file 3 [file DataSheet_3.zip › Figure S7/Figure S7/SGD/SGD.KEGG.png]

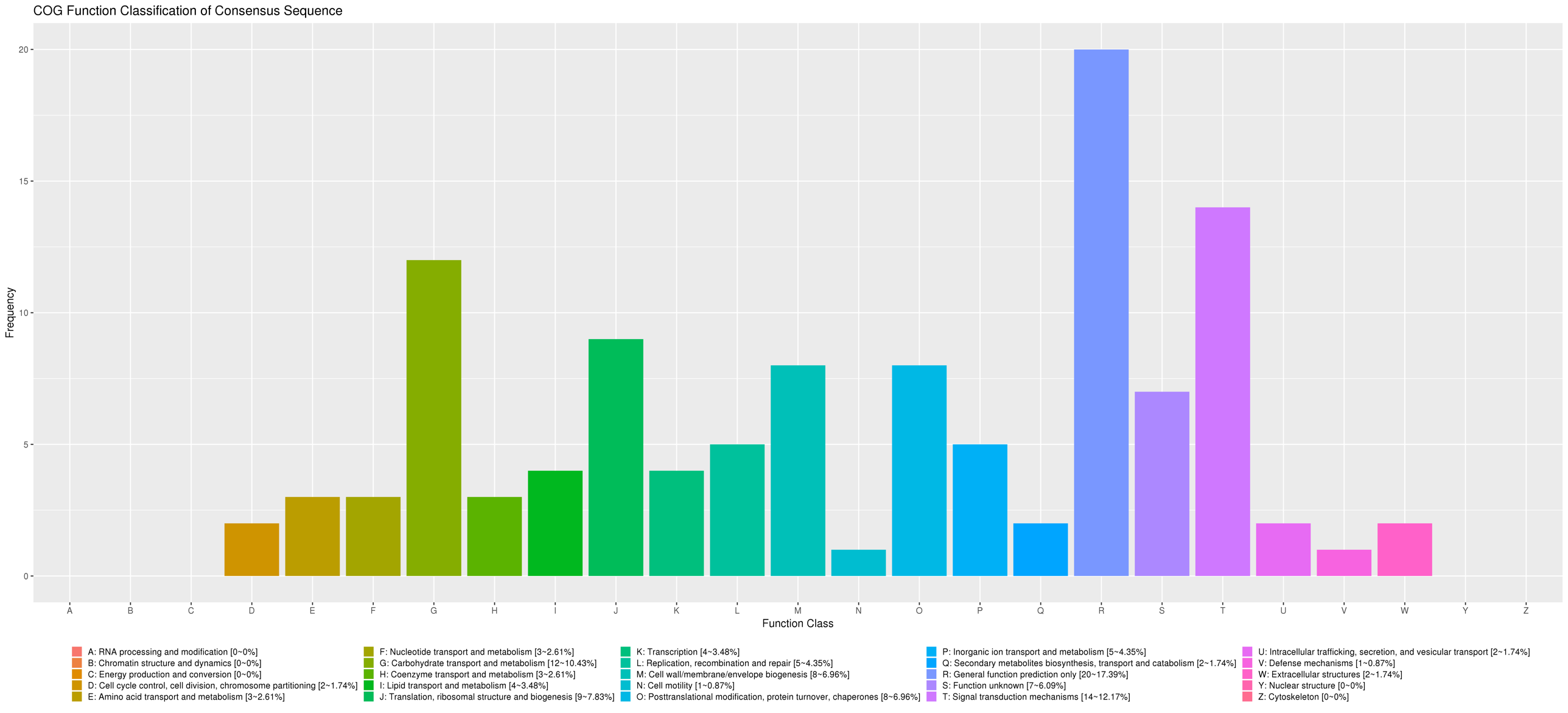

Supplement: Supplementary file 3 [file DataSheet_3.zip › Figure S7/Figure S7/WG/WG.Cog.classify.png]

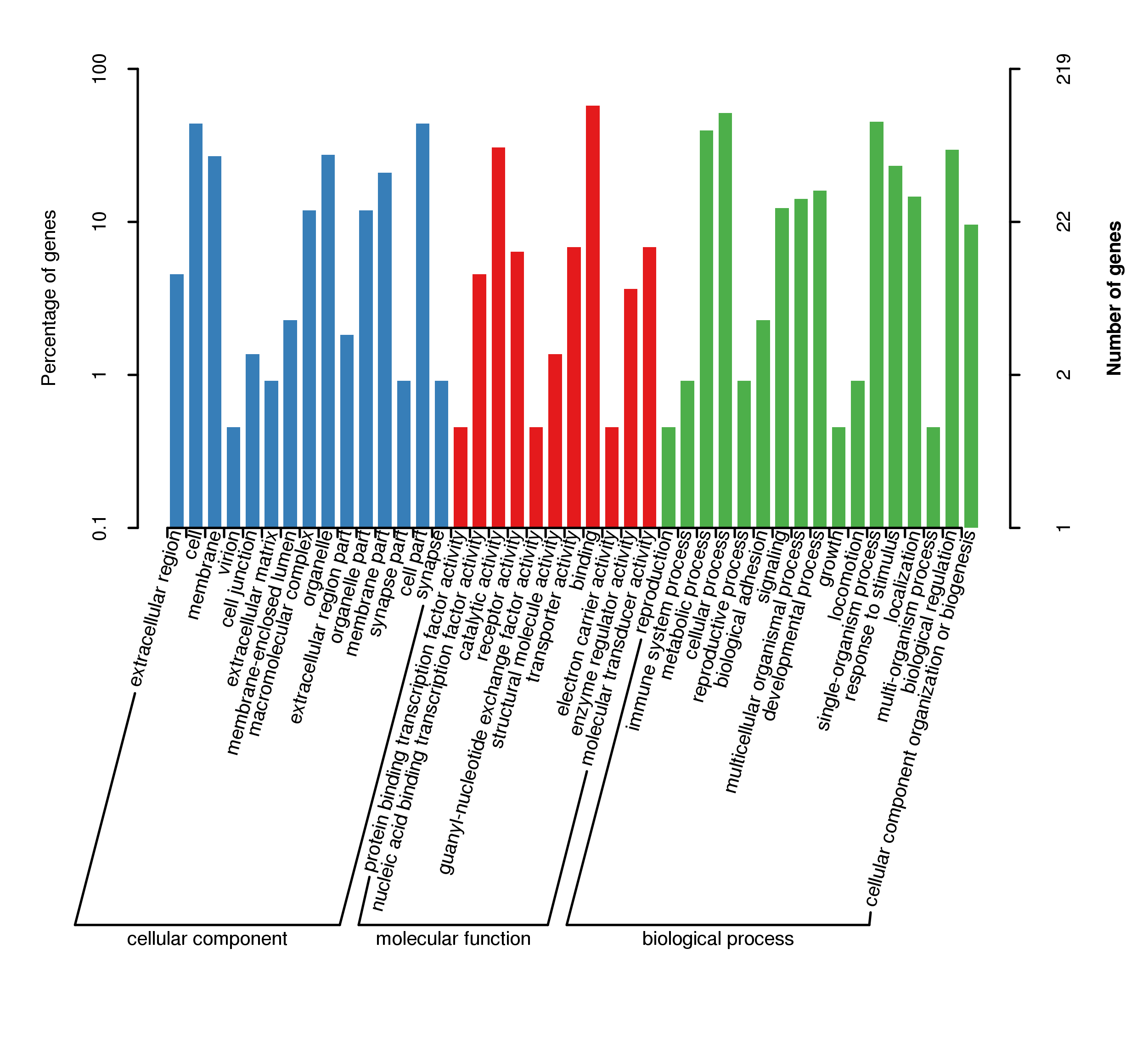

Supplement: Supplementary file 3 [file DataSheet_3.zip › Figure S7/Figure S7/WG/WG.GO.png]

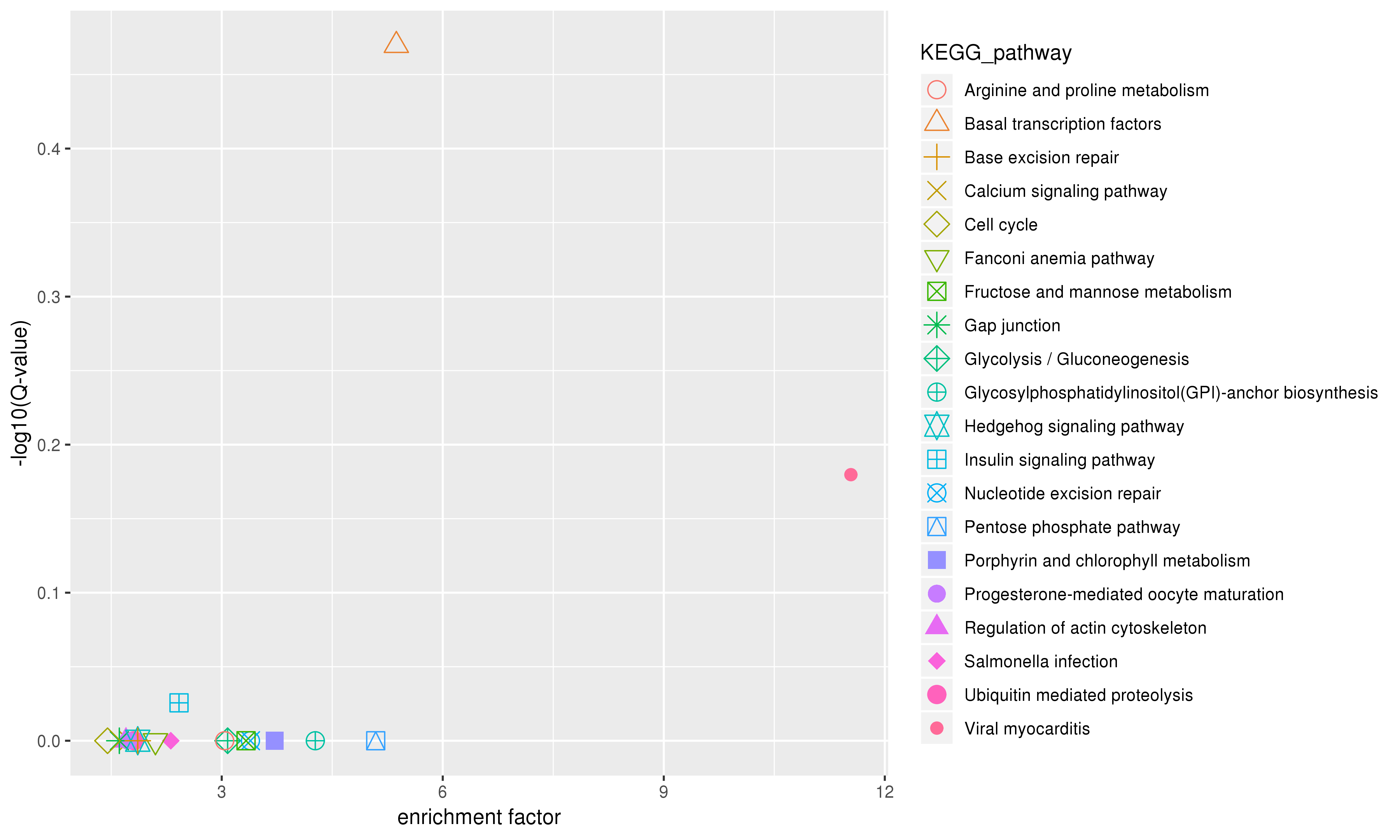

Supplement: Supplementary file 3 [file DataSheet_3.zip › Figure S7/Figure S7/WG/WG.KEGG.Phase.png]

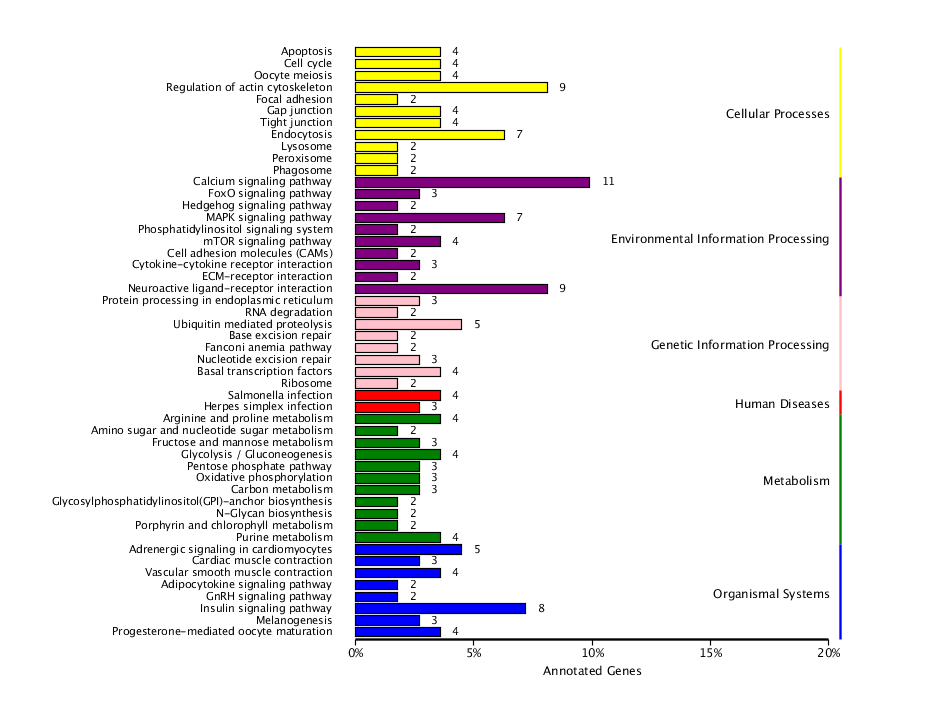

Supplement: Supplementary file 3 [file DataSheet_3.zip › Figure S7/Figure S7/WG/WG.KEGG.png]

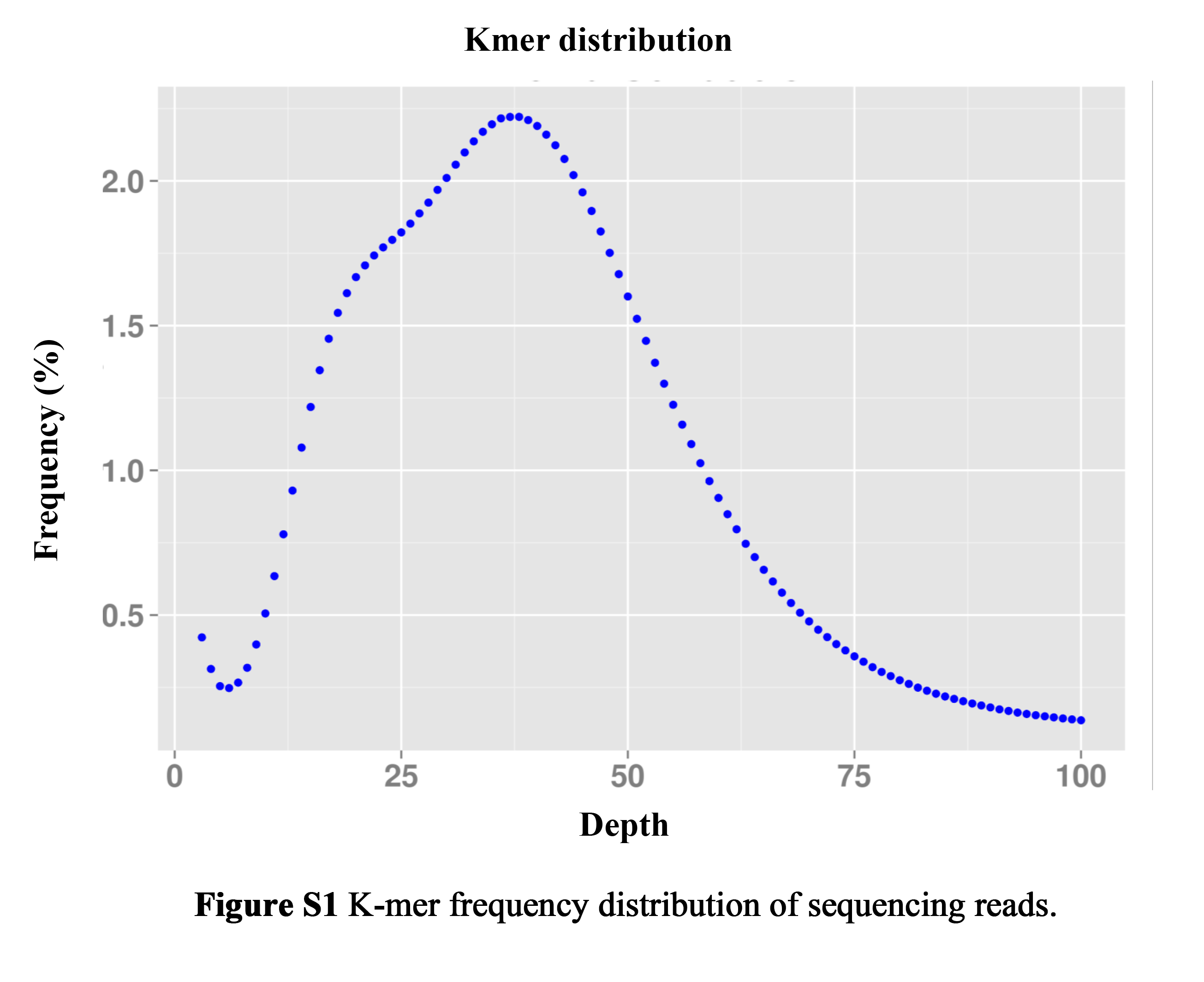

Supplement: Supplementary file 4 [file Image_1.png]

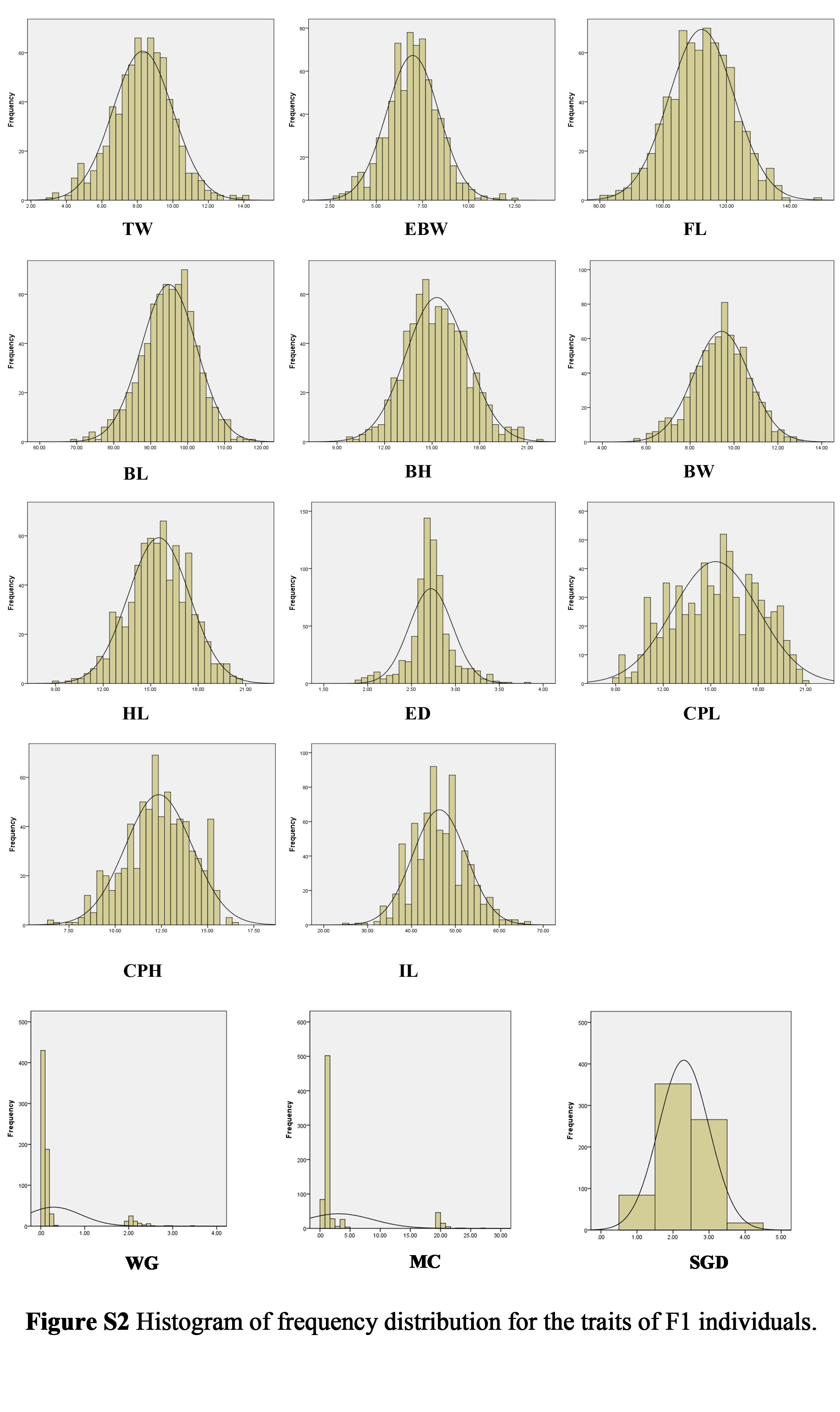

Supplement: Supplementary file 5 [file Image_2.jpg]

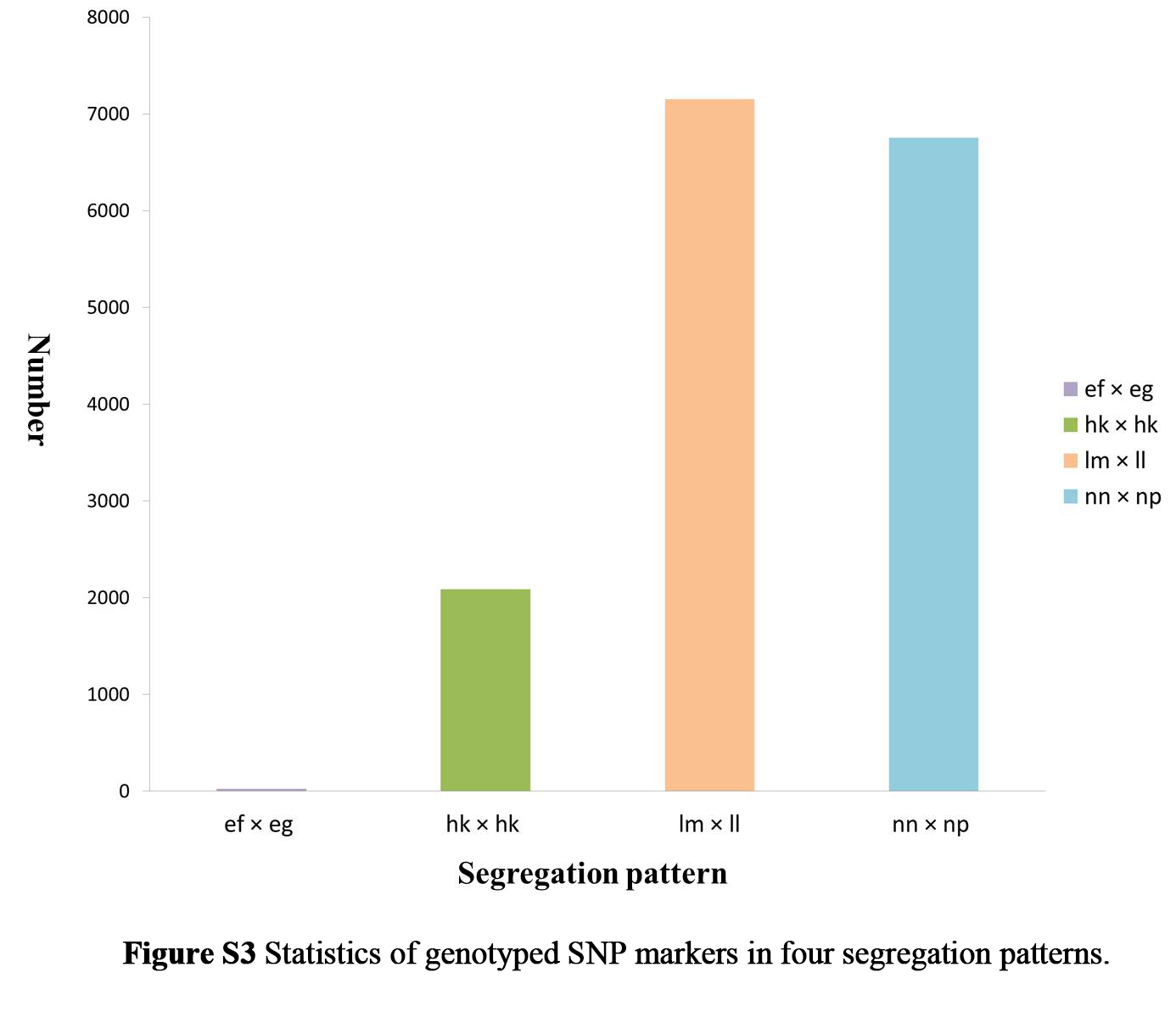

Supplement: Supplementary file 6 [file Image_3.jpg]

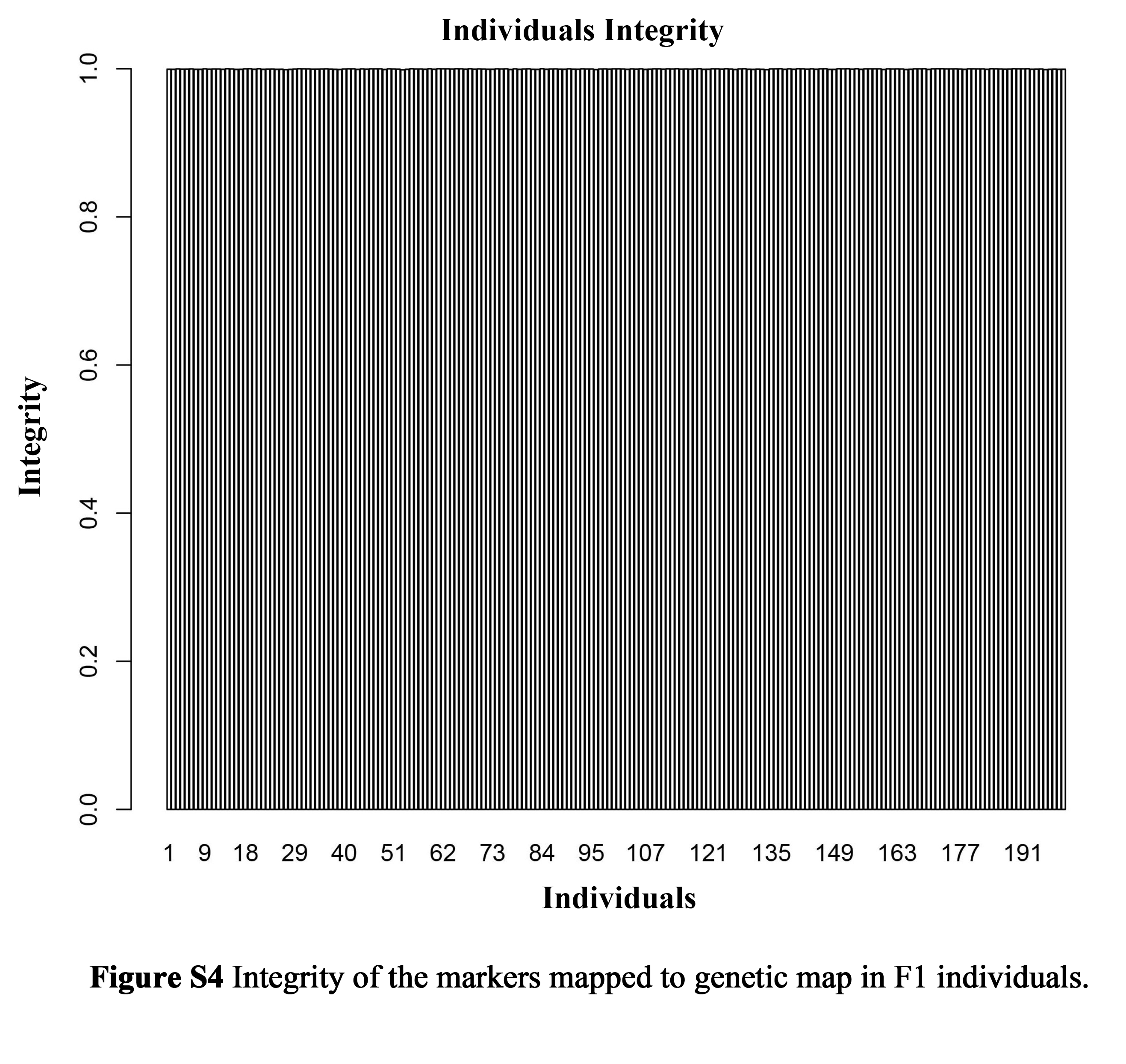

Supplement: Supplementary file 7 [file Image_4.png]
