# Supplementary material for: A High-Density Genetic Linkage Map and QTL Mapping for Sex and Growth-Related Traits of Large-Scale Loach (Paramisgurnus dabryanus)
Source: Front Genet. 2019 Oct 25;10:1023. doi: 10.3389/fgene.2019.01023 (PMC6823184; doi:10.3389/fgene.2019.01023)
Supplement: Supplementary file 3 [file DataSheet_3.zip › Caption and Description of Figure S7.docx]

**File name:** Figure S7

**File format:** ZIP

**Caption of data:** Function enrichment analyses of candidate genes associated with sex.

**Description of data:** The files named ‘Cog.classify’ represent the COG classification of candidate genes associated with sex; The files named ‘GO’ represent the GO classification of candidate genes associated with sex; the files named ‘KEGG’ represent the distribution of sex-related candidate genes annotated in KEGG pathways; the files named ‘KEGG.Phase’ represent the clustering degree of sex-related candidate genes annotated in KEGG pathways.
