# Supplementary material for: A High-Density Genetic Linkage Map and QTL Mapping for Sex and Growth-Related Traits of Large-Scale Loach (Paramisgurnus dabryanus)
Source: Front Genet. 2019 Oct 25;10:1023. doi: 10.3389/fgene.2019.01023 (PMC6823184; doi:10.3389/fgene.2019.01023)
Supplement: Supplementary file 8 [file Presentation_1.pdf]

A High-Density Genetic Linkage Map and QTL Mapping for Sex and Growth-Related Traits of Large-Scale Loach (*Paramisgurnus dabryanus*)

Jin Wei[a], Yuanyuan Chen[a], Weimin Wang*

E-mail Addresses:

Jin Wei[a]: weijin@webmail.hzau.edu.cn

Yuanyuan Chen[a]: chenyuan@webmail.hzau.edu.cn.com

Current Addresses:

[a] College of Fisheries, Key Lab of Agricultural Animal Genetics, Breeding and Reproduction of Ministry of Education/Key Lab of Freshwater Animal Breeding, Ministry of Agriculture, Huazhong Agricultural University, Wuhan, Hubei 430070, China

***Corresponding author: Weimin Wang, PhD, Professor**

E-mail address: wangwm@mail.hzau.edu.cn; **Tel:** +86-27-87284292; **Fax:** +86-27-87284292

***Current address:** College of Fisheries, Huazhong Agricultural University, Wuhan 430070, P. R. China

Table S1 The trait distributions and the gender differences in growth traits of the F1 individuals

	Female (mean±SD)	Male (mean±SD)	*P*-value (t-test)	Total (mean±SD)	Sample (mean±SD)	Normality test		
						***P*-value**	**Skewness**	**Kurtosis**
**Number**	412	307	/	719	200	719	719	719
**TW/g**	8.64±1.42**	7.66±1.51	0.000	8.30±1.69	7.36 ± 2.44	0.063	-0.062	0.509
**EBW/g**	7.27±1.25**	6.44±1.24	0.000	6.97±1.42	6.41 ± 2.00	0.053	0.058	0.716
**FL/mm**	113.79±7.33**	108.31±8.50	0.000	112.13±10.33	106.17 ± 9.88	0.385	-0.047	0.039
**BL/mm**	96.33±5.80**	92.23±6.61	0.000	94.94±7.48	91.59 ± 9.28	0.279	-0.256	0.275
**BH/mm**	15.54±1.33**	14.60±1.56	0.000	15.30±1.95	14.13 ± 1.62	0.073	0.216	0.067
**BW/mm**	9.63±0.95**	8.97±1.14	0.000	9.43±1.28	8.74 ± 1.45	0.048	-0.171	0.045
**HL/mm**	15.83±1.29**	14.89±1.59	0.000	15.52±1.94	14.65 ± 1.78	0.072	-0.097	-0.109
**ED/mm**	2.73±0.25	2.71±0.26	0.313	2.72±0.25	2.75 ± 0.43	0.009	0.012	2.390
**CPL/mm**	15.56±1.90**	14.71±2.52	0.000	15.30±2.70	15.40 ± 2.68	0.101	-0.142	-0.815
**CPH/mm**	12.71±1.27**	11.80±1.51	0.000	12.36±1.81	11.70 ± 1.67	0.067	-0.309	-0.278
**IL/mm**	46.99±4.70	46.88±4.59	0.085	46.91±4.86	45.96 ± 7.76	0.229	0.176	0.292

Note: ** representing a significant difference at 0.01 level.

Table S2 Pearson correlation coefficients among the traits

	TW	EBW	FL	BL	BH	BW	HL	ED	CPL	CPH	IL	WG	MC	SGD
**TW**	1	0.988**	0.932**	0.951**	0.886**	0.875**	0.882**	0.434**	0.659**	0.843**	0.652**	0.699**	0.623**	0.551**
**EBW**		1	0.916**	0.948**	0.862**	0.857**	0.870**	0.458**	0.678**	0.841**	0.655**	0.679**	0.603**	0.521**
**FL**			1	0.971**	0.948**	0.896**	0.942**	0.423**	0.762**	0.893**	0.710**	0.732**	0.667**	0.659**
**BL**				1	0.902**	0.868**	0.911**	0.434**	0.735**	0.859**	0.682**	0.694**	0.625**	0.578**
**BH**					1	0.918**	0.945**	0.453**	0.760**	0.890**	0.694**	0.730**	0.667**	0.699**
**BW**						1	0.896**	0.488**	0.721**	0.857**	0.653**	0.687**	0.625**	0.611**
**HL**							1	0.511**	0.769**	0.884**	0.725**	0.696**	0.633**	0.677**
**ED**								1	0.468**	0.461**	0.434**	0.289**	0.242**	0.282**
**CPL**									1	0.878**	0.704**	0.560**	0.509**	0.605**
**CPH**										1	0.735**	0.673**	0.620**	0.644**
**IL**											1	0.551**	0.497**	0.543**
**WG**												1	0.984**	0.523**
**MC**													1	0.503**
**SGD**														1

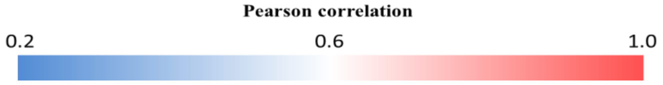

Note: ** representing a significant correlation at 0.01 level.

Table S3 Summary of the markers shared between female-specific and male-specific map

LG ID	Total marker	Shared marker interval (cM)			Female-to-male ratio of marker interval
		Female map	Male map	Sex-averaged map	
LG1	55	3.09	4.36	3.72	0.71
LG2	51	2.98	3.02	3.00	0.99
LG3	46	4.16	3.54	3.85	1.18
LG4	97	2.14	1.75	1.94	1.23
LG5	85	2.34	2.20	2.27	1.06
LG6	47	4.41	3.11	3.76	1.42
LG7	83	2.23	2.47	2.35	0.90
LG8	74	2.56	2.59	2.58	0.99
LG9	90	2.36	2.38	2.37	0.99
LG10	83	2.11	2.23	2.17	0.95
LG11	135	1.65	1.36	1.51	1.21
LG12	75	2.46	2.47	2.47	0.99
LG13	65	2.87	2.61	2.74	1.10
LG14	59	2.99	2.71	2.85	1.10
LG15	123	1.87	1.55	1.71	1.21
LG16	100	1.99	2.02	2.00	0.98
LG17	97	2.26	1.74	2.00	1.30
LG18	86	1.90	2.28	2.09	0.83
LG19	66	2.50	2.54	2.52	0.98
LG20	75	2.49	2.38	2.44	1.04
LG21	139	1.54	1.72	1.63	0.89
LG22	40	4.48	4.64	4.56	0.97
LG23	84	2.42	2.26	2.34	1.07
LG24	92	2.17	1.96	2.07	1.10
**Average**	/	2.58	2.50	2.54	1.03

Table S4 Summary of the markers shared between female-specific and male-specific map

	Mapped	Anchored	Oriented	UnAnchored
**Number of markers**	15,830	15,313	14,845	517
**Number of markers per Mb**	15.8	15.9	16.0	4.4
**Scaffold N50 (Mb)**	/	3.0	3.0	/
**Number of scaffolds**	1,127	774	478	2,626
**Number of scaffolds with 1 marker**	556	258	0	298
**Number of scaffolds with 2 markers**	69	45	39	24
**Number of scaffolds with 3 markers**	39	27	23	12
**Number of scaffolds with >=4 markers**	463	444	416	19
**Total length (Mb)**	1001.81	960.27	925.10	117.51
**Proportion of length in genome sequence**	93.0%	89.1%	85.8%	10.9%

Table S5 Description of the LGs and the anchored chromosomes

Chromosome ID	LG anchored	Chromosome size / Mb	LG length / cM	Spearman correlation coefficients
Chr01	LG01	35	208	0.991
Chr02	LG02	30	156	0.975
Chr03	LG03	45	191	0.963
Chr04	LG04	40	194	0.995
Chr05	LG05	57	209	0.980
Chr06	LG06	37	188	0.993
Chr07	LG07	37	196	0.996
Chr08	LG08	45	193	0.985
Chr09	LG09	42	211	0.983
Chr10	LG10	35	182	0.994
Chr11	LG11	45	202	0.985
Chr12	LG12	34	198	0.966
Chr13	LG08	45	193	0.503
	LG13	36	181	0.990
Chr14	LG14	27	172	0.992
Chr15	LG15	65	215	0.978
	LG21	59	226	0.637
Chr16	LG02	30	156	0.317
	LG16	39	205	0.996
Chr17	LG17	27	193	0.980
Chr18	LG18	31	188	0.997
Chr19	LG19	34	170	0.994
Chr20	LG20	34	187	0.991
Chr21	LG21	59	226	0.980
Chr22	LG17	27	193	0.848
	LG22	41	187	0.991
Chr23	LG23	51	199	0.989
Chr24	LG24	37	193	0.980
**Average**	/	40.1	193.5	0.927

Table S6 Summary of the potentially key markers and genes linked to growth and sex on LG11

Traits	Key markers	Candidate key genes
Sex determination	Marker51869	*Cyp19b, Vtg1, Lgals1*
	Marker282599	*Dmrt1, Irs1*
	Marker51904	*Acta2, Wap65, Dnd*
	Marker329989	/
Growth	Marker114865	*Cyp19b, Gh1*
	Marker172604	*Leo1, Adgrv1*
	Marker159479	/

Table S7 Summary of the sex-related candidate genes identified in *P. dabryanus*

Databases	Number of annotated genes			
	GD	WG	MC	SGD
NR	394	430	467	893
NT	394	430	467	893
trEMBL	387	425	461	881
SwissProt	248	277	303	563
GO	183	219	238	455
KEGG	187	226	243	478
COG	115	119	140	271
Total	394	430	467	893
